# Complex Loci in Human and Mouse Genomes

**DOI:** 10.1371/journal.pgen.0020047

**Published:** 2006-04-28

**Authors:** Pär G Engström, Harukazu Suzuki, Noriko Ninomiya, Altuna Akalin, Luca Sessa, Giovanni Lavorgna, Alessandro Brozzi, Lucilla Luzi, Sin Lam Tan, Liang Yang, Galih Kunarso, Edwin Lian-Chong Ng, Serge Batalov, Claes Wahlestedt, Chikatoshi Kai, Jun Kawai, Piero Carninci, Yoshihide Hayashizaki, Christine Wells, Vladimir B Bajic, Valerio Orlando, James F Reid, Boris Lenhard, Leonard Lipovich

**Affiliations:** 1 Computational Biology Unit, Bergen Center for Computational Science, University of Bergen, Bergen, Norway; 2 Programme for Genomics and Bioinformatics, Department of Cell and Molecular Biology, Karolinska Institutet, Stockholm, Sweden; 3 Genome Exploration Research Group (Genome Network Project Core Group), RIKEN Genomic Sciences Center, RIKEN Yokohama Institute, Yokohama, Japan; 4 Dulbecco Telethon Institute, Institute of Genetics and Biophysics CNR, Naples, Italy; 5 Department of Biological and Technological Research, San Raffaele Scientific Institute, Milan, Italy; 6 Fondazione Istituto FIRC di Oncologia Molecolare, Milan, Italy; 7 Department of Experimental Oncology, Istituto Europeo di Oncologia, Milan, Italy; 8 Knowledge Extraction Laboratory, Institute for Infocomm Research, Singapore; 9 South African National Bioinformatics Institute, University of the Western Cape, Bellville, South Africa; 10 Department of Biological Sciences, National University of Singapore, Singapore; 11 Chemical and Life Sciences, Nanyang Polytechnic, Singapore; 12 Genomics Institute of the Novartis Research Foundation, San Diego, California, United States of America; 13 Scripps Florida, Jupiter, Florida, United States of America; 14 Genome Science Laboratory, Discovery Research Institute, RIKEN Wako Institute, Wako, Japan; 15 School of Biomolecular and Biomedical Science, Eskitis Institute for Cell and Molecular Therapies, Griffith University, Brisbane, Queensland, Australia; 16 Department of Experimental Oncology, Istituto Nazionale per lo Studio e la Cura dei Tumori, Milan, Italy; 17 Genome Institute of Singapore, Singapore; The Jackson Laboratory, US; MRC-Harwell, UK; NHGRI-NIH, US; Lawrence Livermore National Laboratory, US; The Jackson Laboratory, US

## Abstract

Mammalian genomes harbor a larger than expected number of complex loci, in which multiple genes are coupled by shared transcribed regions in antisense orientation and/or by bidirectional core promoters. To determine the incidence, functional significance, and evolutionary context of mammalian complex loci, we identified and characterized 5,248 *cis–*antisense pairs, 1,638 bidirectional promoters, and 1,153 chains of multiple *cis–*antisense and/or bidirectionally promoted pairs from 36,606 mouse transcriptional units (TUs), along with 6,141 *cis–*antisense pairs, 2,113 bidirectional promoters, and 1,480 chains from 42,887 human TUs. In both human and mouse, 25% of TUs resided in *cis–*antisense pairs, only 17% of which were conserved between the two organisms, indicating frequent species specificity of antisense gene arrangements. A sampling approach indicated that over 40% of all TUs might actually be in *cis–*antisense pairs, and that only a minority of these arrangements are likely to be conserved between human and mouse. Bidirectional promoters were characterized by variable transcriptional start sites and an identifiable midpoint at which overall sequence composition changed strand and the direction of transcriptional initiation switched. In microarray data covering a wide range of mouse tissues, genes in *cis–*antisense and bidirectionally promoted arrangement showed a higher probability of being coordinately expressed than random pairs of genes. In a case study on homeotic loci, we observed extensive transcription of nonconserved sequences on the noncoding strand, implying that the presence rather than the sequence of these transcripts is of functional importance. Complex loci are ubiquitous, host numerous nonconserved gene structures and lineage-specific exonification events, and may have a *cis-*regulatory impact on the member genes.

## Synopsis

In the traditional view, most genes occupy their own distinct territory in mammalian genomes. However, it has become apparent that many genes are in fact located in complex regions (complex loci) where they share territory with other genes by utilizing opposite strands of DNA. Such genes either share regions expressed as mRNA (i.e., form *cis–*antisense pairs) or start from a genome region (called a bidirectional promoter) at which transcription can initiate in both directions along the DNA. In this paper, researchers present the one of the most comprehensive censuses of complex loci to date and investigate their general properties and human–mouse differences to discover the rules of this type of gene organization and its effect on gene regulation. They found about 25% of known human and mouse genes to be in *cis–*antisense pairs, and estimate the total fraction to be over 40%. At bidirectional promoters, they demonstrated the existence of mirror DNA sequence composition related to the promoters' ability to initiate transcription in two directions. The researchers found over 2,000 “chains”*—*complex arrangements where three or more genes are coupled by *cis–*antisense pairing and/or bidirectional promoters; among them are many genes whose products control the expression of other genes.

## Introduction

Several recent reports indicate that the transcriptional complexity of mammalian genomes has been significantly underestimated. Large-scale sequencing of full-length transcripts, expressed sequence tags (ESTs), and shorter tags [1] and transcriptional maps constructed by the use of tiling arrays [2–5] demonstrate that human and mouse genomes contain an abundance of complex loci with overlapping transcription on the two DNA strands. Although individual complex loci have been described in detail [6–8], a global description of the general properties of gene arrangements within such complex loci is lacking.

Two types of *cis-*coupling of genes have been reported to be widespread in mammalian genomes. (1) More than a thousand pairs of divergently transcribed, nonoverlapping genes spaced by less than 1,000 bp have been found in the human genome, comprising 9% of known genes [9]. The genes in such a pair typically share a bidirectional promoter. (2) Numerous pairs of oppositely transcribed genes whose exons overlap in the genome (*cis–*antisense pairs) have been identified in human and mouse genomes [10,11]. Human cDNA and EST data indicate that 22% of transcripts are involved in *cis–*antisense pairs [12]. Data from tiling array experiments and sequencing of short tags representing 5′- and 3′-ends of transcripts suggest that *cis–*antisense pairs might be even more widespread, perhaps involving more than 60% of all loci [4,13]. For both bidirectionally promoted pairs and *cis–*antisense pairs, there is evidence that paired genes tend to be coexpressed [9,11,13–15]. In some bacteria, it is well established that natural antisense transcripts from *cis–*antisense pairs can regulate expression of the gene encoded on the opposite strand (for review, see [16]). Numerous case studies suggest that *cis-*encoded natural antisense transcripts are important regulators in eukaryotes as well, potentially affecting a range of processes including transcription, imprinting, DNA methylation, and RNA splicing, editing, and degradation (for review, see [17,18]).

Cross-species genome comparisons can reveal conserved genomic features that are likely to be functionally important, and species-specific features that might underlie phenotypic differences. For the great majority (81%) of human bidirectionally promoted pairs where the genes have mouse orthologs, the bidirectional arrangement is conserved, suggesting that it is functionally important [9]. Similarly, for human *cis–*antisense pairs where the genes have orthologs in pufferfish, proximity and orientation of the paired genes is conserved in pufferfish significantly more often than for pairs of neighboring genes on the same strand [19]. However, evidence for cross-species conservation of actual overlapping arrangements of genes has been more limited. Searches for human–mouse orthologs that form *cis–*antisense pairs in both organisms have previously reported at most 347 gene pairs [19–21], a very small number compared to the thousands of species-specific pairs found. In addition, the actual exon overlaps within *cis–*antisense pairs have been reported to lack elevated conservation in general [20], contrary to the hypothesis that blocks of conservation in untranslated regions (UTRs) and extreme conservation in translated regions of transcripts indicate antisense regulation [22]. A limitation of the aforementioned comparative studies of *cis–*antisense pairs might have been their exclusive focus on protein-coding genes: recent unbiased surveys of mouse transcripts have indicated that *cis–*antisense pairs most frequently consist of one coding and one noncoding transcript [11,13].

We define complex loci as genomic regions in which multiple genes share transcribed regions in antisense orientation and/or bidirectional core promoters. In this study we construct comprehensive and highly reliable genome-wide datasets of *cis–*antisense and bidirectionally promoted gene pairs from human and mouse transcript sequence data and present an analysis of the higher-level organization of these pairs in complex loci. We further explore human–mouse conservation of complex loci at both sequence and structure levels, taking into account both coding and noncoding transcripts. We describe a widespread occurrence of “chains” of overlapping transcriptional units (TUs), a several times greater number of human–mouse conserved *cis–*antisense pairs than previously reported, and additional species-specific complex arrangements. We perform sampling to reach an estimate of the total fraction of genes in *cis–*antisense arrangement, and of the fraction of such arrangements that are conserved between human and mouse. We study the sequence composition of bidirectional promoters and its relation to the positioning of transcriptional start sites (TSSs). Finally, we take a closer look at a number of homeotic genes, to assess the extent of transcription from the opposite strand at these loci and the conservation of the transcripts.

## Results

### *cis–*Antisense Pairs and Bidirectional Promoters Are Abundant in Mammalian Genomes

We inferred TUs from genomic mappings of EST and full-length cDNA sequences from FANTOM3 and the public databases. Particularly rigorous criteria were applied to thoroughly eliminate the inclusion of artificially reversed sequences (see Materials and Methods). This is straightforward for spliced sequences, since their orientation can be verified by sequence motifs at splice junctions. We assessed the performance of the part of the procedure that handles mappings of unspliced sequences. Mappings based on EST sequence only (EST mappings) were treated more stringently than mappings with cDNA support (cDNA mappings), because of the higher quality and higher methodological reliability of transcript strand annotation of the latter set. We estimate that the procedure, when applied to human data, correctly determined the orientation of 99.8% of unspliced cDNA mappings and 99.8% of unspliced EST mappings. Tests on mouse data gave very similar estimates (Figure S1). Only 0.09% of unspliced human and mouse cDNA mappings were rejected, but 52% of unspliced human EST mappings and 34% of unspliced mouse EST mappings were rejected because of insufficient information on original strand orientations. The lower rejection rate for mouse EST mappings largely reflects higher availability and consistency of read direction (5′/3′) annotation for mouse EST sequences, where a higher proportion of ESTs were produced using cap trapping technology [23].

Starting from 161,805 human and 140,769 mouse cDNA sequences and a total of ~8 million ESTs, we obtained 42,887 human and 36,606 mouse TUs (Table 1). Using the estimated reversal rates and the empirical genomic distribution of mappings, we performed simulations that indicated that the frequency of false TUs due to mapping reversal was about one in 1,000 for human and about one in 800 for mouse (see “Accuracy Assessment of Orientation Procedure” in Materials and Methods). This would yield about 40 false human and 50 false mouse TUs, which is an acceptable rate that would not impact the conclusions of any of the further analyses we performed.

**Table 1:**
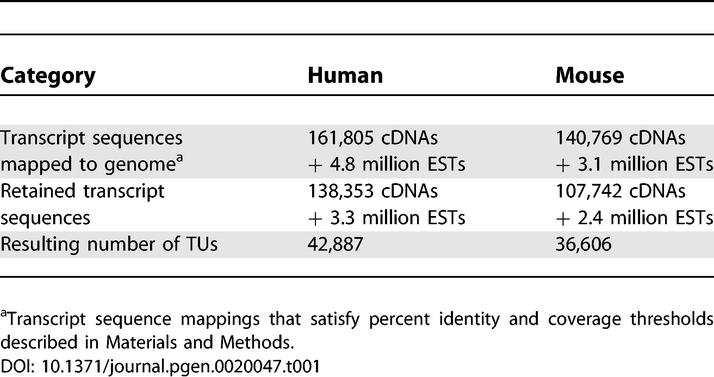
Numbers of cDNA and EST Sequences Used and Resulting TUs

#### cDNA and EST sequences support involvement of at least 25% of all TUs in *cis–*antisense pairs and 9% of all TUs in bidirectionally promoted pairs.

**Figure 1:**
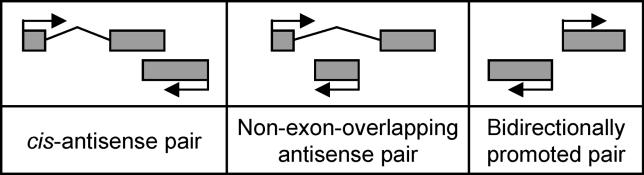
TU Pairs Searched For We defined a *cis–*antisense pair as two oppositely transcribed TUs that share at least 20 bp of exon sequence, a non-exon-overlapping antisense pair as two oppositely transcribed TUs that overlap by at least 20 bp, but not within exons, and a bidirectionally promoted pair as two divergently transcribed TUs that overlap by less than 20 bp and are less than 1,000 bp apart.

Nearly half of all TUs were involved in one or more of the types of bidirectional transcription defined in Figure 1: *cis–*antisense pairs, non-exon-overlapping antisense pairs, and bidirectionally promoted pairs (Table 2). The most common arrangement was the *cis–*antisense pair, which involved 25% of TUs in both human and mouse. Putative bidirectionally promoted pairs involved 9%–10% of TUs and were also roughly equally frequent in the two organisms. On the other hand, the raw frequency of non-exon-overlapping antisense pairs differed between human and mouse. Since TUs in non-exon-overlapping antisense pairs need not share exon sequence similarity, this dataset may contain a number of TUs that are artificially nested because of genome assembly and transcript sequence mapping errors. For this reason, the subsequent analysis focused on *cis–*antisense pairs and bidirectionally promoted pairs.

**Table 2:**
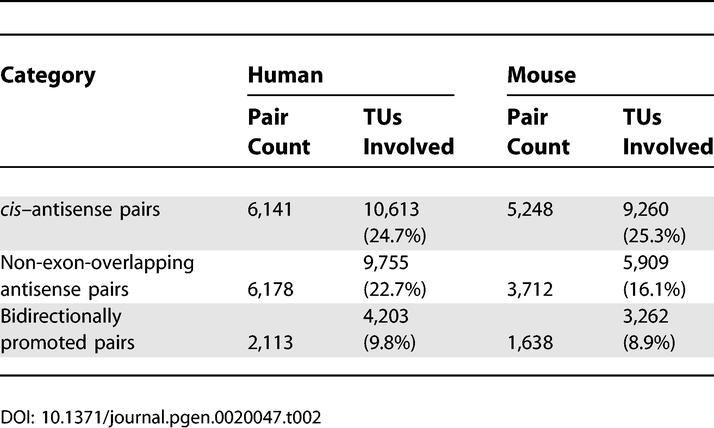
Numbers of TU Pairs Detected and TUs Involved

To further investigate the reliability of the *cis–*antisense pair dataset, we categorized the pairs based on the types of mappings supporting exon overlaps (Figure S2). Most pairs (human: 60%; mouse: 58%) were supported by spliced mappings on both strands. The great majority of *cis–*antisense pairs (human: 78%; mouse: 88%) were supported by sequence types generally considered to be of high quality (either cDNA or spliced EST mappings), indicating that our set of *cis–*antisense pairs is highly reliable.

#### RT-PCR validation of a sample of *cis–*antisense pairs suggests that at least 80% are expressed from both strands.

**Figure 2:**
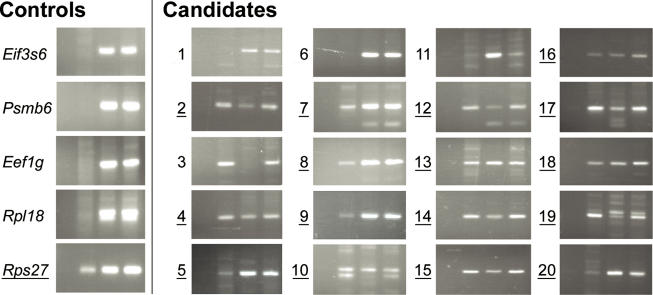
Validation of the Expression of Randomly Selected *cis–*Antisense Pairs by RT-PCR To confirm the expression of complementary transcripts, we performed orientation-specific RT-PCR as described previously [12,24]. Primers were designed to amplify regions of exon overlap. For each candidate or control, four RT-PCR reactions (corresponding to the four lanes in each gel image) were carried out using adult mouse brain RNA as template. Orientation specificity was achieved by restricting which primers were present during reverse transcription single-strand synthesis: no primer (first lane), only sense primer (second lane), only antisense primer (third lane), and both sense and antisense primers (fourth lane). In all reactions, both primers were present during the subsequent PCR reactions. For candidates, sense and antisense primers were designed with respect to the genomic plus strand. For controls, primers were designed with respect to the control transcript. Out of five highly expressed control genes with no evidence of antisense transcription in sequence databases, we detected antisense transcription for one *(Rps27)*. We reproducibly observed evidence of anti-*Rps27* transcripts using two different primer pairs (unpublished data). We tested 20 *cis–*antisense pairs from our computationally constructed dataset and detected expression of both strands for 16 (underlined). For one additional *cis–*antisense pair (number 11), the result was ambiguous because of the presence of many bands of unexpected size. The 20 *cis–*antisense pairs were selected at random from the mouse dataset, with the requirements that exon overlaps be at least 200 bp (to allow amplicons of at least 100 bp) and that there be at least one cDNA or EST from adult brain supporting the exon overlap on each strand.

To experimentally assess the validity of our *cis–*antisense pair dataset, we performed orientation-specific RT-PCR as previously described [12,24]. We investigated the expression in adult mouse brain of complementary transcripts corresponding to 20 randomly selected *cis–*antisense pairs supported by at least one cDNA or EST from adult mouse brain on each strand. As negative controls, we selected five highly expressed genes for which we could find no evidence of antisense transcription in sequence databases. We were able to detect the coexpression of sense and antisense transcripts in brain for 16 of the 20 *cis–*antisense pairs (Figure 2). For one of the remaining pairs, the result was ambiguous because of the presence of many additional bands of unexpected sizes. One of the negative controls *(Rps27)* also reproducibly showed evidence of antisense transcription. In retrospect, this control was ill-chosen: there are several copies of *Rps27* pseudogenes in the mouse genome, so the complementary transcripts need not be transcribed from the same loci as *Rps27* itself. In conclusion, the RT-PCR results suggest that at least 80% of the *cis–*antisense pairs in our dataset are expressed from both strands, and that there might exist a significant number of antisense transcripts that are yet to be discovered.

#### Properties of *cis–*antisense overlaps are highly similar between human and mouse.

Many *cis–*antisense pairs in our set (34% of human pairs and 34% of mouse pairs) had multiple distinct exon-to-exon overlaps. Both genomes had on average 1.6 distinct exon-to-exon overlaps per *cis–*antisense pair. The size distributions for exon overlaps ranged from 1 bp to 5,200 bp with medians of 159 bp (human) and 172 bp (mouse), and distinct peaks around 100 bp. The average repeat content within exon overlaps was 9.5% in human and 5.9% in mouse. For comparison, we measured the repeat content within the entire exonic sequence of each TU. The average repeat content of entire TUs was nearly three times as high for TUs in general (25% and 17% of exon sequence for human and mouse, respectively), and nearly two times as high for TUs involved in *cis–*antisense pairs (human: 17%; mouse: 12%). We therefore concluded that *cis–*antisense pairs tend to involve TUs with low repeat content, and that exon overlaps tend to be located in repeat-poor regions of those TUs. However, for a subset of *cis–*antisense pairs (human: 213; mouse: 53), more than 90% of the exon overlap was repeat sequence. (Here it should be noted that there might be an underrepresentation of repeat-rich transcripts in the dataset because of the inherent difficulty of unambiguously mapping them onto the genome.)

We classified the *cis–*antisense pairs—based on transcriptional direction of participant TUs—as divergently transcribed (head-to-head overlapping), convergently transcribed (tail-to-tail overlapping), or fully overlapping (one TU completely spanned by the other). In agreement with previous observations on the FANTOM3 dataset [13], we found these three classes to be roughly equally common in mouse (Table S1). In human, divergent and convergent *cis–*antisense pairs were also roughly equally common, but fully overlapping pairs were more frequent, constituting 42% of all pairs. A significant number of these fully overlapping pairs might represent actual divergent/convergent cases that were not detected as such because of the lower availability of full-length cDNA sequence for human.

#### Over 40% of all TUs might be involved in *cis–*antisense pairs.

**Figure 3:**
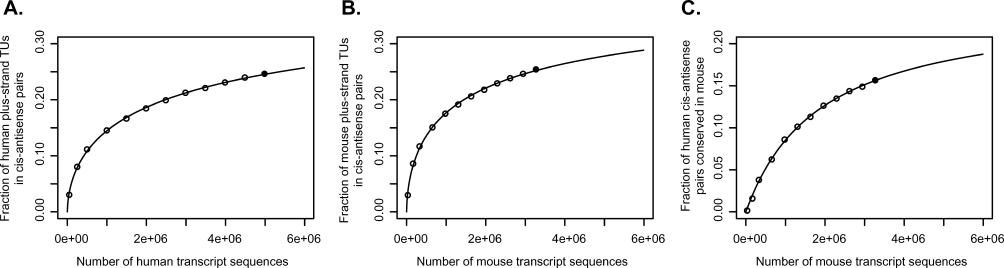
Estimating the Extent and Conservation of Antisense Transcription (A and B) Estimation of proportion of TUs involved in *cis–*antisense pairs. Open circles indicate the fraction of all human TUs on the plus strand (A) and all mouse TUs on the plus strand (B) that were found to be involved in *cis–*antisense pairs when the minus-strand TUs were recomputed starting from random transcript sequence samples of different sizes. Filled circles represent the full datasets based on all available transcript sequences. The saturation curves (see Equation 1) indicated by the lines fit almost perfectly to the sampled data. Fitted human and mouse saturation curves approach 0.45 and 0.43, respectively, as the number of transcript sequences increases, indicating that more than 40% of all TUs might be involved in *cis–*antisense pairs. Similar estimates were obtained by other sampling approaches (Figure S3). (C) Estimation of the proportion of human *cis–*antisense pairs that are conserved in mouse. Open circles indicate the proportion of human *cis–*antisense pairs found to be conserved in mouse when the full human dataset was compared to mouse datasets recomputed from random mouse transcript sequence samples of different sizes. The same type of saturation curve as in (A) was fitted to the data. Here, a model with *c* = 1 (i.e., hyperbolic saturation) was preferable as it provided an equally good fit while being simpler. The fitted curve approaches 0.25 as the number of mappings grows, indicating that about 25% of human *cis–*antisense pairs are conserved in mouse.

To estimate the true proportion of TUs that are involved in *cis–*antisense pairs, we recomputed the TU and *cis–*antisense pair datasets using random subsets of all available transcript sequences. Figure 3A and 3B show the fraction of TUs we observed to be involved in *cis–*antisense pairs as a function of the number of transcript sequences used. For both human and mouse, a saturation curve

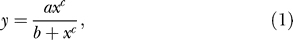
fitted almost perfectly to the sampled data: here *a* is the fraction of TUs involved in *cis–*antisense pairs at saturation, and *c* (the equivalent of the Hill coefficient) is a measure of sequence redundancy in the set and depends on the choice of sampled set (e.g., all transcripts or only one sequence per cDNA clone). The saturation curves predicted that the fraction of TUs involved in *cis–*antisense pairs approaches 0.45 for human and 0.43 for mouse as the number of transcript sequences increases. Using two other sampling approaches, we obtained closely similar estimates (Figure S3). Thus, based on the current data, over 40% of human and mouse TUs might eventually be found to be involved in *cis–*antisense pairs if transcript sequencing continues.

#### Nearly 1,000 *cis–*antisense pairs are conserved between human and mouse.

As noted above, we found a striking agreement between human and mouse in prevalence and general properties of *cis–*antisense pairs. We proceeded to assess the agreement between the human and mouse datasets at the individual pair level. First, we counted the number of human and mouse *cis–*antisense pairs that had exon overlaps in corresponding positions in a BLASTZ net alignment of the two genomes (alignments were obtained from the University of California Santa Cruz (UCSC) Genome Browser Database [25]; see Materials and Methods). There were 962 such pairs in human, and 943 corresponding pairs in mouse, constituting 16% and 18% of all human and mouse *cis–*antisense pairs, respectively (Table S2). The human and mouse numbers differ slightly because a small proportion of mouse pairs corresponded to several human pairs and vice versa. We consider this a strict assessment of conservation, because exon overlaps were required to be in corresponding places (implying conserved structure). However, we did not set any explicit sequence conservation threshold, since sequence might not be of primary importance for antisense regulation and previous work has indicated that antisense overlaps do not tend to have elevated sequence conservation [20]. The majority of *cis–*antisense pairs (69% of human pairs and 82% of mouse pairs) had more than 90% of their exon overlap sequence within BLASTZ net alignments, indicating that the implicit requirement for sequence similarity imposed by the use of precomputed alignments did not severely limit our ability to detect conserved *cis–*antisense pairs. However, it is likely that a large number of truly conserved pairs were not detected as such because of transcript sequences that have not been discovered yet. We attempted to estimate the true extent of conservation of *cis–*antisense pairs by a sampling approach equivalent to the one we employed above to estimate the fraction of TUs involved in *cis–*antisense pairs. To estimate how observed conservation grows with increasing transcript sequence data, we compared the entire human dataset against mouse datasets computed from different-sized random samples of all available mouse transcript sequences (Figure 3C). The same type of saturation curve as used above fit well to the data. Here, a curve with *c* = 1 (i.e., a hyperbolic saturation model) was preferable as it provided an equally good fit while being simpler. The curve predicts that up to about 25% of human *cis–*antisense pairs are conserved in mouse per the definition of conservation employed here. To estimate whether mouse *cis–*antisense pairs are likely to be conserved at a similar rate in human, we repeated the analysis in an analogous manner, sampling human transcripts instead of mouse transcripts. A hyperbolic saturation model again fit well to the data and predicted that about 26% of mouse *cis–*antisense pairs are conserved in human at the saturation level (unpublished data).

#### Several conserved genes are in *cis-*antisense or bidirectionally promoted arrangement with nonconserved TUs.

Our saturation estimates indicated that most *cis–*antisense pairs are not conserved between human and mouse. Accordingly, detailed inspection of homeotic and other transcription factor loci provided several examples of nonconserved *cis–*antisense and bidirectionally promoted transcripts, some with experimentally supported regulatory roles (see below). We therefore wanted to examine the genome-wide occurrence of nonconserved transcripts in *cis–*antisense or bidirectionally promoted arrangement with known genes. To find such TUs in the *cis–*antisense pair dataset, we focused on the subset of *cis–*antisense pairs where one member (the known gene) had detectable conservation outside the region of antisense overlap, and the other member (the nonconserved TU) showed no conservation outside the region of overlap. Among all 3,442 divergent and convergent *cis–*antisense pairs in mouse (Table S1), there were only 50 pairs that fulfilled this criterion. We applied the same analysis to bidirectionally promoted pairs. Of the 1,638 bidirectionally promoted pairs in mouse, 40 fulfilled our criterion for conservation of one member only. (We did not perform this analysis on fully overlapping *cis–*antisense pairs because of difficulties in attributing conservation to individual genes that are completely overlapped by another gene.) Thus, we identified a total of 90 nonconserved TUs in bidirectionally promoted or *cis–*antisense arrangement with known genes.

**Figure 4:**
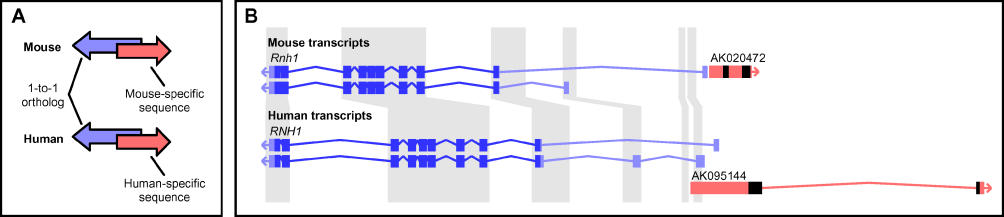
Positional Equivalents (A) Schematic depiction of positional equivalents. By positional equivalents (red arrows), we mean mouse and human TUs that are at genomically equivalent locations relative to well annotated genes at orthologous loci (blue arrows), but that do not share sequence similarity. (B) Positional equivalents divergently transcribed with the putative tumor suppressor *RNH1* [48]. Two transcript isoforms of *RNH1* are shown for both mouse (top) and human (bottom). A mouse TU supported by cDNA AK020472 shares a putative bidirectional promoter with *Rnh1*. The human equivalent (cDNA AK095144) is head-to-head *cis–*antisense to *RNH1*. Regions with gray background are within a BLASTZ net alignment of the two genomes. For *Rnh1* and *RNH1,* protein-coding sequence is indicated in dark blue and UTRs in light blue. The positional equivalents lack sequence conservation, assessed by BLASTZ net coverage and BL2SEQ alignment of transcripts, demonstrate gene structure differences, and contain lineage-specific repeats (indicated in black).

These TUs may represent either lineage-specific transcripts, or instances where the location of transcription is conserved between human and mouse, but the transcribed sequence is not. We use the term positional equivalents to refer to the latter: human and mouse TUs that are at genomically equivalent locations relative to well-annotated genes at orthologous loci, but that do not share sequence similarity (Figure 4A). The evidence for positional equivalents was limited, resulting in 16 manually curated positional equivalents involved in *cis–*antisense pairs, and a further 17 sharing bidirectional promoters with known genes (Table S3). A representative mouse TU with a human positional equivalent is shown in Figure 4B. The 33 identified positional equivalents showed no or weak evidence of protein-coding potential (Table S3). Additionally, their transcribed regions had often been modified substantially after species divergence, via species-specific insertions of repeat elements: 17/33 (52%) mouse TUs with human positional equivalents contained rodent-specific B1–B4 SINEs, and 13/33 (39%) human TUs contained primate-specific Alu SINEs and MER1 elements. In six cases there were both primate-specific repeats in human transcripts and rodent-specific repeats in the corresponding mouse positional equivalents.

### Broad Transcriptional Start Regions and a Mirror Sequence Composition Define Midpoints of Bidirectional Promoters

#### Bidirectional promoters are associated with broad transcriptional start regions.

**Figure 5:**
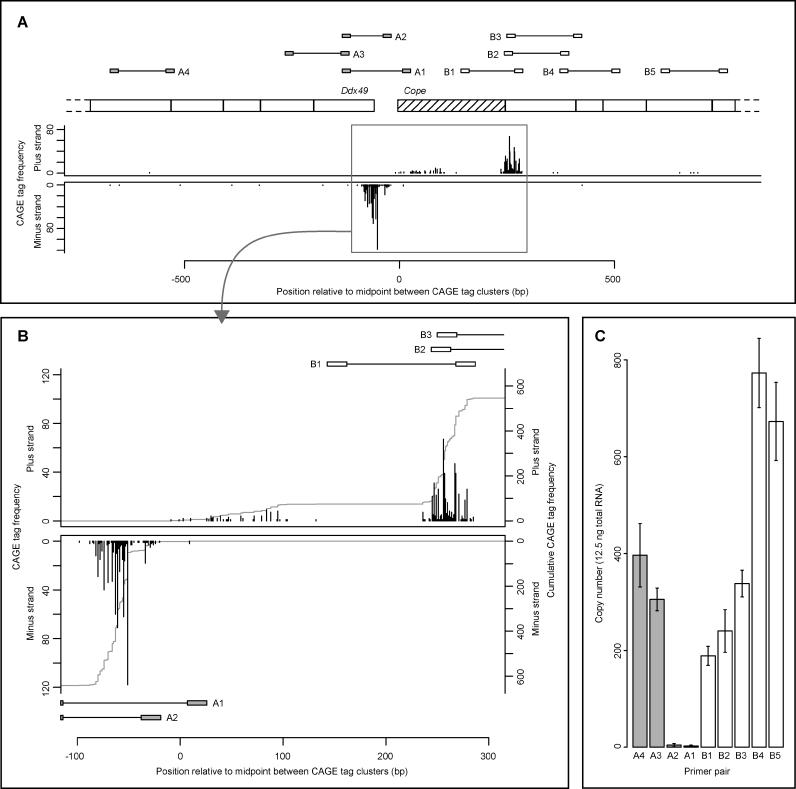
TSS Variability at the *Ddx49*/*Cope* Bidirectional Promoter in Mouse (A) The charts show the distribution of CAGE tag 5′-ends over the first five exons of each of the two genes *Ddx49* and *Cope,* and over their intergenic region. CAGE tag mappings indicate that transcription of *Cope* can start within two wide regions in the first exon of the gene. The initial part of this first exon (hatched) has support from several ESTs, but no cDNA sequences. The three large TCs at the *Ddx49*/*Cope* locus span 79, 114, and 150 bp, indicating great variability of transcriptional initiation within each cluster. To confirm the existence of such variability by qRT-PCR, primers (connected boxes) were designed to measure expression of selected regions of the *Ddx49* (primer pairs A1–A4) and *Cope* (primer pairs B1–B5) transcripts. (B) Detailed view of CAGE tag frequencies and primer locations over the three transcription initiation regions indicated by CAGE tags. Gray lines show cumulative CAGE tag frequencies. (C) Expression levels of different regions of the *Ddx49* and *Cope* transcripts in adult brain RNA as measured by qRT-PCR. Primer pairs A1 and A2 confirmed low level of expression of the longest *Ddx49* transcripts indicated by CAGE (copy numbers in 12.5 ng of total RNA were 3.2 [standard deviation = 1.1] and 5.1 [standard deviation = 3.0] for A1 and A2, respectively). Primer pair B1 confirmed transcription of *Cope* from upstream of the canonical initiation region. Primer pairs B2–B4 supported variability of transcriptional initiation within the canonical region.

Analysis of cap analysis of gene expression (CAGE) data has confirmed two major types of TSS regions associated with different types of core promoters (P. Carninci, A. Sandelin, B. Lenhard, D. A. Hume, Y. Hayashizaki, et al., unpublished data). TATA-box promoters typically initiate transcription from a single position in the genome, while TATA-less promoters can initiate transcription within an interval of 100 bp or more that often coincides with a CpG island [26]. We assembled a dataset of putative bidirectional promoters in the mouse genome well supported by CAGE tag data, and analyzed their genome-wide sequence properties. Bidirectional promoters were identified by scanning for pairs of divergently oriented CAGE tag clusters (TCs) (see Materials and Methods). Our final set consisted of 766 bidirectional promoters, each defined by a divergent TC pair at a separation up to 500 bp. Compared to a control set of 8,056 unidirectional promoters, the bidirectional promoter TCs showed a markedly larger dispersion of CAGE-determined TSS locations (Figure S4). Consistent with this finding, bidirectional promoters were associated with CpG islands more often than were unidirectional promoters (94% of bidirectional promoter TCs were CpG-island-associated, compared to 60% of unidirectional promoter TCs; *p* < 2.2 × 10^−16^, Chi-squared test). In addition, CpG islands associated with bidirectional promoters were significantly larger than CpG islands associated with unidirectional promoters (median CpG island sizes of 760 and 557 bp, respectively; *p* < 2.2 × 10^−16^, Wilcoxon rank sum test). To experimentally confirm the observed size of transcriptional initiation regions in bidirectional promoters, we used quantitative real-time PCR (qRT-PCR) to measure expression levels in mouse brain RNA samples of different regions near the 5′-ends of transcripts from the genes *Ddx49* and *Cope,* which share a bidirectional promoter (Figure 5). For *Ddx49,* we could confirm a very low level of expression of the longest transcripts, and much higher expression levels of transcripts initiated further downstream. For *Cope,* we could confirm great variability within the canonical TSS region, and the existence of an alternative upstream TSS region. Figure 5 demonstrates that the real-time PCR results support the observed distribution of CAGE tags, confirming the breadth of transcription initiation regions and relative TSS usage within them.

#### Bidirectional promoters display a mirror sequence composition.

**Figure 6:**
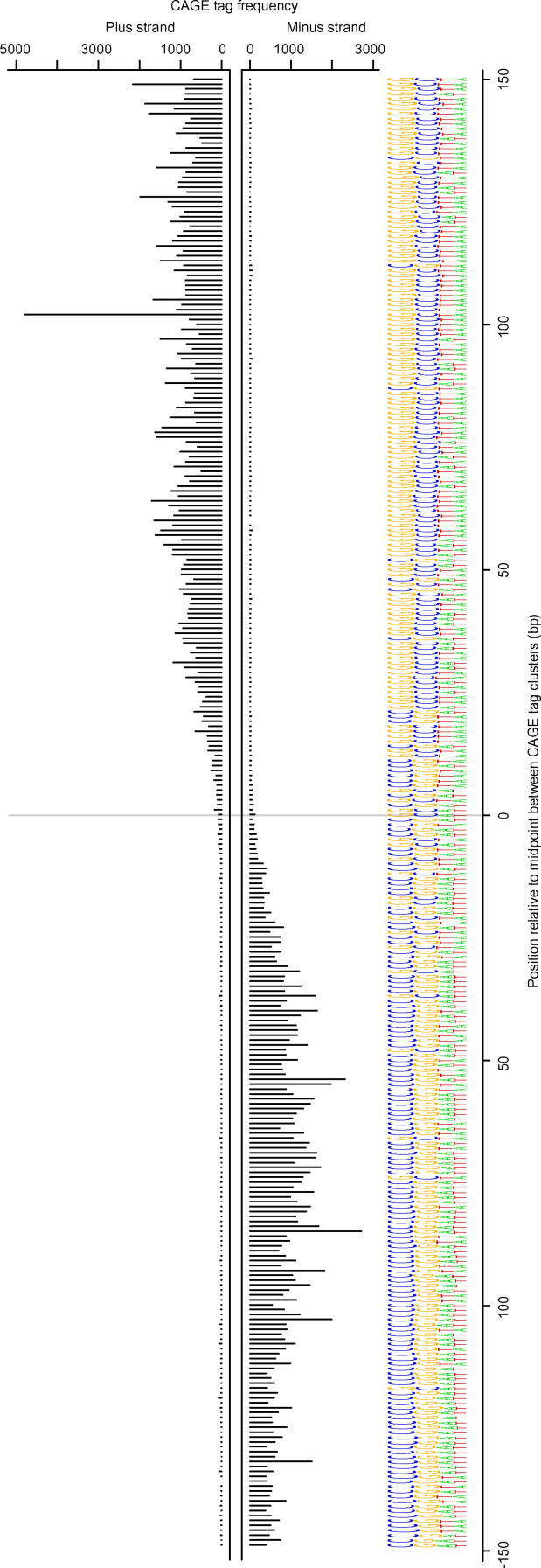
Landmark Sequence Composition of Bidirectional Promoters We defined the midpoint of a bidirectional promoter as the midpoint between the most 5′ TSS in each of the two divergently oriented TCs defining the bidirectional promoter. Sequences corresponding to the region spanned by the TCs were extracted from the genomic plus strand. All bidirectional promoter sequences were aligned at their midpoint and the logo created with WebLogo [49]. The logo displays the four nucleotides ranked by their frequency at each position, so that more common nucleotides appear above less common ones. The charts above the logo show the distribution of CAGE tag 5′-ends mapping to the plus strand (upper chart) and minus strand (lower chart) around bidirectional promoter midpoints. The CAGE tag distribution was computed as the sum of tag counts at each position over all bidirectional promoters. The peak of nearly 5,000 tags on the plus strand is due to the *Rps2* gene, which appears to be most highly expressed from a single TSS.

The two divergently oriented transcription start regions identifying a bidirectional promoter were generally closely spaced, but for only 12% of bidirectional promoters did the TCs overlap by one or more bases (Figure S4). To investigate an association between the separation of divergent TCs and the sequence composition of bidirectional promoters, we aligned the entire set of bidirectional promoter sequences at the midpoint between the TCs and visualized the result as a compositional sequence logo (Figure 6). On the genomic plus strand, there was an apparent excess of cytosines to the left of the midpoint, and a corresponding excess of guanines to the right of the midpoint. There was also small excess of adenines to the left of the midpoint and a corresponding excess of thymines to the right of the midpoint. This mirror-image sequence composition is a landmark of bidirectional promoters, making them markedly different from unidirectional CpG-island-overlapping promoters or random genomic regions (Figure S5).

CpG islands often contain multiple binding sites for the transcription factor Sp1 [26]. Considering that the Sp1 binding consensus motif is GGGGCGGGGT [27], the bias in guanine and thymine frequencies we observed across the midpoints of bidirectional promoters would be consistent with a corresponding bias in directionality of Sp1 binding. We scanned the region to the right of bidirectional promoter midpoints for putative Sp1 sites and found 43% more sites on plus strands than on minus strands. The relationship was reversed to the left of the midpoint (60% more binding sites on minus strands than on plus strands). On both sides of the midpoint, both plus and minus strands were significantly enriched for putative Sp1 binding sites compared to random sequences with the same lengths and background nucleotide frequencies (emitted from a first-order Markov chain to preserve dinucleotide composition) (Figure S6). This supports the idea of Sp1 as the probable key general transcriptional factor that binds to CpG island promoters [26].

### Coexpressed *cis–*Antisense Pairs Display Conserved Overlaps Containing Noncanonical TSSs

**Figure 7:**
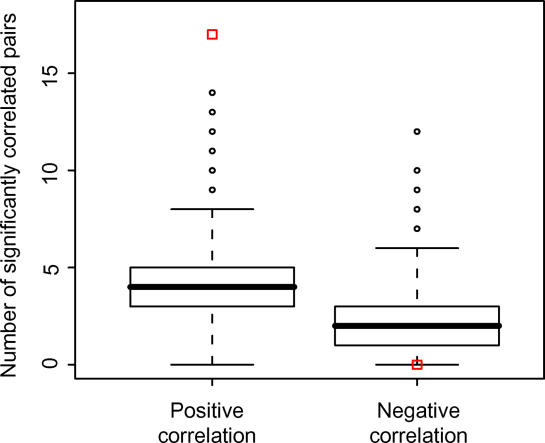
Members of *cis–*Antisense Pairs Have Positively Correlated Expression Profiles More Often than Expected by Chance Out of 242 murine *cis–*antisense pairs with expression data for 61 tissues, 17 showed significant positive correlation across the entire set of tissues after correction for multiple testing, and no pairs showed significant negative correlation (red squares). The same test was applied to 10,000 sets of 242 random TU pairs (box plots, with circles indicating outliers), demonstrating that members of *cis–*antisense pairs have positively correlated expression profiles more often than expected by chance.

To pinpoint *cis–*antisense pairs with regulatory interactions between pair members, we concentrated on 242 pairs from mouse with available microarray expression data for 61 tissues (GNF1M data; [28]). We selected only probesets that mapped to regions of exon overlap between *cis–*antisense partners, in order to avoid detecting mixed signal from antisense-overlapping and nonoverlapping transcript isoforms. The majority (84%) of pairs with available probesets were convergently (tail-to-tail) overlapping. We found a significant positive correlation across the entire panel of tissues for 58/242 (24%) *cis–*antisense pairs at the 0.05 level, and a significant negative correlation for only 14/242 (6%) pairs. After correcting for multiple testing, 17/242 (7%) pairs remained significantly positively correlated, and no pairs remained significantly negatively correlated. We assessed how likely it would be to obtain this result if TUs were paired at random. In only three out of 10,000 sets of 242 random TU pairs did we obtain 17 or more significantly correlated pairs, and none of the sets contained more than 14 significant positive correlations (Figure 7). By the same methodology, members of bidirectionally promoted pairs were also found to have positively correlated expression profiles more often than would be expected by chance (unpublished data). The 17 *cis–*antisense pairs identified as positively correlated all belonged to the convergent class (Table S4). For 15 of these pairs, the overlap included UTRs at the 3′-ends of both TUs. Of these exon overlaps at apparent noncoding regions, 11 contained stretches of high conservation. In three cases, this conservation was clearly limited to the overlap region (Table S4), indicating possible functional importance of exon overlaps [22]. Inspection of CAGE tag mappings to the 17 positively correlated *cis–*antisense pairs revealed that for 13 pairs there was evidence of TSSs at one or both of the 3′-ends involved in the overlap (Table S4). The largest 3′-end TC (supported by 18 tags) among coexpressed *cis–*antisense pairs was observed in the 3′-UTR of *Ppp1ca,* which encodes a catalytic subunit of a protein phosphatase required for cell division. The 3′-UTR of *Ppp1ca* is conserved in human, and in both genomes overlaps by about 220 bp the 3′-UTR of *Rad9,* which encodes a cell-cycle checkpoint protein required for DNA damage repair [29].

### Chains of Overlapping TUs Occur in Gene-Dense Areas with Antisense Transcription

**Figure 8:**
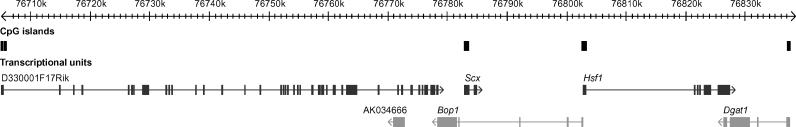
A Five-TU Chain on Mouse Chromosome 15 TUs on the genomic plus and minus strands are shown in dark gray and light gray, respectively (boxes represent exons). CpG islands are shown as black boxes. From left to right, the chain contains a member of the aminoacyl tRNA transferase class II family *(D330001F17Rik),* which has two *cis–*antisense transcripts: fully overlapping (cDNA AK034666) and convergent *(Bop1)*. The latter encodes a ribosome biogenesis protein and shares a CpG-island bidirectional promoter with the heat-shock-induced transcription factor 1 gene *(Hsf1)*. *Hsf1,* in turn, is convergently *cis–*antisense to the diacylglycerol O-acyltransferase 1 gene *(Dgat1)*.

Next we investigated whether local gene density was related to the incidence of antisense transcription. To avoid bias due to the fact that *cis–*antisense pairs will always have an average density higher than that of individual random genes, we examined 100-kbp regions directly flanking each pair, rather than the region covered by the pair itself. Regions flanking *cis–*antisense pairs had roughly 30% higher TU density and 30% more exon sequence than regions flanking TUs not involved in *cis–*antisense pairs (Table S5). Many TUs in our dataset formed *cis–*antisense pairs and/or bidirectionally promoted pairs with several other TUs. To quantify this phenomenon, we searched for chains of bidirectional transcription, where we defined a chain as a group of three or more TUs associated by *cis*–antisense and/or bidirectionally promoted arrangement. Since TUs represent clusters of transcript sequences that in cases of incomplete coverage might not correspond to entire genes, and in rare instances contain sequence from adjacent genes [1], we applied strict rules on TU structure in order not to overestimate the occurrence and extent of chains (see Materials and Methods). In human we detected 1,480 chains, containing 5,263 TUs (12 % of all TUs). In mouse, there were 1,153 chains, containing 3,987 TUs (11% of all TUs) (Table 3). The largest computationally predicted chain involved 11 TUs: the human gene encoding the giant muscle protein titin, nine antisense TUs overlapping titin exons, and one TU that might represent an alternative 3′-end of titin transcripts. The titin chain aside, the largest human chains involved eight TUs, and the largest mouse chains involved seven TUs. An example of a five-TU chain from mouse is given in Figure 8. This region contains a gene encoding a well studied transcriptional regulator *(Hsf1)* and three metabolic genes, allowing the possibility of *cis-*regulation of genomically adjacent genes of diverse functions and resulting effects on their downstream targets. The genomic distribution of chains, *cis–*antisense pairs, and bidirectionally promoted pairs is illustrated in Figure S7. Human Chromosome 19, which has the highest gene density of all human chromosomes [30], also had the highest densities of *cis–*antisense pairs, bidirectionally promoted pairs, and chains.

**Table 3:**
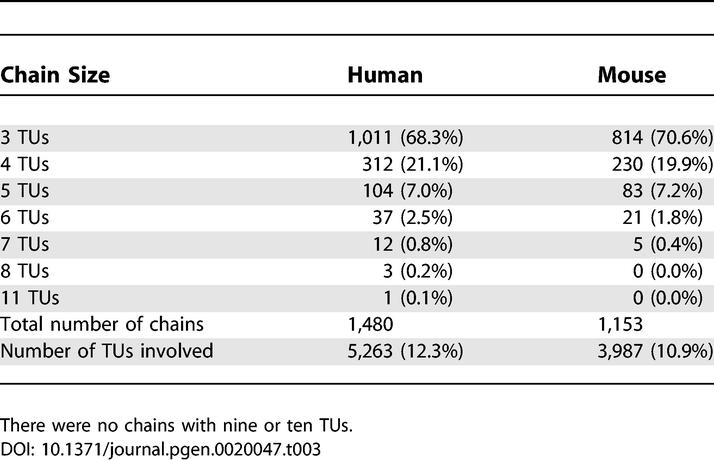
Numbers of Chains Detected and Their Sizes

### Nonconserved and Noncoding TUs Are Transcribed Antiparallel to Many Homeotic Genes

**Figure 9:**
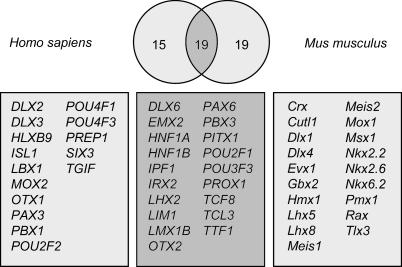
Dispersed Human and Mouse Homeotic Loci at Which ESTs Were Detected on the Opposite Strand from the Homeotic Gene Loci with opposite-strand ESTs in both genomes are listed in the center box.

Antisense transcripts to the *HOXA11* gene have been found to be conserved between human and mouse [31], and we have recently reported chains at the *HOXA* cluster in both organisms [13]. In mouse, the *Hoxa3* and *Hoxa7* genes formed a chain together with two uncharacterized TUs. In human, there were three chains of three TUs each, including *HOXA3, HOXA4, HOXA9, HOXA10, HOXA11,* and four uncharacterized TUs. We have further shown that several noncoding transcripts in the human *HOXA* cluster are coexpressed with adjacent coding *HOXA* genes in various human tissues, and are likely to be involved in the opening and closing of chromatin and sequential transcriptional activation of *HOX* cluster members (L. Sessa, A. Breiling, G. Lavorgna, L. Silvestri, V. Orlando, et al., unpublished data). Based on these findings, we selected homeotic genes as a group to focus on. *HOX* genes are arranged into four clusters in both human and mouse genomes [32]. Within each of the four clusters, the *HOX* genes are transcribed in the same direction. To assess the extent of transcription from the opposite strand at *HOX* loci and around dispersed homeotic genes (structurally and functionally related to the *HOX* genes), we specifically searched for EST sequences that mapped to the opposite strand at such loci and either overlapped homeotic genes or were intergenically located. We detected 232 human and 46 mouse ESTs on the opposite strand at *HOX* loci (Table 4), and a total of 445 opposite-strand ESTs distributed over 53 out of 95 dispersed human and mouse homeotic loci analyzed (Figure 9). The detected ESTs did not display any significant open reading frames or similarities to known proteins. Thus, transcription from the noncoding strand appears to be a general feature of homeotic loci.

**Table 4:**
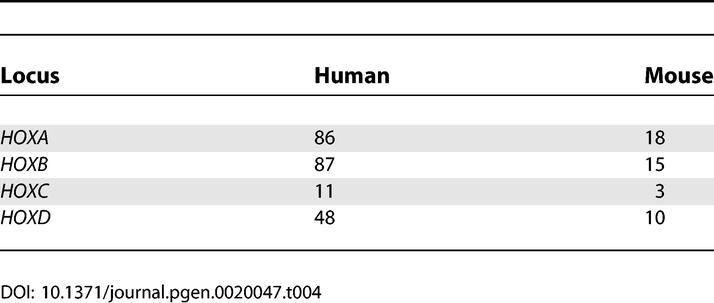
Numer of ESTs Detected on the Opposite Strand of Human and Mouse *HOX* Loci

ESTs at corresponding locations in human and mouse *HOX* clusters completely lacked similarities in exon–intron structures, indicating poor evolutionary conservation of these transcripts. To assess conservation at the sequence level, we attempted to match human and mouse homologs over the entire EST set from both *HOX* clusters and dispersed homeotic loci. For only one of all *HOX* genes (*HOXA11* [31]) was a significant alignment obtained between human and mouse ESTs. Similarly, out of 19 dispersed homeotic loci with ESTs on the opposite strand in both human and mouse, only four *(OTX2, DLX6, TCF8,* and *PITX1)* displayed ESTs with a high degree of sequence similarity. These matches did not span entire transcripts, but in each case were limited to a portion of a single exon. Given that *HOX* gene clusters and other homeotic gene loci are known to be spanned by arrays of highly conserved putative regulatory elements [33,34], these matches might be unrelated to the transcription from the noncoding strand that we had observed. In support of this, careful inspection of conservation at homeotic loci indicated no correlation between locations of detected ESTs and conserved segments.

Additionally, we manually inspected conservation in human of 234 mouse chains containing genes for other transcriptional regulatory proteins. Consistent with the homeotic genes case study, the results suggest lack of association between sequence conservation of chained transcriptional regulators and structure conservation of their chains (see Table S6).

## Discussion

As part of our effort to characterize complex loci, we report to our knowledge the most comprehensive list to date: 6,141 *cis–*antisense pairs in the human genome and 5,248 in the mouse. While methodological differences in redundancy reduction and clustering preclude direct comparison to earlier estimates also based on cDNA and EST data [10,12,21], the observed 2-fold difference in *cis–*antisense pair counts compared to these previous reports and widespread chaining of bidirectional transcription indicate that the earlier studies underestimated the prevalence and complexity of antisense transcription in human.

Taking advantage of the newly available CAGE tag data on TSSs [1], we have provided novel insight into the functional and sequence organization of a large set of bidirectional promoters that we showed to have a clearly identifiable midpoint at which overall sequence and motif composition changed strand and direction of transcriptional initiation switched. There is little or no spacing between the two segments used as transcription start regions in opposite directions, raising intriguing questions about the organization of events and specific transcription factor binding in a region that is used for transcriptional initiation along its entire length.

There is a paucity of previous studies on genes organized into structures that we refer to as chains. Veeramachaneni et al. [20] presented an account of 18 triplets of genes with overlapping exons in human and eight triplets in mouse. In this study we report a comprehensive catalog of more than 2,600 human and mouse chains of bidirectional and/or *cis–*antisense transcription with up to 11 TUs per chain. We identified 13 chains whose structures were entirely conserved between human and mouse, and that contained genes encoding transcriptional regulators (Table S6): these chains represent attractive candidates for testing the hypothesis that the putative *cis-*regulatory relationships suggested by chain structures (antisense regulation and coexpression from bidirectional promoters) have *trans-*regulatory impact when chains contain transcription factor genes. The structure of the complex loci also indicates that they should be taken into account in the process of designing microarray probes, which for the purpose of assessing expression levels of individual TUs in these loci should both be strand-specific and avoid targeting sequences shared by multiple TUs.

There is a striking agreement between the human and mouse datasets regarding proportions of TUs involved in *cis–*antisense and bidirectionally promoted pairs, as well as the structural properties of *cis–*antisense overlaps. The amount of sequence evidence that supports the existence of these structures is sufficient to reject the explanation that these transcripts are due to methodological “noise” [1]. Our finding that a limited but significant proportion (16%–18%) of *cis–*antisense pairs are conserved between human and mouse is in agreement with earlier data [19–21,24]. However, since these earlier studies were either smaller-scale or limited to protein-coding genes, the nearly 1,000 conserved *cis–*antisense pairs we report here are close to seven [19] or three times [21] more than previously found. We have also provided a quantitative estimation of the total number of genes in *cis–*antisense pairs, which is about 40% both in human and in mouse, regardless of the sampling method, sequence dataset redundancy, and different average quality of EST and full-length cDNA sequence data between the two species. Even though there exists a possibility that an even higher number of unsampled noncoding transcripts are present, the clear saturation of the sampling plot, the number of ESTs, and the diversity of sampled libraries make it unlikely that our method seriously underestimates the total count. Our estimate is in agreement with a recent report where hundreds of novel transcripts were characterized by RACE, and where it was found that 44% of all investigated transcripts overlapped a transcript on the opposite strand [4].

*HOX* genes are master regulators of vertebrate development and differentiation. Even though the loci of the four *HOX* gene clusters contain abundant evidence of transcription from the opposite strand in both human and mouse, we were unable to detect significantly evolutionary conserved antisense ESTs. This is unlikely to be entirely due to incomplete EST coverage of the regions, given the depth of recent EST sequencing efforts [23,35] and the observed cross-species differences in exon–intron structures on antisense strands. Alternatively, we suggest that antisense-strand transcription per se has been maintained throughout the evolution of different loci, regardless of the sequence being transcribed. The latter scenario is in agreement with the lack of long conserved open reading frames in antisense-strand ESTs (unpublished data), which is another property they share with positional equivalents in other complex loci. In a parallel work, we showed that transcripts from the antisense strand of the human *HOXA* locus are induced upon retinoic acid treatment, following the timely colinearity of the *HOX* sense transcripts (L. Sessa, A. Breiling, G. Lavorgna, L. Silvestri, V. Orlando, et al., unpublished data). The results suggest that antisense-strand transcription is involved in the opening and activation of mammalian *HOX* clusters and prevents, as an anti-silencing mechanism, the re-repression of the cluster. Therefore, transcription at a fixed location but without a fixed sequence can be functionally relevant to regulation of ancient conserved gene clusters fundamental to vertebrate development, implying that nonhomeotic positional equivalents, similarly, may impact the expression of their paired conserved adjacent genes.

Even though we managed to reproduce the previous findings [9,15] that genes in *cis–*antisense and bidirectionally promoted arrangement show a higher probability of being coordinately expressed than random pairs of genes, our overall understanding of their coregulation remains lacking. Current publicly available microarray datasets are of limited utility for studying the expression of overlapping genes, because of uncertainties about whether probes outside of *cis–*antisense overlaps sufficiently well represent transcript isoforms comprising the *cis–*antisense pairs, the possibility of mixed-signal detection unless expression measurements are strand-specific, and the limited representation of noncoding genes on commercially available microarrays.

In conclusion, we have shown that complex loci are widespread and include numerous lineage-specific transcripts and nonconserved gene structures. They are likely involved in regulatory events affecting large numbers of transcription factor genes, and are also associated with locus-specific synergistic expression profiles of paired genes. While many questions about the complex loci remain open, our complex loci catalog establishes a foundation for querying the regulatory significance of complex loci components by strand-specific microarray-based expression analyses [36,37], targeted disruptions in transgenic animals, and genome-wide perturbations using siRNA and overexpression constructs [13].

## Materials and Methods

### TU inference procedure.

Mappings of all public human cDNA and EST sequences to human genome assembly hg17 were obtained from the UCSC Genome Browser Database [25] in October 2004. Mappings of FANTOM3 and public cDNA and EST sequences to mouse genome assembly mm5 were produced in the FANTOM3 collaboration [1]. Mappings were post-processed by an algorithm designed to extend spliced alignments by using information about exon positions from neighboring mappings (P. Engstrom and B. Lenhard, unpublished data). Each mapping was assigned a score based on percent identity, transcript sequence coverage, and intron count. For each transcript sequence, we retained only its best-scoring mapping or none if it had several best-scoring mappings to the assembled chromosome sequences. Mappings with fewer than 150 nt and more than 75% of the transcript sequence mapped were discarded, as were mappings with a percent identity below 98.0% for cDNAs or 97.0% for ESTs. Mappings to any of the seven immunoglobulin and T-cell receptor loci were also discarded, because of the difficulty of obtaining accurate gene structures from transcripts with rearranged sequences. All remaining mappings were passed into a pipeline designed to filter out artifacts, reliably assign mappings to the correct genomic strand, and cluster them into TUs. The steps of this pipeline were as follows. (1) Each mapping was represented as a set of genomic exons, corresponding to mapped segments. Unmapped stretches of less than four bases (presumed cloning/sequencing errors or polymorphisms) were allowed within exons. A gap between exons was regarded as an intron if it spanned more than 19 bases and its initial/terminal dinucelotides (splice signals) were GT/AG, GC/AG, or AT/AC. (2) To trim mapped vector sequence or poly-A tails from ends of mappings, we removed external exons that were either (a) shorter than 11 bases or (b) shorter than 31 bases and consisted of 80% or more adenines or 80% or more thymines. (3) Mappings of sequences annotated with the same cDNA clone ID were merged if they mapped less than 100 kb apart and did not indicate conflicting gene structures. (4) Since our aim was to detect cases of bidirectional transcription, and the transcript sequence artifact most likely to result in false-positive cases of bidirectional transcription is sequence reversal, we designed a procedure to determine transcript sequence orientation with very high accuracy. Each mapping was assigned to a genomic strand (plus or minus) that should correspond to the sense strand of the gene identified by the mapping, or excluded if strand assignment was not possible. Mappings with two or more introns were oriented according their splice signals. Other mappings were oriented according to a combined assessment of splice signals, poly-A tails, polyadenylation signals, and annotated EST read direction. Further details are given in Figure S1. (5) To exclude mappings of transcript sequences resulting from priming at adenine stretches in genomic DNA or upstream of the poly-A tail in RNA transcripts, we discarded mappings if they lacked a polyadenylation signal (defined in Figure S1) and ended close to an adenine-rich region (ten or more adenines in a 14-base window in the genomic region [−11,+14] relative to a mapping's 3′-end). (6) Mappings were clustered into TUs by joining mappings that were on the same genomic strand and shared one or more bases of exon sequence. The gene structure of a TU was obtained by collapsing the exons of its participant mappings. (7) A TU made only from unspliced EST mappings was discarded if the mappings were fewer than a threshold *t*. The threshold *t* was set to the smallest integer greater than two for which *P*(Bin(*n*, *p*) ≥ *t*) < 0.01 (where *n* is the total number of EST-containing mappings in the assessed TU and all other-strand TUs that it has exon overlaps with, and *p* is 0.002, the estimated rate of misorientation of unspliced ESTs). The rationale behind the above threshold calculation was that, to infer a TU from unspliced ESTs only, we wanted the probability that those ESTs are misoriented (and therefore should belong to other-strand TUs) to be less than 0.01. The thresholding eliminated 128 potential TUs supported by more than three mappings.

### Accuracy assessment of orientation procedure.

We used spliced mappings that could be unambiguously oriented by their splice signals to estimate the accuracy of the part of the orientation procedure that handles unspliced mappings (Figure S1). All mappings with at least two introns and consistent splice signals were separately passed to the parts of the procedure that handle (a) spliced and (b) unspliced mappings. For each mapping, we regarded the result from (b) as correct if it agreed with the result from (a). Using the resulting accuracy rates (Figure S1), we simulated how many false TUs could be expected due to misorientation of unspliced mappings. The expected total numbers of misoriented unspliced cDNA and EST mappings were calculated, and the same numbers of cDNA and EST mappings randomly selected from the actual genome-wide set of unspliced mappings. The selected mappings were reversed and passed through steps 6 and 7 of the TU inference pipeline (see above), and the number of resulting TUs counted. In 100 simulations, we obtained on average 43 (standard deviation = 1.7) false human TUs and 48 (standard deviation = 1.0) false mouse TUs.

### Orientation-specific RT-PCR and qRT-PCR.

Adult male C57BL/6J mice were killed according to the RIKEN Institute's guidelines and the tissues were removed. Total RNA was extracted by the acid phenol-guanidinium thiocyanate-chloroform method [38]. RNA was checked by agarose gel electrophoresis and was treated with DNaseI before RT-PCR as described elsewhere [39]. Primer pairs were designed using Primer3 software [40], with an optimal primer size of 20 bases and annealing temperature of 60 °C (see Table S7). The uniqueness of the designed primer pairs was checked by a BLAST search [41] to avoid cross-amplification. The orientation-specific RT-PCR was performed as described elsewhere [12,24]. For qRT-PCR, first-strand cDNA synthesis (5 μg of total RNA per 20-μl reaction) was carried out using a random primer and the ThermoScript RT-PCR System (Invitrogen; http://www.invitrogen.com) in accordance with the manufacturer's protocol. qRT-PCR was carried out with first-strand cDNA corresponding to 12.5 ng of total RNA per test well using the tailor-made reaction [39]. The PCR reactions were performed with an ABI Prism machine (Applied Biosystems; http://www.appliedbiosystems.com) using the following cycling protocols: 15-min hot start at 94 °C, followed by 40 cycles of 15 s at 94 °C, 30 s at 60 °C, and 30 s at 72 °C. The threshold cycle (Ct) value was calculated from amplification plots, in which the fluorescence signal detected was plotted against the PCR cycle. The number of transcripts was calculated from the slope of the standard curve using genomic DNA or the corresponding cDNAs. Averages and standard deviations were calculated based on six qRT-PCR measurements for each primer pair.

### Automated identification of conserved of *cis–*antisense pairs.

For the comparative analyses, alignments between the human and mouse genomes (BLASTZ net and tight alignments) were retrieved from the UCSC Genome Browser Database [25]. Human-to-mouse net alignments are filtered to contain the best match in the mouse genome for every part of the human genome, and mouse-to-human net alignments are filtered to contain the best match in the human genome for every part of the mouse genome. We considered a human *cis–*antisense pair to be conserved in mouse if it had a region of exon overlap that aligned with a region of exon overlap from a mouse *cis–*antisense pair over at least 20 bp in both human-to-mouse and mouse-to-human BLASTZ net alignments. Saturation curves were fitted using nonlinear regression (nls function) in R (http://www.R-project.org).

### Identification of positional equivalents.

Candidate pairs for manual curation were selected by assessing the conservation of exon sequence outside regions of *cis–*antisense exon overlap. Regions where exons overlapped were excluded from consideration, since conservation in such regions cannot be specifically attributed to one of the pair members. We required that one pair member have exon sequence that overlapped with a BLASTZ tight alignment, and the other pair member have no exon sequence that overlapped with a BLASTZ net alignment. We further eliminated pairs where the “nonconserved” member gave a significant BL2SEQ alignment (*E* < 1,000; word size = 7; filter off) [42] with the genomic sequence from the other organism, and pairs where we failed to identify a human transcript sequence for the conserved member by manual curation.

### Analysis of TSS distribution and sequence composition of bidirectional promoters.

TCs were defined by associating CAGE tag mappings that overlapped on the genome (CAGE tag sequences are typically 20 bases long) (P. Carninci, A. Sandelin, B. Lenhard, D. A. Hume, Y. Hayashizaki, et al., unpublished data). To study the locations of TSSs at bidirectional promoters, we constructed a set of 766 bidirectional promoters defined by TCs by scanning the mouse genome for pairs of divergently oriented TCs spaced by less than 500 bp and having no intervening genome assembly gaps. Divergent TCs were allowed to overlap only partially, i.e., the most downstream TSS in each TC was required to be outside the overlap. We only considered TCs that contained at least ten tags and overlapped the 5′-end of a TU or cDNA sequence approved by the TU inference pipeline. To avoid redundancy, we paired a TC only with the nearest divergent TC satisfying these criteria. A control set of unidirectional promoters was constructed by scanning for single TCs fulfilling the same criteria for tag counts and TU/cDNA overlap and that had no assembly gaps or divergently oriented TCs with any number of tags in the 500 bp upstream.

Using locations of CpG islands obtained from the UCSC Genome Browser Database [25], a TC was considered to be CpG-island-associated if there was a CpG island in the 500 bp upstream of the most downstream TSS in the TC. One bidirectional and one unidirectional TC that each spanned more than 500 bases were not classified with respect to CpG islands. We searched for Sp1 binding sites using the Sp1 position weight matrix from the Jaspar database [43] and the TFBS Perl modules [44]. We applied a relative matrix score threshold of 80%.

### Expression analysis.

MAS5-processed expression data for 61 mouse tissues measured in duplicate on the GNF1M chip [28] were obtained from the Genomics Institute of the Novartis Research Foundation (http://wombat.gnf.org). *cis–*antisense pairs in mouse were matched with GNF1M probesets using probe-to-FANTOM cDNA clone mappings produced in the FANTOM3 collaboration [1]. For *cis–*antisense pairs we selected only probesets that mapped to regions of exon overlap between the *cis–*antisense partners. When multiple probesets were available for a TU, the probeset mapping to the most cDNA sequences was selected in order to preferentially measure major transcript isoforms. Spearman rank correlations and *p*-values were computed for each pair over all tissues, using the function cor.test in R. *p*-Values were adjusted for multiple testing using the Bonferroni method.

### Automated identification of chains.

Chains were identified by searching the entire TU dataset for clusters of three or more TUs connected by *cis–*antisense overlaps and/or putative bidirectional promoters. In order not to overestimate the occurrence and extent of chains, we did not allow chains to be held together by (1) gaps between EST end-reads, (2) other gaps that separated exons and were not classified as introns by the criteria described above, (3) introns with GC/AG or AT/AG splice signals and longer than 15 kb, and (4) introns with GT/AG splice signals and longer than 45 kb. Cases 2 to 4 were allowed if supported by more than one mapping. When counting the number of TUs in a chain, we ignored unspliced TUs that started within 1.5 kb of the end of a mapping that were on the same strand and part of the same chain, since such unspliced TUs might represent 3′-UTR extensions. We note that this rule might be overly conservative in light of the recent discovery of widespread transcription initiation within 3′-UTRs [1].

### Analysis of antisense transcription at homeotic loci.

The precise genomic organization of homeotic loci was determined by visualizing them in the UCSC Genome Browser [25] and by BLASTN searches of the nonredundant (nr) and unfinished high-throughput genomic sequences (htgs) databases [41] with cDNA and EST sequences representing homeotic genes. Following initial structure and conservation analysis, we queried entire *HOX* clusters and 10-kb regions centered on TSSs of dispersed homeotic genes for presence of antisense-strand transcripts using the AntiHunter software [45] with default parameters. An all-against-all comparison of antisense-strand EST sequences was performed using the Unix command line version of the program seqmatchall from the EMBOSS package [46]. Human and mouse sequences aligned over at least 30 nt were realigned with BL2SEQ [42] with default parameters. Alignments with an *E*-value below 0.001 were considered significant. Genomic comparisons between human and mouse loci were performed using PipMaker [47] with default parameters.

### Datasets.

The dataset of TUs, *cis–*antisense pairs, bidirectionally promoted pairs, and chains can be obtained at http://www.genereg.net/complex_loci. A detailed image is provided for each chain.

## Supporting Information

Figure S1 Flowchart Describing the Procedure Used to Assign a Mapping to a Genomic Strand (32 KB PDF)

Figure S2 Transcript Sequence Types Supporting Exon Overlaps in *cis–*Antisense Pairs (191 KB PDF)

Figure S3 Alternative Approaches to Estimate the Extent of Antisense Transcription (30 KB PDF)

Figure S4 Properties of TSS Distributions at Bidirectional Promoters (319 KB PDF)

Figure S5 Landmark Sequence Composition of Bidirectional Promoters (66 KB PDF)

Figure S6 Enrichment of Putative Sp1 Binding Sites at Bidirectional Promoters (14 KB PDF)

Figure S7 Chromosomal Distribution of *cis–*Antisense Pairs, Bidirectionally Promoted Pairs, and Chains (6.4 MB TIF)

Table S1 *cis–*Antisense Pairs Classified According to Splicing Status and Relative Orientation of Participant TUs (28 KB PDF)

Table S2 *cis–*Antisense Pairs Conserved between Human and Mouse by Automated Assessment of Conservation (590 KB PDF)

Table S3 Mouse–Human Positional Equivalents Detected among *cis–*Antisense and Bidirectionally Promoted Pairs by Manual Curation (25 KB PDF)

Table S4 *cis–*Antisense Pairs with Significantly Correlated Expression between Pair Members (25 KB PDF)

Table S5 Measures of Gene Density around *cis–*Antisense Pairs and TUs Not Involved in *cis–*Antisense Pairs (17 KB PDF)

Table S6 Conservation of Transcriptional-Regulator-Containing Chains by Manual Curation (18 KB PDF)

Table S7 Primer Pairs for RT-PCR (19 KB PDF)

### Accession Numbers

The NCBI EntrezGene (http://www.ncbi.nlm.nih.gov/entrez/query.fcgi?db=gene) accession numbers for the genes discussed in this paper are *Bop1* (12181), *Cope* (59042), *D330001F17Rik* (223658); *Ddx49* (234374), *Dgat1* (13350), *DLX6* (1750), *HOXA10* (human) (3206), *HOXA11* (human) (3207), *HOXA3* (human) (3200), *Hoxa3* (mouse) (15400), *HOXA4* (human) (3201), *Hoxa7* (mouse) (15404), *HOXA9* (human) (3205), *Hsf1* (15499), *OTX2* (5015), *PITX1* (5307), *Ppp1ca* (19045), *Rad9* (19367), *RNH1* (human) (6050), *Rnh1* (mouse) (107702), *Rps2* (16898), *Rps27* (57294), *TCF8* (6935), and *titin* (7273).

## Abbreviations

CAGE: cap analysis of gene expression
EST: expressed sequence tag
qRT-PCR: quantitative real-time PCR
TC: CAGE tag cluster
TSS: transcriptional start site
TU: transcriptional unit
UCSC: University of California Santa Cruz
UTR: untranslated region
