# Supplementary material for: Complex Loci in Human and Mouse Genomes
Source: PLoS Genet. 2006 Apr 28;2(4):e47. doi: 10.1371/journal.pgen.0020047 (PMC1449890; doi:10.1371/journal.pgen.0020047)
Supplement: Figure S1 — (32 KB PDF) [file pgen.0020047.sg001.pdf]

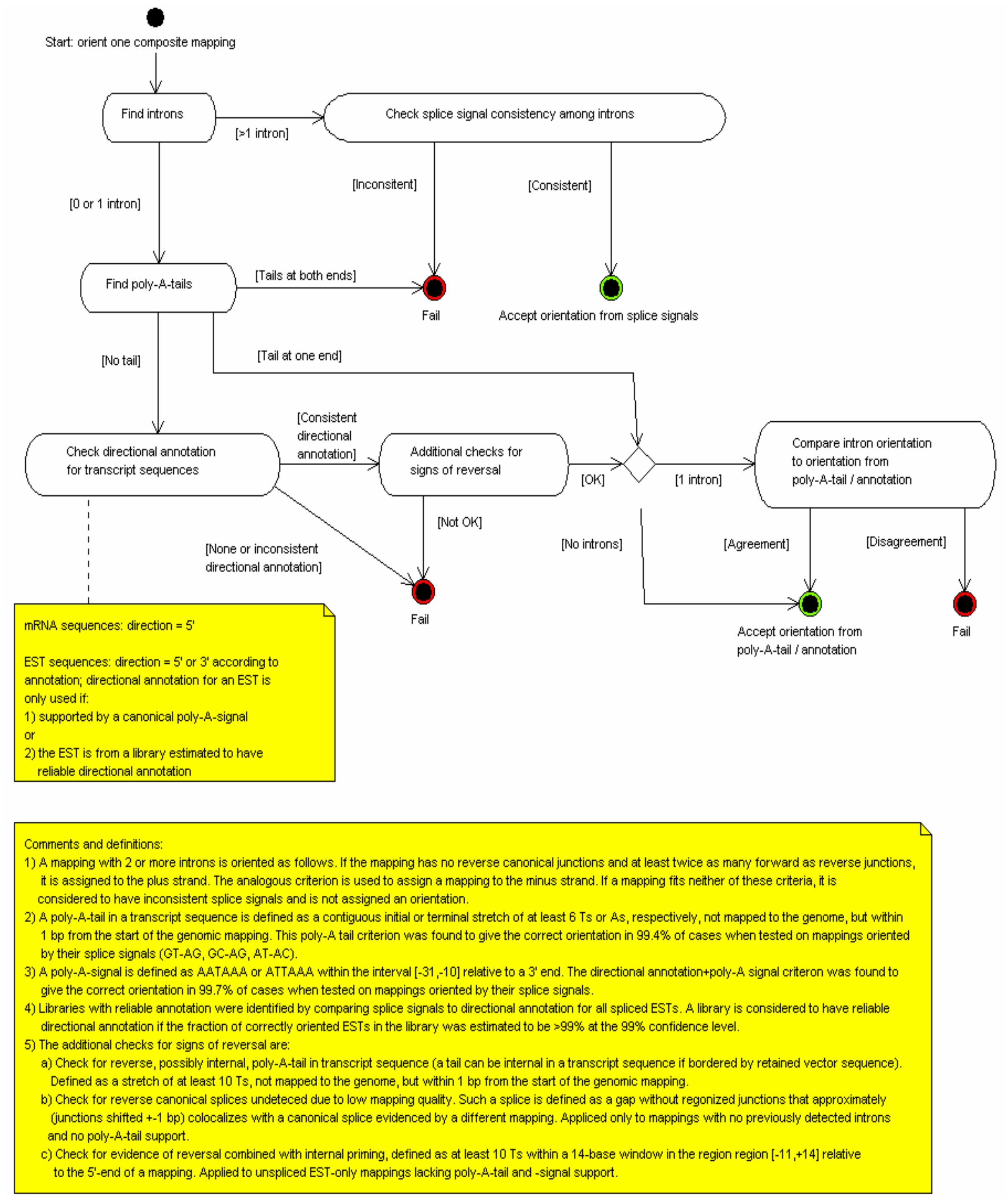

Figure S1 Flowchart describing the procedure used to assign a mapping to a genomic strand. Accuracy rates for orientation of unspliced mappings were estimated as described in Methods. Ninety-five percent confidence intervals, calculated using the binomial distribution, for the estimates were (99.80%, 99.85%) for human cDNA mappings, (99.75%, 99.77%) for human EST mappings, (99.73%, 99.79%) for mouse cDNA mappings and (99.81%, 99.83%) for mouse EST mappings.
