# Supplementary material for: Complex Loci in Human and Mouse Genomes
Source: PLoS Genet. 2006 Apr 28;2(4):e47. doi: 10.1371/journal.pgen.0020047 (PMC1449890; doi:10.1371/journal.pgen.0020047)
Supplement: Figure S2 — (191 KB PDF) [file pgen.0020047.sg002.pdf]

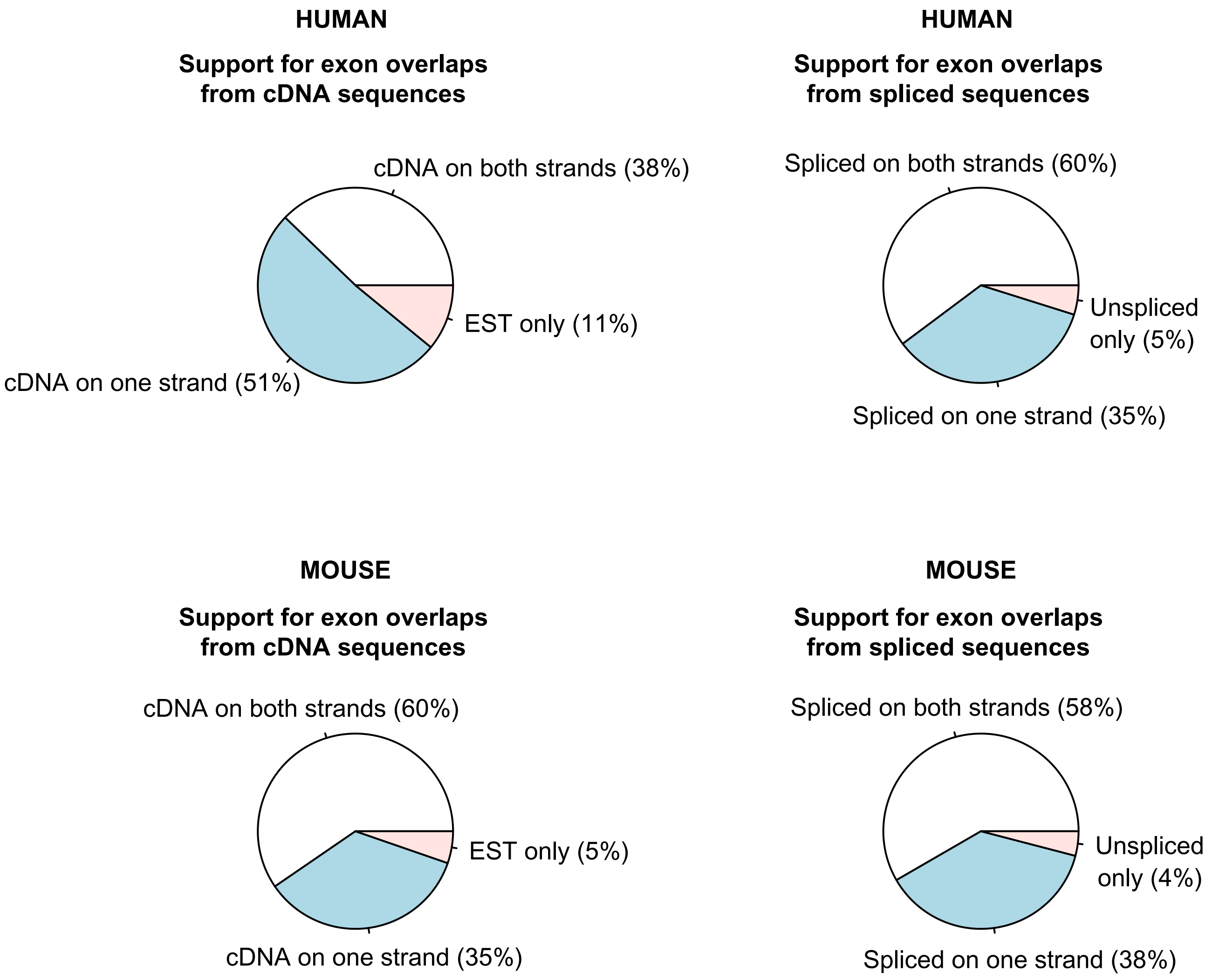

Figure S2 Transcript sequence types supporting exon overlaps in *cis*-antisense pairs. Shown are the proportions of *cis*-antisense pairs that have exon overlaps supported by the different sequence types.
