# Supplementary material for: Complex Loci in Human and Mouse Genomes
Source: PLoS Genet. 2006 Apr 28;2(4):e47. doi: 10.1371/journal.pgen.0020047 (PMC1449890; doi:10.1371/journal.pgen.0020047)
Supplement: Figure S3 — (30 KB PDF) [file pgen.0020047.sg003.pdf]

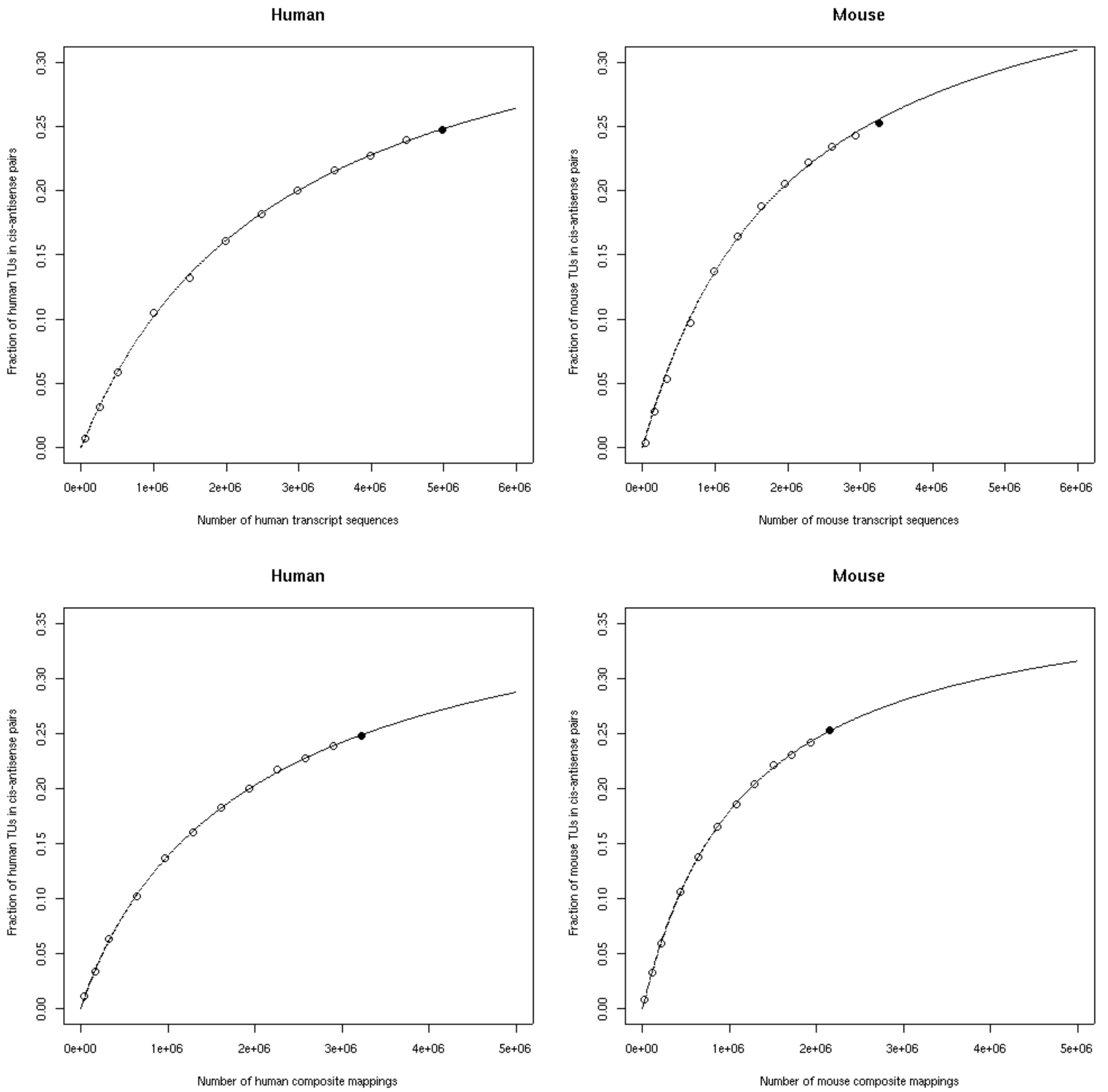

Figure S3 Alternative approaches to estimate the extent of antisense transcription. In addition to the approach described in Figure 3, we estimated the proportion of TUs involved in *cis*-antisense pairs in two other ways, both of which gave very similar estimates to those described in Figure 3. In all cases, a saturation curve ($y = \dfrac{ax^c}{b + x^c}$) indicated by the line fit very well to the sampled data.

(A) Open circles indicate the fraction of human (left panel) or mouse (right panel) TUs that were found to be involved in *cis*-antisense pairs when the the entire TU datasets was recomputed starting from random transcript sequence samples of different sizes. Filled circles represent the full datasets based on all available transcript sequences. Fitted human and mouse saturation curves approach 0.39 and 0.42, respectively, as the number of transcript sequences increases. Curves with c=1 were preferable as the more complex model did not provide a significantly better fit.

(B) The sampling approach was the same as in panel A, except that composite transcript-to-genome mappings (one mapping per cDNA clone) that had passed all quality criteria applied in the TU inference procedure (steps 1-5 of the TU inference procedure; see Methods) were sampled instead of transcript sequences. With this appriach, fitted human and mouse saturation curves approach 0.42 and 0.39, respectively, as the number of transcript sequences increases.
