# Supplementary material for: Complex Loci in Human and Mouse Genomes
Source: PLoS Genet. 2006 Apr 28;2(4):e47. doi: 10.1371/journal.pgen.0020047 (PMC1449890; doi:10.1371/journal.pgen.0020047)
Supplement: Figure S4 — (319 KB PDF) [file pgen.0020047.sg004.pdf]

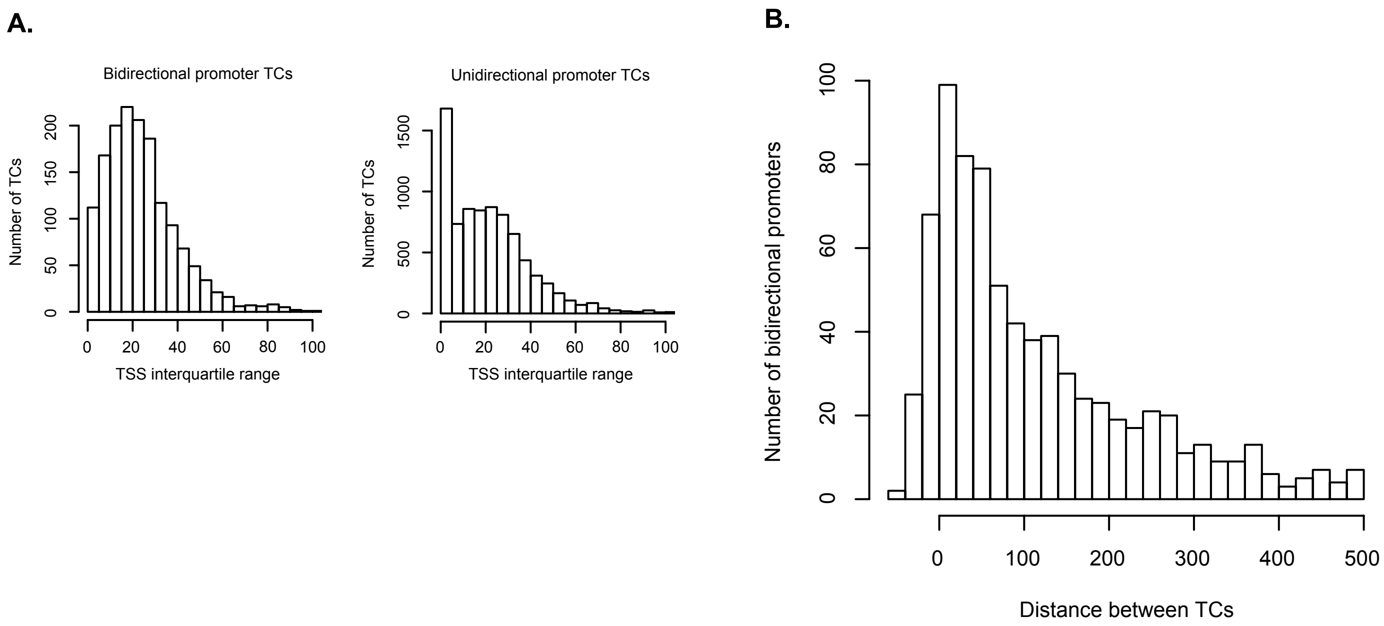

Figure S4 Properties of TSS distributions at bidirectional promoters

(A) Bidirectional promoters are associated with broad TSS regions. The histograms show interquartile range distributions (in bases) of TSS locations for CAGE tag clusters (TCs) at bidirectional and unidirectional promoters. Bidirectional and unidirectional promoters were identified as described in Methods.
The TSS interquartile range measures the dispersion of TSSs in a TC. The large differences in dispersion between unidirectional and bidirectional promoter TCs can not be explained by higher CAGE tag counts for the bidirectional promoter TCs, because CAGE tag counts were not significantly different between the two TC sets, and the same differences in dispersion are seen when the comparison is limited to TCs containing at least 100 tags each (data not shown).

(B) Rarity of overlap between divergent TSSs at bidirectional promoters. Distances (bp) between divergently oriented TCs at bidirectional promoters. Negative distances indicate that the TCs overlap. The divergent TCs are often closely spaced, but rarely overlap.
