# Supplementary material for: Complex Loci in Human and Mouse Genomes
Source: PLoS Genet. 2006 Apr 28;2(4):e47. doi: 10.1371/journal.pgen.0020047 (PMC1449890; doi:10.1371/journal.pgen.0020047)
Supplement: Figure S5 — (66 KB PDF) [file pgen.0020047.sg005.pdf]

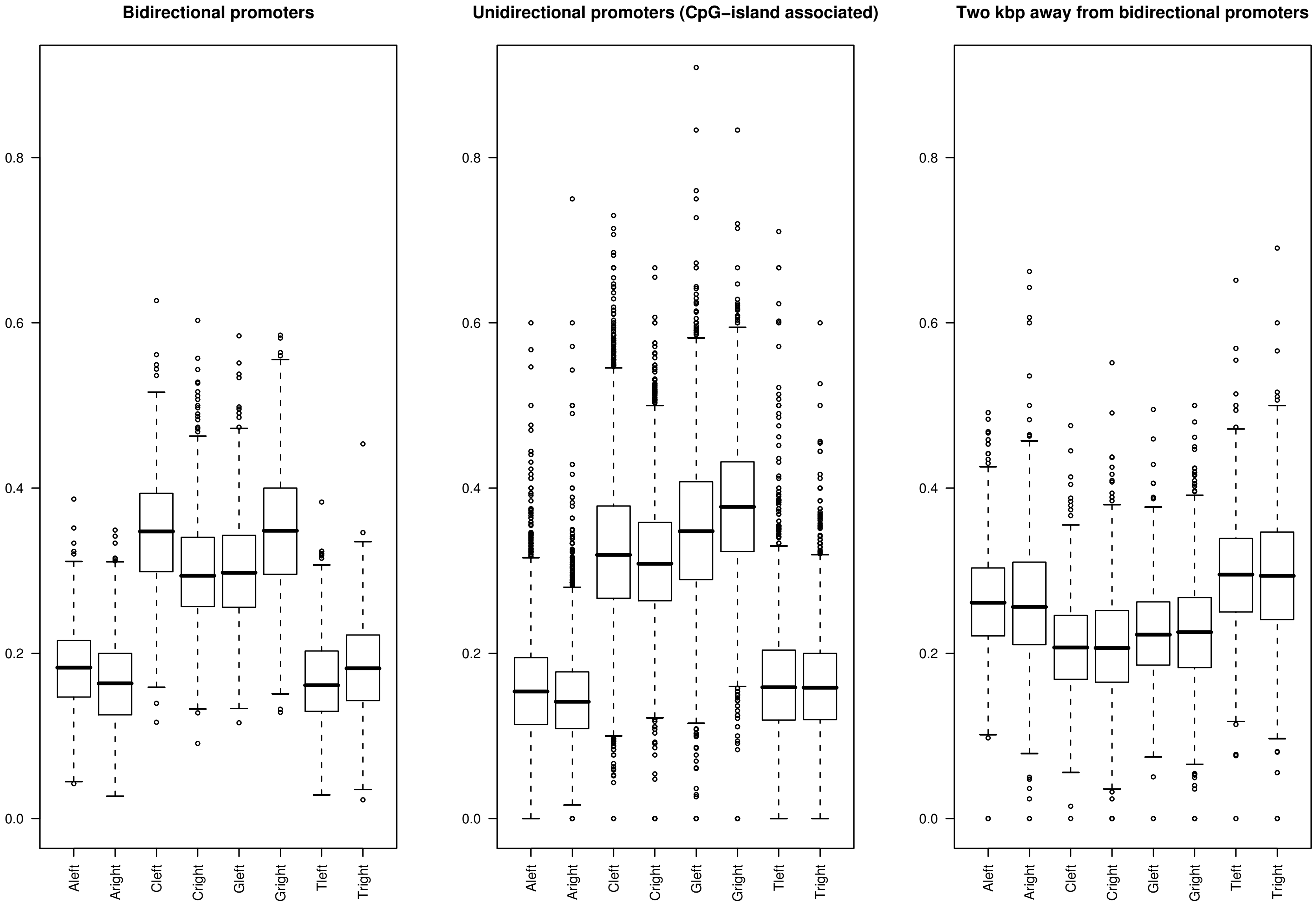

Bidirectional promoters
Unidirectional promoters (CpG−island associated)
Two kbp away from bidirectional promoters
0.0
0.2
0.4
0.6
0.8
Aleft
Aright
Cleft
Cright
Gleft
Gright
Tleft
Tright

Figure S5 Landmark sequence composition of bidirectional promoters

To quantify the differences in sequence composition across the midpoints of bidirectional promoters, we determined the proportion of each nucleotide (A,G,C,T) on the left and right side of bidirectional promoter midpoints. For each bidirectional promoter, we counted nucleotides in the region between the extreme ends of the two divergent CAGE tag clusters identifying the bidirectional promoter.

In the left panel, the boxplots show the distribution of nucleotide frequencies for all 766 bidirectional promoters identified. The distributions indicate a mirror sequence composition across the midpoints of bidirectional promoters. In the middle panel, the boxplots show the corresponding distributions for all 4,282 CpG-island overlapping unidirectional promoters (all promoters in our control set of unidirectional promoters where the TC was completely within a CpG island). Here, the right side corresponds to the CAGE tag cluster identifying the unidirectional promoter, and the left side to an equally sized region immediately upstream. The right panel shows the result for 766 regions, each 2 kb away from a bidirectional promoters and of the same size as that bidirectional promoter.
