# Supplementary material for: Complex Loci in Human and Mouse Genomes
Source: PLoS Genet. 2006 Apr 28;2(4):e47. doi: 10.1371/journal.pgen.0020047 (PMC1449890; doi:10.1371/journal.pgen.0020047)
Supplement: Figure S6 — (14 KB PDF) [file pgen.0020047.sg006.pdf]

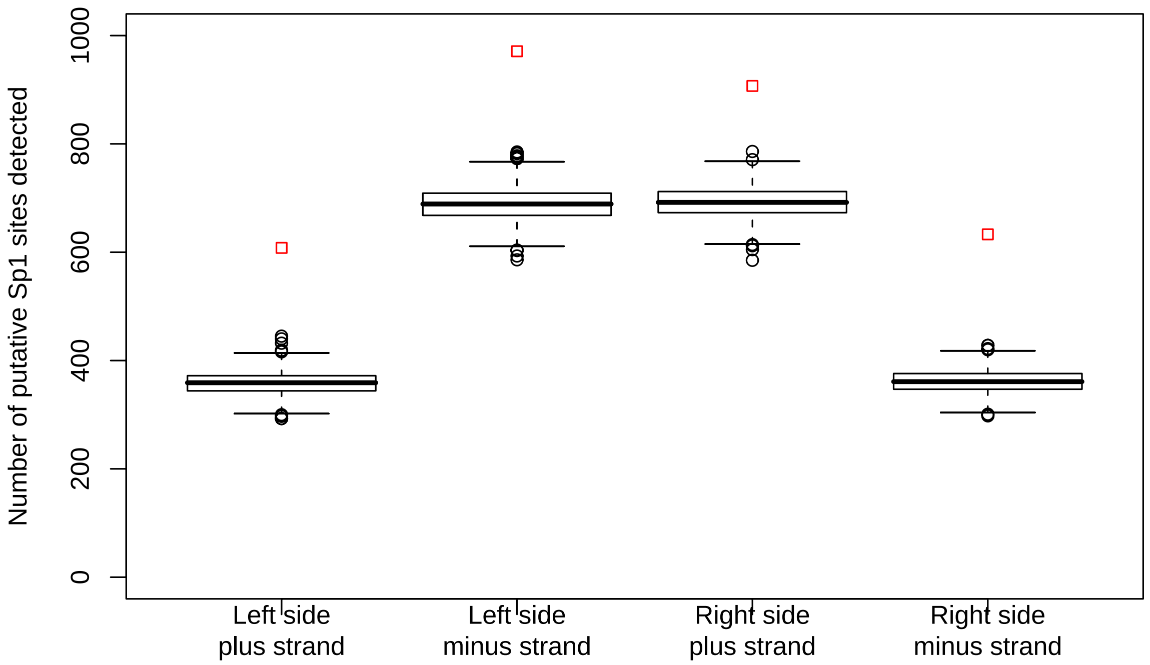

Figure S6 Enrichment of putative Sp1 binding sites at bidirectional promoters

Red squares indicate the total number of putative Sp1 sites identified at all 766 bidirectional promoters. Sp1 sites were detected by scanning the sequences with a position weight matrix as described in Materials and Methods. We counted putative Sp1 sites separately on the left and right sides of the midpoint of each bidirectional promoter, and separately on plus and minus strands. The box plots show the results from applying the same search to 1,000 sets of 766 random sequences with the same lengths and dinucleotide frequencies as the bidirectional promoters. The random sequences were generated from a first-order Markov chain in order to preserve the dinucleotide composition of each of the four regions. For none of the 1,000 random sets did we detect as many putative sites as for the bidirectional promoters in any of the four regions considered.
