# Supplementary material for: Complex Loci in Human and Mouse Genomes
Source: PLoS Genet. 2006 Apr 28;2(4):e47. doi: 10.1371/journal.pgen.0020047 (PMC1449890; doi:10.1371/journal.pgen.0020047)
Supplement: Table S1 — (28 KB PDF) [file pgen.0020047.st001.pdf]

Table S1 *Cis*-antisense pairs classified according to splicing status and relative orientation of participant TUs.

A. Human *cis*-antisense pairs, all

	Both unspliced	1 spliced + 1 unspliced	Both spliced	Total
Convergent	33 (22%)	271 (15%)	1554 (38%)	1858 (30%)
Divergent	40 (27%)	364 (19%)	1287 (31%)	1691 (28%)
Full overlap	75 (51%)	1233 (66%)	1284 (31%)	2592 (42%)
Total	148	1868	4125	6141

B. Mouse *cis*-antisense pairs, all

	Both unspliced	1 spliced + 1 unspliced	Both spliced	Total
Convergent	22 (18%)	220 (13%)	1320 (38%)	1562 (30%)
Divergent	71 (57%)	650 (38%)	1159 (34%)	1880 (36%)
Full overlap	32 (26%)	823 (49%)	951 (28%)	1806 (34%)
Total	125	1693	3430	5248

C. Human *cis*-antisense pairs with exon overlaps supported by cDNA sequences on both strands

	Both unspliced	1 spliced + 1 unspliced	Both spliced	Total
Convergent	5 (11%)	91 (12%)	753 (49%)	849 (37%)
Divergent	12 (26%)	173 (23%)	389 (25%)	574 (25%)
Full overlap	30 (64%)	483 (65%)	385 (25%)	898 (39%)
Total	47	747	1527	2321

D. Mouse *cis*-antisense pairs with exon overlaps supported by cDNA sequences on both strands

	Both unspliced	1 spliced + 1 unspliced	Both spliced	Total
Convergent	16 (21%)	135 (12%)	913 (47%)	1064 (34%)
Divergent	47 (61%)	439 (40%)	563 (29%)	1049 (34%)
Full overlap	14 (18%)	516 (47%)	483 (25%)	1013 (32%)
Total	77	1090	1959	3126
