# Supplementary material for: Complex Loci in Human and Mouse Genomes
Source: PLoS Genet. 2006 Apr 28;2(4):e47. doi: 10.1371/journal.pgen.0020047 (PMC1449890; doi:10.1371/journal.pgen.0020047)
Supplement: Table S2 — (590 KB PDF) [file pgen.0020047.st002.pdf]

Table S2 Cis-antisense pairs conserved between human and mouse from automated assessment of conservation.
Each row represents one TU in the human or mouse genome. Each group of four rows represents one human and one mouse cis-antisense pair found to correspond by automated assessment of pair conservation (see Methods).

Org.: organism
TU location: location of transcriptional unit (chromosome, start, end, strand) in human genome assembly hg17 or mouse genome assembly mm5
Overlap seq.: Accession number or RIKEN clone ID for a representative cDNA or EST sequence supporting the cis-antisense exon overlap
TU cDNA: Accession number or RIKEN clone ID for representative cDNA sequence for the TU if available (this sequence may map outside the region of exon overlap)
Description: Description obtained from the sequence record of the cDNA representative for the TU

Org.	TU location	Overlap seq.	TU cDNA	Description
Human	chr1:900349-920104:+	BC033213	BC024295	Homo sapiens hypothetical protein MGC45873, mRNA (cDNA clone MGC:39333 IMAGE:3354502), complete cds.
Human	chr1:919726-986491:-	AK092491	BX648399	Homo sapiens mRNA; cDNA DKFZp686M11224 (from clone DKFZp686M11224).
Mouse	chr4:154132299-154140990:-	A930008A01	A930008A01	similar to early development regulator 2 (Homolog of polyhomeotic 2), polyhomeotic 2 protein, DNA segment, Chr 4, ERATO Doi 810, expressed [Homo sapiens]
Mouse	chr4:154121343-154132949:+	B130021F16	B130021F16	UPF0120 protein DKFZp564C186 homolog [Mus musculus]
Human	chr1:1283870-1286982:+	AK027721	AK075292	Homo sapiens cDNA FLJ90811 fis, clone Y79AA1000888, weakly similar to TRNA PSEUDOURIDINE SYNTHASE A (EC 4.2.1.70).
Human	chr1:1286888-1299989:-	AL136813	AL136813	Homo sapiens mRNA; cDNA DKFZp434A1923 (from clone DKFZp434A1923); complete cds.
Mouse	chr4:153773821-153778076:-	2810021I11	I830030E08	hypothetical tRNA pseudouridine synthase containing protein
Mouse	chr4:153755482-153775045:+	G630016P22	G630016P22	OVARC1001232 PROTEIN homolog [Homo sapiens]
Human	chr1:1308411-1310619:+	AK122959	AK122959	Homo sapiens cDNA FLJ16679 fis, clone TLIVE2003381, moderately similar to Mus musculus taste receptor, type 1, member 3 (Tas1r3).
Human	chr1:1310579-1324683:-	BC050454	AK095867	Homo sapiens cDNA FLJ38548 fis, clone HCHON2001768, highly similar to SEGMENT POLARITY PROTEIN DISHEVELLED HOMOLOG DVL-1.
Mouse	chr4:153745214-153749304:-	AF337039	AB049994	Mus musculus mRNA for taste receptor T1R3, complete cds. CDS=33..2609
Mouse	chr4:153733344-153745395:+	U10115	F630221L20	dishevelled, dsh homolog 1 (Drosophila)
Human	chr1:1455523-1463529:+	AK096773	AK075366	Homo sapiens cDNA PSEC0053 fis, clone NT2RP2000396, weakly similar to COLLAGEN ALPHA 1(XII) CHAIN PRECURSOR.
Human	chr1:1459753-1461411:-	AI142091	-	-
Mouse	chr4:153654437-153660640:-	AY030094	4932416A11	VON WILLEBRAND FACTOR A-RELATED PROTEIN homolog [Mus musculus]
Mouse	chr4:153655911-153662103:+	9530003H10	9530003H10	inferred: Mus musculus von Willebrand factor A-related protein (Warp) mRNA, complete cds
Human	chr1:2355374-2369045:+	CA310671	AF157324	Homo sapiens RER1 protein (RER1) mRNA, complete cds.
Human	chr1:2367382-2377398:-	BC000543	AB013818	Homo sapiens PEX10 mRNA for peroxisome biogenesis factor (peroxin) 10, complete cds.
Mouse	chr4:152960051-152972324:-	1110060F11	1110060F11	RER1 PROTEIN homolog [Homo sapiens]
Mouse	chr4:152958751-152960096:+	K530022N05	K530022N05	unclassifiable
Human	chr1:6199801-6215625:+	AK127700	AK056658	Homo sapiens cDNA FLJ32096 fis, clone OCBBF2001075.
Human	chr1:6215518-6230328:-	AL117548	AF064084	Homo sapiens prenylcysteine carboxyl methyltransferase (PCCMT) mRNA, complete cds.
Mouse	chr4:150192439-150204935:-	A630086J06	A630086J06	hypothetical Zn-finger, B-box/Zn-finger, RING containing protein
Mouse	chr4:149785075-150193456:+	BC006724	BC006724	Mus musculus isoprenylcysteine carboxyl methyltransferase, mRNA (cDNA clone MGC:6999 IMAGE:3155246), complete cds. CDS=616..894
Human	chr1:9842642-9844478:+	BG189868	-	-
Human	chr1:9842594-9904651:-	AB021262	AB021262	Homo sapiens mRNA for beta-catenin-interacting protein ICAT, complete cds.
Mouse	chr4:147451612-147456883:-	0610010J11	0610010J11	inferred: beta-catenin-interacting protein ICAT
Mouse	chr4:147404178-147452381:+	AB021261	9630044H20	BETA-CATENIN-INTERACTING PROTEIN ICAT (2310001I19RIK PROTEIN) (1110008O09RIK PROTEIN) homolog [Mus musculus]
Human	chr1:9937247-9980017:+	AI244413	AF459819	Homo sapiens nicotinamide mononucleotide adenylyl transferase mRNA, complete cds.
Human	chr1:9916437-9937726:-	BX491165	AB060688	Homo sapiens mRNA for Leucine zipper & ICAT homologous protein LZIC, complete cds.
Mouse	chr4:147353511-147372000:-	G830010G13	I830068L10	NMN adenylyltransferase; nicotinamide mononucleotide adenylyl transferase
Mouse	chr4:147371171-147389457:+	I920062A06	I920062A06	leucine zipper and CTNNBIP1 domain containing
Human	chr1:11006684-11024851:+	H25391	BC071657	Homo sapiens TAR DNA binding protein, mRNA (cDNA clone MGC:87845 IMAGE:5498250), complete cds.
Human	chr1:11020847-11041556:-	Y09926	Y09926	Homo sapiens mRNA for MASP-2 protein.
Mouse	chr4:146498324-146512961:-	1190002A23	4932416M19	TAR DNA binding protein
Mouse	chr4:146488486-146501441:+	C730007K24	BC013893	Mus musculus mannan-binding lectin serine protease 2, mRNA (cDNA clone MGC:13718 IMAGE:4211293), complete cds. CDS=17..2074
Human	chr1:15919665-16129127:+	AL096858	AF356524	Homo sapiens nuclear receptor transcription cofactor (SHARP) mRNA, complete cds.
Human	chr1:16012204-16047927:-	BU740481	Y09723	H.sapiens mRNA for Miz-1 protein.
Mouse	chr4:139924191-139928704:-	AI451144	-	-
Mouse	chr4:139900956-139924498:+	5930405B01	5930405B01	zinc finger protein 100
Human	chr1:25409296-25434830:+	BC066908	AL136627	Homo sapiens mRNA; cDNA DKFZp564C162 (from clone DKFZp564C162); complete cds.
Human	chr1:25432466-25501999:-	X63095	AK127547	Homo sapiens cDNA FLJ45640 fis, clone COLON2005735, highly similar to Blood group RH(CE) polypeptide.
Mouse	chr4:133352475-133369650:-	G430084L05	F830206N18	Small membrane protein 1
Mouse	chr4:133352653-133353103:+	M12449	M12449	Mouse cell adhesion molecule (CAM) uvomorulin mRNA.

Human	chr1:26180534-26181916:+	BC002580	BC002580	Homo sapiens zinc finger protein, mRNA (cDNA clone MGC:717 IMAGE:3143091), complete cds.
Human	chr1:26180149-26182454:-	BM717234	-	-
Mouse	chr4:132697915-132700218:-	5330410E07	5330410E07	Zinc finger protein T86
Mouse	chr4:132698288-132700499:+	E130106C14	E130218I03	unclassifiable
Human	chr1:28246748-28257552:+	BC009677	BC009677	Homo sapiens, ATPase inhibitor precursor, clone MGC:8898 IMAGE:3877506, mRNA, complete cds.
Human	chr1:28210026-28247075:-	BP220387	CR619944	full-length cDNA clone CS0DF007YK17 of Fetal brain of Homo sapiens (human).
Mouse	chr4:130754665-130759524:+	I830067M06	I830067M06	ATPase inhibitor
Mouse	chr4:130756258-130757309:-	K0C0008L04	-	-
Human	chr1:29034231-29267174:+	BC081539	M14993	Human structural protein 4.1 mRNA, complete cds.
Human	chr1:29266553-29271037:-	BC082989	BC082989	Homo sapiens cDNA DKFZp434C184 gene, mRNA (cDNA clone MGC:90489 IMAGE:5763085), complete cds.
Mouse	chr4:130172395-130324990:-	2310065B16	L00919	Mus musculus protein 4.1 mRNA, 5' end of ORF1, 3' end of ORF2, and 23 exons. CDS=2027..4129
Mouse	chr4:130169536-130172943:+	4022425N20	-	-
Human	chr1:31438926-31507811:+	AF151077	AK022506	Homo sapiens cDNA FLJ12444 fis, clone NT2RM1000187, weakly similar to PUTATIVE PRE-MRNA SPLICING FACTOR ATP-DEPENDENT RNA HELICASE SPAC10F6.02C.
Human	chr1:31475007-31522415:-	CN283506	BC007021	Homo sapiens, fatty acid binding protein 3, muscle and heart (mammary-derived growth inhibitor), clone MGC:12366 IMAGE:3934295, mRNA, complete cds.
Mouse	chr4:128685693-128730311:-	I920064O13	I920064O13	PS1D protein (Similar to hypothetical protein) (Putative S1 RNA binding domain protein) (LDC4)
Mouse	chr4:128678911-128685779:+	U02883	D330002O19	fatty acid binding protein 3, muscle and heart
Human	chr1:32335097-32340084:+	BC002462	BC002462	Homo sapiens hypothetical protein MGC1203, mRNA (cDNA clone MGC:1203 IMAGE:3344738), complete cds.
Human	chr1:32339008-32341501:-	CA425031	-	-
Mouse	chr4:127989588-127994227:-	2310005M03	F930017B17	weakly similar to Chemokine C-C motif receptor-like 1 adjacent [Homo sapiens]
Mouse	chr4:127970982-127989890:+	B230310F21	B230310F21	RIKEN cDNA 4933407D05 gene
Human	chr1:32340341-32343381:+	AK001409	AK001409	Homo sapiens cDNA FLJ10547 fis, clone NT2RP2001907.
Human	chr1:32339008-32341501:-	BI758608	-	-
Mouse	chr4:127978842-127989426:-	BC031493	BC031493	Mus musculus hypothetical protein LOC230767, mRNA (cDNA clone MGC:28034 IMAGE:3665627), complete cds. CDS=16..1284
Mouse	chr4:127970982-127989890:+	B230310F21	B230310F21	RIKEN cDNA 4933407D05 gene
Human	chr1:32494416-32496022:+	AK055014	AK055014	Homo sapiens cDNA FLJ30452 fis, clone BRACE2009293.
Human	chr1:32495964-32498972:-	AK125095	AK125095	Homo sapiens cDNA FLJ43105 fis, clone CTONG2012453.
Mouse	chr4:127862214-127880062:-	AI550540	-	-
Mouse	chr4:127861506-127862272:+	1700051I15	1700125D06	unclassifiable
Human	chr1:32673924-32735676:+	BC029654	AK074546	Homo sapiens cDNA FLJ90065 fis, clone HEMBA1003497, weakly similar to ZINC FINGER PROTEIN 151.
Human	chr1:32734866-32785688:-	AY158005	AY158005	Homo sapiens archease-like protein isoform ABAC (ARCH) mRNA, complete cds; alternatively spliced.
Mouse	chr4:127723944-127748476:-	2410081M15	2410081M15	weakly similar to ZINC FINGER PROTEIN 12 (ZINC FINGER PROTEIN KOX3) (FRAGMENT) [Homo sapiens]
Mouse	chr4:127705924-127724863:+	2310028N13	2310028N13	hypothetical protein
Human	chr1:32785836-32820905:+	AK056550	X74262	H.sapiens RbAp48 mRNA encoding retinoblastoma binding protein.
Human	chr1:32814471-32829966:-	W47135	-	-
Mouse	chr4:127677416-127705686:-	C230048M14	C230048M14	retinoblastoma binding protein 4
Mouse	chr4:127657945-127679698:+	AJ251641	AJ251641	Mus musculus mRNA for syncoilin (dystrobrevin binding protein). CDS=55..1467
Human	chr1:32876579-32909664:+	BC020306	AB040955	Homo sapiens mRNA for KIAA1522 protein, partial cds.
Human	chr1:32909469-32952847:-	F09118	AK125213	Homo sapiens cDNA FLJ43223 fis, clone FEBRA2026984, highly similar to TYROSYL-TRNA SYNTHETASE (EC 6.1.1.1).
Mouse	chr4:127589894-127631720:-	F630111O11	F630035N13	hypothetical Proline-rich region profile/Serine-rich region profile containing protein
Mouse	chr4:127560076-127591425:+	4922501N15	4922501N15	SIMILAR TO TYROSYL-TRNA SYNTHETASE homolog [Mus musculus]
Human	chr1:33020688-33033135:+	BC001777	BC001777	Homo sapiens, similar to hippocalcin, clone MGC:1822 IMAGE:3355354, mRNA, complete cds.
Human	chr1:33029287-33036134:-	CR596164	CR596164	full-length cDNA clone CS0DI013YC09 of Placenta Cot 25-normalized of Homo sapiens (human).
Mouse	chr4:127481885-127492063:-	0710005G22	0710005G22	hippocalcin
Mouse	chr4:127475846-127481942:+	1810017F10	BC019563	Mus musculus RIKEN cDNA 1810017F10 gene, mRNA (cDNA clone MGC:28617 IMAGE:4219997), complete cds. CDS=116..775
Human	chr1:35692167-35701970:+	AL536768	BC024592	Homo sapiens, neurochondrin, clone MGC:9465 IMAGE:3914058, mRNA, complete cds.
Human	chr1:35568184-35692306:-	BU552752	AK022734	Homo sapiens cDNA FLJ12672 fis, clone NT2RM4002339.
Mouse	chr4:125114066-125124091:-	F630106C16	F630110J24	neurochondrin
Mouse	chr4:125122515-125240827:+	M5C1080J16	A730047D20	HYPOTHETICAL 103.9 KDA PROTEIN homolog [Homo sapiens]
Human	chr1:37691613-37701551:+	BC046117	BX647913	Homo sapiens mRNA; cDNA DKFZp686M1712 (from clone DKFZp686M1712).
Human	chr1:37701506-37730646:-	BC000107	BC000107	Homo sapiens nucleolar GTPase, mRNA (cDNA clone MGC:1085 IMAGE:3506131), complete cds.
Mouse	chr4:123425637-123436972:-	4933406D15	4933406D15	DJ423B22.5 (AXONEMAL DYNEIN LIGHT CHAIN (HP28)) homolog [Homo sapiens]
Mouse	chr4:123386901-123425700:+	I730073P03	I730073P03	Testicular antigen homolog

Human	chr1:38994765-39054196:+	CB134700	-	-
Human	chr1:38972962-39076566:-	AK097621	AK097621	Homo sapiens cDNA FLJ40302 fis, clone TESTI2029196, weakly similar to RHOMBOID PROTEIN.
Mouse	chr4:122268948-122288338:-	BC019958	4632401I21	unclassifiable
Mouse	chr4:122275147-122307317:+	BC048478	I830015H15	Ras-related GTP binding C
Human	chr1:44126124-44131293:+	BX641142	AX747604	Sequence 1129 from Patent EP1308459.
Human	chr1:44131248-44166362:-	BI255560	S70612	glycine transporter type 1c {alternatively spliced} [human, substantia nigra, mRNA, 2202 nt].
Mouse	chr4:116198317-116229117:-	4930580H03	G930027M14	UDP-Gal:betaGlcNAc beta 1,4- galactosyltransferase, polypeptide 2
Mouse	chr4:116180147-116220839:+	X67056	4632413G05	glycine transporter 1
Human	chr1:44934726-44940765:+	BC004198	AJ293866	Homo sapiens mRNA for FNK serine/threonine protein kinase.
Human	chr1:44940673-44942050:-	CF541004	-	-
Mouse	chr4:115474296-115479604:-	BC063051	F830047P17	polo-like kinase 3 (Drosophila)
Mouse	chr4:115472454-115474379:+	4833401D15	4833401D15	hypothetical protein
Human	chr1:45474435-45478743:+	BC009364	AY169960	Homo sapiens target of Egr1 protein (TOE1) mRNA, complete cds.
Human	chr1:45478648-45625944:-	AK027573	AB057597	Homo sapiens mRNA for testicular protein kinase 2, complete cds.
Mouse	chr4:115139951-115153281:-	4933424D16	4933424D16	4933424D16RIK PROTEIN
Mouse	chr4:115054212-115151599:+	E130012E07	E130012E07	testis-specific kinase 2
Human	chr1:45718611-45753660:+	CR621701	BC010105	Homo sapiens nuclear autoantigenic sperm protein (histone-binding), transcript variant 2, mRNA (cDNA clone MGC:19722 IMAGE:3538429), complete cds.
Human	chr1:45717632-45719476:-	AK128017	AK128017	Homo sapiens cDNA FLJ46136 fis, clone TESTI2052202.
Mouse	chr4:114946693-115005203:-	AF034610	C920008H08	nuclear autoantigenic sperm protein (histone-binding)
Mouse	chr4:114972484-114973856:+	B930079G11	B930079G11	unclassifiable
Human	chr1:46381033-46398953:+	BC034422	CR590915	full-length cDNA clone CS0DF026YG09 of Fetal brain of Homo sapiens (human).
Human	chr1:46366368-46397997:-	AK056186	AK056186	Homo sapiens cDNA FLJ31624 fis, clone NT2RI2003301, highly similar to Homo sapiens UDP-GlcNAc:a-3-D-mannoside b1,2-N-acetylglucosaminyltransferase I.2 (MGAT1.2) mRNA.
Mouse	chr4:114482369-114496979:-	1520402A15	1520402A15	hypothetical protein
Mouse	chr4:114469481-114484491:+	2510003B16	2510003B16	unclassifiable
Human	chr1:46425380-46456175:+	X97795	X97795	H.sapiens mRNA homologous to S. cerevisiae RAD54.
Human	chr1:46445206-46509870:-	BX537366	BX537366	Homo sapiens mRNA; cDNA DKFZp686P0958 (from clone DKFZp686P0958).
Mouse	chr4:114439905-114469331:-	C130033E24	5730524N08	RAD54 like (S. cerevisiae)
Mouse	chr4:114420910-114443358:+	1190001F19	F630101F18	MUF1 protein
Human	chr1:46425380-46456175:+	X97795	X97795	H.sapiens mRNA homologous to S. cerevisiae RAD54.
Human	chr1:46455154-46455950:-	CA430007	-	-
Mouse	chr4:114439905-114469331:-	C130033E24	5730524N08	RAD54 like (S. cerevisiae)
Mouse	chr4:114420910-114443358:+	1190001F19	F630101F18	MUF1 protein
Human	chr1:51754872-51966696:+	BQ215910	AK128043	Homo sapiens cDNA FLJ46163 fis, clone TESTI4002774, weakly similar to Mus musculus oxysterol binding protein-like 5 (Osbpl5).
Human	chr1:51966543-52056494:-	AW341698	X93209	H.sapiens mRNA for NRD1 convertase.
Mouse	chr4:107406786-107547913:-	BC023759	BC023759	Mus musculus nardilysin, N-arginine dibasic convertase, NRD convertase 1, mRNA (cDNA clone IMAGE:5344071), partial cds.
Mouse	chr4:107346268-107412715:+	6430527E21	6030449O20	NARDILYSIN PRECURSOR (EC 3.4.24.61) (N-ARGININE DIBASIC CONVERTASE) (NRD CONVERTASE) homolog [Rattus norvegicus]
Human	chr1:54641558-54812438:+	AL713723	AF416921	Homo sapiens brown fat inducible thioesterase 1 mRNA, complete cds, alternatively spliced.
Human	chr1:54786871-54801250:-	AK091901	AK091901	Homo sapiens cDNA FLJ34582 fis, clone KIDNE2008649.
Mouse	chr4:105090196-105150631:-	AK122350	A230033M23	BROWN FAT INDUCIBLE THIOESTERASE 2 homolog [Mus musculus]
Mouse	chr4:105079508-105093933:+	AY168619	BC026682	Mus musculus cDNA sequence BC026682, mRNA (cDNA clone MGC:37700 IMAGE:5065146), complete cds. CDS=57..1883
Human	chr1:63770937-63837937:+	BC019920	BC019920	Homo sapiens, clone MGC:29909 IMAGE:5122261, mRNA, complete cds.
Human	chr1:63618061-63771712:-	CK819833	U37139	Human beta 3-endonexin mRNA, long form and short form, complete cds.
Mouse	chr4:98275448-98333327:+	I830082H05	G270126A03	phosphoglucomutase 2
Mouse	chr4:98094610-98275847:-	C230087M13	C230087M13	weakly similar to NUCLEAR RECEPTOR CO-ACTIVATOR NRIF3 (INTEGRIN BETA 3 BINDING PROTEIN) (BETA3-ENDONEXIN) [Homo sapiens]
Human	chr1:67102661-67167119:+	AL831987	AF515447	Homo sapiens mesoderm induction early response 1 N2-beta mRNA, complete cds, alternatively spliced.
Human	chr1:66990589-67102749:-	CN265155	BX648840	Homo sapiens mRNA; cDNA DKFZp686I09143 (from clone DKFZp686I09143).
Mouse	chr4:101460031-101511395:+	AK129405	AK129405	Mus musculus mRNA for mKIAA1610 protein. CDS=91..1755
Mouse	chr4:101380400-101460255:-	A730089E14	BC028975	Mus musculus cDNA sequence BC028975, mRNA (cDNA clone MGC:38950 IMAGE:5363117), complete cds. CDS=69..1904
Human	chr1:74910874-74944382:+	BC009514	CR617954	full-length cDNA clone CS0DD004YI23 of Neuroblastoma Cot 50-normalized of Homo sapiens (human).
Human	chr1:74882301-74911113:-	CN368109	CR595439	full-length cDNA clone CS0DF021YA14 of Fetal brain of Homo sapiens (human).
Mouse	chr3:155181199-155201866:-	5230400J09	5230400J09	hypothetical DUF207 containing protein
Mouse	chr3:155201479-155227950:+	5730435C24	5730435C24	crystallin, zeta

Human	chr1:84721906-84743892:+	BG184610	-	-
Human	chr1:84676014-84752184:-	BC065191	BC024007	Homo sapiens chitobiase, di-N-acetyl-, mRNA (cDNA clone IMAGE:4823479), complete cds.
Mouse	chr3:147029706-147070290:-	4921536I21	4921536I21	weakly similar to SPERM-SPECIFIC PROTEIN SP-2 [Homo sapiens]
Mouse	chr3:147022980-147038359:+	2210401K11	2210401K11	chitobiase, di-N-acetyl-
Human	chr1:84751837-84761324:+	BM920013	-	-
Human	chr1:84676014-84752184:-	BC024007	BC024007	Homo sapiens chitobiase, di-N-acetyl-, mRNA (cDNA clone IMAGE:4823479), complete cds.
Mouse	chr3:147022305-147025668:-	4732401J22	4732401J22	unclassifiable
Mouse	chr3:147022980-147038359:+	E430038L15	2210401K11	chitobiase, di-N-acetyl-
Human	chr1:92207560-92241193:+	BC041475	BC041475	Homo sapiens, Similar to hypothetical protein FLJ22408, clone MGC:50295 IMAGE:5244314, mRNA, complete cds.
Human	chr1:92204179-92207975:-	BM723110	-	-
Mouse	chr5:104844508-104871651:+	A830036I24	-	-
Mouse	chr5:104835031-104845717:-	A830013F20	-	-
Human	chr1:93009438-93019533:+	AK095815	AK095815	Homo sapiens cDNA FLJ38496 fis, clone FELIV1000137, highly similar to 60S RIBOSOMAL PROTEIN L5.
Human	chr1:93010474-93139078:-	BC070342	BC070342	Homo sapiens cDNA clone MGC:88347 IMAGE:6498824, complete cds.
Mouse	chr5:105342150-105350653:+	AW210754	I830067I12	ribosomal protein L5
Mouse	chr5:105349205-105428729:-	E130003P06	C730036B13	hypothetical EF-Hand-like containing protein
Human	chr1:93357497-93456308:+	AL703906	AK126045	Homo sapiens cDNA FLJ44057 fis, clone TESTI4034912, weakly similar to Intracellular protein transport protein USO1.
Human	chr1:93326861-93358305:-	BC070051	BC070051	Homo sapiens CGI-100 protein, mRNA (cDNA clone MGC:87146 IMAGE:5265257), complete cds.
Mouse	chr5:105575525-105680632:+	4922501D03	4932411G06	similar to DJ717I23.1 (NOVEL PROTEIN SIMILAR TO XENOPUS LAEVIS SOJO PROTEIN) (FRAGMENT) [Homo sapiens]
Mouse	chr5:105548213-105576039:-	4432412D15	6430594K11	hypothetical protein
Human	chr1:94997919-95072823:+	CR600415	BC033858	Homo sapiens, hypothetical protein MGC45474, clone IMAGE:5174513, mRNA, partial cds.
Human	chr1:94793929-94998054:-	CN283338	BC030750	Homo sapiens, clone IMAGE:4795773, mRNA.
Mouse	chr3:121842666-121915424:-	BC025548	BC025548	Mus musculus cDNA sequence BC010552, mRNA (cDNA clone MGC:38127 IMAGE:5320841), complete cds. CDS=297..2114
Mouse	chr3:121914640-121919542:+	9530082L20	-	-
Human	chr1:100310030-100328075:+	AK002081	BC075811	Homo sapiens hypothetical protein FLJ10287, mRNA (cDNA clone MGC:87928 IMAGE:5104751), complete cds.
Human	chr1:100326025-100355855:-	BC022420	BC022420	Homo sapiens, clone IMAGE:4245713, mRNA.
Mouse	chr3:116925914-116959774:-	4631408H19	A330067P21	CDNA FLJ11219 FIS, CLONE PLACE1008122 homolog [Homo sapiens]
Mouse	chr3:116908152-116929272:+	D330003K23	D330001M02	hypothetical Leucine-rich repeat containing protein
Human	chr1:101203430-101264956:+	BX538249	BX538249	Homo sapiens mRNA; cDNA DKFZp686H13204 (from clone DKFZp686H13204).
Human	chr1:101167200-101203665:-	BC034669	AK125485	Homo sapiens cDNA FLJ43496 fis, clone PEBLM2001465.
Mouse	chr3:116228344-116234896:-	A930005H10	A930005H10	hypothetical protein
Mouse	chr3:116234618-116281127:+	K630131I09	9430020B04	weakly similar to PUTATIVE DIPHTHINE SYNTHASE [Schizosaccharomyces pombe]
Human	chr1:108454590-108457407:+	BC053880	AK130480	Homo sapiens cDNA FLJ26970 fis, clone SLV01715.
Human	chr1:108389384-108455513:-	BC029905	BC068561	Homo sapiens solute carrier family 25 (mitochondrial carrier; phosphate carrier), member 24, transcript variant 1, mRNA (cDNA clone MGC:87498 IMAGE:5259336), complete cds.
Mouse	chr3:109500568-109503479:-	M230004L01	E130106E22	unclassifiable
Mouse	chr3:109503145-109561817:+	6720455K09	I530025F19	Peroxisomal CA-dependent solute carrier homolog
Human	chr1:109187115-109189490:+	BQ434855	AK000053	Homo sapiens cDNA FLJ20046 fis, clone COL00573.
Human	chr1:109184072-109218163:-	CR606058	AX746511	Sequence 36 from Patent EP1308459.
Mouse	chr3:109058305-109102349:-	F420001D06	F420001D06	G-protein signalling modulator 2 (AGS3-like, C. elegans)
Mouse	chr3:109033948-109059117:+	G430107A16	E430025N17	Mid-1-related chloride channel 1
Human	chr1:109874498-109886798:+	AK025706	AK025706	Homo sapiens cDNA: FLJ22053 fis, clone HEP09502, highly similar to HUMAMPD2 Human AMP deaminase (AMPD2) mRNA.
Human	chr1:109857932-109874593:-	BX090868	BC000233	Homo sapiens, Similar to guanine nucleotide binding protein, alpha transducing 2, clone MGC:2232 IMAGE:3350818, mRNA, complete cds.
Mouse	chr3:108454093-108466720:-	1200014F01	1200014F01	AMP DEAMINASE 2 (EC 3.5.4.6) (AMP DEAMINASE ISOFORM L) homolog [Homo sapiens]
Mouse	chr3:108465619-108468044:+	C630015B10	C630015B10	weakly similar to GLYCOPROTEIN [Rattus norvegicus]
Human	chr1:111394291-111439768:+	BC049196	BC049196	Homo sapiens, choline/ethanolaminephosphotransferase, clone MGC:54042 IMAGE:5226538, mRNA, complete cds.
Human	chr1:111371688-111394997:-	CR602380	CR607093	full-length cDNA clone CS0DI013YN23 of Placenta Cot 25-normalized of Homo sapiens (human).
Mouse	chr3:106881948-106927490:-	9930118K05	9930118K05	CHOLINE/ETHANOLAMINEPHOSPHOTRANSFERASE homolog [Homo sapiens]
Mouse	chr3:106927381-106955029:+	AW475537	D730039I03	hypothetical protein
Human	chr1:111394291-111439768:+	AK095229	BC049196	Homo sapiens, choline/ethanolaminephosphotransferase, clone MGC:54042 IMAGE:5226538, mRNA, complete cds.
Human	chr1:111438463-111459251:-	BU189375	AK026110	Homo sapiens cDNA: FLJ22457 fis, clone HRC09925.
Mouse	chr3:106881948-106927490:-	9930118K05	9930118K05	CHOLINE/ETHANOLAMINEPHOSPHOTRANSFERASE homolog [Homo sapiens]
Mouse	chr3:106862093-106882718:+	F830209E08	F830209E08	hypothetical dDENN domain/DENN (AEX-3) domain/uDENN domain containing protein

Human	chr1:111703523-111716077:+	BP370110	CR614214	full-length cDNA clone CS0DA003YM07 of Neuroblastoma of Homo sapiens (human).
Human	chr1:111668978-111704040:-	CR616007	AY189737	Homo sapiens oviductin mRNA, complete cds.
Mouse	chr3:106260698-106278268:-	BU938103	F830222C13	ATP synthase, H+ transporting, mitochondrial F0 complex, subunit b, isoform 1
Mouse	chr3:106277389-106305448:+	2610003I18	C130075K15	Methylosome protein 50 (MEP50 protein)
Human	chr1:112873825-112926284:+	BX648738	BX648738	Homo sapiens mRNA; cDNA DKFZp686I1935 (from clone DKFZp686I1935).
Human	chr1:112778182-112875489:-	BX537988	BX537988	Homo sapiens mRNA; cDNA DKFZp686N2430 (from clone DKFZp686N2430); complete cds.
Mouse	chr3:104771862-104814279:-	4930508K05	I920091F12	capping protein (actin filament) muscle Z-line, alpha 1
Mouse	chr3:104813174-104877252:+	4933440L10	G370016H02	suppression of tumorigenicity 7-like
Human	chr1:118184389-118215091:+	AF083217	AF083217	Homo sapiens WD repeat protein WDR3 (WDR3) mRNA, complete cds.
Human	chr1:118208330-118439888:-	AY555274	AY555274	Homo sapiens PF6 mRNA, complete cds.
Mouse	chr3:100076048-100100275:-	BC063100	BC063100	Mus musculus WD repeat domain 3, mRNA (cDNA clone MGC:70140 IMAGE:6332749), complete cds. CDS=79..2907
Mouse	chr3:100030809-100081190:+	8430401J01	8430401J01	hypothetical protein
Human	chr1:143038288-143075590:+	AK125158	AX746788	Sequence 313 from Patent EP1308459.
Human	chr1:143075158-143078482:-	CR624046	CR624046	full-length cDNA clone CS0DB005YB11 of Neuroblastoma Cot 10-normalized of Homo sapiens (human).
Mouse	chr3:96700164-96709753:+	BC023128	5730445K20	protein inhibitor of activated STAT 3
Mouse	chr3:96636400-96712342:-	6430519L11	6430519L11	hypothetical NUDIX hydrolase containing protein
Human	chr1:143184577-143185262:+	BM662912	-	-
Human	chr1:143184976-143204683:-	BC014465	AK128370	Homo sapiens cDNA FLJ46513 fis, clone THYMU3032032, weakly similar to Natural killer cell receptor BY55 precursor.
Mouse	chr3:96801751-96803357:+	AI447336	-	-
Mouse	chr3:96802470-96835826:-	B020017C16	B020017C16	CD160 antigen
Human	chr1:147043211-147050551:+	AB025904	AK074765	Homo sapiens cDNA FLJ90284 fis, clone NT2RP1000613, weakly similar to CARBONIC ANHYDRASE VI (EC 4.2.1.1).
Human	chr1:147050508-147067785:-	BE220031	BC068606	Homo sapiens cDNA clone IMAGE:5295354, with apparent retained intron.
Mouse	chr3:95892551-95899474:-	BC046995	B130021C15	carbonic anhydrase 14
Mouse	chr3:95888707-95893375:+	D030023H16	I830062N14	Gamma-secretase subunit APH-1A homolog [Mus musculus]
Human	chr1:147750973-147751653:+	AV661365	-	-
Human	chr1:147746132-147760552:-	AK001105	AK001105	Homo sapiens cDNA FLJ10243 fis, clone HEMBB1000631, weakly similar to LONGEVITY-ASSURANCE PROTEIN 1.
Mouse	chr3:95317970-95351984:-	AF091628	3425403J20	SET domain, bifurcated 1
Mouse	chr3:95309582-95318381:+	C730023N05	C730023N05	TRH3
Human	chr1:147844823-147854046:+	BC022448	BC022448	Homo sapiens, clone IMAGE:4815214, mRNA.
Human	chr1:147835799-147855874:-	CR601047	AF187845	Homo sapiens small protein effector 1 of Cdc42 mRNA, complete cds.
Mouse	chr3:95213321-95226945:-	B230333C11	B230333C11	ALL1-fused gene from chromosome 1q
Mouse	chr3:95212234-95221432:+	9430006E19	9430006E19	hypothetical protein
Human	chr1:147844823-147854046:+	BC022448	BC022448	Homo sapiens, clone IMAGE:4815214, mRNA.
Human	chr1:147835799-147855874:-	CR601047	AF187845	Homo sapiens small protein effector 1 of Cdc42 mRNA, complete cds.
Mouse	chr3:95213321-95226945:-	I830082O22	B230333C11	ALL1-fused gene from chromosome 1q
Mouse	chr3:95223134-95231672:+	BU053908	1300002M12	small protein effector 1 of Cdc42
Human	chr1:148067104-148077729:+	AA836419	CR749307	Homo sapiens mRNA; cDNA DKFZp781I1719 (from clone DKFZp781I1719).
Human	chr1:148077243-148113264:-	AB005910	BC040300	Homo sapiens, Similar to phosphatidylinositol 4-kinase, catalytic, beta polypeptide, clone MGC:42391 IMAGE:4821417, mRNA, complete cds.
Mouse	chr3:95000929-95009870:-	4931408L03	BC060179	Mus musculus RIKEN cDNA 4931408L03 gene, mRNA (cDNA clone MGC:73422 IMAGE:6831344), complete cds. CDS=136..2496
Mouse	chr3:94969087-95001294:+	F930007M02	F930007M02	phosphatidylinositol 4-kinase, catalytic, beta polypeptide
Human	chr1:148548448-148556883:+	AK125876	BC073949	Homo sapiens cDNA clone MGC:90468 IMAGE:5416827, complete cds.
Human	chr1:148552037-148552520:-	AI702988	-	-
Mouse	chr3:94419117-94422346:-	AF175297	AF175297	Mus musculus ornithine decarboxylase antizyme 3 mRNA, complete cds. CDS=207..795
Mouse	chr3:94420846-94422493:+	6330517P16	6330517P16	unclassifiable
Human	chr1:148548448-148556883:+	AK125876	BC073949	Homo sapiens cDNA clone MGC:90468 IMAGE:5416827, complete cds.
Human	chr1:148555684-148576071:-	BG109945	AF119121	Homo sapiens putative RNA binding protein mRNA, alternatively spliced, complete cds.
Mouse	chr3:94419117-94422346:-	AB045835	AF175297	Mus musculus ornithine decarboxylase antizyme 3 mRNA, complete cds. CDS=207..795
Mouse	chr3:94399003-94420582:+	6330407D06	BC057030	Mus musculus tudor and KH domain containing protein, mRNA (cDNA clone MGC:67203 IMAGE:6833743), complete cds. CDS=94..1776
Human	chr1:151819373-151848325:+	AK057347	AK057347	Homo sapiens cDNA FLJ32785 fis, clone TESTI2002251.
Human	chr1:151830738-151849493:-	CR598625	CR598625	full-length cDNA clone CS0DK011YM01 of HeLa cells Cot 25-normalized of Homo sapiens (human).
Mouse	chr3:89775197-89790231:-	4933440C21	4933440C21	similar to CDNA FLJ32785 FIS, CLONE TESTI2002251 [Homo sapiens]
Mouse	chr3:89775045-89778324:+	6430553C16	6430553C16	unclassifiable

Human	chr1:151819373-151848325:+	AK057347	AK057347	Homo sapiens cDNA FLJ32785 fis, clone TESTI2002251.
Human	chr1:151830738-151849493:-	CR598625	CR598625	full-length cDNA clone CS0DK011YM01 of HeLa cells Cot 25-normalized of Homo sapiens (human).
Mouse	chr3:89775197-89790231:-	4933440C21	4933440C21	similar to CDNA FLJ32785 FIS, CLONE TESTI2002251 [Homo sapiens]
Mouse	chr3:89778607-89779879:+	G830001D04	G830001D04	hypothetical protein
Human	chr1:151979732-151988359:+	BM723462	-	-
Human	chr1:151978452-151991979:-	L38969	L38969	Homo sapiens thrombospondin 3 (THBS3) mRNA, complete cds.
Mouse	chr3:89633643-89651640:-	CF182364	D930007L12	METAXIN 1
Mouse	chr3:89639742-89651401:+	BC053023	L04302	Mouse thrombospondin 3 (THBSP3) mRNA, complete cds. CDS=40..2910
Human	chr1:151991563-152017315:+	AA393374	BC001906	Homo sapiens, Similar to metaxin 1, clone MGC:2518 IMAGE:3546178, mRNA, complete cds.
Human	chr1:152014134-152017168:-	AK056250	AK056250	Homo sapiens cDNA FLJ31688 fis, clone NT2RI2005520.
Mouse	chr3:89633643-89651640:-	D930007L12	D930007L12	METAXIN 1
Mouse	chr3:89627487-89637992:+	G430028I15	C330011P10	acid beta glucosidase
Human	chr1:152344845-152347031:+	AK126559	AK126559	Homo sapiens cDNA FLJ44595 fis, clone BLADE2004849.
Human	chr1:152118125-152345397:-	AF257305	AF257305	Homo sapiens ASH1 mRNA, complete cds.
Mouse	chr3:89484648-89515021:-	I730069C22	6430514F02	death associated protein 3
Mouse	chr3:89514553-89552903:+	L030022K10	A630090P13	ash1 (absent, small, or homeotic)-like (Drosophila)
Human	chr1:152528649-152534449:+	BC070067	BC070067	Homo sapiens misato, mRNA (cDNA clone IMAGE:4828998), partial cds.
Human	chr1:152435786-152640081:-	AK000210	BX648802	Homo sapiens mRNA; cDNA DKFZp686A19184 (from clone DKFZp686A19184).
Mouse	chr3:89468952-89477844:-	B020012I07	B020012I07	similar to Hypothetical protein (Misato) [Homo sapiens]
Mouse	chr3:89348250-89473950:+	AK122531	AK122531	Mus musculus mRNA for mKIAA1606 protein. CDS=2..6193
Human	chr1:153511307-153519825:+	CR598294	CR598294	full-length cDNA clone CS0DC020YI02 of Neuroblastoma Cot 25-normalized of Homo sapiens (human).
Human	chr1:153504746-153511609:-	BQ432431	AK095697	Homo sapiens cDNA FLJ38378 fis, clone FEBRA2002933, weakly similar to Homo sapiens XPMC2 protein mRNA.
Mouse	chr3:88412185-88420301:-	F830033B04	BC012224	Mus musculus cDNA sequence BC023814, mRNA (cDNA clone IMAGE:4188109), with apparent retained intron.
Mouse	chr3:88419898-88430272:+	F830002O09	E130308C23	similar to CDNA FLJ12671 FIS, CLONE NT2RM4002323, WEAKLY SIMILAR TO ANTIGEN GOR (SIMILAR TO HYPOTHETICAL PROTEIN FLJ12484) [Homo sapiens]
Human	chr1:157900935-157910190:+	BC046149	BC046149	Homo sapiens, Similar to nitrilase 1, clone MGC:57670 IMAGE:5764281, mRNA, complete cds.
Human	chr1:157903681-157915633:-	AK001497	AK001497	Homo sapiens cDNA FLJ10635 fis, clone NT2RP2005669, highly similar to Homo sapiens mRNA for DEDD protein.
Mouse	chr1:171411803-171416877:-	1300013F05	1300013F05	nitrilase 1
Mouse	chr1:171400040-171414506:+	6330408H11	F830226L08	death effector domain-containing
Human	chr1:158008907-158026160:+	BX537951	BX537951	Homo sapiens mRNA; cDNA DKFZp686I2086 (from clone DKFZp686I2086); complete cds.
Human	chr1:158012528-158021073:-	Z30425	Z30425	H.sapiens mRNA for orphan nuclear hormone receptor.
Mouse	chr1:171287243-171293746:-	C030016B11	3830431O15	NUCLEAR RECEPTOR SUBFAMILY 1, GROUP I, MEMBER 3
Mouse	chr1:171285202-171291933:+	C730009H01	C730009H01	nuclear receptor subfamily 1, group I, member 3
Human	chr1:158451216-158458612:+	BC006286	BC006286	Homo sapiens, dual specificity phosphatase 12, clone MGC:10337 IMAGE:3958403, mRNA, complete cds.
Human	chr1:158449985-158451443:-	BM474343	-	-
Mouse	chr1:170951321-170962596:-	AF268196	AF268196	Mus musculus strain BALB/c VH1 mRNA, complete cds. CDS=59..1078
Mouse	chr1:170962199-170963013:+	2500001D14	2500001D14	hypothetical protein
Human	chr1:162866833-163036657:+	AA331511	-	-
Human	chr1:162770194-162867819:-	CR613414	CR613414	full-length cDNA clone CS0DJ006YP06 of T cells (Jurkat cell line) Cot 10-normalized of Homo sapiens (human).
Mouse	chr1:166999073-167026349:-	K630085B10	BC056189	Mus musculus cDNA clone IMAGE:5711055, partial cds.
Mouse	chr1:167025567-167116562:+	C030014K22	C030014K22	hypothetical protein
Human	chr1:165807218-165833618:+	U16799	X03747	Human mRNA for Na/K-ATPase beta subunit.
Human	chr1:165833005-166068859:-	AF153191	AK094513	Homo sapiens cDNA FLJ37194 fis, clone BRALZ2005467, highly similar to Homo sapiens nm23-H7 (NME7) mRNA.
Mouse	chr1:164453858-164475086:-	1500005L15	1200016M21	ATPase, Na+/K+ transporting, beta 1 polypeptide
Mouse	chr1:164321438-164454497:+	4932418E06	4932418E06	non-metastatic cells 7, protein expressed in
Human	chr1:166362903-166555157:+	AK023532	AK127098	Homo sapiens cDNA FLJ45155 fis, clone BRAWH3042816.
Human	chr1:166549843-166595066:-	BX647352	BX647352	Homo sapiens mRNA; cDNA DKFZp686J19244 (from clone DKFZp686J19244).
Mouse	chr1:163961283-164122432:-	D030013A14	B020028F21	hypothetical Short-chain dehydrogenase/reductase SDR containing protein
Mouse	chr1:163944390-163970417:+	BC043085	A330062M24	similar to Ezrin-binding partner PACE-1 [Homo sapiens]
Human	chr1:166563454-166573621:+	T08160	-	-
Human	chr1:166549843-166595066:-	BX647352	BX647352	Homo sapiens mRNA; cDNA DKFZp686J19244 (from clone DKFZp686J19244).
Mouse	chr1:163961283-164122432:-	D030013A14	B020028F21	hypothetical Short-chain dehydrogenase/reductase SDR containing protein
Mouse	chr1:163944390-163970417:+	BC043085	A330062M24	similar to Ezrin-binding partner PACE-1 [Homo sapiens]

Human	chr1:174794594-175175215:+	BC030292	AF047711	Homo sapiens nGAP mRNA, complete cds.
Human	chr1:174792300-174795270:-	BG826421	BC027927	Homo sapiens, clone IMAGE:5200165, mRNA.
Mouse	chr1:157140630-157414116:-	A330066M24	A330066M24	RAS GTPASE-ACTIVATING PROTEIN NGAP homolog [Homo sapiens]
Mouse	chr1:157413873-157421770:+	BC055845	BC055845	Mus musculus RIKEN cDNA 2810025M15 gene, mRNA (cDNA clone MGC:67868 IMAGE:3585495), complete cds. CDS=493..846
Human	chr1:176582834-176620868:+	AK001780	AK023204	Homo sapiens cDNA FLJ13142 fis, clone NT2RP3003212, moderately similar to Rattus norvegicus lamina associated polypeptide 1C (LAP1C) mRNA.
Human	chr1:176582399-176583387:-	BC004969	BC004969	Homo sapiens, clone MGC:3413 IMAGE:2906290, mRNA, complete cds.
Mouse	chr1:155994299-156027428:-	G530013G01	BC010841	Mus musculus hypothetical protein MGC6357, mRNA (cDNA clone MGC:6357 IMAGE:3493883), complete cds. CDS=61..1623
Mouse	chr1:156026244-156059731:+	A130072J07	A130072J07	weakly similar to CDNA FLJ13142 FIS, CLONE NT2RP3003212, MODERATELY SIMILAR TO RATTUS NORVEGICUS LAMINA ASSOCIATED POLYPEPTIDE 1C (LAP1C) MRNA [Homo sapiens]
Human	chr1:182997068-183015356:+	U70136	AK057197	Homo sapiens cDNA FLJ32635 fis, clone SYNOV2000178, highly similar to Human megakaryocyte stimulating factor mRNA.
Human	chr1:183012441-183076545:-	U69668	X66397	H.sapiens tpr mRNA.
Mouse	chr1:150392155-150408504:-	AB034730	AB034730	Mus musculus mRNA, complete cds, similar to megakaryocyte stimulating factor precursor and cartilage superficial zone protein. CDS=41..3205
Mouse	chr1:150335192-150392274:+	AY046504	4022443J18	translocated promoter region
Human	chr1:198683157-198706992:+	BC001064	AK097372	Homo sapiens cDNA FLJ40053 fis, clone TBAES2000255, highly similar to AMINOPEPTIDASE B (EC 3.4.11.6).
Human	chr1:198703515-198705990:-	BX648806	BX648806	Homo sapiens mRNA; cDNA DKFZp686E11190 (from clone DKFZp686E11190).
Mouse	chr1:135151403-135173102:-	BC019200	I0C0050K22	arginyl aminopeptidase (aminopeptidase B)
Mouse	chr1:135137255-135155137:+	4932433E14	4932433E14	unclassifiable
Human	chr1:199791439-199868190:+	AK127752	L22214	Human adenosine A1 receptor (ADORA1) mRNA exons 1-6, complete cds.
Human	chr1:199828044-199828739:-	AI859182	-	-
Mouse	chr1:134087814-134125915:-	A230061M23	A830058L03	adenosine A1 receptor
Mouse	chr1:134125069-134128077:+	E330018H16	E330018H16	unclassifiable
Human	chr1:202269762-202303707:+	BC036549	AK090831	Homo sapiens cDNA FLJ33512 fis, clone BRAMY2005391.
Human	chr1:202255057-202270081:-	BG703444	AX746585	Sequence 110 from Patent EP1308459.
Mouse	chr1:131895102-131940271:-	BC058790	BC058790	Mus musculus cDNA clone MGC:67710 IMAGE:6395070, complete cds. CDS=20..1558
Mouse	chr1:131937159-131940117:+	G530004A21	G530004A21	unclassifiable
Human	chr1:206317772-206344063:+	AK125601	BX648450	Homo sapiens mRNA; cDNA DKFZp686C0363 (from clone DKFZp686C0363).
Human	chr1:206340948-206367882:-	AK022798	CR601385	full-length cDNA clone CS0DI041YK12 of Placenta Cot 25-normalized of Homo sapiens (human).
Mouse	chr1:193069207-193095405:-	BC036561	6030423D04	BM282D4.3.1 (NOVEL PROTEIN (ISOFORM 1))
Mouse	chr1:193046814-193071534:+	BC055955	BC008515	Mus musculus interferon regulatory factor 6, mRNA (cDNA clone MGC:5918 IMAGE:3592582), complete cds. CDS=142..1545
Human	chr1:208139818-208140476:+	AI621266	-	-
Human	chr1:208133305-208140115:-	AF364518	BX647120	Homo sapiens mRNA; cDNA DKFZp686E1753 (from clone DKFZp686E1753).
Mouse	chr1:191786381-191803679:-	A630024D08	9630030J22	Hypothetical histidine-rich region containing protein
Mouse	chr1:191802919-191809399:+	BC052166	G830042M08	solute carrier family 30 (zinc transporter), member 1
Human	chr1:216655839-216710821:+	AA102067	D28500	Homo sapiens mRNA for mitochondrial isoleucine tRNA synthetase, partial cds.
Human	chr1:216710017-216834260:-	AK021928	AK021928	Homo sapiens cDNA FLJ11866 fis, clone HEMBA1006973, highly similar to Homo sapiens rab3-GAP regulatory domain mRNA.
Mouse	chr1:184922930-184967605:-	6430402E09	A830011B11	similar to MITOCHONDRIAL ISOLEUCINE TRNA SYNTHETASE (FRAGMENT) [Homo sapiens]
Mouse	chr1:184842371-184924954:+	BC052505	BC057872	Mus musculus expressed sequence AW743433, mRNA (cDNA clone MGC:67698 IMAGE:5294131), complete cds. CDS=251..3256
Human	chr1:231048967-231049406:+	BE467114	-	-
Human	chr1:231046745-231052663:-	BC020516	BC020516	Homo sapiens, clone IMAGE:3882977, mRNA, partial cds.
Mouse	chr8:125951425-125974566:+	D030024A19	D030024A19	unclassifiable
Mouse	chr8:125971874-125976515:-	B130064H07	E130305N23	BRAIN MY039 PROTEIN homolog [Homo sapiens]
Human	chr1:231856716-231938324:+	AK093397	CR599260	full-length cDNA clone CS0DM004YE11 of Fetal liver of Homo sapiens (human).
Human	chr1:231936546-231993822:-	W52420	BC029564	Homo sapiens, clone MGC:39558 IMAGE:4826983, mRNA, complete cds.
Mouse	chr13:13331414-13412862:-	I530011E22	BC006798	Mus musculus geranylgeranyl diphosphate synthase 1, mRNA (cDNA clone MGC:11417 IMAGE:2651229), complete cds. CDS=205..915
Mouse	chr13:13303624-13348348:+	I830033H23	I830033H23	Hypothetical glycosyltransferase family 31 containing protein
Human	chr1:232631873-232711206:+	AF027826	AF027826	Homo sapiens putative seven pass transmembrane protein (TM7SF1) mRNA, complete cds.
Human	chr1:232695188-232700610:-	AL832142	AL832142	Homo sapiens mRNA; cDNA DKFZp686A22111 (from clone DKFZp686A22111).
Mouse	chr13:12554780-12719733:-	AF154337	AF154337	Mus musculus putative seven pass transmembrane protein (Tm7sf1) mRNA, complete cds. CDS=95..1252
Mouse	chr13:12554780-12561672:+	A830099C19	A830099C19	unclassifiable
Human	chr1:233007348-233042322:+	BC016486	AK098613	Homo sapiens cDNA FLJ25747 fis, clone TST06105, highly similar to Homo sapiens Po66 carbohydrate binding protein 1 mRNA.
Human	chr1:233029435-233093856:-	BC065205	AK125663	Homo sapiens cDNA FLJ43675 fis, clone SYNOV4008440, highly similar to Protein BAP28.
Mouse	chr13:12385979-12411076:-	BC040243	4921513M21	lectin, galactose binding, soluble 8
Mouse	chr13:12340946-12386322:+	3425401O09	F530006K24	Similar to protein BAP28 (Fragment)

Human	chr1:239744949-240007155:+	AF039690	AF250731	Homo sapiens centrosomal colon cancer autoantigen protein (CCCAP) mRNA, complete cds.
Human	chr1:239977576-240332927:-	AY005799	AY005799	Homo sapiens protein kinase B gamma 1 (AKT3) mRNA, complete cds, alternatively spliced.
Mouse	chr1:176944450-177151064:+	AF250729	AF250729	Mus musculus centrosomal colon cancer autoantigen protein (Cccap) mRNA, complete cds. CDS=330..2483
Mouse	chr1:177150444-177379260:-	CD774429	AF124142	Mus musculus protein kinase B gamma mRNA, complete cds. CDS=37..1476
Human	chr1:241324674-241334404:+	AF086182	CR592197	full-length cDNA clone CS0DF034YM21 of Fetal brain of Homo sapiens (human).
Human	chr1:241329981-241354304:-	AF448858	AK126868	Homo sapiens cDNA FLJ44920 fis, clone BRAMY3011501, moderately similar to Heterogenous nuclear ribonucleoprotein U.
Mouse	chr1:178334666-178338987:+	2310005N03	BC059717	Mus musculus cDNA clone MGC:74109 IMAGE:6543216, complete cds. CDS=16..369
Mouse	chr1:178337024-178353218:-	BC033430	4932409F19	heterogeneous nuclear ribonucleoprotein U
Human	chr2:10812373-10875835:+	AK127836	BC012142	Homo sapiens, clone MGC:20253 IMAGE:4647654, mRNA, complete cds.
Human	chr2:10858514-10928701:-	AK127433	AK131234	Homo sapiens cDNA FLJ16143 fis, clone BRAMY2038516, highly similar to PROBABLE PROTEIN DISULFIDE ISOMERASE P5 PRECURSOR (EC 5.3.4.1).
Mouse	chr12:17410544-17451843:-	AB088357	AB088357	Mus musculus Atp6c2 mRNA for proton-translocating ATPase C subunit isoform C2, complete cds. CDS=131..1414
Mouse	chr12:17392367-17410595:+	4921509C01	4921509C01	PROTEIN DISULFIDE ISOMERASE A6 PRECURSOR (EC 5.3.4.1) (PROTEIN DISULFIDE ISOMERASE P5) homolog [Mesocricetus auratus]
Human	chr2:24211047-24304158:+	BI832169	BC029459	Homo sapiens, clone IMAGE:4305460, mRNA.
Human	chr2:24249621-24258221:-	BC043236	BC029523	Homo sapiens, similar to data source:SPTR, source key:P39825, evidence:ISS~putative~related to PROFILIN, clone MGC:33886 IMAGE:5297707, mRNA, complete cds.
Mouse	chr12:4615015-4648304:-	A830093I24	A830093I24	weakly similar to CDNA FLJ30851 FIS, CLONE FEBRA2002908 [Homo sapiens]
Mouse	chr12:4647868-4657850:+	K230053I20	2900024P18	hypothetical Profilin/allergen containing protein
Human	chr2:24927635-24957354:+	CB960814	AK027859	Homo sapiens cDNA FLJ14953 fis, clone PLACE3000160.
Human	chr2:24924255-24927902:-	BC073803	BC073803	Homo sapiens cDNA clone MGC:88825 IMAGE:4896064, complete cds.
Mouse	chr12:4063868-4106969:-	4831430H22	2810429O05	hypothetical protein
Mouse	chr12:4106718-4113023:+	2410017P09	2410017P09	weakly similar to CG14903 PROTEIN [Drosophila melanogaster]
Human	chr2:24927635-24957354:+	AK027859	AK027859	Homo sapiens cDNA FLJ14953 fis, clone PLACE3000160.
Human	chr2:24953689-25054568:-	BC020148	AK122926	Homo sapiens cDNA FLJ16612 fis, clone TESTI4012010, highly similar to Adenylate cyclase type III (EC 4.6.1.1).
Mouse	chr12:4063868-4106969:-	2810429O05	2810429O05	hypothetical protein
Mouse	chr12:3998037-4079786:+	BC057316	AK122298	Mus musculus mRNA for mKIAA0511 protein. CDS=450..3902
Human	chr2:27185527-27205141:+	BC007415	BC007415	Homo sapiens hypothetical protein FLJ21839, mRNA (cDNA clone MGC:2851 IMAGE:2967512), complete cds.
Human	chr2:27204731-27206371:-	AL049447	AL049447	Homo sapiens mRNA; cDNA DKFZp586A0722 (from clone DKFZp586A0722).
Mouse	chr5:29295601-29315150:+	9530085P07	D030060E19	similar to OVARC1001879 PROTEIN [Homo sapiens]
Mouse	chr5:29314070-29315973:-	2310016E02	2310016E02	P11F3 homolog [Xenopus laevis]
Human	chr2:27258308-27265331:+	BC056403	BC031868	Homo sapiens, Similar to abhydrolase domain containing 1, clone IMAGE:5164180, mRNA.
Human	chr2:27265275-27269194:-	BC008658	BC041032	Homo sapiens, prolactin regulatory element binding, clone MGC:52403 IMAGE:4638313, mRNA, complete cds.
Mouse	chr5:29358249-29363325:+	BC013505	F830020N19	abhydrolase domain containing 1
Mouse	chr5:29359853-29368540:-	4931409I21	4931409I21	prolactin regulatory element binding
Human	chr2:27346432-27351697:+	CR600041	CR600041	full-length cDNA clone CS0DC021YJ02 of Neuroblastoma Cot 25-normalized of Homo sapiens (human).
Human	chr2:27334105-27347469:-	AF288781	AF069307	Homo sapiens sodium-dependent multivitamin transporter (SMVT) mRNA, complete cds.
Mouse	chr5:29456491-29462812:+	K430357D17	K430357D17	Similar to apoptosis related protein APR-3 homolog [Mus musculus]
Mouse	chr5:29444213-29457103:-	K430360E18	BC065061	Mus musculus solute carrier family 5 (sodium-dependent vitamin transporter), member 6, mRNA (cDNA clone IMAGE:6416028), containing frame-shift errors.
Human	chr2:28811338-28985127:+	BC047224	AF092905	Homo sapiens protein phosphatase type-1 catalytic subunit delta isoform (PPCS1D) mRNA, complete cds.
Human	chr2:28944798-29004826:-	BC000952	BC000952	Homo sapiens, hypothetical protein FLJ20628, clone MGC:4955 IMAGE:3450078, mRNA, complete cds.
Mouse	chr17:69959448-70021960:+	4921517J08	4921517J08	hypothetical protein
Mouse	chr17:69989450-70031056:-	6430540A02	6430540A02	unclassifiable
Human	chr2:37335286-37351957:+	BC051746	BC065768	Homo sapiens cDNA clone IMAGE:4738534, partial cds.
Human	chr2:37340361-37370397:-	M37197	M37197	Human CCAAT-box-binding factor (CBF) mRNA, complete cds.
Mouse	chr17:77467296-77471845:+	1110001A16	1110001A16	hypothetical protein
Mouse	chr17:77469800-77487887:-	5730409M10	K920015C02	CCAAT/enhancer binding protein zeta
Human	chr2:39096847-39098863:+	BX108565	-	-
Human	chr2:39098082-39099150:-	BC030087	BC030087	Homo sapiens hypothetical protein LOC375196, mRNA (cDNA clone IMAGE:4792618), partial cds.
Mouse	chr17:78928115-78951811:+	AI844809	9630010N06	unclassifiable
Mouse	chr17:78928775-78930460:-	9430015L11	9430015L11	hypothetical protein
Human	chr2:44414250-44459614:+	D82326	AB033549	Homo sapiens mRNA for hrBAT, complete cds.
Human	chr2:44455071-44500595:-	AB007896	AB007896	Homo sapiens KIAA0436 mRNA, partial cds.
Mouse	chr17:83599814-83639376:+	D88533	3830420H12	solute carrier family 3, member 1
Mouse	chr17:83633782-83661985:-	BC004612	BC004612	Mus musculus RIKEN cDNA D030028O16 gene, mRNA (cDNA clone MGC:7980 IMAGE:3585402), complete cds. CDS=165..2081

Human	chr2:44460027-44461227:+	BG431163	-	-
Human	chr2:44455071-44500595:-	AK131463	AB007896	Homo sapiens KIAA0436 mRNA, partial cds.
Mouse	chr17:83599814-83639376:+	D88533	3830420H12	solute carrier family 3, member 1
Mouse	chr17:83633782-83661985:-	BC004612	BC004612	Mus musculus RIKEN cDNA D030028O16 gene, mRNA (cDNA clone MGC:7980 IMAGE:3585402), complete cds. CDS=165..2081
Human	chr2:46680686-46724028:+	CR594469	CR624597	full-length cDNA clone CS0DE014YM02 of Placenta of Homo sapiens (human).
Human	chr2:46719727-46755902:-	BC029408	BC029408	Homo sapiens, phosphatidylinositol glycan, class F, clone MGC:32646 IMAGE:4664848, mRNA, complete cds.
Mouse	chr17:85561094-85598080:+	BC056363	BC056363	Mus musculus ras homolog gene family, member Q, mRNA (cDNA clone MGC:73481 IMAGE:6821501), complete cds. CDS=496..1113
Mouse	chr17:85595265-85623418:-	BC028862	4930404E18	phosphatidylinositol glycan, class F
Human	chr2:47834325-47946943:+	U28946	BC071594	Homo sapiens mutS homolog 6 (E. coli), mRNA (cDNA clone IMAGE:4374146), partial cds.
Human	chr2:47923917-48044632:-	BC043258	BC043258	Homo sapiens, clone MGC:44383 IMAGE:5296581, mRNA, complete cds.
Mouse	chr17:86576978-86592855:+	E430008K24	E430008K24	mutS homolog 6 (E. coli)
Mouse	chr17:86592077-86668015:-	BC055343	BC055343	Mus musculus F-box only protein 11, mRNA (cDNA clone IMAGE:6811498), partial cds. CDS=3..1568
Human	chr2:55370716-55374693:+	BE901447	S79522	ubiquitin carboxyl extension protein [human, mRNA, 540 nt].
Human	chr2:55314329-55371350:-	BC036908	BC036908	Homo sapiens, clone MGC:46427 IMAGE:5166064, mRNA, complete cds.
Mouse	chr11:29437502-29443676:-	AW519710	0610006J14	ribosomal protein S27a
Mouse	chr11:29443517-29473919:+	1700034F02	1700034F02	hypothetical ARM repeat structure containing protein
Human	chr2:55558063-55560012:+	CR619219	CR619219	full-length cDNA clone CS0DN004YD15 of Adult brain of Homo sapiens (human).
Human	chr2:55426629-55957149:-	AF112218	AF112218	Homo sapiens unknown mRNA.
Mouse	chr11:29267897-29269843:-	A530093K08	A530093K08	unclassifiable
Mouse	chr11:29269629-29343533:+	AI452126	-	-
Human	chr2:58046437-58298667:+	BC036434	BC036434	Homo sapiens, similar to vaccinia related kinase 2, clone IMAGE:4815217, mRNA.
Human	chr2:58298029-58380166:-	BC054517	BC054517	Homo sapiens Fanconi anemia, complementation group L, mRNA (cDNA clone MGC:60295 IMAGE:5180184), complete cds.
Mouse	chr11:26366968-26489566:-	BC013520	F630038O09	vaccinia related kinase 2
Mouse	chr11:26282651-26367450:+	AF513619	AF513619	Mus musculus proliferation of germ cells protein (Pog) mRNA, complete cds. CDS=132..1259
Human	chr2:61156011-61188045:+	BC025779	BC067090	Homo sapiens peroxisome biogenesis factor 13, mRNA (cDNA clone MGC:71242 IMAGE:6285875), complete cds.
Human	chr2:61076682-61157045:-	AL832208	AL832208	Homo sapiens mRNA; cDNA DKFZp686K122 (from clone DKFZp686K122).
Mouse	chr11:23542046-23561526:-	E330040G18	6030495B12	peroxisomal biogenesis factor 13
Mouse	chr11:23561025-23628446:+	4931417G23	4931417G23	hypothetical ATP/GTP-binding site motif A (P-loop) containing protein
Human	chr2:61316202-61329996:+	AL050376	BC050395	Homo sapiens AHA1, activator of heat shock 90kDa protein ATPase homolog 2 (yeast), mRNA (cDNA clone IMAGE:5735095), partial cds.
Human	chr2:61325564-61609640:-	BC022783	AB011142	Homo sapiens mRNA for KIAA0570 protein, partial cds.
Mouse	chr11:23383162-23393606:-	BC038397	BC038397	Mus musculus, Similar to hypothetical protein DKFZp564C236, clone IMAGE:3676293, mRNA.
Mouse	chr11:23299133-23385654:+	AK129165	AK129165	Mus musculus mRNA for mKIAA0570 protein. CDS=2..3667
Human	chr2:63979745-64030916:+	CR602817	CR602817	full-length cDNA clone CS0DJ003YP10 of T cells (Jurkat cell line) Cot 10-normalized of Homo sapiens (human).
Human	chr2:63977966-63980928:-	AK094167	AK094167	Homo sapiens cDNA FLJ36848 fis, clone ASTRO2013802.
Mouse	chr11:21216464-21266837:-	BC061208	BC025585	Mus musculus UDP-glucose pyrophosphorylase 2, mRNA (cDNA clone MGC:38262 IMAGE:5324928), complete cds. CDS=213..1706
Mouse	chr11:21265970-21267576:+	A230049O18	-	-
Human	chr2:65195146-65225795:+	AB011154	AB011154	Homo sapiens mRNA for KIAA0582 protein, partial cds.
Human	chr2:65209486-65269194:-	AK055927	AK055927	Homo sapiens cDNA FLJ31365 fis, clone NB9N41000135, highly similar to RAS-RELATED PROTEIN RAB-1A.
Mouse	chr11:20121985-20144999:-	G370069N16	C130034I15	hypothetical Spectrin repeat containing protein
Mouse	chr11:20096836-20122427:+	AF226873	G830025A07	RAB1, member RAS oncogene family
Human	chr2:68296614-68315781:+	BC008304	BC008304	Homo sapiens, putatative 28 kDa protein, clone MGC:15140 IMAGE:3344225, mRNA, complete cds.
Human	chr2:68261719-68400013:-	AY183476	AY183476	Homo sapiens HZGJ (HZGJ) mRNA, complete cds.
Mouse	chr11:17098765-17107159:-	I730062N24	I730062N24	Putatative 28 kDa protein (RNA-binding protein LOC56902) homolog [Homo sapiens]
Mouse	chr11:16947376-17129372:+	AY266418	AY266418	Mus musculus HZGJ-like protein mRNA, complete cds. CDS=414..1253
Human	chr2:74656743-74657867:+	BQ943201	-	-
Human	chr2:74656913-74665063:-	AK074337	AK074337	Homo sapiens cDNA FLJ23757 fis, clone HEP17946, highly similar to Mus musculus DEAQ RNA-dependent ATPase DQX1 mRNA.
Mouse	chr6:83302874-83304824:-	D130060A17	D130060A17	DEAQ RNA-dependent ATPase
Mouse	chr6:83295351-83304721:+	BC048936	AF318278	Mus musculus DEAQ RNA-dependent ATPase DQX1 (Dqx1) mRNA, complete cds. CDS=19..2175
Human	chr2:74668177-74672407:+	AF141305	AF141305	Homo sapiens serine protease Htra2 mRNA, alternatively spliced, complete cds.
Human	chr2:74665430-74668721:-	BC001658	BC001658	Homo sapiens, Similar to ancient ubiquitous protein 1, clone IMAGE:3050204, mRNA.
Mouse	chr6:83288769-83292776:-	2610024J09	2610024J09	protease, serine, 25
Mouse	chr6:83291971-83295311:+	F630104C13	F630104C13	ancient ubiquitous protein

Human	chr2:74668177-74672407:+	BC000096	AF141305	Homo sapiens serine protease Htra2 mRNA, alternatively spliced, complete cds.
Human	chr2:74671220-74694472:-	AF311313	AF311313	Homo sapiens lysyl oxidase-like 3 protein (LOXL3) mRNA, complete cds.
Mouse	chr6:83288769-83292776:-	I730057O11	2610024J09	protease, serine, 25
Mouse	chr6:83271676-83290069:+	5330429N11	5330429N11	lysyl oxidase-like 3
Human	chr2:85736913-85737370:+	AW205448	-	-
Human	chr2:85737328-85741977:-	AK074505	AK074505	Homo sapiens cDNA FLJ90024 fis, clone HEMBA1000962.
Mouse	chr6:72586447-72593706:-	BC005559	1700047F16	weakly similar to RING-H2 FINGER PROTEIN RHA4A (FRAGMENT) [Arabidopsis thaliana]
Mouse	chr6:72582098-72586938:+	BC028825	BC028825	Mus musculus cDNA sequence BC014685, mRNA (cDNA clone MGC:37802 IMAGE:5097962), complete cds. CDS=182..997
Human	chr2:96757829-96792495:+	AL133022	AL133022	Homo sapiens mRNA; cDNA DKFZp434I0121 (from clone DKFZp434I0121).
Human	chr2:96789957-96791315:-	BC034928	BC034928	Homo sapiens, clone IMAGE:4300626, mRNA.
Mouse	chr1:36739347-36744569:+	4930533C12	4930533C12	hypothetical C2 domain/C2 calcium/lipid-binding domain, CaLB containing protein
Mouse	chr1:36741181-36767944:-	5832448C19	E430029M19	lectin, mannose-binding 2-like
Human	chr2:101077225-101099012:+	AK098124	BC070210	Homo sapiens cDNA clone MGC:88191 IMAGE:4714258, complete cds.
Human	chr2:101080913-101165345:-	AB024057	AB024057	Homo sapiens mRNA for vascular Rab-GAP/TBC-containing protein, complete cds.
Mouse	chr1:39690378-39694423:+	BC008223	3200001N24	ribosomal protein L31
Mouse	chr1:39694007-39801267:-	U88873	C630001K10	TBC1 domain family, member 8
Human	chr2:113119694-113137635:+	BC019944	BC019944	Homo sapiens, clone MGC:8767 IMAGE:3918690, mRNA, complete cds.
Human	chr2:113115638-113119916:-	CR597666	AK124342	Homo sapiens cDNA FLJ42351 fis, clone UTERU2005664.
Mouse	chr2:128947787-128960640:+	F630308K18	F630308K18	solute carrier family 20, member 1
Mouse	chr2:128946275-128948217:-	A730008M19	A730008M19	unclassifiable
Human	chr2:128119788-128129996:+	BX538082	BX538082	Homo sapiens mRNA; cDNA DKFZp686M18273 (from clone DKFZp686M18273); complete cds.
Human	chr2:128112186-128155315:-	CR621396	AF527766	Homo sapiens tissue-type spleen LIM-like protein 2C mRNA, complete cds.
Mouse	chr18:32400085-32406722:-	BE654960	AY255543	Mus musculus G protein-coupled receptor GPR17 mRNA, partial cds. CDS=1..531
Mouse	chr18:32379370-32415706:+	BC010816	BC010816	Mus musculus LIM and senescent cell antigen like domains 2, mRNA (cDNA clone MGC:19129 IMAGE:4212305), complete cds. CDS=35..1060
Human	chr2:128175312-128177679:+	AK074549	AK074549	Homo sapiens cDNA FLJ90068 fis, clone HEMBA1003732, weakly similar to SFT2 PROTEIN.
Human	chr2:128174826-128175657:-	BM711310	-	-
Mouse	chr18:32366180-32368360:-	2610206C24	2610206C24	2610206C24RIK PROTEIN (FRAGMENT)
Mouse	chr18:32367387-32369005:+	BC062274	1810036N14	hypothetical protein
Human	chr2:128359325-128360993:+	BQ220962	-	-
Human	chr2:128335432-128359849:-	BC078174	BC078174	Homo sapiens hypothetical protein MGC4268, mRNA (cDNA clone MGC:88063 IMAGE:6012672), complete cds.
Mouse	chr18:32215582-32217750:-	6230410K11	6230410K11	expressed sequence AU022236
Mouse	chr18:32216913-32241169:+	K1C0001J24	4930554M16	weakly similar to AMME syndrome candidate gene 1 protein [Homo sapiens]
Human	chr2:130816028-130821950:+	AF054996	CR619192	full-length cDNA clone CS0DN002YJ07 of Adult brain of Homo sapiens (human).
Human	chr2:130811736-130816450:-	BM851653	AK054693	Homo sapiens cDNA FLJ30131 fis, clone BRACE1000159.
Mouse	chr1:34723097-34732944:+	AI117064	2810453C09	hypothetical CHROMOSOME RIBONUCLEOPROTEIN U3 IMP4 STATE 40 DNA NUCLEOLAR containing protein
Mouse	chr1:34720134-34723273:-	BC019430	BC019430	Mus musculus RIKEN cDNA 2310061I09 gene, mRNA (cDNA clone MGC:25689 IMAGE:3491825), complete cds. CDS=336..878
Human	chr2:133007879-133237867:+	AK123759	AK122643	Homo sapiens cDNA FLJ16071 fis, clone HSYRA2001003, highly similar to PUTATIVE G PROTEIN-COUPLED RECEPTOR GPR39.
Human	chr2:133236069-133262802:-	AK130005	AK130005	Homo sapiens cDNA FLJ26495 fis, clone KDN06425.
Mouse	chr1:125671305-125704469:+	4933415E13	4933415E13	similar to PUTATIVE G PROTEIN-COUPLED RECEPTOR GPR39 [Homo sapiens]
Mouse	chr1:125703335-125743510:-	B230214B19	B230214B19	Hypothetical snake toxin-like structure containing protein
Human	chr2:135429849-135493377:+	BC016018	BC016018	Homo sapiens, clone MGC:27478 IMAGE:4714682, mRNA, complete cds.
Human	chr2:135432699-135509967:-	CD365509	-	-
Mouse	chr1:127568725-127607310:+	F530007C24	F530007C24	amino carboxymuconate semialdehyde decarboxylase
Mouse	chr1:127592948-127613415:-	2900009J06	2900009J06	unclassifiable
Human	chr2:145110913-145112204:+	BE048858	-	-
Human	chr2:144979300-145111692:-	AB056507	AB056507	Homo sapiens ZFHX1B mRNA for Smad interacting protein 1, complete cds.
Mouse	chr2:45071210-45074078:+	CB204594	-	-
Mouse	chr2:44946782-45077386:-	AJ535778	BC060699	Mus musculus zinc finger homeobox 1b, mRNA (cDNA clone MGC:66977 IMAGE:6408845), complete cds. CDS=264..3911
Human	chr2:148435917-148522128:+	AI142075	M93415	Human activin type II receptor mRNA, complete cds.
Human	chr2:148521698-148612868:-	AK128860	AF022108	Homo sapiens putative replication initiator origin recognition complex subunit Orc4Lp (ORC4L) mRNA, complete cds.
Mouse	chr2:48774100-48863260:+	D330024F24	M65287	Mouse activin receptor (ActR) mRNA, complete cds. CDS=71..1612
Mouse	chr2:48862456-48910268:-	I920075D11	G270054J23	origin recognition complex, subunit 4-like (S. cerevisiae)

Human	chr2:148612350-148616862:+	AA417220	-	-
Human	chr2:148521698-148612868:-	AV708885	AF022108	Homo sapiens putative replication initiator origin recognition complex subunit Orc4Lp (ORC4L) mRNA, complete cds.
Mouse	chr2:48909501-49282275:+	C530034A12	F630328P13	hypothetical Methyl-CpG binding/Proline-rich region profile containing protein
Mouse	chr2:48862456-48910268:-	L930246N15	G270054J23	origin recognition complex, subunit 4-like (S. cerevisiae)
Human	chr2:160451991-160454535:+	AK126410	AK126410	Homo sapiens cDNA FLJ44446 fis, clone UTERU2021380.
Human	chr2:160450646-160586767:-	AY314007	AY314006	Homo sapiens DEC-205/DCL-1 fusion protein variant V33-2 mRNA, complete cds.
Mouse	chr2:60188605-60194042:+	5730458M16	5730458M16	unclassifiable
Mouse	chr2:60190629-60223194:-	0610038N09	1110055L24	18-day embryo whole body cDNA, RIKEN full-length enriched library, clone:1110055L24 product:hypothetical C-type lectin domain containing protein, full insert sequence
Human	chr2:162098189-162667300:+	U49250	BC051190	Homo sapiens, solute carrier family 4, sodium bicarbonate transporter-like, member 10, clone IMAGE:5259366, mRNA.
Human	chr2:162104675-162105595:-	AI221719	-	-
Mouse	chr2:61742888-61752750:+	BC058399	BC052737	Mus musculus T-box brain gene 1, mRNA (cDNA clone MGC:64688 IMAGE:6825394), complete cds. CDS=256..2301
Mouse	chr2:61749504-61750906:-	4833438B03	4833438B03	unclassifiable
Human	chr2:166763548-166983801:+	BC051759	BC051759	Homo sapiens cDNA clone IMAGE:5582690, partial cds.
Human	chr2:166880316-167058004:-	X82835	X82835	H.sapiens mRNA for voltage-activated sodium channel.
Mouse	chr2:66379516-66486108:+	A330052F22	A330052F22	unclassifiable
Mouse	chr2:66418716-66426141:-	CD804204	D130016G11	hypothetical protein
Human	chr2:170480829-170494082:+	BC020818	CR609037	full-length cDNA clone CS0DI033YM01 of Placenta Cot 25-normalized of Homo sapiens (human).
Human	chr2:170492098-170506948:-	AF161482	AF161482	Homo sapiens HSPC133 mRNA, complete cds.
Mouse	chr2:69800213-69810529:+	BC003820	6720477H13	Sjogren syndrome antigen B
Mouse	chr2:69809831-69824251:-	2810410A08	2810410A08	hypothetical SAM (and some other nucleotide) binding motif containing protein
Human	chr2:171610489-171649152:+	AL117430	AL117430	Homo sapiens mRNA; cDNA DKFZp434D156 (from clone DKFZp434D156); partial cds.
Human	chr2:171609565-171611408:-	BC020676	BC020676	Homo sapiens, clone IMAGE:4732187, mRNA.
Mouse	chr2:70600120-70630393:+	5730520M13	9030002I16	golgi reassembly stacking protein 2
Mouse	chr2:70599787-70600930:-	A530079D03	A530079D03	hypothetical Arginine-rich region containing protein
Human	chr2:176826847-176863333:+	BC047605	BC005124	Homo sapiens homeo box D3, mRNA (cDNA clone MGC:10470 IMAGE:3936607), complete cds.
Human	chr2:176812595-176827333:-	BC034000	BC034000	Homo sapiens, clone IMAGE:4128549, mRNA.
Mouse	chr2:74606095-74643201:+	A330050G02	A330050G02	homeo box D3
Mouse	chr2:74597173-74610296:-	E330022G09	1700109F18	RIKEN cDNA 1700109F18 gene
Human	chr2:179213061-179323973:+	AK093733	AK093733	Homo sapiens cDNA FLJ36414 fis, clone THYMU2010848.
Human	chr2:179018732-179497656:-	X90568	AK129531	Homo sapiens cDNA FLJ26020 fis, clone PCD01884, highly similar to Homo sapiens titin (TTN), transcript variant N2-B.
Mouse	chr2:76647657-76648476:+	CD559841	-	-
Mouse	chr2:76647259-76648289:-	CB845268	-	-
Human	chr2:182147221-182229328:+	BC080190	X16983	Human mRNA for integrin alpha-4 subunit.
Human	chr2:182226905-182347339:-	AK129976	AY357073	Homo sapiens ceramide kinase-like protein (CERKL) mRNA, complete cds.
Mouse	chr2:79223109-79227182:+	F830012M05	A430066O12	unclassifiable
Mouse	chr2:79226550-79323056:-	K630012K02	A930029I05	unclassifiable
Human	chr2:198143664-198165359:+	BC022453	BC022453	Homo sapiens, hypothetical protein FLJ13448, clone MGC:26023 IMAGE:4796819, mRNA, complete cds.
Human	chr2:198141815-198144125:-	AK096309	AK096309	Homo sapiens cDNA FLJ38990 fis, clone NT2RI2008455.
Mouse	chr1:55435843-55455954:+	1700030I21	1500041J02	hypothetical protein
Mouse	chr1:55434436-55436229:-	E330011M16	E330011M16	hypothetical protein
Human	chr2:198190227-198243929:+	CN264788	AF250319	Homo sapiens CGI95-iso mRNA, complete cds.
Human	chr2:198176303-198190522:-	BX403622	BX647145	Homo sapiens mRNA; cDNA DKFZp779D1616 (from clone DKFZp779D1616).
Mouse	chr1:55470789-55473979:+	BQ958390	E430004I19	heat shock 10 kDa protein 1 (chaperonin 10)
Mouse	chr1:55460483-55470900:-	I0C0006H15	I920062F22	heat shock protein 1 (chaperonin)
Human	chr2:200148595-200151652:+	AK096649	AK096649	Homo sapiens cDNA FLJ39330 fis, clone OCBBF2016405.
Human	chr2:199959729-200150707:-	AB028957	AB028957	Homo sapiens mRNA for KIAA1034 protein, partial cds.
Mouse	chr1:57357921-57440028:+	B530002L08	B530002L08	unclassifiable
Mouse	chr1:57180801-57364906:-	AK129270	AK129270	Mus musculus mRNA for mKIAA1034 protein. CDS=1292..3475
Human	chr2:200645546-200699247:+	AK026208	AK026208	Homo sapiens cDNA: FLJ22555 fis, clone HSI01193.
Human	chr2:200621246-200646368:-	BI825654	AK095272	Homo sapiens cDNA FLJ37953 fis, clone CTONG2009268, weakly similar to Rattus norvegicus protein associating with small stress protein PASS1 (Pass1) mRNA.
Mouse	chr1:57795318-57806935:+	9430016H08	C130036C08	RIKEN cDNA 9430016H08 gene
Mouse	chr1:57777227-57795664:-	BC023868	BC023868	Mus musculus, Similar to hypothetical protein FLJ37953, clone IMAGE:5354246, mRNA.

Human	chr2:203602443-203674990:+	BC021999	BC021999	Homo sapiens, clone IMAGE:4823540, mRNA.
Human	chr2:203569893-203705027:-	AL548978	AK056092	Homo sapiens cDNA FLJ31530 fis, clone NT2RI2000578, highly similar to Homo sapiens YTM1 mRNA.
Mouse	chr1:60501841-60554633:+	A930038C22	A930038C22	CALCIUM-RESPONSE FACTOR CARF homolog [Mus musculus]
Mouse	chr1:60480462-60502239:-	AB041608	4933402C23	WD repeat domain 12
Human	chr2:204018500-204122402:+	X95632	X95632	H.sapiens mRNA for Arg protein tyrosine kinase-binding protein.
Human	chr2:204018688-204019455:-	AI567466	-	-
Mouse	chr1:60813144-60885024:+	BC056345	BC056345	Mus musculus abl-interactor 2, mRNA (cDNA clone MGC:73916 IMAGE:5705048), complete cds. CDS=6..1346
Mouse	chr1:60812416-60813712:-	M130047K10	-	-
Human	chr2:210692797-210754586:+	AK093026	AK093026	Homo sapiens cDNA FLJ35707 fis, clone SPLEN2020427.
Human	chr2:210711651-210861579:-	AK074441	AK074441	Homo sapiens cDNA FLJ23861 fis, clone LNG08328.
Mouse	chr1:67096684-67193315:+	5730518J08	5730518J08	RIBULOSE-5-PHOSPHATE-EPIMERASE (FRAGMENT) homolog [Homo sapiens]
Mouse	chr1:67115157-67213509:-	C430010P07	I0C0045H18	hypothetical protein
Human	chr2:219109343-219140518:+	AI859398	X12901	Human mRNA for villin.
Human	chr2:219140479-219258588:-	BX538024	BX538024	Homo sapiens mRNA; cDNA DKFZp686C086 (from clone DKFZp686C086); complete cds.
Mouse	chr1:74867804-74894043:+	I920157F22	0610009G14	villin
Mouse	chr1:74893993-75002769:-	5430409E09	4932415L06	hypothetical Ubiquitin carboxyl-terminal hydrolase family 2 containing protein
Human	chr2:219297993-219327409:+	AK023083	BC006355	Homo sapiens phospholipase C, delta 4, mRNA (cDNA clone MGC:12837 IMAGE:4124286), complete cds.
Human	chr2:219326420-219349883:-	BC042182	D87073	Human mRNA for KIAA0236 gene, complete cds.
Mouse	chr1:75001371-75026277:+	D130011E18	D130011E18	phospholipase C, delta 4
Mouse	chr1:75023611-75046700:-	4831430K24	4831430K24	zinc finger protein 142
Human	chr2:219866452-219875706:+	BX397761	AK074983	Homo sapiens cDNA FLJ90502 fis, clone NT2RP3004075.
Human	chr2:219862124-219868392:-	AF151815	AF151815	Homo sapiens CGI-57 protein mRNA, complete cds.
Mouse	chr1:75585126-75592553:+	F630042M24	9430022K03	hypothetical protein
Mouse	chr1:75578790-75586949:-	BC028815	4732490H22	similar to CGI-57 PROTEIN [Homo sapiens]
Human	chr2:219919989-219926896:+	BC028334	BC028334	Homo sapiens, clone IMAGE:4510516, mRNA.
Human	chr2:219926827-219935705:-	BC028370	BC028370	Homo sapiens, clone IMAGE:4827818, mRNA.
Mouse	chr1:75635958-75643215:+	BC057896	1300008P06	hypothetical Ankyrin repeat structure containing protein
Mouse	chr1:75637784-75654205:-	4932405E23	4932405E23	hypothetical Glycoside hydrolase family 35 containing protein
Human	chr2:219935682-219940564:+	BC019915	AF060798	Homo sapiens myristilated and palmitylated serine-threonine kinase MPSK (MPSK1) mRNA, complete cds.
Human	chr2:219939938-219968468:-	AK054731	AK054731	Homo sapiens cDNA FLJ30169 fis, clone BRACE2000864, highly similar to TUBULIN ALPHA-4 CHAIN.
Mouse	chr1:75654221-75659003:+	5330407I02	5330407I02	serine/threonine kinase 16
Mouse	chr1:75657615-75663257:-	K530012D09	6530406I01	tubulin alpha 4
Human	chr2:220233890-220240822:+	BC068567	BC068567	Homo sapiens cDNA clone IMAGE:5275321, partial cds.
Human	chr2:220228997-220234126:-	AB095813	AB095813	Homo sapiens ChPF mRNA for chondroitin polymerizing factor, complete cds.
Mouse	chr1:75924614-75931199:+	K430347I15	K430347I15	hypothetical Proline-rich region profile/Leucine-rich region profile/Arginine-rich region profile containing protein
Mouse	chr1:75920124-75951977:-	BC057050	4931417C03	hypothetical Immunoglobulin and major histocompatibility complex domain containing protein
Human	chr2:241808989-241843057:+	BC051689	BC051689	Homo sapiens protein phosphatase 1, regulatory subunit 7, mRNA (cDNA clone IMAGE:5180137), with apparent retained intron.
Human	chr2:241765504-241809669:-	CR749231	CR749231	Homo sapiens mRNA; cDNA DKFZp686P2031 (from clone DKFZp686P2031).
Mouse	chr1:93279627-93306561:+	6330525M19	AF222867	Mus musculus protein phosphatase-1 regulatory subunit 7 (Ppp1r7) mRNA, complete cds. CDS=16..1101
Mouse	chr1:93246218-93280255:-	F830031M04	D030051B04	PAS domain containing serine/threonine kinase
Human	chr2:242015648-242154251:+	AB018336	AB018336	Homo sapiens mRNA for KIAA0793 protein, complete cds.
Human	chr2:242148933-242152692:-	AK124871	AK124871	Homo sapiens cDNA FLJ42881 fis, clone BRHIP3004968.
Mouse	chr1:93447878-93557778:+	D030026M03	D030026M03	weakly similar to CDEP [Homo sapiens]
Mouse	chr1:93555550-93594458:-	G630071O21	G370001E18	Serine/threonine protein kinase 25 (EC 2.7.1.37) (Sterile 20/oxidant stress-response kinase 1) (Ste20/oxidant stress response kinase-1) (SOK-1) (Ste20-like kinase)
Human	chr2:242015648-242154251:+	AB018336	AB018336	Homo sapiens mRNA for KIAA0793 protein, complete cds.
Human	chr2:242152793-242168703:-	T03735	BC007852	Homo sapiens serine/threonine kinase 25 (STE20 homolog, yeast), mRNA (cDNA clone MGC:14329 IMAGE:4298239), complete cds.
Mouse	chr1:93447878-93557778:+	D030026M03	D030026M03	weakly similar to CDEP [Homo sapiens]
Mouse	chr1:93555550-93594458:-	G630071O21	G370001E18	Serine/threonine protein kinase 25 (EC 2.7.1.37) (Sterile 20/oxidant stress-response kinase 1) (Ste20/oxidant stress response kinase-1) (SOK-1) (Ste20-like kinase)
Human	chr3:3143621-3167562:+	BC012537	BC012537	Homo sapiens, Similar to CGI-47 protein, clone MGC:13334 IMAGE:4097092, mRNA, complete cds.
Human	chr3:3164304-3164787:-	BM873557	-	-
Mouse	chr6:107162488-107175983:+	I830083H20	I830083H20	tRNA nucleotidyl transferase, CCA-adding, 1
Mouse	chr6:107171753-107193596:-	BC046967	C820017J03	PIL

Human	chr3:9622712-9853045:+	BI768875	AK127786	Homo sapiens cDNA FLJ45887 fis, clone OCBBF3021502.
Human	chr3:9796648-9809564:-	BC009240	BC013433	Homo sapiens, transcriptional adaptor 3 (ADA3, yeast homolog)-like (PCAF histone acetylase complex), clone MGC:16840 IMAGE:3897746, mRNA, complete cds.
Mouse	chr6:113899140-113936125:+	L830008P19	BC006830	Mus musculus RIKEN cDNA 4833441J24 gene, mRNA (cDNA clone MGC:11856 IMAGE:3597662), complete cds. CDS=590..1390
Mouse	chr6:113886413-113899420:-	9130008B10	9130008B10	ADA3-LIKE PROTEIN (TRANSCRIPTIONAL ADAPTOR 3 (ADA3, YEAST HOMOLOG)-LIKE) (PCAF HISTONE ACETYLASE COMPLEX) homolog [Homo sapiens]
Human	chr3:9766628-9804898:+	U88620	U88620	Human 8-hydroxyguanine glycosylase (hMMH) mRNA, complete cds.
Human	chr3:9774025-9786661:-	CR607002	L41816	Homo sapiens cam kinase I mRNA, complete cds.
Mouse	chr6:113848440-113856719:+	2010011J20	I920067F05	8-oxoguanine DNA-glycosylase 1
Mouse	chr6:113855775-113865533:-	BC042494	BC014825	Mus musculus calcium/calmodulin-dependent protein kinase I, mRNA (cDNA clone MGC:18933 IMAGE:3969343), complete cds. CDS=120..1244
Human	chr3:9933782-9950314:+	BC006411	AK092907	Homo sapiens cDNA FLJ35588 fis, clone SPLEN2007388.
Human	chr3:9948720-9950348:-	AK075038	AK075038	Homo sapiens cDNA FLJ90557 fis, clone OVARC1000995.
Mouse	chr6:113991966-114003629:+	AF458066	BC004759	Mus musculus interleukin 17 receptor C, mRNA (cDNA clone MGC:6973 IMAGE:3154616), complete cds. CDS=177..1880
Mouse	chr6:113999944-114003666:-	9330110N14	9330110N14	unclassifiable
Human	chr3:9964044-9971477:+	BQ000843	-	-
Human	chr3:9962226-9969099:-	AX746722	BC040508	Homo sapiens, clone IMAGE:5300209, mRNA.
Mouse	chr6:114015233-114117633:+	CD803676	D430030I24	Fanconi anemia, complementation group D2
Mouse	chr6:114013943-114022348:-	6330505P20	9330151C08	hypothetical Leucine-rich region profile containing protein
Human	chr3:11289016-11574663:+	AL122075	AK075221	Homo sapiens cDNA FLJ90740 fis, clone PLACE1011045, highly similar to Homo sapiens E1-like protein mRNA.
Human	chr3:11572539-11741453:-	D50911	AK130542	Homo sapiens cDNA FLJ27032 fis, clone SLV07789.
Mouse	chr6:115032050-115249936:+	I920083E09	I920083E09	autophagy 7-like (S. cerevisiae)
Mouse	chr6:115249209-115357879:-	CB575393	-	-
Human	chr3:12573522-12602330:+	AK124449	BC015715	Homo sapiens, clone MGC:16931 IMAGE:3916199, mRNA, complete cds.
Human	chr3:12599593-12680725:-	BC018119	BC018119	Homo sapiens v-raf-1 murine leukemia viral oncogene homolog 1, mRNA (cDNA clone MGC:9026 IMAGE:3904404), complete cds.
Mouse	chr6:115991977-116011760:+	C630033J23	6030446P03	makorin, ring finger protein, 2
Mouse	chr6:116008517-116066714:-	0710001O06	D030050G20	v-raf-1 leukemia viral oncogene 1
Human	chr3:14964098-15070111:+	BC051670	L27586	Human TR4 orphan receptor mRNA, complete cds.
Human	chr3:15058750-15081846:-	AK096625	AK024433	Homo sapiens mRNA for FLJ00023 protein, partial cds.
Mouse	chr6:92441897-92524283:+	9630033I20	F830210G23	nuclear receptor subfamily 2, group C, member 2
Mouse	chr6:92517815-92519101:-	6330436D07	6330436D07	unclassifiable
Human	chr3:15062062-15065790:+	AK091226	AK091226	Homo sapiens cDNA FLJ33907 fis, clone CTONG2008506.
Human	chr3:15058750-15081846:-	AK024433	AK024433	Homo sapiens mRNA for FLJ00023 protein, partial cds.
Mouse	chr6:92441897-92524283:+	1700102N12	F830210G23	nuclear receptor subfamily 2, group C, member 2
Mouse	chr6:92522255-92534022:-	2810429N01	BC022953	Mus musculus mitochondrial ribosomal protein S25, mRNA (cDNA clone MGC:30559 IMAGE:5126320), complete cds. CDS=38..553
Human	chr3:15067743-15068525:+	AV655948	-	-
Human	chr3:15058750-15081846:-	BC003590	AK024433	Homo sapiens mRNA for FLJ00023 protein, partial cds.
Mouse	chr6:92441897-92524283:+	1700102N12	F830210G23	nuclear receptor subfamily 2, group C, member 2
Mouse	chr6:92522255-92534022:-	2810429N01	BC022953	Mus musculus mitochondrial ribosomal protein S25, mRNA (cDNA clone MGC:30559 IMAGE:5126320), complete cds. CDS=38..553
Human	chr3:15443548-15466844:+	BC029294	BC041329	Homo sapiens ELL associated factor 1, mRNA (cDNA clone MGC:41764 IMAGE:5284115), complete cds.
Human	chr3:15397786-15480256:-	CR612029	AK130406	Homo sapiens cDNA FLJ26896 fis, clone RCT00464.
Mouse	chr14:27529526-27544987:+	4933403C17	A930038B11	EAF1 homolog [Homo sapiens]
Mouse	chr14:27508697-27530167:-	1600013P15	5730403G09	Methyltransferase-like protein 4 homolog (EC 2.1.1.-) homolog [Mus musculus]
Human	chr3:15618248-15662333:+	T53932	U03274	Homo sapiens biotinidase mRNA, complete cds.
Human	chr3:15577215-15618342:-	CN356362	AJ131753	Homo sapiens mRNA for 2-hydroxyphytanoyl-CoA lyase.
Mouse	chr14:27675823-27703374:+	1600020N20	6820408J04	biotinidase
Mouse	chr14:27631488-27676081:-	L930231I10	4932442H07	2-hydroxyphytanoyl-CoA lyase (EC 4.1.-.-) (2-HPCL)
Human	chr3:23822407-23907827:+	CR624061	BC009139	Homo sapiens, ubiquitin-conjugating enzyme E2E 1 (homologous to yeast UBC4/5), clone MGC:9268 IMAGE:3853408, mRNA, complete cds.
Human	chr3:23905879-23907804:-	R19077	-	-
Mouse	chr14:14954757-15004166:-	BC003781	G630012E05	ubiquitin-conjugating enzyme E2E 1, UBC4/5 homolog (yeast)
Mouse	chr14:14943086-14956038:+	G370001C03	G370001C03	NFKB inhibitor interacting Ras-like protein 1
Human	chr3:23933040-23941126:+	BI544251	BC081565	Homo sapiens cDNA clone MGC:88603 IMAGE:5458912, complete cds.
Human	chr3:23908556-23963057:-	CR600782	AK027749	Homo sapiens cDNA FLJ14843 fis, clone PLACE1000040, weakly similar to TRANSFORMING PROTEIN P21/K-RAS 2B.
Mouse	chr14:14939773-14943341:-	1700030F15	I920056F05	ribosomal protein L15
Mouse	chr14:14943086-14956038:+	4632424N06	G370001C03	NFKB inhibitor interacting Ras-like protein 1

Human	chr3:25443024-25614427:+	BC060794	BC030234	Homo sapiens, retinoic acid receptor, beta, clone IMAGE:5229160, mRNA.
Human	chr3:25612451-25613994:-	F11864	-	-
Mouse	chr14:13077623-13838942:-	S56660	X56569	Mouse RAR-beta mRNA for retinoic acid receptor-beta 1 isoform. CDS=139..360
Mouse	chr14:13012452-13079426:+	D330024O17	BC041106	Mus musculus, topoisomerase (DNA) II beta, clone IMAGE:5368508, mRNA.
Human	chr3:25443024-25614427:+	BC060794	BC030234	Homo sapiens, retinoic acid receptor, beta, clone IMAGE:5229160, mRNA.
Human	chr3:25614400-25681434:-	BE467111	X68060	H.sapiens topIIb mRNA for topoisomerase IIb.
Mouse	chr14:13077623-13838942:-	S56660	X56569	Mouse RAR-beta mRNA for retinoic acid receptor-beta 1 isoform. CDS=139..360
Mouse	chr14:13012452-13079426:+	D330024O17	BC041106	Mus musculus, topoisomerase (DNA) II beta, clone IMAGE:5368508, mRNA.
Human	chr3:25799568-25811037:+	BQ057854	AK000611	Homo sapiens cDNA FLJ20604 fis, clone KAT06449.
Human	chr3:25735439-25799999:-	CR611129	CR611129	full-length cDNA clone CS0DL004YG23 of B cells (Ramos cell line) Cot 25-normalized of Homo sapiens (human).
Mouse	chr14:12885727-12897013:-	D030054J07	D030054J07	weakly similar to AGR_C_2030P [Agrobacterium tumefaciens str. C58]
Mouse	chr14:12896346-12959097:+	I920090E18	3002002K11	peptide N-glycanase homolog (S.cerevisiae)
Human	chr3:28258079-28336271:+	CR749370	CR749370	Homo sapiens mRNA; cDNA DKFZp779D0833 (from clone DKFZp779D0833).
Human	chr3:28331498-28365579:-	AW629502	BX648471	Homo sapiens mRNA; cDNA DKFZp686E14155 (from clone DKFZp686E14155).
Mouse	chr9:118035879-118120093:-	2010110K16	2010110K16	hypothetical protein
Mouse	chr9:118012431-118041690:+	BC054402	9630036C09	5-azacytidine induced gene 2
Human	chr3:33405023-33405511:+	BE670150	-	-
Human	chr3:33404400-33456874:-	CR749798	BC047235	Homo sapiens, similar to upstream binding protein 1 (LBP-1a), clone MGC:57378 IMAGE:5262788, mRNA, complete cds.
Mouse	chr9:113978676-114060965:-	C230086C13	G430047M01	LEUCINE-RICH REPEATS CONTAINING F-BOX PROTEIN FBL3 homolog [Homo sapiens]
Mouse	chr9:113946904-113992337:+	BC034749	BC034749	Mus musculus upstream binding protein 1, mRNA (cDNA clone IMAGE:4946022), with apparent retained intron.
Human	chr3:38055700-38140044:+	AB020522	AB020522	Homo sapiens DLEC1 (deleted in lung and esophageal cancer 1; DLEC1 alias DLC1) mRNA, complete cds.
Human	chr3:38139108-38153703:-	X51460	CR626439	full-length cDNA clone CS0DI077YD15 of Placenta Cot 25-normalized of Homo sapiens (human).
Mouse	chr9:119072781-119118549:+	F430108N02	5730593F21	Similar to deleted in lung and ESOPHAGEAL cancer 1
Mouse	chr9:119118326-119127396:-	AY273812	AY273812	Mus musculus 3-ketoacyl-CoA thiolase B mRNA, complete cds. CDS=98..1372
Human	chr3:39068483-39113166:+	D17163	AB040882	Homo sapiens mRNA for KIAA1449 protein, partial cds.
Human	chr3:39113099-39124858:-	AK027096	AK124755	Homo sapiens cDNA FLJ42765 fis, clone BRAWH3002853, highly similar to Homo sapiens mRNA for golgi perepheral membrane protein p65.
Mouse	chr9:119880872-119912581:+	BC048155	5930436P16	hypothetical Trp-Asp (WD) repeats profile/Trp-Asp (WD) repeats circular profile/G-protein beta WD-40 repeats containing protein
Mouse	chr9:119909984-119923559:-	5430411C10	BC012251	Mus musculus golgi reassembly stacking protein 1, mRNA (cDNA clone MGC:19028 IMAGE:4166619), complete cds. CDS=42..1382
Human	chr3:42598342-42611610:+	BX114027	AF201950	Homo sapiens kiaa-iso protein mRNA, complete cds.
Human	chr3:42564463-42617706:-	AK074576	CR595143	full-length cDNA clone CS0DI007YF16 of Placenta Cot 25-normalized of Homo sapiens (human).
Mouse	chr9:121709381-121711185:+	E030046P19	E030046P19	unclassifiable
Mouse	chr9:121683048-121710059:-	5930407I15	4932412K21	similar to VESICLE TRAFFICKING PROTEIN [Homo sapiens]
Human	chr3:44878400-44882164:+	AL834253	AL834253	Homo sapiens mRNA; cDNA DKFZp434J174 (from clone DKFZp434J174).
Human	chr3:44877867-44878625:-	CF126629	-	-
Mouse	chr9:123029034-123075977:+	BC049583	9530008N10	similar to DORSAL PROSTATE PROTEIN-GLUTAMINE GAMMA-GLUTAMYLTRANSFERASE (EC 2.3.2.13) (DORSAL PROSTATE TRANSGLUTAMINASE) (DORSAL PROTEIN 1) (DP1) [Rattus norvegicus]
Mouse	chr9:123026699-123029282:-	4933429E06	4933429E06	hypothetical protein
Human	chr3:49019778-49028390:+	AL833910	AF099100	Homo sapiens WD-repeat protein 6 (WDR6) mRNA, complete cds.
Human	chr3:49027925-49034730:-	BC032440	AK093204	Homo sapiens cDNA FLJ35885 fis, clone TESTI2009018.
Mouse	chr9:108579411-108586050:-	BC054367	AB041854	Mus musculus wdp122 mRNA for WD-repeat protein p122, complete cds. CDS=147..3521
Mouse	chr9:108574706-108579989:+	BC013483	6330580J24	hypothetical Anticodon-binding domain of a subclass of class I aminoacyl-tRNA synthetases structure containing protein
Human	chr3:49701553-49733966:+	AL832138	AL832034	Homo sapiens mRNA; cDNA DKFZp451P244 (from clone DKFZp451P244).
Human	chr3:49729274-49736425:-	AB058754	AB058754	Homo sapiens mRNA for KIAA1851 protein, partial cds.
Mouse	chr9:108073802-108105894:-	BC057082	9830164A22	ring finger protein 123
Mouse	chr9:108071510-108077989:+	AK129456	AK129456	Mus musculus mRNA for mKIAA1851 protein. CDS=1..4019
Human	chr3:49701553-49733966:+	AL832138	AL832034	Homo sapiens mRNA; cDNA DKFZp451P244 (from clone DKFZp451P244).
Human	chr3:49729274-49736425:-	AB058754	AB058754	Homo sapiens mRNA for KIAA1851 protein, partial cds.
Mouse	chr9:108075914-108078054:-	BM934659	-	-
Mouse	chr9:108071510-108077989:+	E430002N15	AK129456	Mus musculus mRNA for mKIAA1851 protein. CDS=1..4019
Human	chr3:50291522-50300551:+	AK095927	BC028000	Homo sapiens, hypothetical gene LOC132228, clone MGC:40187 IMAGE:5197901, mRNA, complete cds.
Human	chr3:50294093-50311903:-	Y12395	Y12395	Homo sapiens mRNA for inteferon related IFRD2 (PC4-B) protein (IFRD2 gene), SM15 homologue.
Mouse	chr9:107612562-107617010:-	K630027K09	-	-
Mouse	chr9:107607577-107612989:+	F830033L02	G730042B14	interferon-related developmental regulator 2

Human	chr3:51403751-51410379:+	AL831838	AL831838	Homo sapiens mRNA; cDNA DKFZp547N2215 (from clone DKFZp547N2215).
Human	chr3:51408338-51514667:-	AB018343	AB018343	Homo sapiens mRNA for KIAA0800 protein, complete cds.
Mouse	chr9:106858111-106911914:-	BC025849	2700052B17	arginine-rich, mutated in early stage tumors
Mouse	chr9:106841813-106900927:+	D730020L12	AK122372	Mus musculus mRNA for mKIAA0800 protein. CDS=79..4625
Human	chr3:51964437-51967381:+	BC026357	BC026357	Homo sapiens, G protein-coupled receptor 62, clone MGC:26943 IMAGE:4812283, mRNA, complete cds.
Human	chr3:51966510-51977459:-	AF176330	AK023993	Homo sapiens cDNA FLJ13931 fis, clone Y79AA1000752, moderately similar to PUTATIVE HETEROGENEOUS NUCLEAR RIBONUCLEOPROTEIN X.
Mouse	chr9:106481783-106483083:-	4933402E03	1700013N11	unclassifiable
Mouse	chr9:106471064-106482174:+	AF176328	C130093L04	poly(rC) binding protein 4
Human	chr3:52296145-52302533:+	AF448855	AF448855	Homo sapiens glycerate kinase 1 (GLYCTK1) mRNA, complete cds.
Human	chr3:52297636-52308123:-	AI936688	-	-
Mouse	chr9:106093752-106099037:-	BC025935	BC025935	Mus musculus RIKEN cDNA 6230410P16 gene, mRNA (cDNA clone MGC:37792 IMAGE:5097665), complete cds. CDS=235..1806
Mouse	chr9:106089597-106100650:+	D030055H07	D030055H07	unclassifiable
Human	chr3:52504396-52533555:+	AK093774	D87433	Homo sapiens mRNA for KIAA0246 protein, partial cds.
Human	chr3:52533425-52544110:-	AF131781	AK023995	Homo sapiens cDNA FLJ13933 fis, clone Y79AA1000782, weakly similar to CYTOSOLIC PURINE 5'-NUCLEOTIDASE (EC 3.1.3.5).
Mouse	chr14:27174101-27203735:-	BC022136	AF290914	Mus musculus stabilin-1 mRNA, complete cds. CDS=81..7796
Mouse	chr14:27166272-27174215:+	BC011230	BC011230	Mus musculus RIKEN cDNA 2510015F01 gene, mRNA (cDNA clone MGC:19108 IMAGE:4207917), complete cds. CDS=156..1328
Human	chr3:52714011-52717727:+	BG724036	AF092138	Homo sapiens HSPC033 mRNA, complete cds.
Human	chr3:52703209-52715139:-	BI559798	CR749821	Homo sapiens mRNA; cDNA DKFZp781O20198 (from clone DKFZp781O20198).
Mouse	chr14:27034706-27037382:-	BC034270	BC034270	Mus musculus, clone IMAGE:4208827, mRNA.
Mouse	chr14:27036600-27047657:+	5430414N14	5430414N14	AD-017 PROTEIN (GLYCOSYLTRANSFERASE) (CDNA FLJ14611 FIS, CLONE NT2RP1000988) homolog [Homo sapiens]
Human	chr3:53503723-53822800:+	BU679327	M76558	Human neuronal DHP-sensitive, voltage-dependent, calcium channel alpha-1D subunit mRNA, complete cds.
Human	chr3:53822155-53822594:-	BF436153	-	-
Mouse	chr14:26076267-26528197:-	BC058783	AJ437292	Mus musculus mRNA for L-type voltage-gated calcium channel Cav1.3(1b) subunit (cacna1d gene). CDS=56..6556
Mouse	chr14:26045129-26076807:+	BQ959159	C430028B04	choline dehydrogenase
Human	chr3:56566231-56630877:+	AL833326	AL833326	Homo sapiens mRNA; cDNA DKFZp686C0433 (from clone DKFZp686C0433).
Human	chr3:56629197-56692305:-	AB029028	BC070096	Homo sapiens cDNA clone IMAGE:5294992, partial cds.
Mouse	chr14:23510154-23548446:-	E030027N17	K230049C06	hypothetical protein
Mouse	chr14:23467347-23535486:+	BC064458	4933409E02	similar to RETINOBLASTOMA-ASSOCIATED PROTEIN RAP140 [Homo sapiens]
Human	chr3:57517041-57615744:+	AW183704	BX647169	Homo sapiens mRNA; cDNA DKFZp686D19223 (from clone DKFZp686D19223).
Human	chr3:57586221-57653844:-	BC040291	AK074156	Homo sapiens mRNA for FLJ00229 protein.
Mouse	chr14:22648367-22658145:-	4930570N19	4930570N19	hypothetical protein
Mouse	chr14:22594286-22652059:+	D030012D10	7420700D11	weakly similar to 1700027J05RIK PROTEIN [Mus musculus]
Human	chr3:64522062-64551202:+	AK125532	AK125532	Homo sapiens cDNA FLJ43544 fis, clone PROST2009388.
Human	chr3:64476367-64648714:-	AF488803	AF488803	Homo sapiens a disintegrin-like and metalloprotease with thrombospondin type 1 motifs 9B (ADAMTS9) mRNA, complete cds.
Mouse	chr6:93172698-93203394:+	A730049H05	A730049H05	hypothetical protein
Mouse	chr6:93128915-93257661:-	8430403M15	8430403M15	weakly similar to ADAMTS-9 PRECURSOR (EC 3.4.24.-) (A DISINTEGRIN AND METALLOPROTEINASE WITH THROMBOSPONDIN MOTIFS 9) (ADAM-TS 9) (ADAM-TS9) [Homo sapiens]
Human	chr3:66376317-66513135:+	BC012852	BC012852	Homo sapiens, Similar to RIKEN cDNA 4930433D19 gene, clone MGC:9811 IMAGE:3860705, mRNA, complete cds.
Human	chr3:66511907-66634125:-	AF381545	BC071561	Homo sapiens leucine-rich repeats and immunoglobulin-like domains 1, mRNA (cDNA clone MGC:87003 IMAGE:30340674), complete cds.
Mouse	chr6:94865286-94969644:+	BC019170	BC019170	Mus musculus, Similar to RIKEN cDNA 4930433D19 gene, clone IMAGE:5003084, mRNA.
Mouse	chr6:94969501-95064906:-	D78572	D78572	Mus musculus mRNA for membrane glycoprotein, complete cds. CDS=454..3729
Human	chr3:99075350-99146520:+	BX647079	AK130907	Homo sapiens cDNA FLJ27397 fis, clone WMC02447.
Human	chr3:99145710-99173978:-	CR627479	AY302110	Homo sapiens MDIG (MDIG) mRNA, complete cds.
Mouse	chr16:59747898-59812368:-	9530036A08	BC043118	Mus musculus cDNA sequence BC043118, mRNA (cDNA clone MGC:58045 IMAGE:6408859), complete cds. CDS=402..3722
Mouse	chr16:59728898-59750321:+	BC058242	1810047J07	hypothetical RmlC-like structure containing protein
Human	chr3:99733568-99734650:+	BC069437	BC069437	Homo sapiens G protein-coupled receptor 15, mRNA (cDNA clone MGC:97025 IMAGE:7262234), complete cds.
Human	chr3:99733610-99757771:-	CD520440	-	-
Mouse	chr16:58975816-58978001:-	I6C0040A17	-	-
Mouse	chr16:58929558-58978241:+	A930103L13	D16333	Mus musculus mRNA for coproporphyrinogen oxidase, complete cds. CDS=402..1466
Human	chr3:101602727-101657864:+	BC040562	BC040562	Homo sapiens, clone IMAGE:5266758, mRNA.
Human	chr3:101564965-101602952:-	BC065555	BC065555	Homo sapiens translocase of outer mitochondrial membrane 70 homolog A (yeast), mRNA (cDNA clone MGC:75021 IMAGE:6067022), complete cds.
Mouse	chr16:57296119-57297660:-	G830025C03	A730057D15	unclassifiable
Mouse	chr16:57297457-57332907:+	M130016B12	AK122356	Mus musculus mRNA for mKIAA0719 protein. CDS=37..1917

Human	chr3:102877964-102880745:+	BC003066	BC016930	Homo sapiens, clone IMAGE:4402152, mRNA, partial cds.
Human	chr3:102780252-102878665:-	BC028062	U69274	Human zinc finger protein mRNA, complete cds.
Mouse	chr16:56140530-56141855:-	2310061J03	2310061J03	unclassifiable
Mouse	chr16:56133537-56176177:+	F730230N09	9230110G02	hypothetical ATP/GTP-binding site motif A (P-loop)/Zinc finger, C2H2 type containing protein
Human	chr3:114203771-114207107:+	BX448534	-	-
Human	chr3:114203976-114221270:-	CR749341	CR749341	Homo sapiens mRNA; cDNA DKFZp686B18261 (from clone DKFZp686B18261).
Mouse	chr16:44694555-44704152:-	0610037H22	0610037H22	hypothetical P-loop containing nucleotide triphosphate hydrolases structure containing protein
Mouse	chr16:44682092-44699206:+	D430011P07	G730009P16	Similar to hypothetical gene supported by AK024325, AL117573
Human	chr3:122950822-122952722:+	BC036879	BC036879	Homo sapiens, clone IMAGE:5243718, mRNA.
Human	chr3:122864736-122951292:-	X75304	X75304	H.sapiens giantin mRNA.
Mouse	chr16:36641040-36726890:-	4933413H08	BC056626	Mus musculus ELL associated factor 2, mRNA (cDNA clone MGC:67901 IMAGE:5008372), complete cds. CDS=484..882
Mouse	chr16:36726642-36741047:+	F630226B12	F630225N06	hypothetical protein
Human	chr3:123585713-123612040:+	AF250321	AF250321	Homo sapiens DC16 mRNA, complete cds.
Human	chr3:123611524-123612137:-	BQ182661	-	-
Mouse	chr16:35886457-35918882:-	BC010826	BC010826	Mus musculus RIKEN cDNA 2310056P07 gene, mRNA (cDNA clone MGC:19151 IMAGE:4219531), complete cds. CDS=22..489
Mouse	chr16:35883818-35886618:+	C730037F06	C730037F06	WD repeat domain 5B
Human	chr3:123765830-123776740:+	AY225123	AK125086	Homo sapiens cDNA FLJ43096 fis, clone CTONG1000467, moderately similar to Mus musculus mRNA for Deltex3.
Human	chr3:123729447-123766213:-	AF307338	AF307338	Homo sapiens B aggressive lymphoma long isoform (BAL) mRNA, complete cds.
Mouse	chr16:35769203-35781841:-	G630007B09	G630007B09	hypothetical protein
Mouse	chr16:35781160-35815311:+	CF729070	BC010312	Mus musculus cDNA sequence BC003281, mRNA (cDNA clone IMAGE:3595501), partial cds.
Human	chr3:127596480-127638097:+	AK097402	AK097402	Homo sapiens cDNA FLJ40083 fis, clone TESTI2002351.
Human	chr3:127634341-127636110:-	AK000130	AK000130	Homo sapiens cDNA FLJ20123 fis, clone COL06041.
Mouse	chr6:90742368-90767466:-	C230069K22	C230069K22	hypothetical protein
Mouse	chr6:90708104-90745995:+	M5H1101C15	A930012H20	hypothetical protein, MGC:7160
Human	chr3:129355011-129610187:+	CR596126	CR596126	full-length cDNA clone CS0DD004YB04 of Neuroblastoma Cot 50-normalized of Homo sapiens (human).
Human	chr3:129266323-129355455:-	AK092163	AK092163	Homo sapiens cDNA FLJ34844 fis, clone NT2NE2011154, highly similar to Homo sapiens mRNA for TIP49.
Mouse	chr6:88591791-88782581:-	BC024915	4432405A17	eukaryotic elongation factor, selenocysteine-tRNA-specific
Mouse	chr6:88781729-88782555:+	G270015A21	-	-
Human	chr3:130081031-130117623:+	CR613592	BC007970	Homo sapiens acyl-Coenzyme A dehydrogenase family, member 9, mRNA (cDNA clone MGC:14452 IMAGE:4304209), complete cds.
Human	chr3:130111407-130204233:-	AK056377	AK056377	Homo sapiens cDNA FLJ31815 fis, clone NT2RI2009595.
Mouse	chr6:88006678-88015321:+	E230015B07	E230015B07	hypothetical protein
Mouse	chr6:88009889-88025143:-	1810020O05	1810020O05	unclassifiable
Human	chr3:130516671-130527763:+	BI551905	AX747004	Sequence 529 from Patent EP1308459.
Human	chr3:130516312-130518255:-	BC000426	BC000426	Homo sapiens H1 histone family, member X, mRNA (cDNA clone MGC:8350 IMAGE:2819756), complete cds.
Mouse	chr6:88315309-88341302:+	A730016L07	6230424B18	hypothetical protein
Mouse	chr6:88315056-88315927:-	BQ178483	-	-
Human	chr3:133798835-133820509:+	AA258335	AF110640	Homo sapiens orphan seven-transmembrane receptor (VSHK1) mRNA, complete cds.
Human	chr3:133759680-133924001:-	BC019607	AB082531	Homo sapiens mRNA for KIAA2000 protein.
Mouse	chr9:104007749-104048687:-	AY072938	AY072938	Mus musculus chemokine receptor CCX CKR mRNA, complete cds, alternatively spliced. CDS=793..1845
Mouse	chr9:103924057-104049410:+	BC006042	B020037L14	hypothetical Acyl-CoA dehydrogenase, C-terminal/Acyl-CoA dehydrogenase, central domain/Acyl-CoA dehydrogenase, middle and N-terminal containing protein
Human	chr3:135687273-135776557:+	BC014050	AK023738	Homo sapiens cDNA FLJ13676 fis, clone PLACE1011922, weakly similar to MYOSIN HEAVY CHAIN, NONMUSCLE TYPE B.
Human	chr3:135678933-135688256:-	AK001285	AK001285	Homo sapiens cDNA FLJ10423 fis, clone NT2RP1000259.
Mouse	chr9:102478434-102519931:-	BG800206	4921501M07	similar to CDNA FLJ31903 FIS, CLONE NT2RP7004260, WEAKLY SIMILAR TO MYOSIN HEAVY CHAIN, NONMUSCLE TYPE B [Homo sapiens]
Mouse	chr9:102519682-102527809:+	1810004D07	1810004D07	HYPOTHETICAL 8.5 KDA PROTEIN homolog [Homo sapiens]
Human	chr3:144582878-144583810:+	CR748637	-	-
Human	chr3:144466762-145050019:-	AK092932	BC035779	Homo sapiens, Similar to solute carrier family 9 (sodium/hydrogen exchanger), isoform 7, clone MGC:46316 IMAGE:5590356, mRNA, complete cds.
Mouse	chr9:94973698-94974281:-	CK386235	-	-
Mouse	chr9:94520579-95083924:+	F830027D23	F830027D23	solute carrier family 9 (sodium/hydrogen exchanger), isoform 9
Human	chr3:150330069-150377724:+	AK026357	AY033141	Homo sapiens Hermansky-Pudlak syndrome type-3 protein (HPS3) mRNA, complete cds.
Human	chr3:150362895-150422527:-	BX647327	AK095290	Homo sapiens cDNA FLJ37971 fis, clone CTONG2009958, highly similar to CERULOPLASMIN PRECURSOR (EC 1.16.3.1).
Mouse	chr3:19670167-19709538:-	F630001B08	F630307H22	Hermansky-Pudlak syndrome 3 homolog (human)
Mouse	chr3:19684592-19685613:+	A730036D06	A730036D06	unclassifiable

Human	chr3:152635066-152637163:+	AK124913	AK124913	Homo sapiens cDNA FLJ42923 fis, clone BRSSN2001275.
Human	chr3:152625870-152659195:-	AF087980	AY273815	Homo sapiens bone specific CMF608 mRNA, complete cds.
Mouse	chr3:59138593-59450513:+	6530405F15	C030020L10	NOPAR2 homolog [Homo sapiens]
Mouse	chr3:59450020-59461101:-	C130092D21	C130092D21	hypothetical Immunoglobulin and major histocompatibility complex domain/Immunoglobulin C-2 type/Immunoglobulin-like/Immunoglobulin subtype containing protein
Human	chr3:157875081-157907370:+	BX537965	BX537965	Homo sapiens mRNA; cDNA DKFZp686N0351 (from clone DKFZp686N0351); complete cds.
Human	chr3:157872353-157876166:-	BC033324	BC033324	Homo sapiens, clone IMAGE:4827091, mRNA.
Mouse	chr3:65766561-65793664:+	U55178	4632422G22	hypothetical WWE domain/Zinc finger C-x8-C-x5-C-x3-H type containing protein
Mouse	chr3:65745403-65767729:-	7530401I03	4731413C16	60S ribosomal protein L29 homolog [Mus musculus]
Human	chr3:158360412-158375382:+	BG697350	-	-
Human	chr3:158346993-158361251:-	AK122738	AK122738	Homo sapiens cDNA FLJ16257 fis, clone HLUNG2017307, highly similar to Homo sapiens cyclin L ania-6a mRNA.
Mouse	chr3:66196064-66200381:+	9030013E13	A930008H02	unclassifiable
Mouse	chr3:66184260-66196600:-	AF467251	BC007177	Mus musculus cyclin L1, mRNA (cDNA clone IMAGE:3494871), complete cds. CDS=72..608
Human	chr3:159867278-159867770:+	AA526937	-	-
Human	chr3:159846319-159873184:-	AF282626	BC005346	Homo sapiens, latexin protein, clone MGC:12439 IMAGE:3932274, mRNA, complete cds.
Mouse	chr3:67741661-67788094:+	8030465C11	BC013093	Mus musculus G elongation factor, mRNA (cDNA clone MGC:7961 IMAGE:3584730), complete cds. CDS=128..2383
Mouse	chr3:67769559-67775491:-	D88769	6430407E02	latexin
Human	chr3:171166875-171196955:+	T98627	BX648539	Homo sapiens mRNA; cDNA DKFZp779P0521 (from clone DKFZp779P0521).
Human	chr3:171139846-171167238:-	AK098335	AL833309	Homo sapiens mRNA; cDNA DKFZp313A137 (from clone DKFZp313A137).
Mouse	chr3:30558878-30587111:+	E430004A04	E430004A04	TRANSLOCATIONAL PROTEIN-1 (SIMILAR TO TRANSLOCATION PROTEIN 1) homolog [Homo sapiens]
Mouse	chr3:30558595-30559574:-	4933429H19	4933429H19	hypothetical TonB-dependent receptor protein containing protein
Human	chr3:180763385-180788954:+	BC001391	AK098691	Homo sapiens cDNA FLJ25825 fis, clone TST08011, highly similar to Homo sapiens BAF53a (BAF53a) mRNA.
Human	chr3:180782954-180805144:-	BE892901	BC032522	Homo sapiens, Similar to mitochondrial ribosomal protein L47, clone MGC:45403 IMAGE:5500168, mRNA, complete cds.
Mouse	chr3:32491713-32510184:+	BC001994	I920165I07	53 kDa BRG1-associated factor A (Actin-related protein Baf53a)
Mouse	chr3:32508609-32520811:-	BC029173	BC029173	Mus musculus RIKEN cDNA 4833424P18 gene, mRNA (cDNA clone IMAGE:4954230), partial cds. CDS=3..755
Human	chr3:181802620-181818837:+	AY358762	AY358762	Homo sapiens clone DNA23336 DRDL5813 (UNQ5813) mRNA, complete cds.
Human	chr3:181803348-182071495:-	AW181942	BC047103	Homo sapiens, clone IMAGE:5295615, mRNA.
Mouse	chr3:33594135-33610080:+	A430086E15	9330180L23	hypothetical TPR repeat containing protein
Mouse	chr3:33604951-33638497:-	4921507O14	4921507O14	hypothetical protein
Human	chr3:181814885-181815537:+	CA392910	-	-
Human	chr3:181803348-182071495:-	AL122120	BC047103	Homo sapiens, clone IMAGE:5295615, mRNA.
Mouse	chr3:33594135-33610080:+	A430086E15	9330180L23	hypothetical TPR repeat containing protein
Mouse	chr3:33604951-33638497:-	4921507O14	4921507O14	hypothetical protein
Human	chr3:185536419-185546765:+	AY358435	BC037299	Homo sapiens, clone MGC:33603 IMAGE:4825140, mRNA, complete cds.
Human	chr3:185546675-185562093:-	BC072004	BC072004	Homo sapiens chloride channel 2, mRNA (cDNA clone MGC:88707 IMAGE:6424015), complete cds.
Mouse	chr16:20465934-20475741:+	BC003957	C630035L06	hypothetical Bacterial extracellular solute-binding proteins, family 3 containing protein
Mouse	chr16:20475659-20490475:-	AF097415	E130114N01	chloride channel 2
Human	chr3:187983728-187990770:+	BM679630	AL117412	Homo sapiens mRNA; cDNA DKFZp566L241 (from clone DKFZp566L241).
Human	chr3:187990383-188006992:-	M87339	M87339	Human replication factor C, 37-kDa subunit mRNA, complete cds.
Mouse	chr16:22951437-22958132:+	G730016D15	G730016D15	eukaryotic translation initiation factor 4A2
Mouse	chr16:22957935-22971731:-	I920079E18	BC003335	Mus musculus replication factor C (activator 1) 4, mRNA (cDNA clone MGC:6326 IMAGE:3257208), complete cds. CDS=58..1152
Human	chr3:188043173-188058958:+	D45371	AL832470	Homo sapiens mRNA; cDNA DKFZp313G1421 (from clone DKFZp313G1421).
Human	chr3:188052380-188061859:-	BC036509	BC036509	Homo sapiens, clone IMAGE:5266441, mRNA.
Mouse	chr16:22990530-23002022:+	1010001N20	5830442E05	adipocyte, C1Q and collagen domain containing
Mouse	chr16:23000834-23001202:-	1700074D10	1700074D10	unclassifiable
Human	chr3:189353511-190079968:+	AL833171	U49957	Human LIM protein (LPP) mRNA, partial cds.
Human	chr3:189351693-189354581:-	BC042414	BC042414	Homo sapiens, clone IMAGE:4820887, mRNA.
Mouse	chr16:24239224-24829706:+	D130037F19	D130037F19	LIPOMA PREFERRED PARTNER (LPP) homolog [Homo sapiens]
Mouse	chr16:24237963-24240008:-	A330104M08	A330104M08	unclassifiable
Human	chr4:654693-665822:+	AK123974	AK123974	Homo sapiens cDNA FLJ41980 fis, clone SMINT2007391.
Human	chr4:656213-658127:-	D50371	D50371	Homo sapiens mRNA for ATP synthase subunit e, complete cds.
Mouse	chr5:105882031-105883673:+	D830035I06	D830035I06	unclassifiable
Mouse	chr5:105880905-105882960:-	AA475877	2610008D24	ATP synthase, H+ transporting, mitochondrial F1F0 complex, subunit e

Human	chr4:654693-665822:+	L03785	AK123974	Homo sapiens cDNA FLJ41980 fis, clone SMINT2007391.
Human	chr4:665613-673230:-	CR600243	AK025922	Homo sapiens cDNA: FLJ22269 fis, clone HRC03179.
Mouse	chr5:105885631-105889257:+	BX640083	-	-
Mouse	chr5:105888441-105896759:-	4732482E20	4732482E20	hypothetical protein
Human	chr4:970615-988175:+	M74715	M74715	Human alpha-L-iduronidas (IDUA) mRNA, complete cds.
Human	chr4:962695-977054:-	AY124771	BC015517	Homo sapiens solute carrier family 26 (sulfate transporter), member 1, transcript variant 2, mRNA (cDNA clone IMAGE:3885323), complete cds.
Mouse	chr5:106108269-106154598:+	F730041M10	F730041M10	iduronidase, alpha-L-
Mouse	chr5:106117463-106125962:-	AY093420	AY093420	Mus musculus sulfate anion transporter SAT1 (Slc26a1) mRNA, complete cds. CDS=359..2473
Human	chr4:4459411-4538859:+	BC034227	AK094573	Homo sapiens cDNA FLJ37254 fis, clone BRAMY2008583, highly similar to NEURON SPECIFIC PROTEIN FAMILY MEMBER 1.
Human	chr4:4538767-4662145:-	BX647083	CR609491	full-length cDNA clone CS0DL002YA09 of B cells (Ramos cell line) Cot 25-normalized of Homo sapiens (human).
Mouse	chr5:36623562-36646283:-	BC008272	7120465L15	neuron specific gene family member 1
Mouse	chr5:36522274-36624335:+	I920162F24	I920162F24	syntaxin 18
Human	chr4:6759890-6762544:+	AK123360	AK123360	Homo sapiens cDNA FLJ41366 fis, clone BRCAN2004173, highly similar to Homo sapiens T-cell activation protein (PGR1) gene.
Human	chr4:6758778-6761108:-	BF972193	-	-
Mouse	chr5:35259369-35261274:-	1500031F15	9130413I22	T-CELL ACTIVATION PROTEIN homolog [Homo sapiens]
Mouse	chr5:35260730-35262237:+	E330010H09	E330010H09	unclassifiable
Human	chr4:17488831-17522898:+	AK027511	AB013299	Homo sapiens hCAP-G mRNA for chromosome-associated protein-G, complete cds.
Human	chr4:17519086-17699768:-	AL133031	BC037322	Homo sapiens, clone IMAGE:5260413, mRNA, partial cds.
Mouse	chr5:44293321-44328159:+	7420496B13	7420496B13	similar to Condensin subunit 3 (Chromosome-associated protein G) (Condensin subunit CAP-G) (hCAP-G) (XCAP-G homolog) (NY-MEL-3 antigen) [Homo sapiens]
Mouse	chr5:44320580-44478668:-	9330137C05	A730006J22	inferred: chromosome condensation protein G
Human	chr4:20407697-20410449:+	AK125392	AK125392	Homo sapiens cDNA FLJ43402 fis, clone OCBBF2012525.
Human	chr4:20406499-21375666:-	BX648990	BX648990	Homo sapiens mRNA; cDNA DKFZp686J0484 (from clone DKFZp686J0484).
Mouse	chr5:47000902-47042866:+	5730480H06	5730480H06	unclassifiable
Mouse	chr5:47018074-47914232:-	C530018I18	7120434N07	Kv channel interacting protein 4
Human	chr4:38488386-38525702:+	BC051687	BC030662	Homo sapiens Kruppel-like factor 3 (basic), mRNA (cDNA clone MGC:33736 IMAGE:5262512), complete cds.
Human	chr4:38436788-38489088:-	AK023259	AK023259	Homo sapiens cDNA FLJ13197 fis, clone NT2RP3004451.
Mouse	chr5:63433956-63462869:+	2410066O15	2410066O15	Kruppel-like factor 3 (basic)
Mouse	chr5:63432469-63434434:-	B930073D03	D930019G21	hypothetical protein
Human	chr4:44521365-44547910:+	BX647242	BX647242	Homo sapiens mRNA; cDNA DKFZp686M0649 (from clone DKFZp686M0649).
Human	chr4:44525141-44569579:-	AI492449	AF247786	Homo sapiens glucosamine-6-phosphate isomerase SB52 mRNA, complete cds.
Mouse	chr5:68168540-68187588:+	A230069N09	D330023H16	hypothetical GTP-binding elongation factor containing protein
Mouse	chr5:68184726-68203950:-	4933412A11	4933412A11	similar to GLUCOSAMINE-6-PHOSPHATE ISOMERASE (EC 3.5.99.6) (GLUCOSAMINE-6- PHOSPHATE DEAMINASE) (GNPDA) (GLCN6P DEAMINASE) (OSCILLIN) [Mesocricetus auratus]
Human	chr4:48184267-48269157:+	BC040993	AB040891	Homo sapiens mRNA for KIAA1458 protein, partial cds.
Human	chr4:48182765-48185281:-	AK096470	AK096470	Homo sapiens cDNA FLJ39151 fis, clone OCBBF2001749.
Mouse	chr5:71551919-71617295:+	I730090B06	AK129365	Mus musculus mRNA for mKIAA1458 protein. CDS=70..1998
Mouse	chr5:71549539-71552655:-	C230059D16	C230059D16	weakly similar to HYPOTHETICAL 20.7 KDA PROTEIN (FRAGMENT) [Homo sapiens]
Human	chr4:54084738-55005342:+	AK091371	AY229892	Homo sapiens FIP1L1/PDGFRA fusion protein (FIP1L1/PDGFRA fusion) mRNA, complete cds; alternatively spliced.
Human	chr4:54167367-54403234:-	BC022983	BC022983	Homo sapiens, Similar to multi-PDZ-domain-containing protein, clone MGC:29996 IMAGE:4995278, mRNA, complete cds.
Mouse	chr5:73231727-73295134:+	C530024M13	C530024M13	FIP1 like 1 (S. cerevisiae)
Mouse	chr5:73288791-73399550:-	A830092N08	AF034745	Mus musculus LNXp80 (LNX) mRNA, complete cds. CDS=198..2384
Human	chr4:57684864-57738262:+	AU142677	BC023503	Homo sapiens polymerase (RNA) II (DNA directed) polypeptide B, 140kDa, mRNA (cDNA clone MGC:1677 IMAGE:3346389), complete cds.
Human	chr4:57670383-57685917:-	AK128696	AK128696	Homo sapiens cDNA FLJ46863 fis, clone UTERU3011558.
Mouse	chr5:76046455-76085534:+	CA879582	A330105I18	DNA-DIRECTED RNA POLYMERASE II 140 KDA POLYPEPTIDE (EC 2.7.7.6) (RNA POLYMERASE II SUBUNIT 2) (RPB2) homolog [Homo sapiens]
Mouse	chr5:76030476-76046714:-	BF781309	F830208B22	hypothetical protein
Human	chr4:57684864-57738262:+	X63563	BC023503	Homo sapiens polymerase (RNA) II (DNA directed) polypeptide B, 140kDa, mRNA (cDNA clone MGC:1677 IMAGE:3346389), complete cds.
Human	chr4:57737501-57817771:-	BC066339	BC066339	Homo sapiens insulin-like growth factor binding protein 7, mRNA (cDNA clone MGC:87449 IMAGE:5296697), complete cds.
Mouse	chr5:76046455-76085534:+	BC016415	A330105I18	DNA-DIRECTED RNA POLYMERASE II 140 KDA POLYPEPTIDE (EC 2.7.7.6) (RNA POLYMERASE II SUBUNIT 2) (RPB2) homolog [Homo sapiens]
Mouse	chr5:76084526-76319227:-	1810047F02	BC047202	Mus musculus insulin-like growth factor binding protein 7, mRNA (cDNA clone IMAGE:4913609), partial cds. CDS=1..792
Human	chr4:75344926-75533851:+	BC037529	BC032771	Homo sapiens, Similar to RIKEN cDNA 1110019K23 gene, clone MGC:45532 IMAGE:3885359, mRNA, complete cds.
Human	chr4:75387267-75389122:-	BC045815	BC045815	Homo sapiens, clone IMAGE:4830703, mRNA, partial cds.
Mouse	chr5:89745063-89835607:+	BC054409	1110019K23	#############
Mouse	chr5:89744182-89745689:-	CF723084	-	-

Human	chr4:75595895-75619512:+	D30783	D30783	Homo sapiens mRNA for epiregulin, complete cds.
Human	chr4:75509035-75596193:-	CF126154	BC016361	Homo sapiens, clone IMAGE:4105785, mRNA.
Mouse	chr5:89888443-89907474:+	D30782	D30782	Mouse mRNA for epiregulin, complete cds. CDS=139..627
Mouse	chr5:89866621-89888594:-	2210040K03	2210040K03	epiregulin
Human	chr4:80054675-80194705:+	AL137275	AL137661	Homo sapiens mRNA; cDNA DKFZp434P0116 (from clone DKFZp434P0116).
Human	chr4:80165393-80217771:-	BC029604	BC031256	Homo sapiens progestin and adipoQ receptor family member III, mRNA (cDNA clone MGC:39698 IMAGE:5271599), complete cds.
Mouse	chr5:94314270-94408880:+	AY050249	AY050249	Mus musculus BMP-2 inducible kinase mRNA, complete cds. CDS=240..3656
Mouse	chr5:94398832-94428582:-	F830229G03	AY424292	Mus musculus progestin and adipoQ receptor family member III (Paqr3) mRNA, complete cds. CDS=154..1089
Human	chr4:83708680-83739429:+	CF593672	AF113125	Homo sapiens E-1 enzyme (MASA) mRNA, complete cds.
Human	chr4:83700901-83708797:-	BF979634	D89678	Homo sapiens mRNA for A+U-rich element RNA binding factor, complete cds.
Mouse	chr5:97362939-97392339:+	K530356J15	I530023G02	E-1 enzyme homolog
Mouse	chr5:97356937-97363293:-	B020043M01	AB017020	Mus musculus mRNA for JKTBP, complete cds. CDS=124..1029
Human	chr4:83761132-83761673:+	AA825341	-	-
Human	chr4:83761502-83762150:-	BF508344	-	-
Mouse	chr5:97401378-97418689:+	5830403M04	5830403M04	unclassifiable
Mouse	chr5:97401288-97483224:-	BC055321	BC055321	Mus musculus hypothetical protein LOC231503, mRNA (cDNA clone MGC:65588 IMAGE:6419443), complete cds. CDS=354..1103
Human	chr4:107594457-107628218:+	AK095951	AK095951	Homo sapiens cDNA FLJ38632 fis, clone HHDPC2000656, highly similar to Human endothelial-monocyte activating polypeptide II mRNA.
Human	chr4:107324840-107595399:-	BC009208	AK074305	Homo sapiens cDNA FLJ23725 fis, clone HEP14024.
Mouse	chr3:133207777-133231444:-	AA117872	BC002054	Mus musculus small inducible cytokine subfamily E, member 1, mRNA (cDNA clone MGC:6083 IMAGE:3496254), complete cds. CDS=68..1000
Mouse	chr3:133231218-133388871:+	BC060502	9430001M19	hypothetical Tyrosine protein kinase/Serine/Threonine protein kinase/Eukaryotic protein kinase/RabGAP/TBC domain containing protein
Human	chr4:109210129-109232225:+	BC012027	AY343323	Homo sapiens cytochrome P450 mRNA, complete cds.
Human	chr4:109142239-109257683:-	AK123292	AK123292	Homo sapiens cDNA FLJ41298 fis, clone BRAMY2040478.
Mouse	chr3:131874759-131887495:-	A630013N17	D830039I11	cytochrome P450, family 2, subfamily u, polypeptide 1
Mouse	chr3:131886911-131901053:+	G630019B16	G630019B16	unclassifiable
Human	chr4:109446285-109535596:+	BC020624	BC020624	Homo sapiens, clone IMAGE:4809416, mRNA, partial cds.
Human	chr4:109325469-109448039:-	AF288571	AF288571	Homo sapiens lymphoid enhancer factor-1 (LEF1) mRNA, complete cds.
Mouse	chr3:131693432-131694726:-	5830467E13	5830467E13	inferred: lymphoid enhancer binding factor 1
Mouse	chr3:131693006-131807134:+	2610034L06	K330303I08	lymphoid enhancer binding factor 1
Human	chr4:120733554-120831392:+	CR610292	CR610292	full-length cDNA clone CS0DA011YD20 of Neuroblastoma of Homo sapiens (human).
Human	chr4:120515333-120907746:-	D89094	D89094	Homo sapiens mRNA for 3',5'-cyclic GMP phosphodiesterase, complete cds.
Mouse	chr7:27671404-27678300:+	4930447N08	4930447N08	unclassifiable
Mouse	chr7:27668775-27679028:-	G930039B01	-	-
Human	chr4:122200702-122202363:+	BC030136	BC030136	Homo sapiens PR domain containing 5, mRNA (cDNA clone IMAGE:4137157), partial cds.
Human	chr4:121847806-122201630:-	BC066942	AF272897	Homo sapiens PR-domain zinc finger protein 5 (PRDM5) mRNA, complete cds.
Mouse	chr6:65877258-65887964:-	A730092M24	A730092M24	unclassifiable
Mouse	chr6:65887677-66046418:+	E130112L17	E130112L17	weakly similar to ZINC FINGER PROTEIN (FRAGMENT) [Mus musculus]
Human	chr4:123080077-123095783:+	CR621291	BX649106	Homo sapiens mRNA; cDNA DKFZp686L2298 (from clone DKFZp686L2298).
Human	chr4:123095204-123102692:-	CR619143	CR604810	full-length cDNA clone CS0DM008YO22 of Fetal liver of Homo sapiens (human).
Mouse	chr3:36354350-36367845:+	AF152841	BC005622	Mus musculus polymyositis/scleroderma autoantigen 1, mRNA (cDNA clone MGC:11686 IMAGE:3711930), complete cds. CDS=60..1376
Mouse	chr3:36366605-36373894:-	Z26580	B130012D23	cyclin A2
Human	chr4:123897743-123967916:+	BC045668	BC045668	Homo sapiens, clone IMAGE:5298591, mRNA.
Human	chr4:123891388-123899825:-	AF254069	AF254069	Homo sapiens interleukin 21 (IL21) mRNA, complete cds.
Mouse	chr3:37033072-37035081:+	AA276740	-	-
Mouse	chr3:37024500-37034373:-	AF254070	AF254070	Mus musculus interleukin 21 (Il21) mRNA, complete cds. CDS=54..494
Human	chr4:124105468-124176995:+	J04513	J04513	Human basic fibroblast growth factor (bFGF) 22.5 kd, 21 kd and 18 kd protein mRNA, complete cds.
Human	chr4:124167457-124201728:-	AF019633	AF019632	Homo sapiens antisense basic fibroblast growth factor A mRNA, complete cds.
Mouse	chr3:37114258-37211845:+	6030474N20	AF065904	Mus musculus strain C57BL/6J basic fibroblast growth factor (Fgf2) mRNA, complete cds. CDS=1..465
Mouse	chr3:37206649-37221950:-	BC027267	AF453427	Mus musculus anti-sense basic fibroblast growth factor A (Asfgf2a) mRNA, complete cds. CDS=634..1077
Human	chr4:140362457-140363307:+	BM797032	-	-
Human	chr4:140306862-140455977:-	U43189	U43188	Human Ets transcription factor (NERF-2) mRNA, complete cds.
Mouse	chr3:51340874-51344800:+	4930577N17	D930001I15	hypothetical protein
Mouse	chr3:51317581-51390185:-	E430016E08	I920061H01	E74-like factor 2

Human	chr4:159449521-159478344:+	AK129723	AK129723	Homo sapiens cDNA FLJ26212 fis, clone ADG07859.
Human	chr4:159403337-159451807:-	BC043193	BC043193	Homo sapiens hypothetical protein DKFZp434L142, mRNA (cDNA clone MGC:44228 IMAGE:5288748), complete cds.
Mouse	chr3:80252910-80280423:-	4833423B13	4833423B13	unclassifiable
Mouse	chr3:80278150-80340038:+	AF285091	D330040O09	AD021 PROTEIN homolog [Homo sapiens]
Human	chr4:173108135-173111467:+	AK124240	AK124240	Homo sapiens cDNA FLJ42246 fis, clone TKIDN2006761.
Human	chr4:173108019-173109488:-	AF131741	AF131741	Homo sapiens clone 25058 mRNA sequence.
Mouse	chr8:57686929-57687383:-	K330044N12	-	-
Mouse	chr8:57685872-57688413:+	BC030500	BC030500	Mus musculus cDNA sequence BC030500, mRNA (cDNA clone MGC:40983 IMAGE:5401612), complete cds. CDS=152..523
Human	chr4:177616264-177628545:+	AK026302	AK026302	Homo sapiens cDNA: FLJ22649 fis, clone HSI07332.
Human	chr4:177604258-177616789:-	BM564298	-	-
Mouse	chr8:53696299-53705863:-	C130032B15	C130032B15	MICROSOMAL SIGNAL PEPTIDASE 23 KDA SUBUNIT (EC 3.4.-.-) (SPASE 22 KDA SUBUNIT) (SPC22/23) homolog [Canis familiaris]
Mouse	chr8:53705445-53763707:+	F830302I22	1110018D09	ankyrin repeat and SOCs box-containing protein 5
Human	chr4:184395463-184617076:+	AL832424	BX647378	Homo sapiens mRNA; cDNA DKFZp686M24111 (from clone DKFZp686M24111).
Human	chr4:184614369-184616470:-	AK098064	AK098064	Homo sapiens cDNA FLJ40745 fis, clone TRACH2000287, weakly similar to CLAUDIN-6.
Mouse	chr8:46988324-47155307:-	9630050D06	M5C1098O04	claudin 22
Mouse	chr8:46988847-46989840:+	2210404A22	2210404A22	hypothetical PMP-22/EMP/MP20 and claudin family containing protein
Human	chr4:185945916-185991261:+	BC047467	BC047467	Homo sapiens, Similar to hypothetical protein FLJ33167, clone IMAGE:5303049, mRNA.
Human	chr4:185990385-186030435:-	BX355581	AF469667	Homo sapiens KSHV latent nuclear antigen interacting protein 1 (KLIP1) mRNA, complete cds.
Mouse	chr8:45712044-45753762:-	BC065112	BC065112	Mus musculus RIKEN cDNA 1700029A22 gene, mRNA (cDNA clone MGC:86034 IMAGE:30532532), complete cds. CDS=151..1764
Mouse	chr8:45688369-45716468:+	C130069M11	C130069M11	hypothetical protein
Human	chr4:186692235-186696656:+	AY296056	AY296056	Homo sapiens low density lipoprotein receptor-related protein 2-binding protein mRNA, complete cds.
Human	chr4:186660181-186722284:-	AK091635	AK126913	Homo sapiens cDNA FLJ44965 fis, clone BRAWH2016223.
Mouse	chr8:45120230-45129033:-	A930017A02	A930017A02	unclassifiable
Mouse	chr8:45122625-45152937:+	4933402N21	4933402N21	hypothetical TPR repeat containing protein
Human	chr4:186692235-186696656:+	AY296056	AY296056	Homo sapiens low density lipoprotein receptor-related protein 2-binding protein mRNA, complete cds.
Human	chr4:186660181-186722284:-	AK091635	AK126913	Homo sapiens cDNA FLJ44965 fis, clone BRAWH2016223.
Mouse	chr8:45120230-45129033:-	BC039642	A930017A02	unclassifiable
Mouse	chr8:45099006-45120280:+	6430590N12	6430590N12	hypothetical protein
Human	chr5:32209259-32211135:+	AK055939	AK055939	Homo sapiens cDNA FLJ31377 fis, clone NESOP1000087.
Human	chr5:32160573-32210198:-	AK075156	AK057544	Homo sapiens cDNA FLJ32982 fis, clone THYMU1000002, highly similar to Homo sapiens mRNA for Golgi protein (GPP34 gene).
Mouse	chr15:12231175-12232216:-	1810049J17	1810049J17	hypothetical Glycine-rich region profile containing protein
Mouse	chr15:12231750-12262141:+	5730410D03	BC031445	Mus musculus golgi phosphoprotein 3, mRNA (cDNA clone IMAGE:4482612), containing frame-shift errors.
Human	chr5:36287054-36287831:+	BU568414	-	-
Human	chr5:36284861-36337761:-	BC047660	BC047660	Homo sapiens, Similar to hypothetical protein FLJ25422, clone MGC:51303 IMAGE:5261736, mRNA, complete cds.
Mouse	chr15:8946531-8948327:-	9230105E03	9230105E03	unclassifiable
Mouse	chr15:8841135-8952073:+	D630003A19	BC058706	Mus musculus RIKEN cDNA C130037N17 gene, mRNA (cDNA clone MGC:76413 IMAGE:6839130), complete cds. CDS=308..1783
Human	chr5:43638573-43742315:+	AL831822	AL831822	Homo sapiens mRNA; cDNA DKFZp451B113 (from clone DKFZp451B113); complete cds.
Human	chr5:43607418-43638936:-	BM510280	BC012288	Homo sapiens, clone IMAGE:2900205, mRNA.
Mouse	chr13:116305563-116383447:-	G430147B22	G430147B22	nicotinamide nucleotide transhydrogenase
Mouse	chr13:116379743-116380706:+	4930423F13	4930423F13	hypothetical protein
Human	chr5:54639345-54757282:+	BC065258	BC065258	Homo sapiens KIAA0052 protein, mRNA (cDNA clone IMAGE:6146338), partial cds.
Human	chr5:54756263-54866663:-	Y14436	Y14436	Homo sapiens mRNA for phosphatidic acid phosphatase type 2.
Mouse	chr13:109807830-109868339:-	BC014810	BC029230	Mus musculus RIKEN cDNA 2610528A15 gene, mRNA (cDNA clone MGC:36225 IMAGE:4488921), complete cds. CDS=14..3136
Mouse	chr13:109740154-109808306:+	5033401M15	5033401M15	phosphatidic acid phosphatase 2a
Human	chr5:61637790-61960276:+	BU150229	AK001696	Homo sapiens cDNA FLJ10834 fis, clone NT2RP4001207.
Human	chr5:61719535-61735523:-	CR594603	CR598329	full-length cDNA clone CS0DB004YC06 of Neuroblastoma Cot 10-normalized of Homo sapiens (human).
Mouse	chr13:103709521-103773078:-	6330556J23	D12644	Mus musculus mRNA for KIF2 protein, complete cds. CDS=505..2655
Mouse	chr13:103698369-103711369:+	6430407C24	6430407C24	PUTATIVE DIMETHYLADENOSINE TRANSFERASE (FRAGMENT) homolog [Homo sapiens]
Human	chr5:64956344-64997709:+	BC047475	AK023673	Homo sapiens cDNA FLJ13611 fis, clone PLACE1010802.
Human	chr5:64997511-65000996:-	AK090594	AK090594	Homo sapiens cDNA FLJ33275 fis, clone ASTRO2008040.
Mouse	chr13:100902248-100939210:-	D230034D01	D230034D01	hypothetical protein
Mouse	chr13:100869868-100902594:+	6330524H23	6330524H23	small glutamine-rich tetratricopeptide repeat (TPR)-containing, beta

Human	chr5:68700880-68746384:+	AF126424	AL122068	Homo sapiens mRNA; cDNA DKFZp434A1135 (from clone DKFZp434A1135); complete cds.
Human	chr5:68682567-68701682:-	AF110777	AF110777	Homo sapiens adrenal gland protein AD-004 mRNA, complete cds.
Mouse	chr13:97323240-97357067:-	6720482I03	5930425O05	RAD17 homolog (S. pombe)
Mouse	chr13:97356985-97389169:+	AA545585	2810046E22	ADRENAL GLAND PROTEIN AD-004 homolog [Homo sapiens]
Human	chr5:73971604-74054223:+	CR601804	AK130002	Homo sapiens cDNA FLJ26492 fis, clone KDN06302, highly similar to Beta-hexosaminidase beta chain precursor (EC 3.2.1.52).
Human	chr5:74052785-74098952:-	AF111808	AF367997	Homo sapiens elongation factor G2 (EFG2) mRNA, complete cds; nuclear gene for mitochondrial product.
Mouse	chr13:93907321-93929315:-	I830026A18	I830026A18	hexosaminidase B
Mouse	chr13:93869126-93912190:+	6030430C17	6030430C17	Elongation factor G 2, mitochondrial precursor (mEF-G 2) (Elongation factor G2) homolog [Mus musculus]
Human	chr5:73971604-74054223:+	CR601804	AK130002	Homo sapiens cDNA FLJ26492 fis, clone KDN06302, highly similar to Beta-hexosaminidase beta chain precursor (EC 3.2.1.52).
Human	chr5:74052785-74098952:-	AF111808	AF367997	Homo sapiens elongation factor G2 (EFG2) mRNA, complete cds; nuclear gene for mitochondrial product.
Mouse	chr13:93903034-93906949:-	6330524M08	6330524M08	unclassifiable
Mouse	chr13:93869126-93912190:+	9630008A09	6030430C17	Elongation factor G 2, mitochondrial precursor (mEF-G 2) (Elongation factor G2) homolog [Mus musculus]
Human	chr5:74098553-74110252:+	BF678123	AF077615	Homo sapiens TGF beta inducible nuclear protein TINP1 (TINP1) mRNA, complete cds.
Human	chr5:74109155-74198532:-	BX640902	BX640902	Homo sapiens mRNA; cDNA DKFZp686O1754 (from clone DKFZp686O1754).
Mouse	chr13:93860610-93869115:-	5730427N09	5730427N09	HYPOTHETICAL 30.1 KDA PROTEIN (TGF BETA INDUCIBLE NUCLEAR PROTEIN TINP1) (HAIRY CELL LEUKEMIA PROTEIN 1) homolog [Homo sapiens]
Mouse	chr13:93798580-93861632:+	D130013O06	B230112C05	unclassifiable
Human	chr5:78316341-78318114:+	CR604870	CR604870	full-length cDNA clone CS0DI022YA21 of Placenta Cot 25-normalized of Homo sapiens (human).
Human	chr5:78111149-78317522:-	BC029051	M32373	Human arylsulfatase B (ASB) mRNA, complete cds.
Mouse	chr13:90496640-90497885:-	A030001P10	A030001P10	unclassifiable
Mouse	chr13:90497321-90668031:+	B230322K18	B230322K18	arylsulfatase B
Human	chr5:78443358-78463871:+	U50929	BC012616	Homo sapiens, clone MGC:13645 IMAGE:4045543, mRNA, complete cds.
Human	chr5:78451856-78567517:-	AW444926	-	-
Mouse	chr13:90339043-90368372:-	I1C0027L03	I1C0027L03	betaine-homocysteine methyltransferase
Mouse	chr13:90320686-90369637:+	4930572N12	4930572N12	unclassifiable
Human	chr5:86725245-86725976:+	AI742769	-	-
Human	chr5:86707365-86744597:-	BC016705	BX537673	Homo sapiens mRNA; cDNA DKFZp686E04169 (from clone DKFZp686E04169).
Mouse	chr13:81836504-81840425:-	A930002L21	A930002L21	unclassifiable
Mouse	chr13:81815797-81851895:+	6330408H09	6330408H09	cyclin H
Human	chr5:92944795-92956077:+	BU507999	AF087978	Homo sapiens full length insert cDNA clone YW26E10.
Human	chr5:92703702-92947103:-	BG701915	BC042879	Homo sapiens, clone IMAGE:5313822, mRNA.
Mouse	chr13:74723403-74797658:-	X74134	A230057M04	unclassifiable
Mouse	chr13:74797380-74878492:+	C230008G15	D130047J24	inferred: ORF2 consensus sequence encoding endonuclease and reverse transcriptase minus RNaseH {Rattus norvegi
Human	chr5:95891281-96143696:+	AY189741	BC013579	Homo sapiens, Similar to calpastatin, clone MGC:9402 IMAGE:3878564, mRNA, complete cds.
Human	chr5:96121000-96297269:-	AB011097	AK094985	Homo sapiens cDNA FLJ37666 fis, clone BRHIP2011576.
Mouse	chr13:71137586-71253085:-	9930114B02	4732460P18	calpastatin
Mouse	chr13:71084946-71138900:+	E330015C24	9830133K10	type 1 tumor necrosis factor receptor shedding aminopeptidase regulator
Human	chr5:110101758-110128760:+	BI562237	AK091427	Homo sapiens cDNA FLJ34108 fis, clone FCBBF3008251, highly similar to Human TB1 gene mRNA.
Human	chr5:110101520-110102097:-	AI968300	-	-
Mouse	chr18:32012749-32067235:-	4930509H08	6530437D17	similar to TB1 PROTEIN (FRAGMENT) [Homo sapiens]
Mouse	chr18:32066215-32067456:+	B930094E09	B930094E09	hypothetical protein
Human	chr5:111524122-111527872:+	CR601802	AB048207	Homo sapiens mRNA for TIGA1, complete cds.
Human	chr5:111506037-111783315:-	AL117425	AB030240	Homo sapiens mRNA for hNBL4, complete cds.
Mouse	chr18:34236281-34238566:+	C130077E22	C130077E22	unclassifiable
Mouse	chr18:34235691-34446675:-	BC011099	BC007166	Mus musculus erythrocyte protein band 4.1-like 4a, mRNA (cDNA clone MGC:7458 IMAGE:3490061), complete cds. CDS=502..2562
Human	chr5:131308002-131331812:+	BG207204	-	-
Human	chr5:131170582-131375678:-	AF099740	AF099740	Homo sapiens long chain fatty acyl CoA synthetase 2 (LACS2) mRNA, complete cds.
Mouse	chr11:53980081-53993219:-	AA422420	-	-
Mouse	chr11:53943547-54004495:+	AK122384	M5C1006O09	acyl-CoA synthetase long-chain family member 6
Human	chr5:134396869-134719153:+	AK026965	AK026965	Homo sapiens cDNA: FLJ23312 fis, clone HEP11874.
Human	chr5:134697963-134763476:-	AK023409	AF054174	Homo sapiens histone macroH2A1.2 mRNA, complete cds.
Mouse	chr13:55060833-55183995:+	A830006E17	A830006E17	unclassifiable
Mouse	chr13:55172533-55234697:-	E430002M18	E430002M18	H2A histone family, member Y

Human	chr5:137251559-137306331:+	BC044581	AF182034	Homo sapiens polycystic kidney disease-like 2 protein (PKDL2) mRNA, complete cds.
Human	chr5:137301548-137415503:-	AF251038	AF251038	Homo sapiens GAP-like protein (N61) mRNA, complete cds.
Mouse	chr18:34847852-34883480:+	4932443G22	4932443G22	polycystic kidney disease 2-like 2
Mouse	chr18:34879935-34946195:-	BC029030	BC031465	Mus musculus expressed sequence AW060714, mRNA (cDNA clone MGC:25564 IMAGE:3981146), complete cds. CDS=498..3053
Human	chr5:137542586-137551761:+	AK025790	AK025790	Homo sapiens cDNA: FLJ22137 fis, clone HEP20932, highly similar to AF153329 Homo sapiens RAB6KIFL mRNA.
Human	chr5:137551227-137576932:-	AF053977	AF053977	Homo sapiens cell division cycle protein 23 (CDC23) mRNA, complete cds.
Mouse	chr18:35063977-35072641:+	D330036L06	D330036L06	Rab6, kinesin-like
Mouse	chr18:35070310-35091114:-	C030040E15	D530027D05	ANAPHASE-PROMOTING COMPLEX SUBUNIT 8 homolog [Homo sapiens]
Human	chr5:137701136-137713317:+	AK027412	AK027412	Homo sapiens cDNA FLJ14506 fis, clone NT2RM1000365.
Human	chr5:137648853-137701943:-	AK097710	AK097710	Homo sapiens cDNA FLJ40391 fis, clone TESTI2036833, highly similar to M-PHASE INDUCER PHOSPHATASE 3 (EC 3.1.3.48).
Mouse	chr18:35198850-35214008:+	BC057111	BC057111	Mus musculus RIKEN cDNA 2810012G03 gene, mRNA (cDNA clone MGC:61397 IMAGE:6417613), complete cds. CDS=392..1573
Mouse	chr18:35196650-35200146:-	AI504636	-	-
Human	chr5:139924225-139929327:+	CB989077	AK122601	Homo sapiens cDNA FLJ16009 fis, clone NT2RI2003993.
Human	chr5:139896949-139924470:-	BC013158	AK054960	Homo sapiens cDNA FLJ30398 fis, clone BRACE2008402, highly similar to Homo sapiens steroid receptor RNA activator isoform 3 mRNA.
Mouse	chr18:37122415-37127068:+	K630072C13	F630012G11	solute carrier family 35, member A4
Mouse	chr18:37109881-37122674:-	BC024809	BC024809	Mus musculus amyloid beta (A4) precursor protein-binding, family B, member 3, mRNA (cDNA clone MGC:38710 IMAGE:5357681), complete cds. CDS=322..1782
Human	chr5:139956335-140005575:+	BC065828	CR594879	full-length cDNA clone CS0DI064YE19 of Placenta Cot 25-normalized of Homo sapiens (human).
Human	chr5:139998707-140007545:-	AF047185	AF077029	Homo sapiens NADH-ubiquinone oxidoreductase B8 subunit mRNA, nuclear gene encoding mitochondrial protein, complete cds.
Mouse	chr18:37178239-37185597:+	BC005637	BC005637	Mus musculus RIKEN cDNA 2410015B03 gene, mRNA (cDNA clone MGC:7060 IMAGE:3156734), complete cds. CDS=825..1589
Mouse	chr18:37185532-37187777:-	BQ746689	AF124786	Mus musculus NADH-ubiquinone oxidoreductase B8 subunit (Ndufa2) mRNA, complete cds; nuclear gene for mitochondrial product. CDS=20..319
Human	chr5:140024581-140033893:+	BC002482	BC002482	Homo sapiens hypothetical protein FLJ20195, mRNA (cDNA clone IMAGE:3347441).
Human	chr5:140030563-140033355:-	BC033496	BC033496	Homo sapiens, Similar to cDNA sequence BC013481, clone MGC:34750 IMAGE:5172595, mRNA, complete cds.
Mouse	chr18:37203420-37207010:+	BC011484	I530029I21	16 days embryo lung cDNA, RIKEN full-length enriched library, clone:8430437C04 product:hypothetical protein FLJ21702 homolog (CDNA: FLJ21702 FIS)
Mouse	chr18:37206866-37209414:-	BC034897	BC034897	Mus musculus dead end homolog 1 (zebrafish), mRNA (cDNA clone MGC:41452 IMAGE:3470697), complete cds. CDS=420..1442
Human	chr5:140051202-140059087:+	BC014982	BC014982	Homo sapiens, histidyl-tRNA synthetase-like, clone MGC:23192 IMAGE:4859849, mRNA, complete cds.
Human	chr5:140033673-140051631:-	Z11518	Z11518	H.sapiens mRNA for histidyl-tRNA synthetase.
Mouse	chr18:37226208-37235767:+	4631412B19	BC004596	Mus musculus histidyl-tRNA synthetase-like, mRNA (cDNA clone MGC:6398 IMAGE:3584555), complete cds. CDS=114..1631
Mouse	chr18:37209728-37226779:-	AU067020	I920075H21	histidyl-tRNA synthetase
Human	chr5:140146060-140372120:+	AF152311	AF152305	Homo sapiens protocadherin alpha 1 (PCDH-alpha1) mRNA, complete cds.
Human	chr5:140159865-140230137:-	BC043441	BC043441	Homo sapiens protocadherin alpha 6, mRNA (cDNA clone IMAGE:5297539), partial cds.
Mouse	chr18:37373341-38285036:+	AY013758	AF464180	Mus musculus protocadherin mRNA, complete cds. CDS=26..2785
Mouse	chr18:37452026-37463836:-	A830007O22	A830007O22	protocadherin alpha 11
Human	chr5:140996690-141000828:+	BC009560	AK054889	Homo sapiens cDNA FLJ30327 fis, clone BRACE2007191.
Human	chr5:140999053-141011170:-	AY217346	AY217346	Homo sapiens FLJ00007-like protein mRNA, complete cds.
Mouse	chr18:38398236-38402336:+	A830089I08	A830089I08	hypothetical protein
Mouse	chr18:38400589-38412931:-	BC006858	F630104O09	FCH and double SH3 domains 1
Human	chr5:175597974-175705748:+	AA292933	BC066980	Homo sapiens FLJ44216 protein, mRNA (cDNA clone MGC:87612 IMAGE:4821958), complete cds.
Human	chr5:175705670-175721577:-	BC006316	BX648859	Homo sapiens mRNA; cDNA DKFZp686K02233 (from clone DKFZp686K02233).
Mouse	chr13:53593909-53641485:+	4732471D19	BC059035	Mus musculus RIKEN cDNA 4732471D19 gene, mRNA (cDNA clone IMAGE:6812002), partial cds. CDS=2..2395
Mouse	chr13:53638328-53655623:-	G830027I08	G830027I08	Aa2-141 homolog [Rattus norvegicus]
Human	chr5:176170105-176240505:+	AB075856	AK131380	Homo sapiens cDNA FLJ16449 fis, clone BRAWH2006395, highly similar to Rattus norvegicus transmembrane receptor Unc5H1.
Human	chr5:176240473-176258946:-	U42303	BC028129	Homo sapiens, hexokinase 3 (white cell), clone MGC:40018 IMAGE:5211860, mRNA, complete cds.
Mouse	chr13:54039166-54095781:+	BC058084	BC058084	Mus musculus unc-5 homolog A (C. elegans), mRNA (cDNA clone MGC:66671 IMAGE:6813463), complete cds. CDS=252..2780
Mouse	chr13:54095748-54111676:-	G530115K02	F730311O19	hexokinase 3
Human	chr5:176663369-176666566:+	AF151864	BC018904	Homo sapiens px19-like protein, mRNA (cDNA clone IMAGE:3957371).
Human	chr5:176660805-176711484:-	AX747759	CR599240	full-length cDNA clone CS0DG007YN18 of B cells (Ramos cell line) of Homo sapiens (human).
Mouse	chr13:54409802-54414575:+	BC025859	BC059038	Mus musculus RIKEN cDNA 2610524G07 gene, mRNA (cDNA clone MGC:69780 IMAGE:6812461), complete cds. CDS=1691..2344
Mouse	chr13:54414470-54419046:-	A430041D12	A430041D12	Max dimerization protein 3
Human	chr5:176876668-176877507:+	CA307819	-	-
Human	chr5:176871183-176877076:-	AK091545	AK091545	Homo sapiens cDNA FLJ34226 fis, clone FCBBF3023667, highly similar to Homo sapiens DEAD-box protein abstrakt (ABS) mRNA.
Mouse	chr13:54632986-54633913:+	BE650060	-	-
Mouse	chr13:54627401-54633649:-	2900024F02	I830030A19	DEAD (Asp-Glu-Ala-Asp) box polypeptide 41

Human	chr5:177564097-177570790:+	BC036708	AK097657	Homo sapiens cDNA FLJ40338 fis, clone TESTI2032067, highly similar to Rattus norvegicus mRNA for type A/B hnRNP protein p40.
Human	chr5:177567436-177592481:-	AK093737	BC037238	Homo sapiens, clone IMAGE:5198523, mRNA.
Mouse	chr11:51235917-51246620:-	2900056O08	2900056O08	heterogeneous nuclear ribonucleoprotein A/B
Mouse	chr11:51224496-51244228:+	4932420A11	4932420A11	hypothetical Aminotransferase class-III containing protein
Human	chr5:179165994-179197954:+	BC017222	BC001874	Homo sapiens, Similar to sequestosome 1, clone MGC:1276 IMAGE:3535436, mRNA, complete cds.
Human	chr5:179194009-179218466:-	BX537968	BX537968	Homo sapiens mRNA; cDNA DKFZp686L2452 (from clone DKFZp686L2452).
Mouse	chr11:49839104-49850742:-	4732468D23	4732468D23	sequestosome 1
Mouse	chr11:49814183-49839855:+	BC028428	3010026O09	hypothetical protein
Human	chr6:4024442-4119377:+	BC047663	BC047663	Homo sapiens, Similar to RIKEN cDNA 4933417A18 gene, clone IMAGE:5266746, mRNA.
Human	chr6:4060922-4080830:-	BC025287	AK057175	Homo sapiens cDNA FLJ32613 fis, clone STOMA2000104, highly similar to DBI-RELATED PROTEIN 1.
Mouse	chr13:34396218-34422688:+	2700007L21	2700007L21	peroxisomal delta3, delta2-enoyl-Coenzyme A isomerase
Mouse	chr13:34378850-34459332:-	AF153613	C630015K10	unclassifiable
Human	chr6:7671331-7826962:+	M60315	M60315	Human transforming growth factor-beta BMP protein (tgf-beta) mRNA, complete cds.
Human	chr6:7826482-8047827:-	AY326464	BC052310	Homo sapiens thioredoxin domain containing 5, mRNA (cDNA clone MGC:59718 IMAGE:6671945), complete cds.
Mouse	chr13:37785823-37942988:+	A530090N18	A530090N18	bone morphogenetic protein 6
Mouse	chr13:37942620-37971368:-	D230014I09	D230014I09	thioredoxin domain containing 5
Human	chr6:10487688-10818001:+	BX438453	AK000631	Homo sapiens cDNA FLJ20624 fis, clone KAT04557.
Human	chr6:10779637-10803016:-	BX280692	BC016820	Homo sapiens, clone IMAGE:4096427, mRNA, partial cds.
Mouse	chr13:40443417-40455587:+	5930415H02	AF386076	Mus musculus p21-activated protein kinase-interacting protein 1 mRNA, complete cds. CDS=307..1551
Mouse	chr13:40400006-40443560:-	A730081D07	A730081D07	glucosaminyltransferase, I-branching enzyme
Human	chr6:10520536-10524707:+	BC037167	BC037167	Homo sapiens cDNA clone IMAGE:4132069, partial cds.
Human	chr6:10501405-10527874:-	M36711	M36711	Human sequence-specific DNA-binding protein (AP-2) mRNA, complete cds.
Mouse	chr13:40171315-40178669:+	5430439A09	5430439A09	transcription factor AP-2, alpha
Mouse	chr13:40158810-40181738:-	U17285	X74216	M.musculus mRNA for transcription activator AP-2. CDS=71..1384
Human	chr6:13594505-13595063:+	R27426	-	-
Human	chr6:13472106-13595030:-	AL597577	AK055131	Homo sapiens cDNA FLJ30569 fis, clone BRAWH2005974, weakly similar to Oxidoreductase.
Mouse	chr13:42754709-42757522:+	C230040D14	C230040D14	unclassifiable
Mouse	chr13:42716722-42755715:-	E130012P04	E130012P04	CDNA FLJ20330 FIS, CLONE HEP10296 homolog [Homo sapiens]
Human	chr6:13723538-13740902:+	AK075301	AK075301	Homo sapiens cDNA FLJ90820 fis, clone Y79AA1001272.
Human	chr6:13729707-13820057:-	BC063849	BC052781	Homo sapiens RAN binding protein 9, mRNA (cDNA clone IMAGE:5272048), partial cds.
Mouse	chr13:42850476-42859960:+	AB041566	AB041566	Mus musculus brain cDNA, clone MNCb-1192, similar to Homo sapiens retinoic acid repressible protein (RARG-1) mRNA. CDS=16..420
Mouse	chr13:42854776-42933581:-	BC050877	AF006465	Mus musculus B cell antigen receptor Ig beta associated protein 1 (IBAP-1) mRNA, complete cds. CDS=46..2007
Human	chr6:24603014-24645414:+	BC034321	AJ427355	Homo sapiens mRNA for succinic semialdehyde dehydrogenase (SSADH gene).
Human	chr6:24532176-24603407:-	AI802084	AJ308108	Homo sapiens mRNA for glycosylphosphatidylinositol phospholipase D (GPLD1 gene).
Mouse	chr13:24287527-24317598:-	F530115F16	F530115F16	Succinate semialdehyde dehydrogenase
Mouse	chr13:24285530-24318986:+	4932702P03	4932702P03	aldehyde dehydrogenase family 5, subfamily A1
Human	chr6:30142465-30146266:+	BC003144	X81003	Homo sapiens HCG V mRNA.
Human	chr6:30146022-30151643:-	AF238317	AF238317	Homo sapiens HZFw3 protein mRNA, complete cds.
Mouse	chr17:35471292-35474738:-	9630054D02	9630054D02	protein phosphatase 1, regulatory (inhibitor) subunit 11
Mouse	chr17:35465609-35471360:+	G830031A11	BC051578	Mus musculus cDNA clone IMAGE:1513964.
Human	chr6:30632206-30639479:+	U63336	U63336	Human MHC Class I region proline rich protein mRNA, complete cds.
Human	chr6:30621633-30632987:-	BC018366	BC018366	Homo sapiens, clone MGC:9650 IMAGE:3924808, mRNA, complete cds.
Mouse	chr17:34479354-34487168:-	AJ504719	AJ504719	Mus musculus mRNA for proline rich protein (CAT56 gene). CDS=999..1571
Mouse	chr17:34486783-34495349:+	6430407H01	6430407H01	GUANINE NUCLEOTIDE-BINDING PROTEIN-LIKE 1 (GTP-BINDING PROTEIN MMR1)
Human	chr6:30722795-30728966:+	BC064397	BC073975	Homo sapiens chromosome 6 open reading frame 136, mRNA (cDNA clone IMAGE:5764635), partial cds.
Human	chr6:30728248-30748793:-	BE393203	AB011149	Homo sapiens mRNA for KIAA0577 protein, complete cds.
Mouse	chr17:34404348-34409162:-	G830039F06	G830039F06	hypothetical Proline-rich region profile containing protein
Mouse	chr17:34391520-34404435:+	BC009147	BC009147	Mus musculus DEAH (Asp-Glu-Ala-His) box polypeptide 16, mRNA (cDNA clone MGC:6487 IMAGE:2647184), complete cds. CDS=94..3228
Human	chr6:30818685-30819566:+	CN408738	-	-
Human	chr6:30818955-30820304:-	S81914	S81914	IEX-1=radiation-inducible immediate-early gene [human, placenta, mRNA Partial, 1223 nt].
Mouse	chr17:34332121-34332764:-	G370022D09	G370022D09	unclassifiable
Mouse	chr17:34330861-34332797:+	D030053M22	X67644	M.musculus gly96 mRNA.

Human	chr6:31740990-31749302:+	CR625128	CR598133	full-length cDNA clone CS0DK010YC12 of HeLa cells Cot 25-normalized of Homo sapiens (human).
Human	chr6:31736985-31742039:-	AK123707	AK123707	Homo sapiens cDNA FLJ41713 fis, clone HLUNG2011833, highly similar to Homo sapiens MSH55 gene.
Mouse	chr17:33610313-33618422:-	BU962052	4930407M15	casein kinase II, beta subunit
Mouse	chr17:33617844-33621194:+	I1C0007K21	BC057902	Mus musculus HLA-B associated transcript 4, mRNA (cDNA clone MGC:65455 IMAGE:5027029), complete cds. CDS=265..1314
Human	chr6:31794350-31802470:+	AX747518	AX747518	Sequence 1043 from Patent EP1308459.
Human	chr6:31801342-31802468:-	AF116606	AF116606	Homo sapiens PRO0890 mRNA, complete cds.
Mouse	chr17:33559062-33562553:-	9830125K14	9830125K14	hypothetical G6b/Immunoglobulin-like containing protein
Mouse	chr17:33555404-33559839:+	BG068814	BC003328	Mus musculus dimethylarginine dimethylaminohydrolase 2, mRNA (cDNA clone MGC:5866 IMAGE:3158175), complete cds. CDS=251..1108
Human	chr6:31815720-31840606:+	AL122094	AJ245661	Homo sapiens mRNA for G7 protein (G7 gene located in the class III region of the major histocompatibility complex.
Human	chr6:31839891-31841344:-	BU680278	-	-
Mouse	chr17:33522328-33524285:-	2810436B06	2810436B06	Ng23 protein
Mouse	chr17:33512948-33523110:+	AF134319	AF134319	Mus musculus G7c protein (G7c) mRNA, G7c-d allele, complete cds. CDS=205..2880
Human	chr6:32229207-32244040:+	AF020544	AF020544	Homo sapiens inactive palmitoyl-protein thioesterase-2i (PPT2) mRNA, complete cds.
Human	chr6:32224112-32230128:-	AL050203	BC013201	Homo sapiens, Similar to chromosome 6 open reading frame 31, clone MGC:18030 IMAGE:3924575, mRNA, complete cds.
Mouse	chr17:33112563-33125406:-	BM508770	BC052330	Mus musculus palmitoyl-protein thioesterase 2, mRNA (cDNA clone IMAGE:6530235), partial cds. CDS=1..1017
Mouse	chr17:33123429-33124596:+	2610029K11	2610029K11	unclassifiable
Human	chr6:32229207-32244040:+	BC052591	AF020544	Homo sapiens inactive palmitoyl-protein thioesterase-2i (PPT2) mRNA, complete cds.
Human	chr6:32243961-32253847:-	BC004310	BC002402	Homo sapiens 1-acylglycerol-3-phosphate O-acyltransferase 1 (lysophosphatidic acid acyltransferase, alpha), transcript variant 2, mRNA (cDNA clone MGC:8511 IMAGE:2822348), complete cds.
Mouse	chr17:33109251-33111872:-	BC055829	7120439H06	similar to NG3 (C6orf8 protein) [Homo sapiens]
Mouse	chr17:33099978-33109349:+	BC009651	D930018E17	
Human	chr6:32919863-32955354:+	AK092738	BC018885	Homo sapiens, clone IMAGE:3626664, mRNA.
Human	chr6:32916471-32920458:-	BC001114	BC001114	Homo sapiens proteasome (prosome, macropain) subunit, beta type, 8 (large multifunctional protease 7), transcript variant 1, mRNA (cDNA clone MGC:1491 IMAGE:2967119), complete cds.
Mouse	chr17:32696101-32703656:-	K630018J04	-	-
Mouse	chr17:32695864-32699598:+	I730030J16	I730030J16	proteosome (prosome, macropain) subunit, beta type 8 (large multifunctional protease 7)
Human	chr6:33276204-33280194:+	BX338266	BC000645	Homo sapiens solute carrier family 39 (zinc transporter), member 7, mRNA (cDNA clone MGC:1928 IMAGE:3345970), complete cds.
Human	chr6:33269340-33276422:-	BC001167	BC001167	Homo sapiens, retinoid X receptor, beta, clone MGC:1831 IMAGE:3502936, mRNA, complete cds.
Mouse	chr17:32522676-32528791:-	F420006M21	E430010A02	solute carrier family 39 (zinc transporter), member 7
Mouse	chr17:32528537-32535546:+	I420027M12	I420027M12	retinoid X receptor beta
Human	chr6:34312589-34322089:+	BC071864	M23616	Human HMG-Y protein isoform mRNA (HMGI gene), clone 8A.
Human	chr6:34320585-34321981:-	BI253382	-	-
Mouse	chr17:26145023-26152123:+	E430022O13	F730014A09	high mobility group AT-hook 1
Mouse	chr17:26151754-26154198:-	BU526131	0710007O20	hypothetical protein
Human	chr6:34324348-34341636:+	CB121890	-	-
Human	chr6:34322053-34325291:-	BM149750	CR619587	full-length cDNA clone CS0DC014YF03 of Neuroblastoma Cot 25-normalized of Homo sapiens (human).
Mouse	chr17:26153561-26154669:+	C130040N14	C130040N14	hypothetical protein
Mouse	chr17:26151754-26154198:-	0710007O20	0710007O20	hypothetical protein
Human	chr6:36462586-36467746:+	AI831895	-	-
Human	chr6:36466306-36518847:-	AF486827	BC031105	Homo sapiens peroxisomal, testis specific 1, mRNA (cDNA clone IMAGE:5297409).
Mouse	chr17:27520067-27531958:+	BF023599	-	-
Mouse	chr17:27528723-27537112:-	AF486826	1700001G18	hypothetical protein
Human	chr6:41855942-41865857:+	BU182495	AL137721	Homo sapiens mRNA; cDNA DKFZp761H221 (from clone DKFZp761H221).
Human	chr6:41865612-41868781:-	AK023682	AK023682	Homo sapiens cDNA FLJ13620 fis, clone PLACE1010947.
Mouse	chr17:45851763-45856529:-	2410012F04	BC021590	Mus musculus RIKEN cDNA 1110002E23 gene, mRNA (cDNA clone MGC:35768 IMAGE:5068609), complete cds. CDS=366..590
Mouse	chr17:45795084-45851887:+	1110030K09	1110030K09	unclassifiable
Human	chr6:43060264-43088062:+	BC001095	L76702	Homo sapiens protein phosphatase 2A B56-delta (PP2A) mRNA, complete cds.
Human	chr6:43060370-43092560:-	CR606344	BC001754	Homo sapiens, male-enhanced antigen, clone MGC:2286 IMAGE:3355279, mRNA, complete cds.
Mouse	chr17:44840256-44862475:-	AY338234	E430029F03	protein phosphatase 2, regulatory subunit B (B56), delta isoform
Mouse	chr17:44837833-44840509:+	BC013344	2500003F22	MALE-ENHANCED ANTIGEN-1 (MEA-1) homolog [Mus musculus]
Human	chr6:43097318-43150815:+	AK127018	AK127018	Homo sapiens cDNA FLJ45074 fis, clone BRAWH3027440, highly similar to Probable kinesin light chain 3.
Human	chr6:43129745-43135220:-	BP317302	AF132956	Homo sapiens CGI-22 protein mRNA, complete cds.
Mouse	chr17:44787838-44803688:-	BI738683	F630201A09	Kinesin-like protein 8
Mouse	chr17:44803454-44807457:+	1810032H21	1810032H21	mitochondrial ribosomal protein L2

Human	chr6:43503270-43526146:+	AL122095	AK000002	Homo sapiens mRNA for FLJ00002 protein, partial cds.
Human	chr6:43526068-43532348:-	BC000230	AK055380	Homo sapiens cDNA FLJ30818 fis, clone FEBRA2001584, weakly similar to DELTA-LIKE PROTEIN PRECURSOR.
Mouse	chr17:44459566-44484580:-	F630107G01	F630107G01	ATP-binding cassette, sub-family C (CFTR/MRP), member 10
Mouse	chr17:44453798-44459618:+	BC019431	BC019431	Mus musculus EGF-like-domain, multiple 9, mRNA (cDNA clone MGC:30524 IMAGE:4913786), complete cds. CDS=190..1338
Human	chr6:71434200-71660049:+	BC036123	AF442495	Homo sapiens putative protein mRNA, complete cds.
Human	chr6:71623633-71723471:-	AB075843	AB075843	Homo sapiens mRNA for KIAA1963 protein.
Mouse	chr1:24083367-24160414:-	4930465E04	BC006946	Mus musculus stromal membrane-associated protein 1, mRNA (cDNA clone MGC:6912 IMAGE:2810817), complete cds. CDS=135..1457
Mouse	chr1:24000274-24087679:+	AB055902	D630010E12	
Human	chr6:74161192-74184010:+	AJ278110	AJ278110	Homo sapiens mRNA for DEAD-box protein (HAGE gene).
Human	chr6:74134997-74161577:-	BC024931	BC024931	Homo sapiens, clone IMAGE:3918981, mRNA.
Mouse	chr9:78792382-78820196:+	8030471J02	-	-
Mouse	chr9:78791561-78792763:-	4930542C12	4930542C12	hypothetical protein
Human	chr6:80000154-80003236:+	BC042096	BC042096	Homo sapiens, clone IMAGE:5768746, mRNA.
Human	chr6:79967679-80001276:-	AY043282	AY043282	Homo sapiens TRIP7-like protein mRNA, complete cds.
Mouse	chr9:83573984-83575433:+	8030494I21	8030494I21	unclassifiable
Mouse	chr9:83537751-83574754:-	I830128D11	I830128D11	high mobility group nucleosomal binding domain 3
Human	chr6:83834104-83937789:+	AL162056	BX648667	Homo sapiens mRNA; cDNA DKFZp686E0245 (from clone DKFZp686E0245).
Human	chr6:83927588-83959728:-	AL117443	BC001258	Homo sapiens, N-acetylglucosamine-phosphate mutase, clone MGC:5002 IMAGE:3451677, mRNA, complete cds.
Mouse	chr9:86909977-87271757:+	BC035275	6720465G24	hypothetical Histidine-rich region profile containing protein
Mouse	chr9:86988027-87013286:-	9830133N08	9830133N08	PHOSPHOACETYLGLUCOSAMINE MUTASE (EC 5.4.2.3) (PAGM) (ACETYLGLUCOSAMINE PHOSPHOMUTASE) (N-ACETYLGLUCOSAMINE-PHOSPHATE MUTASE) homolog [Homo sapiens]
Human	chr6:88438473-88441973:+	CN262270	-	-
Human	chr6:88441297-88468703:-	CR624811	CR599380	full-length cDNA clone CS0DF008YM02 of Fetal brain of Homo sapiens (human).
Mouse	chr4:34658414-34706578:-	A530092B15	D930038I10	origin of replication 3 homolog (S. cerevisiae)
Mouse	chr4:34642260-34664030:+	9430002H02	2700059D21	hypothetical protein
Human	chr6:90716553-90718429:+	AI081835	-	-
Human	chr6:90692968-91063182:-	AF357835	AJ271878	Homo sapiens mRNA for putative transcription factor (BACH2 gene).
Mouse	chr4:32791040-32803021:-	BC024582	BC024582	Mus musculus cDNA clone IMAGE:5065356, containing frame-shift errors.
Mouse	chr4:32469928-32812640:+	D86604	D86604	Mouse mRNA for Bach protein 2, complete cds. CDS=409..2559
Human	chr6:99979519-99987920:+	BC029846	AK025621	Homo sapiens cDNA: FLJ21968 fis, clone HEP05670.
Human	chr6:99952648-99979924:-	AK074628	AK074628	Homo sapiens cDNA FLJ90147 fis, clone HEMBB1001847, weakly similar to NEUROGENIC PROTEIN BIG BRAIN.
Mouse	chr4:21772440-21774563:-	4921507O08	A330029F17	unclassifiable
Mouse	chr4:21773698-21802590:+	2610316I04	6330513G17	hypothetical Serine-rich region containing protein
Human	chr6:127651906-127653253:+	BI770775	-	-
Human	chr6:127651548-127706443:-	AL834469	AL834469	Homo sapiens mRNA; cDNA DKFZp762M1110 (from clone DKFZp762M1110).
Mouse	chr10:29133805-29152104:-	9530083F22	BC050795	Mus musculus RIKEN cDNA 2610509H23 gene, mRNA (cDNA clone MGC:58704 IMAGE:6742885), complete cds. CDS=611..1690
Mouse	chr10:29101633-29143884:+	I0C0043H03	4732455H22	similar to DJ351K20.2.1 (NOVEL ENOYL COA/ACYL COA HYDRATASE/DEHYDROGENASE TYPE PROTEIN (ISOFORM 1)) (SIMILAR TO UNCHARACTERIZED HYPOTHALAMUS PROTEIN HCDASE) [Homo sapiens]
Human	chr6:147566568-147753305:+	BC043645	BC043645	Homo sapiens, Similar to tomosyn, clone IMAGE:5272542, mRNA.
Human	chr6:147204217-147567375:-	BC044307	BC044307	Homo sapiens, clone IMAGE:5240803, mRNA.
Mouse	chr10:9434113-9579788:-	F630208B08	AF516607	Mus musculus strain Swiss Albino b-tomosyn mRNA, partial cds. CDS=1..2787
Mouse	chr10:9578987-9581973:+	B930083N07	-	-
Human	chr6:150162703-150224671:+	BM803139	AK098739	Homo sapiens cDNA FLJ25873 fis, clone CBR02349, highly similar to PROTEIN-L-ISOASPARTATE(D-ASPARTATE) O-METHYLTRANSFERASE (EC 2.1.1.77).
Human	chr6:150137565-150162910:-	BI562397	AK074311	Homo sapiens cDNA FLJ23731 fis, clone HEP14545.
Mouse	chr10:7297960-7351136:-	L630021I10	BC040750	Mus musculus protein-L-isoaspartate (D-aspartate) O-methyltransferase 1, mRNA (cDNA clone IMAGE:4018001), partial cds.
Mouse	chr10:7333292-7334588:+	C630017G20	A630066F11	unclassifiable
Human	chr6:154452557-154660114:+	AY036623	AY036623	Homo sapiens mu opioid receptor variant MOR-1C (OPRM) mRNA, complete cds, alternatively spliced.
Human	chr6:154567744-154770013:-	AB007863	AJ310566	Homo sapiens mRNA for phosphoinositide-binding protein, isolate PIP3-E.
Mouse	chr10:3308859-3589871:-	AF167568	AF074972	Mus musculus MOR-1M mRNA, complete cds. CDS=106..1218
Mouse	chr10:3366542-3461664:+	F830212P16	F830212P16	Interactor protein for cytohesin exchange factors 1 (Fragment) homolog [Rattus norvegicus]
Human	chr6:155561452-155673864:+	AF120323	AF120323	Homo sapiens T-cell lymphoma invasion and metastasis 2 (TIAM2) mRNA, complete cds.
Human	chr6:155666386-155670821:-	AK022993	AK022993	Homo sapiens cDNA FLJ12931 fis, clone NT2RP2004861.
Mouse	chr17:3326211-3531159:+	AK129474	AB022915	Mus musculus Stef mRNA for sif and Tiam1-like exchange factor, complete cds. CDS=312..5459
Mouse	chr17:3517355-3557598:-	BC032930	BC032930	Mus musculus transcription factor B1, mitochondrial, mRNA (cDNA clone MGC:41387 IMAGE:1382837), complete cds. CDS=1..1038

Human	chr6:169919840-169924086:+	BC051700	BC051700	Homo sapiens PHD finger protein 10, mRNA (cDNA clone IMAGE:4792634), partial cds.
Human	chr6:169921619-169941783:-	BC020954	AJ420510	Homo sapiens mRNA full length insert cDNA clone EUROIMAGE 2120537.
Mouse	chr17:13810441-13857682:+	4832428J15	4832428J15	RIKEN cDNA 1600012H06 gene
Mouse	chr17:13812251-13859713:-	1810055P05	1810055P05	CDNA FLJ10975 FIS, CLONE PLACE1001383, WEAKLY SIMILAR TO ZINC-FINGER PROTEIN UBI-D4 homolog [Homo sapiens]
Human	chr6:170803801-170806547:+	AK094428	AK094428	Homo sapiens cDNA FLJ37109 fis, clone BRACE2020756.
Human	chr6:170803888-170811412:-	S78085	S78085	PDCD2=programmed cell death-2/Rp8 homolog [human, fetal lung, mRNA, 1282 nt].
Mouse	chr17:14337480-14339758:+	A230027F19	A230027F19	unclassifiable
Mouse	chr17:14338671-14344397:-	U10903	B230330P13	programmed cell death 2
Human	chr7:1343609-1355925:+	AL831913	AF059194	Homo sapiens basic-leucine zipper transcription factor MafK (MAFK) mRNA, complete cds.
Human	chr7:1355112-1369169:-	BF508046	AK075043	Homo sapiens cDNA FLJ90562 fis, clone OVARC1001163.
Mouse	chr5:137200173-137211313:+	D42124	F430006H13	v-maf musculoaponeurotic fibrosarcoma oncogene family, protein K (avian)
Mouse	chr5:137210480-137228577:-	M130008F02	C820003N21	similar to DJ5O6.2 (NOVEL PROTEIN SIMILAR TO C. ELEGANS F40E10.6 (ISOFORM 2)) (FRAGMENT) [Homo sapiens]
Human	chr7:5822117-5837519:+	U24169	U24169	Human JTV-1 (JTV-1) mRNA, complete cds.
Human	chr7:5835119-5872082:-	AB037790	AB037790	Homo sapiens mRNA for KIAA1369 protein, partial cds.
Mouse	chr5:141362205-141369346:-	BC024410	BC024480	Mus musculus JTV1 gene, mRNA (cDNA clone MGC:37389 IMAGE:4977193), complete cds. CDS=116..958
Mouse	chr5:141296264-141363761:+	6430574E03	6430574E03	##############
Human	chr7:16233619-16247252:+	BI114370	-	-
Human	chr7:15904398-16234113:-	BX349946	-	-
Mouse	chr12:31407470-31408409:-	D630036H23	D630036H23	hypothetical protein
Mouse	chr12:31407678-31715175:+	B230325G10	BC050993	Mus musculus, clone IMAGE:6507154, mRNA.
Human	chr7:21356073-21724451:+	AJ320497	AJ320497	Homo sapiens mRNA for axonemal beta heavy chain dynein type 11 (DNAH11 gene).
Human	chr7:21713755-21758757:-	BC014630	AK022955	Homo sapiens cDNA FLJ12893 fis, clone NT2RP2004165.
Mouse	chr12:112865720-113236717:-	AF183144	AF183144	Mus musculus left-right dynein (Lrd) mRNA, complete cds. CDS=212..13678
Mouse	chr12:112829550-112866451:+	BC006933	BC006933	Mus musculus cDNA sequence BC006933, mRNA (cDNA clone MGC:11998 IMAGE:3602150), complete cds. CDS=74..1390
Human	chr7:26960022-26968787:+	AK096334	AX747263	Sequence 788 from Patent EP1308459.
Human	chr7:26966575-26967337:-	BF970710	-	-
Mouse	chr6:52122530-52161955:+	I530023N17	I530023N17	unclassifiable
Mouse	chr6:52161368-52168655:-	BC036986	BC036986	Mus musculus homeo box A7, mRNA (cDNA clone IMAGE:4986801), complete cds. CDS=499..825
Human	chr7:27552666-27654178:+	AI833003	BC024600	Homo sapiens, Tax1 (human T-cell leukemia virus type I) binding protein 1, clone IMAGE:4339438, mRNA.
Human	chr7:27643432-27993602:-	BC047229	AX746909	Sequence 434 from Patent EP1308459.
Mouse	chr6:52663203-52717639:+	AI591476	I530028L05	Tax1 (human T-cell leukemia virus type I) binding protein 1
Mouse	chr6:52717541-52859112:-	BU898405	BC048577	Mus musculus expressed sequence AI591476, mRNA (cDNA clone MGC:58600 IMAGE:6706223), complete cds. CDS=251..790
Human	chr7:28770509-28826225:+	AW005639	AK124888	Homo sapiens cDNA FLJ42898 fis, clone BRHIP3009318.
Human	chr7:28766214-28771408:-	BC036337	AB014544	Homo sapiens mRNA for KIAA0644 protein, complete cds.
Mouse	chr6:53770967-53773137:+	BC006619	BC006619	Mus musculus RIKEN cDNA 1200009O22 gene, mRNA (cDNA clone IMAGE:3967858), partial cds. CDS=2..67
Mouse	chr6:53766374-53816913:-	BC043099	A330104H23	unclassifiable
Human	chr7:28959445-29327191:+	AK126784	U28926	Human beta2-chimaerin mRNA, complete cds.
Human	chr7:29323156-29376526:-	BC038570	BC038570	Homo sapiens, clone IMAGE:4347271, mRNA.
Mouse	chr6:53929684-54252951:+	1500040P07	1500040P07	chimerin (chimaerin) 2
Mouse	chr6:54220674-54382570:-	6430501H15	6430501H15	hypothetical protein
Human	chr7:39945828-39947447:+	CR594841	CR594841	full-length cDNA clone CS0CAP005YF11 of Thymus of Homo sapiens (human).
Human	chr7:39945532-39947702:-	BC026265	CR618070	full-length cDNA clone CS0DH004YF17 of T cells (Jurkat cell line) of Homo sapiens (human).
Mouse	chr13:16230817-17066611:-	A230079O06	AF397014	Mus musculus unknown mRNA. CDS=50..862
Mouse	chr13:17066226-17070405:+	5330439N04	5330439N04	hypothetical Glycine-rich region containing protein
Human	chr7:43739275-43768975:+	BC004104	AK001446	Homo sapiens cDNA FLJ10584 fis, clone NT2RP2003737, highly similar to UBIQUITIN-CONJUGATING ENZYME E2-17 KD 2 (EC 6.3.2.19).
Human	chr7:43679390-43739545:-	CK819206	CR749398	Homo sapiens mRNA; cDNA DKFZp686O0457 (from clone DKFZp686O0457).
Mouse	chr11:5656909-5695768:+	2310007D07	1600028I17	unclassifiable
Mouse	chr11:5598771-5657198:-	A630048K14	9530070K24	hypothetical protein
Human	chr7:44697792-44699232:+	BC047792	BC047792	Homo sapiens, clone IMAGE:6471309, mRNA.
Human	chr7:44693884-44698200:-	AY039216	AY039216	Homo sapiens Pur-beta (PURB) mRNA, complete cds.
Mouse	chr11:6369818-6388323:+	BC002209	BC002209	Mus musculus cDNA clone IMAGE:3490279.
Mouse	chr11:6362385-6370897:-	M5H1201E19	BC019459	Mus musculus, Similar to RIKEN cDNA 2310015K15 gene, clone IMAGE:4035590, mRNA.

Human	chr7:71794557-72161784:+	AB014518	AB014518	Homo sapiens mRNA for KIAA0618 protein, complete cds.
Human	chr7:71862773-72167515:-	BC056405	CR601630	full-length cDNA clone CS0DB004YO05 of Neuroblastoma Cot 10-normalized of Homo sapiens (human).
Mouse	chr5:132821248-132839654:-	AF516680	F630037F14	Pom121 protein
Mouse	chr5:132815061-132821913:+	BC025592	F830035B06	NOL1R homolog
Human	chr7:76970394-77053656:+	BC059402	BC059402	Homo sapiens FLJ42526 protein, mRNA (cDNA clone MGC:71764 IMAGE:30347338), complete cds.
Human	chr7:76931628-76971313:-	AF290475	BC037783	Homo sapiens cDNA clone IMAGE:4791585, partial cds.
Mouse	chr5:19275136-19331070:-	8430412F05	8430412F05	weakly similar to CDNA FLJ11220 FIS, CLONE PLACE1008129 [Homo sapiens]
Mouse	chr5:19330237-19332383:+	A730015K04	A730015K04	unclassifiable
Human	chr7:86426379-86584620:+	AK002073	AK095828	Homo sapiens cDNA FLJ38509 fis, clone HCHON2000344, highly similar to Homo sapiens cyclin-D binding Myb-like protein mRNA.
Human	chr7:86469824-86494554:-	BC002837	BC002837	Homo sapiens chromosome 7 open reading frame 23, mRNA (cDNA clone MGC:4175 IMAGE:3634983), complete cds.
Mouse	chr5:8756583-9161703:-	4732497F22	A430062G07	unclassifiable
Mouse	chr5:9100379-9158020:+	4930420K17	B430011A04	unclassifiable
Human	chr7:86426379-86584620:+	AK002073	AK095828	Homo sapiens cDNA FLJ38509 fis, clone HCHON2000344, highly similar to Homo sapiens cyclin-D binding Myb-like protein mRNA.
Human	chr7:86469824-86494554:-	BC002837	BC002837	Homo sapiens chromosome 7 open reading frame 23, mRNA (cDNA clone MGC:4175 IMAGE:3634983), complete cds.
Mouse	chr5:8756583-9161703:-	BC045141	A430062G07	unclassifiable
Mouse	chr5:9119438-9120367:+	1500011L05	1500011L05	unclassifiable
Human	chr7:91721381-91767141:+	BU852969	BC031091	Homo sapiens, hypothetical protein RG083M05.2, clone MGC:33873 IMAGE:5275963, mRNA, complete cds.
Human	chr7:91760987-91802495:-	AB008112	AF026086	Homo sapiens peroxisome biogenesis disorder protein 1 (PEX1) mRNA, complete cds.
Mouse	chr5:3635781-3637884:-	4833413G10	4833413G10	unclassifiable
Mouse	chr5:3602072-3643236:+	5430414H02	5430414H02	peroxisome biogenesis factor 1
Human	chr7:91721381-91767141:+	BU852969	BC031091	Homo sapiens, hypothetical protein RG083M05.2, clone MGC:33873 IMAGE:5275963, mRNA, complete cds.
Human	chr7:91760987-91802495:-	AB008112	AF026086	Homo sapiens peroxisome biogenesis disorder protein 1 (PEX1) mRNA, complete cds.
Mouse	chr5:3638184-3663191:-	9130430G15	9130430G15	hypothetical protein
Mouse	chr5:3602072-3643236:+	5430414H02	5430414H02	peroxisome biogenesis factor 1
Human	chr7:98650915-98661891:+	CR624167	CR624167	full-length cDNA clone CS0DC007YA15 of Neuroblastoma Cot 25-normalized of Homo sapiens (human).
Human	chr7:98661386-98681107:-	BC080580	BC080580	Homo sapiens pentatricopeptide repeat domain 1, mRNA (cDNA clone MGC:99605 IMAGE:6645909), complete cds.
Mouse	chr5:142611313-142619029:+	BC057025	BC057025	Mus musculus cDNA clone MGC:67182 IMAGE:6823459, complete cds. CDS=205..516
Mouse	chr5:142618465-142638060:-	E430025I22	I830022G17	pentatricopeptide repeat domain 1
Human	chr7:99343781-99352621:+	AK131236	BC018705	Homo sapiens, clone MGC:31748 IMAGE:5013882, mRNA, complete cds.
Human	chr7:99334733-99344151:-	CN407213	D55716	Human mRNA for P1cdc47, complete cds.
Mouse	chr5:135552764-135561414:+	6030465F23	6030465F23	adaptor-related protein complex AP-4, mu 1
Mouse	chr5:135545344-135553195:-	4922505B22	4922505B22	minichromosome maintenance deficient 7 (S. cerevisiae)
Human	chr7:99343781-99352621:+	BC018705	BC018705	Homo sapiens, clone MGC:31748 IMAGE:5013882, mRNA, complete cds.
Human	chr7:99349344-99361636:-	CR627390	CR627390	Homo sapiens mRNA; cDNA DKFZp781E21155 (from clone DKFZp781E21155).
Mouse	chr5:135552764-135561414:+	BC011174	6030465F23	adaptor-related protein complex AP-4, mu 1
Mouse	chr5:135559380-135568218:-	F630110A21	A630064K15	contigs=84 ver=1 seqid=36119
Human	chr7:99349735-99352169:+	BU509040	-	-
Human	chr7:99349344-99361636:-	CR624352	CR627390	Homo sapiens mRNA; cDNA DKFZp781E21155 (from clone DKFZp781E21155).
Mouse	chr5:135552764-135561414:+	BC011174	6030465F23	adaptor-related protein complex AP-4, mu 1
Mouse	chr5:135559380-135568218:-	F630110A21	A630064K15	contigs=84 ver=1 seqid=36119
Human	chr7:99828565-99843391:+	BC032497	BC032497	Homo sapiens, Similar to F-box only protein 24, clone IMAGE:5221820, mRNA.
Human	chr7:99831673-99846312:-	AK097705	AK097705	Homo sapiens cDNA FLJ40386 fis, clone TESTI2036114.
Mouse	chr5:134997443-135017439:-	4933422D21	4933422D21	F-box only protein 24
Mouse	chr5:134993538-135072036:+	6330530D12	6330530D12	unclassifiable
Human	chr7:101699093-101707669:+	BC009436	AL133057	Homo sapiens mRNA; cDNA DKFZp434K1815 (from clone DKFZp434K1815); partial cds.
Human	chr7:101707267-101924392:-	BC065711	AF468111	Homo sapiens DNA-directed RNA polymerase II subunit 11 mRNA, complete cds.
Mouse	chr5:133567880-133581182:-	I730034M15	D430018G14	hypothetical Trp-Asp (WD) repeats profile/Leucine-rich repeat/Leucine-rich region/Trp-Asp (WD) repeats circular profile/G-protein beta WD-40 repeats containing protein
Mouse	chr5:133561739-133568055:+	K230026J18	K230026J18	polymerase (RNA) II (DNA directed) polypeptide J
Human	chr7:102147389-102179509:+	U32907	U32907	Human p37NB mRNA, complete cds.
Human	chr7:102047628-102309248:-	AY359238	AY359238	Homo sapiens F-box and leucine-rich repeat protein 13 transcript variant 1 (FBXL13) mRNA, complete cds.
Mouse	chr5:19922314-19954785:+	4833425M04	4833425M04	37 kDa leucine-rich repeat (LRR) protein
Mouse	chr5:19862633-20024550:-	4933409A11	4921539K22	hypothetical F-box domain containing protein

Human	chr7:104248582-104349555:+	AY147037	AY147037	Homo sapiens MLL5 (MLL5) mRNA, complete cds; alternatively spliced.
Human	chr7:104090632-104633767:-	AF520791	U88666	Homo sapiens serine kinase SRPK2 mRNA, complete cds.
Mouse	chr5:21887522-21891502:+	E230022I02	E230022I02	myeloid/lymphoid or mixed-lineage leukemia 5
Mouse	chr5:21890348-22071894:-	9330195C10	BC062941	Mus musculus serine/arginine-rich protein specific kinase 2, mRNA (cDNA clone IMAGE:6842723), complete cds. CDS=447..917
Human	chr7:106403374-106436931:+	AK122785	BC017069	Homo sapiens, clone MGC:9634 IMAGE:3914986, mRNA, complete cds.
Human	chr7:106420170-106420988:-	BQ024805	-	-
Mouse	chr12:26913689-26938115:-	4732469M08	4732469M08	high mobility group box transcription factor 1
Mouse	chr12:26642121-26925210:+	5430405C01	5430405C01	PUTATIVE 13 S GOLGI TRANSPORT COMPLEX 90KD SUBUNIT BRAIN-SPECIFIC ISOFORM homolog [Homo sapiens]
Human	chr7:106403374-106436931:+	BC017069	BC017069	Homo sapiens, clone MGC:9634 IMAGE:3914986, mRNA, complete cds.
Human	chr7:106435766-106798657:-	AU144340	BC068540	Homo sapiens component of oligomeric golgi complex 5, mRNA (cDNA clone MGC:87389 IMAGE:4374289), complete cds.
Mouse	chr12:26913689-26938115:-	4732469M08	4732469M08	high mobility group box transcription factor 1
Mouse	chr12:26642121-26925210:+	5430405C01	5430405C01	PUTATIVE 13 S GOLGI TRANSPORT COMPLEX 90KD SUBUNIT BRAIN-SPECIFIC ISOFORM homolog [Homo sapiens]
Human	chr7:115444498-115492927:+	BC001451	AF245356	Homo sapiens TESTIN 2 mRNA, complete cds.
Human	chr7:115443236-115445516:-	BC040208	BC040208	Homo sapiens, clone IMAGE:4823377, mRNA.
Mouse	chr6:16808904-16848782:+	I1C0049D04	I1C0049D04	testis derived transcript
Mouse	chr6:16802558-16809294:-	A430084P19	G730045L04	unclassifiable
Human	chr7:134290046-134308222:+	CR623230	AK001862	Homo sapiens cDNA FLJ11000 fis, clone PLACE1002794.
Human	chr7:134234316-134312914:-	BC024018	BC050676	Homo sapiens hypothetical protein MGC5242, mRNA (cDNA clone IMAGE:6043548), partial cds.
Mouse	chr6:34763986-34774751:+	BC023802	BC023802	Mus musculus RIKEN cDNA 1110007F12 gene, mRNA (cDNA clone MGC:38515 IMAGE:5352975), complete cds. CDS=233..790
Mouse	chr6:34770103-34778948:-	5730422L01	A630086K18	similar to HYPOTHETICAL 13.7 KDA PROTEIN [Homo sapiens]
Human	chr7:137940007-137980101:+	BG401569	-	-
Human	chr7:137848294-137940181:-	AF245517	AF245517	Homo sapiens vacuolar proton pump 116 kDa accessory subunit (ATP6N1B) mRNA, complete cds, alternatively spliced.
Mouse	chr6:38038736-38045290:+	D630002J15	D630002J15	hypothetical protein
Mouse	chr6:37912979-38126859:-	BC046979	AK128892	Mus musculus cDNA fis, clone TRACH2004887, moderately similar to Homo sapiens ATPase, H+ transporting, lysosomal V0 subunit a isoform 4 (ATP6V0A4). CDS=277..1722
Human	chr7:138177416-138178875:+	BG829700	-	-
Human	chr7:138167707-138178030:-	BC008842	BC008842	Homo sapiens similar to RIKEN cDNA 1200014N16 gene, mRNA (cDNA clone MGC:14289 IMAGE:4136104), complete cds.
Mouse	chr6:38229796-38234552:+	9830147F01	9830147F01	unclassifiable
Mouse	chr6:38218155-38230020:-	D030064C04	B130055L09	hypothetical protein
Human	chr7:139825873-139876047:+	CR610566	CR610711	full-length cDNA clone CS0DF010YK23 of Fetal brain of Homo sapiens (human).
Human	chr7:139848304-139850124:-	AK129584	AK129584	Homo sapiens cDNA FLJ26073 fis, clone RCT01314.
Mouse	chr6:39501774-39536647:+	4732401K03	4732401K03	aarF domain containing kinase 2
Mouse	chr6:39511548-39520689:-	K630055D03	-	-
Human	chr7:147832879-147935852:+	BP230087	AK096163	Homo sapiens cDNA FLJ38844 fis, clone MESAN2003662, highly similar to CULLIN.
Human	chr7:147832007-147833905:-	CR598519	CR598519	full-length cDNA clone CS0DK009YG14 of HeLa cells Cot 25-normalized of Homo sapiens (human).
Mouse	chr6:47438236-47511704:+	D030060P08	BC029260	Mus musculus cullin 1, mRNA (cDNA clone MGC:35976 IMAGE:2812400), complete cds. CDS=322..2652
Mouse	chr6:47436326-47439897:-	A630042M01	A930035D04	hypothetical protein
Human	chr7:150125753-150149343:+	M93718	BC063294	Homo sapiens nitric oxide synthase 3 (endothelial cell), mRNA (cDNA clone MGC:71636 IMAGE:30340813), complete cds.
Human	chr7:150146945-150159234:-	AY316116	AY515311	Homo sapiens sONE (NOS3AS) mRNA, complete cds.
Mouse	chr5:22762836-22782816:+	F930031B19	6030422B05	nitric oxide synthase 3, endothelial cell
Mouse	chr5:22764220-22781956:-	A330072D07	A330072D07	unclassifiable
Human	chr7:150149347-150150385:+	BX098174	-	-
Human	chr7:150146945-150159234:-	AK027791	AY515311	Homo sapiens sONE (NOS3AS) mRNA, complete cds.
Mouse	chr5:22762836-22782816:+	6030422B05	6030422B05	nitric oxide synthase 3, endothelial cell
Mouse	chr5:22782699-22786308:-	2310065A03	2310065A03	unclassifiable
Human	chr7:150367200-150373747:+	AY358407	AK056331	Homo sapiens cDNA FLJ31769 fis, clone NT2RI2007956.
Human	chr7:150373498-150411898:-	AI291485	U66619	Human SWI/SNF complex 60 KDa subunit (BAF60c) mRNA, complete cds.
Mouse	chr5:22987037-22998110:+	A430083F20	A430083F20	Hypothetical Prokaryotic membrane lipoprotein lipid attachment site containing protein homolog [Mus musculus]
Mouse	chr5:22991121-23024725:-	F930017E24	F930017E24	SWI/SNF related, matrix associated, actin dependent regulator of chromatin, subfamily d, member 3
Human	chr7:150375103-150375774:+	BM715580	-	-
Human	chr7:150373498-150411898:-	BC002628	U66619	Human SWI/SNF complex 60 KDa subunit (BAF60c) mRNA, complete cds.
Mouse	chr5:22987037-22998110:+	A430083F20	A430083F20	Hypothetical Prokaryotic membrane lipoprotein lipid attachment site containing protein homolog [Mus musculus]
Mouse	chr5:22991121-23024725:-	F930017E24	F930017E24	SWI/SNF related, matrix associated, actin dependent regulator of chromatin, subfamily d, member 3

Human	chr7:150476506-150513195:+	AF300717	AF300717	Homo sapiens NUB1 (NUB1) mRNA, complete cds.
Human	chr7:150500103-150503337:-	AK055458	AK055458	Homo sapiens cDNA FLJ30896 fis, clone FEBRA2005458.
Mouse	chr5:23086480-23111327:+	D930019L13	D930019L13	NY-REN-18 antigen
Mouse	chr5:23097853-23131464:-	4931404D21	BC059069	Mus musculus cDNA clone IMAGE:6825904, partial cds.
Human	chr7:150476506-150513195:+	AF300717	AF300717	Homo sapiens NUB1 (NUB1) mRNA, complete cds.
Human	chr7:150512605-150515152:-	AK127717	AK127717	Homo sapiens cDNA FLJ45817 fis, clone NT2RP7020343.
Mouse	chr5:23086480-23111327:+	D930019L13	D930019L13	NY-REN-18 antigen
Mouse	chr5:23110379-23111405:-	D430019G13	D430019G13	unclassifiable
Human	chr7:155932829-155969300:+	AL136874	AL136874	Homo sapiens mRNA; cDNA DKFZp434C135 (from clone DKFZp434C135); complete cds.
Human	chr7:155961116-156185386:-	AA262100	AF107454	Homo sapiens clone cD622 mRNA sequence.
Mouse	chr5:27594963-27624492:+	1700026E24	2700025B22	limb region 2
Mouse	chr5:27622756-27625918:-	C630011O12	C630011O12	unclassifiable
Human	chr7:155932829-155969300:+	CR533513	AL136874	Homo sapiens mRNA; cDNA DKFZp434C135 (from clone DKFZp434C135); complete cds.
Human	chr7:155962211-155968352:-	BU154247	AK126189	Homo sapiens cDNA FLJ44201 fis, clone THYMU3000841.
Mouse	chr5:27594963-27624492:+	1700026E24	2700025B22	limb region 2
Mouse	chr5:27622756-27625918:-	C630011O12	C630011O12	unclassifiable
Human	chr8:9450832-9677268:+	BC023578	AF082556	Homo sapiens TRF1-interacting ankyrin-related ADP-ribose polymerase mRNA, complete cds.
Human	chr8:9450066-9451659:-	CR590086	CR590086	full-length cDNA clone CS0DF038YF19 of Fetal brain of Homo sapiens (human).
Mouse	chr8:33785888-33925119:-	B430206N15	B430206N15	TRF1-INTERACTING ANKYRIN-RELATED ADP-RIBOSE POLYMERASE homolog [Homo sapiens]
Mouse	chr8:33924481-33927416:+	4930554K12	4930554K12	hypothetical protein
Human	chr8:17148777-17204307:+	AK090607	AL834189	Homo sapiens mRNA; cDNA DKFZp762I185 (from clone DKFZp762I185).
Human	chr8:17198657-17315207:-	AK123201	CR749240	Homo sapiens mRNA; cDNA DKFZp781E194 (from clone DKFZp781E194).
Mouse	chr8:39625724-39664915:+	2210018P21	F830219O21	hypothetical Modifier of rudimentary (Mod/Proline-rich region profile/Ubiquitin-conjugating enzymes containing protein
Mouse	chr8:39662020-39749359:-	C330026C13	BC032254	Mus musculus myotubularin related protein 7, mRNA (cDNA clone MGC:40839 IMAGE:5368816), complete cds. CDS=74..1477
Human	chr8:22491737-22517608:+	BC012750	AY217349	Homo sapiens PDZ-LIM protein mRNA, complete cds.
Human	chr8:22516591-22518449:-	BC031867	BC031867	Homo sapiens, Similar to forkhead box L2, clone MGC:42588 IMAGE:4825132, mRNA, complete cds.
Mouse	chr14:61930361-61935308:-	3830406K03	3830406K03	hypothetical protein
Mouse	chr14:61929281-61934495:+	E130115M17	E130115M17	unclassifiable
Human	chr8:22518115-22534232:+	BX640952	BC065495	Homo sapiens p30 DBC protein, transcript variant 2, mRNA (cDNA clone MGC:74489 IMAGE:5496068), complete cds.
Human	chr8:22516591-22518449:-	BC031867	BC031867	Homo sapiens, Similar to forkhead box L2, clone MGC:42588 IMAGE:4825132, mRNA, complete cds.
Mouse	chr14:61913993-61929767:-	BC021475	BC021475	Mus musculus RIKEN cDNA 2610301G19 gene, mRNA (cDNA clone MGC:36426 IMAGE:5343456), complete cds. CDS=232..3000
Mouse	chr14:61929281-61934495:+	E130115M17	E130115M17	unclassifiable
Human	chr8:24827179-24832516:+	BC071752	BC071752	Homo sapiens neurofilament 3 (150kDa medium), mRNA (cDNA clone IMAGE:6464368), partial cds.
Human	chr8:24827215-24828266:-	BX431039	-	-
Mouse	chr14:60071503-60113803:-	B230114B02	A730009A11	neurofilament, medium polypeptide
Mouse	chr14:60112604-60120284:+	K430312O12	-	-
Human	chr8:24868911-24870541:+	H22722	-	-
Human	chr8:24864385-24870164:-	AK057731	AK075003	Homo sapiens cDNA FLJ90522 fis, clone NT2RP4000108, highly similar to Human gene for neurofilament subunit NF-L.
Mouse	chr14:60071503-60113803:-	A730009A11	A730009A11	neurofilament, medium polypeptide
Mouse	chr14:60072729-60078008:+	7120495C04	7120495C04	neurofilament, light polypeptide
Human	chr8:33462227-33478342:+	BC039740	BC039740	Homo sapiens, similar to RNA binding protein, clone IMAGE:6066906, mRNA.
Human	chr8:33451343-33490200:-	AK026916	BC066935	Homo sapiens hypothetical protein FLJ23263, mRNA (cDNA clone MGC:87253 IMAGE:4839169), complete cds.
Mouse	chr8:30095823-30105355:-	BC013079	BC013079	Mus musculus RIKEN cDNA 2600016B03 gene, mRNA (cDNA clone MGC:6379 IMAGE:3499754), complete cds. CDS=61..933
Mouse	chr8:30086422-30101202:+	A830073O18	A830073O18	Hypothetical ARM repeat structure containing protein
Human	chr8:37760867-37820662:+	AB040964	AK027296	Homo sapiens cDNA FLJ14390 fis, clone HEMBA1003071, weakly similar to INSULIN-LIKE GROWTH FACTOR BINDING PROTEIN COMPLEX ACID LABILE CHAIN PRECURSOR (ALS).
Human	chr8:37820412-37826575:-	CR604537	CR604537	full-length cDNA clone CS0DC024YJ19 of Neuroblastoma Cot 25-normalized of Homo sapiens (human).
Mouse	chr8:25873431-25911213:+	9530074E10	AF378759	Mus musculus tumor endothelial marker 5 precursor (Tem5) mRNA, complete cds. CDS=127..4116
Mouse	chr8:25911124-25916457:-	5730512K07	5730512K07	BRF2, subunit of RNA polymerase III transcription initiation factor, BRF1-like
Human	chr8:38082175-38120751:+	BE349850	CR602034	full-length cDNA clone CS0DJ012YJ24 of T cells (Jurkat cell line) Cot 10-normalized of Homo sapiens (human).
Human	chr8:38120322-38127767:-	AF035277	CR616953	full-length cDNA clone CS0DD008YM18 of Neuroblastoma Cot 50-normalized of Homo sapiens (human).
Mouse	chr8:24589994-24626687:-	2310021I05	I730043C01	ash2 (absent, small, or homeotic)-like (Drosophila)
Mouse	chr8:24584016-24593445:+	BC060970	E330028H19	steroidogenic acute regulatory protein

Human	chr8:38117083-38119116:+	W85900	-	-
Human	chr8:38118422-38118954:-	AV703733	-	-
Mouse	chr8:24589994-24626687:-	2310021I05	I730043C01	ash2 (absent, small, or homeotic)-like (Drosophila)
Mouse	chr8:24584016-24593445:+	BC060970	E330028H19	steroidogenic acute regulatory protein
Human	chr8:41638148-41642241:+	BF435750	-	-
Human	chr8:41629896-41774297:-	BX647936	X16609	Human mRNA for ankyrin (variant 2.1).
Mouse	chr8:21901344-21922482:-	A630066O12	A630066O12	ankyrin 1, erythroid
Mouse	chr8:21729510-21923258:+	M5C1005P16	M5C1005P16	ankyrin 1, erythroid
Human	chr8:42515455-42527918:+	CR616059	BC013035	Homo sapiens hypothetical protein BC013035, mRNA (cDNA clone MGC:4729 IMAGE:3535608), complete cds.
Human	chr8:42393137-42516225:-	BC028600	BC028600	Homo sapiens solute carrier family 20 (phosphate transporter), member 2, mRNA (cDNA clone MGC:33076 IMAGE:4820601), complete cds.
Mouse	chr8:21213813-21229234:-	5430408M21	5430408M21	Similar to fasciculation and elongation protein zeta 2 (zygin II) homolog [Homo sapiens]
Mouse	chr8:21229052-21324378:+	F630215A16	BC046510	Mus musculus solute carrier family 20, member 2, mRNA (cDNA clone MGC:51401 IMAGE:4035182), complete cds. CDS=415..2385
Human	chr8:48336010-48841205:+	AK026929	D63480	Human mRNA for KIAA0146 gene, partial cds.
Human	chr8:48810749-48814201:-	BF108849	M83667	Human NF-IL6-beta protein mRNA, complete cds.
Mouse	chr16:15662846-15920446:-	BC046558	I530027A02	Hypothetical aminoacyl-transfer RNA synthetases class-II containing protein
Mouse	chr16:15660552-15664653:+	G530013D06	G530013D06	CCAAT/enhancer binding protein (C/EBP), delta
Human	chr8:56954918-57087146:+	BC059394	BC059394	Homo sapiens v-yes-1 Yamaguchi sarcoma viral related oncogene homolog, mRNA (cDNA clone IMAGE:30332044), partial cds.
Human	chr8:56955047-57085685:-	AW169914	-	-
Mouse	chr4:3605262-3718760:+	I830119M13	1810073A02	Yamaguchi sarcoma viral (v-yes-1) oncogene homolog
Mouse	chr4:3604506-3606140:-	B930046N17	-	-
Human	chr8:62363085-62576758:+	BC048992	BC048992	Homo sapiens, clone IMAGE:5284856, mRNA.
Human	chr8:62575549-62789751:-	AF339775	U03109	Human aspartyl beta-hydroxylase mRNA, complete cds.
Mouse	chr4:9196440-9378838:+	BC062923	BC062923	Mus musculus cDNA clone MGC:73431 IMAGE:6848546, complete cds. CDS=281..1345
Mouse	chr4:9372862-9596219:-	AF289487	AF289487	Mus musculus aspartyl beta-hydroxylase 6.6 kb transcript (Asph) mRNA, complete cds; alternatively spliced. CDS=175..2394
Human	chr8:72902956-73193427:+	AK123315	BX647470	Homo sapiens mRNA; cDNA DKFZp686E16147 (from clone DKFZp686E16147).
Human	chr8:72916331-72919257:-	BC067827	BC067827	Homo sapiens musculin (activated B-cell factor-1), mRNA (cDNA clone MGC:87363 IMAGE:30336424), complete cds.
Mouse	chr1:14915378-14936855:+	E130016A22	A630063H21	unclassifiable
Mouse	chr1:14913270-14915938:-	A530004K05	AF087035	Mus musculus musculin mRNA, complete cds. CDS=219..824
Human	chr8:74141616-74142166:+	AI458328	-	-
Human	chr8:74140004-74198877:-	AK097340	AK097340	Homo sapiens cDNA FLJ40021 fis, clone STOMA2006904.
Mouse	chr1:16015102-16018270:+	B930031D20	B930031D20	unclassifiable
Mouse	chr1:16013993-16054505:-	9530008A11	9530008A11	RPE-spondin (Fragment) homolog [Homo sapiens]
Human	chr8:86276637-86317019:+	U31556	U31556	Human transcription factor E2F-5 mRNA, complete cds.
Human	chr8:86313539-86319902:-	BC055092	BC055092	Homo sapiens cDNA clone IMAGE:4737427, partial cds.
Mouse	chr3:14659706-14687566:+	BC003220	BC003220	Mus musculus E2F transcription factor 5, mRNA (cDNA clone MGC:6043 IMAGE:3482306), complete cds. CDS=150..1157
Mouse	chr3:14687542-14692533:-	AL023036	1810022K09	unclassifiable
Human	chr8:86320059-86548525:+	AK096880	AK093753	Homo sapiens cDNA FLJ36434 fis, clone THYMU2012002.
Human	chr8:86541370-86562535:-	BG215466	-	-
Mouse	chr3:14919204-14928263:+	1110013P06	1110013P06	carbonic anhydrase 3
Mouse	chr3:14904877-14922642:-	A530078D15	-	-
Human	chr8:87424076-87559765:+	AL050082	BC036065	Homo sapiens, WW domain-containing protein 1, clone MGC:33686 IMAGE:5296005, mRNA, complete cds.
Human	chr8:87548461-87595693:-	AL582101	AK000672	Homo sapiens cDNA FLJ20665 fis, clone KAIA713, highly similar to AF151848 Homo sapiens CGI-90 protein mRNA.
Mouse	chr4:19533714-19636137:-	B730024E11	6430508G12	similar to DJ468O1.1 (ATROPHIN 1 INTERACTING PROTEIN 4 (AIP4)) (FRAGMENT) [Homo sapiens]
Mouse	chr4:19536516-19538587:+	D930011O12	-	-
Human	chr8:91872954-92040812:+	AF414126	AF414126	Homo sapiens EF-hand calcium binding protein 1 (EFCBP1) mRNA, complete cds.
Human	chr8:92037343-92066661:-	BM667874	-	-
Mouse	chr4:14878568-15076231:-	AY278200	AY278200	Mus musculus EF-hand calcium binding protein 1 (Efcbp1) mRNA, complete cds. CDS=199..1257
Mouse	chr4:14856964-14879354:+	1700003H21	1700003H21	unclassifiable
Human	chr8:95904681-95975678:+	AB161944	AB161944	Homo sapiens mRNA for KAONASHI protein 1, complete cds.
Human	chr8:95961628-95978082:-	AF091433	AF091433	Homo sapiens cyclin E2 mRNA, complete cds.
Mouse	chr4:11125481-11180585:-	C430018J16	C430018J16	hypothetical protein
Mouse	chr4:11117249-11131102:+	5930406G12	K230305H19	cyclin E2

Human	chr8:97316604-97319438:+	AI018197	-	-
Human	chr8:97311910-97317038:-	BC005230	BC005230	Homo sapiens, ubiquinol-cytochrome c reductase binding protein, clone MGC:12253 IMAGE:3961169, mRNA, complete cds.
Mouse	chr13:63912705-63916957:+	B020047E20	A530092F16	hypothetical protein
Mouse	chr13:63908382-63913169:-	2210415M14	2210415M14	UBIQUINOL-CYTOCHROME C REDUCTASE COMPLEX 14 KDA PROTEIN (EC 1.10.2.2) (COMPLEX III SUBUNIT VI) homolog [Mus musculus]
Human	chr8:102573836-102749157:+	BP356683	BC069638	Homo sapiens transcription factor CP2-like 3, mRNA (cDNA clone MGC:97170 IMAGE:7262413), complete cds.
Human	chr8:102572661-102573966:-	CB996838	-	-
Mouse	chr15:37291572-37424140:+	0610015A08	BC055035	Mus musculus grainyhead like 2 (Drosophila), mRNA (cDNA clone MGC:62708 IMAGE:6306106), complete cds. CDS=141..2018
Mouse	chr15:37290348-37291857:-	1600010A02	1600010A02	unclassifiable
Human	chr8:104379843-104414270:+	BX640609	BX640609	Homo sapiens mRNA; cDNA DKFZp686I13174 (from clone DKFZp686I13174); complete cds.
Human	chr8:104247048-104496660:-	AK125733	AK125733	Homo sapiens cDNA FLJ43745 fis, clone TESTI2019648.
Mouse	chr15:38978052-39010780:+	M130006E10	BC026150	Mus musculus frizzled homolog 6 (Drosophila), mRNA (cDNA clone MGC:14004 IMAGE:3983985), complete cds. CDS=309..2438
Mouse	chr15:38975304-38978273:-	E230021H07	E230021H07	unclassifiable
Human	chr8:110411408-110427365:+	CN389990	AK095651	Homo sapiens cDNA FLJ38332 fis, clone FCBBF3025528.
Human	chr8:110322324-110415790:-	AF283301	BC043406	Homo sapiens, chronic myelogenous leukemia tumor antigen 66, clone MGC:50930 IMAGE:6059975, mRNA, complete cds.
Mouse	chr15:44450628-44460797:+	1810057B09	4932441N08	similar to E(Y)2 HOMOLOG (DC6) (DC6 PROTEIN) [Homo sapiens]
Mouse	chr15:44397768-44450848:-	AF521132	5730516H14	CHRONIC MYELOGENOUS LEUKEMIA TUMOR ANTIGEN 66 homolog [Homo sapiens]
Human	chr8:120955117-121132342:+	AL136678	AK023916	Homo sapiens cDNA FLJ13854 fis, clone THYRO1000952.
Human	chr8:120948840-120955909:-	AK123921	AK123921	Homo sapiens cDNA FLJ41927 fis, clone PERIC2003919.
Mouse	chr15:55290542-55433202:+	G930020B17	B020032E21	DEP domain containing 6
Mouse	chr15:55288482-55291943:-	C130071N01	C130071N01	hypothetical protein
Human	chr8:145177436-145178536:+	BX433886	-	-
Human	chr8:145178155-145186959:-	BC041576	AB122018	Homo sapiens 5-OPase mRNA for 5-oxo-L-prolinase, complete cds.
Mouse	chr15:76617971-76620136:+	1700010G02	1700010G02	inferred: 5-OXOPROLINASE (EC 3.5.2.9) (5-OXO-L-PROLINASE) (PYROGLUTAMASE) (5-OPASE). [Rat] {Rattus norvegicus}
Mouse	chr15:76619799-76630586:-	D330022L02	BC034522	Mus musculus 5-oxoprolinase (ATP-hydrolysing), mRNA (cDNA clone MGC:28663 IMAGE:4236444), complete cds. CDS=72..3938
Human	chr8:145231288-145234509:+	BC031273	BC031273	Homo sapiens, homolog of yeast MAF1, clone MGC:39758 IMAGE:5288176, mRNA, complete cds.
Human	chr8:145225523-145235015:-	BG723928	AB052765	Homo sapiens hRBCKL1-beta mRNA for protein kinase C-interacting protein RBCC like 1-beta, complete cds.
Mouse	chr15:76675421-76678505:+	BC016260	BC016260	Mus musculus RIKEN cDNA 1110068E11 gene, mRNA (cDNA clone MGC:28902 IMAGE:4914167), complete cds. CDS=405..1181
Mouse	chr15:76671166-76675862:-	I020025I07	BC055758	Mus musculus RIKEN cDNA 0610041B22 gene, mRNA (cDNA clone MGC:67162 IMAGE:6415354), complete cds. CDS=433..1350
Human	chr8:145486047-145509197:+	AF299094	M64673	Human heat shock factor 1 (TCF5) mRNA, complete cds.
Human	chr8:145508642-145521381:-	CN292150	BC015762	Homo sapiens, clone MGC:23162 IMAGE:4863905, mRNA, complete cds.
Mouse	chr15:76802950-76827568:+	C230058A10	C230058A10	heat shock factor 1
Mouse	chr15:76826544-76837620:-	M230038M19	BC003717	Mus musculus diacylglycerol O-acyltransferase 1, mRNA (cDNA clone MGC:5740 IMAGE:3486724), complete cds. CDS=38..1534
Human	chr8:145662532-145670310:+	BC015171	BC017311	Homo sapiens likely ortholog of mouse kinesin family member C2, mRNA (cDNA clone MGC:29716 IMAGE:5090622), complete cds.
Human	chr8:145669923-145672526:-	BC065377	AF076292	Homo sapiens TGF-beta/activin signal transducer FAST-1p (FAST1) mRNA, complete cds.
Mouse	chr15:76985752-76994538:+	D49545	G630012I08	kinesin family member C2
Mouse	chr15:76994173-76996231:-	BM210465	AF110506	Mus musculus forkhead activin signal transducer 2 (Fast2) mRNA, complete cds. CDS=1..1206
Human	chr8:145714171-145721370:+	D25216	D25216	Homo sapiens KIAA0014 mRNA, complete cds.
Human	chr8:145718569-145725324:-	CR607461	CR607461	full-length cDNA clone CS0DF013YE11 of Fetal brain of Homo sapiens (human).
Mouse	chr15:77042059-77044344:+	A730001P22	A730001P22	unclassifiable
Mouse	chr15:77041925-77050495:-	C230002N12	6430402H13	
Human	chr9:2611786-2644762:+	L20470	L20470	Human very low density lipoprotein receptor mRNA, complete cds.
Human	chr9:2525652-2612767:-	BC004474	BC004474	Homo sapiens cDNA clone IMAGE:3928143, partial cds.
Mouse	chr19:26493119-26530723:+	9330117M18	9330117M18	very low density lipoprotein receptor
Mouse	chr19:26378271-26494360:-	BE863986	-	-
Human	chr9:19322006-19364427:+	CR627367	CR627367	Homo sapiens mRNA; cDNA DKFZp686I09113 (from clone DKFZp686I09113).
Human	chr9:19361383-19363307:-	F07163	-	-
Mouse	chr4:85023005-85125053:+	BC040249	F630209B03	weakly similar to C-MYC promoter-binding protein IRLB [Homo sapiens]
Mouse	chr4:85123131-85125427:-	A230054N22	A230054N22	unclassifiable
Human	chr9:19322006-19364427:+	CR627367	CR627367	Homo sapiens mRNA; cDNA DKFZp686I09113 (from clone DKFZp686I09113).
Human	chr9:19363571-19365007:-	N20552	-	-
Mouse	chr4:85023005-85125053:+	BC040249	F630209B03	weakly similar to C-MYC promoter-binding protein IRLB [Homo sapiens]
Mouse	chr4:85123131-85125427:-	A230054N22	A230054N22	unclassifiable

Human	chr9:32540631-32558622:+	CR604036	CR604036	full-length cDNA clone CS0DI064YO22 of Placenta Cot 25-normalized of Homo sapiens (human).
Human	chr9:32445300-32542605:-	BC060884	BC070029	Homo sapiens cDNA clone IMAGE:5271892, containing frame-shift errors.
Mouse	chr4:40360986-40362357:+	5330408M09	2010003O02	RIKEN cDNA 2010003O02 gene
Mouse	chr4:40351018-40361267:-	BC040797	AB104865	Mus musculus Topors mRNA for topoisomerase 1-binding RING finger protein, complete cds. CDS=151..3252
Human	chr9:34169003-34242521:+	AK074745	AK074812	Homo sapiens cDNA FLJ90331 fis, clone NT2RP2002015.
Human	chr9:34242378-34249703:-	AK125872	AK125872	Homo sapiens cDNA FLJ43884 fis, clone TESTI4009160.
Mouse	chr4:41440399-41481928:+	D130037G03	2310033J24	ubiquitin-associated protein
Mouse	chr4:41481022-41556310:-	BU756676	D430019P19	Kinesin superfamily protein KIF24
Human	chr9:34655493-34673031:+	AK125920	AK125920	Homo sapiens cDNA FLJ43932 fis, clone TESTI4013675.
Human	chr9:34651880-34656109:-	BC066358	BC037171	Homo sapiens, clone IMAGE:5248278, mRNA, partial cds.
Mouse	chr4:41865453-41902563:+	4932410C13	4932410C13	hypothetical protein
Mouse	chr4:41860710-41981299:-	K630150M08	1700008B15	unclassifiable
Human	chr9:34655493-34673031:+	AK125920	AK125920	Homo sapiens cDNA FLJ43932 fis, clone TESTI4013675.
Human	chr9:34651880-34656109:-	BC066358	BC037171	Homo sapiens, clone IMAGE:5248278, mRNA, partial cds.
Mouse	chr4:41865103-42189845:+	BG100702	1700031K17	unclassifiable
Mouse	chr4:41860710-41981299:-	CN839002	1700008B15	unclassifiable
Human	chr9:35480114-35551895:+	BC029647	AB002373	Homo sapiens mRNA for KIAA0375 protein, partial cds.
Human	chr9:35551827-35553867:-	AA441812	-	-
Mouse	chr4:42627072-42672185:+	G830026E07	G830026E07	RUN and SH3 domain containing 2
Mouse	chr4:42672113-42674227:-	4833436C18	4833436C18	hypothetical protein
Human	chr9:35595281-35600326:+	BC011799	BC067130	Homo sapiens testis-specific kinase 1, mRNA (cDNA clone MGC:70770 IMAGE:6153652), complete cds.
Human	chr9:35599950-35636833:-	M54992	M54992	Human B cell differentiation antigen mRNA, complete cds.
Mouse	chr4:42687032-42693168:+	G630012H19	AB003494	Mus musculus mRNA for testis-specific protein kinase 1, complete cds. CDS=1497..3380
Mouse	chr4:42692817-42699721:-	G430088K09	5830446P09	CD72 antigen
Human	chr9:35663853-35671156:+	BC014950	BC014950	Homo sapiens, carbonic anhydrase IX, clone MGC:22967 IMAGE:4865275, mRNA, complete cds.
Human	chr9:35648872-35665863:-	BC038673	BC038673	Homo sapiens, Similar to hypothetical protein FLJ14642, clone IMAGE:5266209, mRNA.
Mouse	chr4:42752117-42758822:+	AJ245857	AJ245857	Mus musculus mRNA for carbonic anhydrase (MN/CA9 gene). CDS=33..1346
Mouse	chr4:42741232-42753840:-	F630119O22	E430002C01	hypothetical protein
Human	chr9:35722083-35728885:+	AF211848	AF211848	Homo sapiens cAMP responsive element binding protein 3 (CREB3) mRNA, complete cds.
Human	chr9:35687334-35722392:-	AB028950	AB028950	Homo sapiens mRNA for KIAA1027 protein, partial cds.
Mouse	chr4:42807425-42812763:+	AU079989	G430114C06	cAMP responsive element binding protein 3
Mouse	chr4:42776605-42807676:-	1700018G09	X56123	Mouse mRNA for talin. CDS=160..7785
Human	chr9:35722083-35728885:+	AF211848	AF211848	Homo sapiens cAMP responsive element binding protein 3 (CREB3) mRNA, complete cds.
Human	chr9:35726859-35739225:-	BC011363	AJ309567	Homo sapiens mRNA for bile acid beta-glucosidase.
Mouse	chr4:42807425-42812763:+	G430114C06	G430114C06	cAMP responsive element binding protein 3
Mouse	chr4:42812021-42823986:-	BC034105	F930037M19	glucosidase beta 2
Human	chr9:35739277-35745220:+	BC001725	BC001725	Homo sapiens, KIAA0258 gene product, clone MGC:755 IMAGE:3533857, mRNA, complete cds.
Human	chr9:35742987-35747068:-	BC071800	BC071800	Homo sapiens cDNA clone IMAGE:4666428, partial cds.
Mouse	chr4:42823808-42832580:+	AK129103	F630019D11	hypothetical protein
Mouse	chr4:42828395-42829587:-	CA481548	-	-
Human	chr9:37770000-37770824:+	CN362885	-	-
Human	chr9:37755998-37791434:-	AF151860	BC002437	Homo sapiens exosome component Rrp40, mRNA (cDNA clone MGC:723 IMAGE:3346075), complete cds.
Mouse	chr4:44542248-44562178:+	G270084C23	5330415K23	hypothetical EF-hand containing protein
Mouse	chr4:44561705-44565709:-	2310005D06	BC023669	Mus musculus RIKEN cDNA 2310005D06 gene, mRNA (cDNA clone IMAGE:5012335), partial cds.
Human	chr9:68380360-68384224:+	AK126504	AK126504	Homo sapiens cDNA FLJ44540 fis, clone UTERU3005230.
Human	chr9:68381052-68385337:-	BC029780	BC029780	Homo sapiens, LOC169693, clone MGC:34760 IMAGE:5185747, mRNA, complete cds.
Mouse	chr19:23953064-23954232:-	D330023H14	D330023H14	unclassifiable
Mouse	chr19:23948797-23954468:+	F430103N22	F430103N22	hypothetical protein
Human	chr9:70257653-70259129:+	BM705037	-	-
Human	chr9:70229057-70259094:-	D31716	D31716	Human mRNA for GC box bindig protein, complete cds.
Mouse	chr19:22412702-22414861:-	E230024B12	E230024B12	hypothetical protein
Mouse	chr19:22413416-22441303:+	2410048J15	2410048J15	basic transcription element binding protein 1

Human	chr9:76238354-76243640:+	BG424218	-	-
Human	chr9:76229987-76243281:-	AK002011	BC007069	Homo sapiens, hypothetical protein FLJ11149, clone MGC:12524 IMAGE:3997678, mRNA, complete cds.
Mouse	chr19:16672987-16673731:-	A630039F22	A630039F22	hypothetical protein
Mouse	chr19:16673254-16681160:+	0610038L10	0610038L10	hypothetical Riboflavin kinase / FAD synthetase containing protein
Human	chr9:77021915-77261954:+	AF337532	AF337532	Homo sapiens chorea-acanthocytosis (CHAC) mRNA, complete cds.
Human	chr9:77021226-77022625:-	BI828209	-	-
Mouse	chr19:16027911-16058701:-	F530013G14	-	-
Mouse	chr19:16058317-16059780:+	4930516E05	4930516E05	hypothetical Gram-positive cocci surface protein 'anchoring' hexapeptide containing protein
Human	chr9:83674073-83682052:+	AI205792	-	-
Human	chr9:83681070-83765896:-	AL133654	AY237536	Homo sapiens KIF27A mRNA, complete cds, alternatively spliced.
Mouse	chr13:57394678-57412641:+	4930414F17	4930414F17	unclassifiable
Mouse	chr13:57400998-57457692:-	6720471I09	6720471I09	similar to hypothetical protein DKFZp434D0917.1 (fragments) [Homo sapiens]
Human	chr9:92167296-92457046:+	AK092603	AK092603	Homo sapiens cDNA FLJ35284 fis, clone PROST2007871.
Human	chr9:92454579-92518556:-	AK024267	BC026154	Homo sapiens chromosome 9 open reading frame 12, mRNA (cDNA clone MGC:26027 IMAGE:4839268), complete cds.
Mouse	chr13:48962903-49149015:-	1700022C02	1700022C02	RIKEN cDNA 1700022C02 gene
Mouse	chr13:48920103-48963453:+	9930116C11	I530016B18	Hypothetical protein FLJ13163 (C9orf12) (Chromosome 9 open reading frame 12) (Inositol 1,3,4,5,6-pentakisphosphate 2-kinase) homolog [Homo sapiens]
Human	chr9:93293495-93408238:+	D80005	BC075701	Homo sapiens cDNA clone IMAGE:30344681, containing frame-shift errors.
Human	chr9:93406499-93407578:-	BQ432329	-	-
Mouse	chr13:48376014-48464220:-	9430083N20	BC042582	Mus musculus cDNA sequence BC010304, mRNA (cDNA clone IMAGE:4952796), containing frame-shift errors.
Mouse	chr13:48376023-48377156:+	CD742368	-	-
Human	chr9:95717499-95856408:+	AK129624	BC035183	Homo sapiens, clone IMAGE:5266310, mRNA.
Human	chr9:95615764-95717816:-	AF194971	AF194971	Homo sapiens unknown (NAG12) mRNA, complete cds.
Mouse	chr13:62149019-62234241:+	C330042K07	C630005J05	PUTATIVE REPAIR AND RECOMBINATION HELICASE RAD26L (FRAGMENT) homolog [Mus musculus]
Mouse	chr13:62147771-62149253:-	BE655364	C030010C08	unclassifiable
Human	chr9:97343476-97443585:+	AK095748	BC002660	Homo sapiens tropomodulin 1, mRNA (cDNA clone MGC:3643 IMAGE:3610014), complete cds.
Human	chr9:97441913-97475406:-	BC022958	AK128615	Homo sapiens cDNA FLJ46774 fis, clone TRACH3026303.
Mouse	chr4:45284028-45361125:+	9430057C23	S76831	Tmod=tropomodulin [mice, myogenic C2 cells, mRNA, 1572 nt]. CDS=65..1144
Mouse	chr4:45359839-45383802:-	3010020C06	3010020C06	CDNA FLJ31891 FIS, CLONE NT2RP7003304, WEAKLY SIMILAR TO YCEA PROTEIN HOMOLOG YBFQ homolog [Homo sapiens]
Human	chr9:97475070-97518839:+	BG827246	D32002	Human mRNA for nuclear cap binding protein, complete cds.
Human	chr9:97441913-97475406:-	AK128615	AK128615	Homo sapiens cDNA FLJ46774 fis, clone TRACH3026303.
Mouse	chr4:45383601-45418046:+	6030427C23	5730600J02	80 kDa nuclear cap binding protein (NCBP 80 kDa subunit) (CBP80) homolog [Homo sapiens]
Mouse	chr4:45359839-45383802:-	C330044H19	3010020C06	CDNA FLJ31891 FIS, CLONE NT2RP7003304, WEAKLY SIMILAR TO YCEA PROTEIN HOMOLOG YBFQ homolog [Homo sapiens]
Human	chr9:99941057-100144432:+	BC063847	BC063847	Homo sapiens inversin, mRNA (cDNA clone IMAGE:6503936), partial cds.
Human	chr9:100143912-100194814:-	AJ420436	AB060968	Homo sapiens nbla10363 mRNA, complete cds.
Mouse	chr4:47524893-47677047:+	AF034860	AF034860	Mus musculus Inv (inv) mRNA, complete cds. CDS=183..3371
Mouse	chr4:47674608-47718556:-	BC006867	C430021H21	Nbla10363 protein homolog [Homo sapiens]
Human	chr9:112992792-113005976:+	AK057903	AX810714	Sequence 38 from Patent EP1333092.
Human	chr9:113005478-113007344:-	Z41708	-	-
Mouse	chr4:60590065-60625915:+	2010208F24	2010208F24	solute carrier family 31, member 2
Mouse	chr4:60625865-60627177:-	5730427C19	5730427C19	unclassifiable
Human	chr9:122106489-122173173:+	BI754479	BC013049	Homo sapiens, Similar to RIKEN cDNA 2400002D02 gene, clone MGC:17776 IMAGE:3882448, mRNA, complete cds.
Human	chr9:122079457-122106687:-	AK057676	AK057676	Homo sapiens cDNA FLJ33114 fis, clone TRACH2001289, weakly similar to SEX-LETHAL PROTEIN HOMOLOG.
Mouse	chr2:36096380-36150638:+	AI930475	6430528J02	hypothetical Ribosome recycling factor containing protein
Mouse	chr2:36076069-36096764:-	6430594K01	6430594K01	RNA binding motif protein 18
Human	chr9:123198003-123222168:+	AK126775	AK123000	Homo sapiens cDNA FLJ16786 fis, clone NT2RI3009524, weakly similar to Crumbs protein homolog 1 precursor.
Human	chr9:123221487-123771973:-	AK074151	BC009616	Homo sapiens KIAA1608, mRNA (cDNA clone IMAGE:3905823), partial cds.
Mouse	chr2:37736240-37759094:+	BC043114	BC043114	Mus musculus cDNA sequence BC043114, mRNA (cDNA clone IMAGE:6413034), partial cds. CDS=3..3629
Mouse	chr2:37758981-38247375:-	9830132A21	BC023016	Mus musculus RIKEN cDNA 6030446I19 gene, mRNA (cDNA clone IMAGE:5363934), complete cds. CDS=256..930
Human	chr9:123853592-123875134:+	AI563896	BC034458	Homo sapiens, clone IMAGE:5180630, mRNA.
Human	chr9:123873451-123874381:-	AA394287	-	-
Mouse	chr2:38299272-38329722:+	E130111G23	E130111G23	LIM-homeodomain protein MLHX2 (Lhx2) mRNA
Mouse	chr2:38328444-38328990:-	CA463275	-	-

Human	chr9:124196102-124201021:+	CR627469	CR627469	Homo sapiens mRNA; cDNA DKFZp547D176 (from clone DKFZp547D176).
Human	chr9:124195297-124257304:-	CR618251	D38048	Human mRNA for proteasome subunit z, complete cds.
Mouse	chr2:38550797-38553637:+	F630310A07	F630310A07	unclassifiable
Mouse	chr2:38548027-38603918:-	D83585	E430025I23	proteasome (prosome, macropain) subunit, beta type 7
Human	chr9:127454071-127537853:+	AF004563	AF004563	Homo sapiens hUNC18b alternatively-spliced mRNA, complete cds.
Human	chr9:127531931-127566706:-	CB162387	AK090922	Homo sapiens cDNA FLJ33603 fis, clone BRAMY2014387, weakly similar to PEPTIDYL-TRNA HYDROLASE (EC 3.1.1.29).
Mouse	chr2:32747593-32807230:-	1200015C19	BC031728	Mus musculus syntaxin binding protein 1, mRNA (cDNA clone MGC:30503 IMAGE:4480978), complete cds. CDS=74..1885
Mouse	chr2:32735781-32750197:+	BM231705	2210013M04	hypothetical Peptidyl-tRNA hydrolase containing protein
Human	chr9:127548822-127557835:+	AL833241	AK094948	Homo sapiens cDNA FLJ37629 fis, clone BRCOC2015824.
Human	chr9:127531931-127566706:-	CR611847	AK090922	Homo sapiens cDNA FLJ33603 fis, clone BRAMY2014387, weakly similar to PEPTIDYL-TRNA HYDROLASE (EC 3.1.1.29).
Mouse	chr2:32737364-32744419:-	1700019L03	1700019L03	hypothetical protein
Mouse	chr2:32735781-32750197:+	2210013M04	2210013M04	hypothetical Peptidyl-tRNA hydrolase containing protein
Human	chr9:127557899-127573433:+	AK057342	AK057342	Homo sapiens cDNA FLJ32780 fis, clone TESTI2002105, weakly similar to MICRONUCLEAR LINKER HISTONE POLYPROTEIN (MIC LH) [CONTAINS: LINKER HISTONE PROTEINS ALPHA, BETA, DELTA AND GAMMA].
Human	chr9:127573357-127577182:-	AK075520	BC062340	Homo sapiens torsin family 2, member A, mRNA (cDNA clone IMAGE:6372013), partial cds.
Mouse	chr2:32717017-32735624:-	1700129A21	1700129A21	Hypothetical tetratricopeptide repeat homolog [Mus musculus]
Mouse	chr2:32717225-32722241:+	BC003466	BC003466	Mus musculus, clone IMAGE:2649388, mRNA.
Human	chr9:128164341-128203303:+	AF151850	BC065003	Homo sapiens solute carrier family 27 (fatty acid transporter), member 4, mRNA (cDNA clone MGC:75102 IMAGE:6023438), complete cds.
Human	chr9:128150933-128164586:-	CR622527	CR622527	full-length cDNA clone CS0DK002YL18 of HeLa cells Cot 25-normalized of Homo sapiens (human).
Mouse	chr2:29747486-29757957:+	B020008B06	B020008B06	weakly similar to Ubiquinone biosynthesis protein COQ4 homolog (Coenzyme Q biosynthesis protein 4 homolog) (CGI-92) [Homo sapiens]
Mouse	chr2:29721246-29748524:-	G430055L02	E030027A11	similar to UNKNOWN (CDNA FLJ11094 FIS, CLONE PLACE1005373, WEAKLY SIMILAR TO TRNA PSEUDOURIDINE SYNTHASE B) (EC 4.2.1.70) (HYPOTHETICAL 36.7 KDA PROTEIN) [Homo sapiens]
Human	chr9:128297015-128343097:+	BC010629	AY366499	Homo sapiens outer dense fiber of sperm tails 2 (ODF2) mRNA, complete cds; alternatively spliced.
Human	chr9:128310790-128314845:-	AW303426	-	-
Mouse	chr2:29849225-29891768:+	AF000968	G430030G24	outer dense fiber of sperm tails 2
Mouse	chr2:29860472-29863486:-	4931422C16	4931422C16	outer dense fiber of sperm tails 2
Human	chr9:128544337-128562753:+	BC041581	BC041581	Homo sapiens, Similar to protein kinase PKNbeta, clone IMAGE:4647727, mRNA.
Human	chr9:128562715-128565975:-	BM844241	-	-
Mouse	chr2:30037706-30051044:+	D330006L06	F630107C11	protein kinase N3
Mouse	chr2:30050966-30053670:-	1190004A01	E330003I02	hypothetical DHHC-type Zn-finger containing protein
Human	chr9:128660302-128664516:+	CR603254	BC016351	Homo sapiens, clone MGC:24455 IMAGE:4080883, mRNA, complete cds.
Human	chr9:128661484-128671654:-	AL110193	AL110193	Homo sapiens mRNA; cDNA DKFZp566D143 (from clone DKFZp566D143); partial cds.
Mouse	chr2:30131516-30134092:+	AB012108	G630073A15	endonuclease G
Mouse	chr2:30133470-30138482:-	B130017L23	9930028P05	similar to HYPOTHETICAL 43.8 KDA PROTEIN (FRAGMENT) [Homo sapiens]
Human	chr9:131299014-131405138:+	AB011087	BC002872	Homo sapiens KIAA0515, mRNA (cDNA clone MGC:10526 IMAGE:3944379), complete cds.
Human	chr9:131402420-131405132:-	BC058910	BC058910	Homo sapiens cDNA clone IMAGE:4478733, partial cds.
Mouse	chr2:32111103-32194563:+	M5C1106J21	M5C1106J21	hypothetical BAT2, N-terminal/Glutamine-rich region profile/Serine-rich region profile/HPr serine phosphorylation site containing protein
Mouse	chr2:32192805-32196405:-	4921524C02	4921524C02	unclassifiable
Human	chr9:132574976-132599896:+	BC060821	BC060821	Homo sapiens general transcription factor IIIC, polypeptide 4, 90kDa, mRNA (cDNA clone IMAGE:4825930), partial cds.
Human	chr9:132497938-132575342:-	AK027484	AF427339	Homo sapiens DEAD/DEXH helicase DDX31 mRNA, complete cds.
Mouse	chr2:28782319-28800700:-	G830034H19	BC061476	Mus musculus cDNA clone MGC:69954 IMAGE:30290478, complete cds. CDS=67..876
Mouse	chr2:28800397-28865592:+	5830444G11	G730028J02	DEAD/H (Asp-Glu-Ala-Asp/His) box polypeptide 31
Human	chr9:133252975-133257780:+	BC014411	Z35094	H.sapiens mRNA for SURF-2.
Human	chr9:133247412-133253106:-	AI479876	BC071658	Homo sapiens surfeit 1, mRNA (cDNA clone MGC:87849 IMAGE:5539506), complete cds.
Mouse	chr2:26876389-26882293:+	G830006I21	F830203J05	Surfeit locus protein 2 (Surf-2)
Mouse	chr2:26873400-26876798:-	BC004755	BC004755	Mus musculus surfeit gene 1, mRNA (cDNA clone MGC:6703 IMAGE:3584373), complete cds. CDS=200..1120
Human	chr9:134759570-134962634:+	M76729	M76729	Human pro-alpha-1 (V) collagen mRNA, complete cds.
Human	chr9:134937206-134990409:-	BC058547	BC058547	Homo sapiens cDNA clone IMAGE:5468304, partial cds.
Mouse	chr2:27846075-27999536:+	G370005J07	AB009993	Mus musculus mRNA for collagen a1(V), complete cds. CDS=132..5648
Mouse	chr2:27983883-27990459:-	F930048M12	F930048M12	hypothetical protein
Human	chr9:136234471-136236386:+	AA286944	-	-
Human	chr9:136232372-136236654:-	BC021231	BC021231	Homo sapiens hypothetical gene supported by AK023162, mRNA (cDNA clone IMAGE:4109561), partial cds.
Mouse	chr2:26098568-26099565:+	1810012K08	1810012K08	unclassifiable
Mouse	chr2:26096833-26100543:-	F830016C21	C330006A16	unclassifiable

Human	chr9:136961614-137011477:+	AB075864	BC032104	Homo sapiens, clone IMAGE:4650210, mRNA.
Human	chr9:136970923-136979137:-	AK055547	AK055547	Homo sapiens cDNA FLJ30985 fis, clone HHDPC2000462.
Mouse	chr2:25568656-25569378:-	4930402J23	-	-
Mouse	chr2:25567885-25591783:+	AW226668	A230086C11	hypothetical protein
Human	chr9:137411502-137414067:+	AK026594	AK026594	Homo sapiens cDNA: FLJ22941 fis, clone KAT08078, highly similar to HSBT278 Homo sapiens mRNA for beta tubulin.
Human	chr9:137413873-137418059:-	AK097419	AK097419	Homo sapiens cDNA FLJ40100 fis, clone TESTI2004675.
Mouse	chr2:25182178-25184724:-	BC022919	I920030A10	Tubulin beta-2 chain homolog [Homo sapiens]
Mouse	chr2:25178767-25182303:+	BC061039	BC061039	Mus musculus 4931415M17 mRNA, mRNA (cDNA clone MGC:74147 IMAGE:6704633), complete cds. CDS=60..1019
Human	chr10:170405-290577:+	CR619559	BC034784	Homo sapiens, Similar to adenovirus 5 E1A binding protein, clone MGC:34429 IMAGE:5171494, mRNA, complete cds.
Human	chr10:289685-290565:-	BU902898	-	-
Mouse	chr13:9566501-9646802:-	I530012C13	5730564N10	ADENOVIRUS 5 E1A-BINDING PROTEIN (BS69 PROTEIN) homolog [Homo sapiens]
Mouse	chr13:9565898-9571209:+	BU936549	-	-
Human	chr10:1084980-1200612:+	BC018044	AB023199	Homo sapiens mRNA for KIAA0982 protein, complete cds.
Human	chr10:1075848-1092650:-	BC057827	BC057827	Homo sapiens isopentenyl-diphosphate delta isomerase, mRNA (cDNA clone MGC:71798 IMAGE:30337530), complete cds.
Mouse	chr13:8657345-8726288:-	9630045H05	I730034E13	WD repeat domain 37
Mouse	chr13:8724720-8726484:+	4933417A01	4933417A01	unclassifiable
Human	chr10:3099687-3169907:+	CR621705	BC029138	Homo sapiens, phosphofructokinase, platelet, clone MGC:35337 IMAGE:5180268, mRNA, complete cds.
Human	chr10:3165147-3205012:-	BX649017	BX649017	Homo sapiens mRNA; cDNA DKFZp686C03109 (from clone DKFZp686C03109).
Mouse	chr13:6371426-6458395:-	B930046O19	C630002O05	phosphofructokinase, platelet
Mouse	chr13:6339647-6371830:+	2310012C15	I730091N12	pitrilysin metalloprotease 1
Human	chr10:5766831-5845941:+	AK128051	AB095927	Homo sapiens mRNA for KIAA2006 protein.
Human	chr10:5845415-5924101:-	CR592483	Y13286	Homo sapiens mRNA for GDP dissociation inhibitor beta.
Mouse	chr13:3318432-3373410:-	BC045617	F830015I20	hypothetical protein
Mouse	chr13:3290361-3320268:+	B230030K06	C530041M08	guanosine diphosphate (GDP) dissociation inhibitor 3
Human	chr10:8135614-8157266:+	BC006793	BC006793	Homo sapiens, GATA-binding protein 3, clone MGC:5199 IMAGE:2985843, mRNA, complete cds.
Human	chr10:8136452-8138947:-	CD722170	-	-
Mouse	chr2:9808179-9841139:-	F930010J11	F930010J11	GATA binding protein 3
Mouse	chr2:9828361-9830108:+	A530052G17	-	-
Human	chr10:11693309-11694231:+	BC037281	BC037281	Homo sapiens, clone MGC:33041 IMAGE:4838780, mRNA, complete cds.
Human	chr10:11542515-11693787:-	BC010351	D13644	Homo sapiens mRNA for KIAA0019 protein, complete cds.
Mouse	chr2:6270106-6270923:-	1700014B07	1700014B07	hypothetical protein
Mouse	chr2:6270510-6394233:+	F630119J10	F630119J10	Similar to related to the N terminus of tre homolog [Mus musculus]
Human	chr10:12150940-12251966:+	BC026179	AB046850	Homo sapiens mRNA for KIAA1630 protein, partial cds.
Human	chr10:12246842-12278149:-	AF218818	AF218818	Homo sapiens nudix hydrolase NUDT5 mRNA, complete cds.
Mouse	chr2:5818820-5843275:-	2610019K07	5730536E22	SEC61, alpha subunit 2 (S. cerevisiae)
Mouse	chr2:5792862-5818895:+	2600001M11	E430007J15	nudix (nucleoside diphosphate linked moiety X)-type motif 5
Human	chr10:12277967-12332607:+	D14878	D14878	Human mRNA for protein D123, complete cds.
Human	chr10:12246842-12278149:-	BC000025	AF218818	Homo sapiens nudix hydrolase NUDT5 mRNA, complete cds.
Mouse	chr2:5742137-5793007:-	AA914699	G431001I09	D123 homolog [Rattus norvegicus]
Mouse	chr2:5792862-5818895:+	E430007J15	E430007J15	nudix (nucleoside diphosphate linked moiety X)-type motif 5
Human	chr10:13668933-13737935:+	BC002572	BC002572	Homo sapiens PRP18 pre-mRNA processing factor 18 homolog (yeast), mRNA (cDNA clone IMAGE:3160552), partial cds.
Human	chr10:13725712-14544149:-	AB037715	AB037715	Homo sapiens mRNA for KIAA1294 protein, partial cds.
Mouse	chr2:4561778-4599978:-	M5H1101O08	BC028306	Mus musculus cDNA clone IMAGE:3496857, containing frame-shift errors.
Mouse	chr2:3965560-4561885:+	CB337708	BC058672	Mus musculus RIKEN cDNA 2700017I06 gene, mRNA (cDNA clone MGC:76363 IMAGE:6830456), complete cds. CDS=240..3335
Human	chr10:13718036-13757996:+	W03645	-	-
Human	chr10:13725712-14544149:-	AB037715	AB037715	Homo sapiens mRNA for KIAA1294 protein, partial cds.
Mouse	chr2:4533867-4535130:-	1110023J12	1110023J12	unclassifiable
Mouse	chr2:3965560-4561885:+	G370139E21	BC058672	Mus musculus RIKEN cDNA 2700017I06 gene, mRNA (cDNA clone MGC:76363 IMAGE:6830456), complete cds. CDS=240..3335
Human	chr10:16518969-16596093:+	BX648556	BX648556	Homo sapiens mRNA; cDNA DKFZp779I2035 (from clone DKFZp779I2035).
Human	chr10:16593843-16602702:-	AK094686	AK094686	Homo sapiens cDNA FLJ37367 fis, clone BRAMY2024489.
Mouse	chr2:12875146-12954559:+	0610042L23	0610042L23	phosphotriesterase related
Mouse	chr2:12952886-12962868:-	1110065A22	AB044560	Mus musculus mRNA for Gliacolin, complete cds. CDS=901..1668

Human	chr10:21843946-21847212:+	AK055656	AK055656	Homo sapiens cDNA FLJ31094 fis, clone IMR321000165.
Human	chr10:21842410-21854617:-	AK131456	AK131456	Homo sapiens cDNA FLJ16611 fis, clone TESTI4011829.
Mouse	chr2:18103768-18104684:+	BE948681	-	-
Mouse	chr2:18096207-18104788:-	BC050860	BC050860	Mus musculus cDNA clone MGC:59463 IMAGE:6331376, complete cds. CDS=120..2588
Human	chr10:21843946-21847212:+	AK055656	AK055656	Homo sapiens cDNA FLJ31094 fis, clone IMR321000165.
Human	chr10:21842410-21854617:-	AK131456	AK131456	Homo sapiens cDNA FLJ16611 fis, clone TESTI4011829.
Mouse	chr2:18101287-18268147:+	CN538007	F630019N14	myeloid/lymphoid or mixed lineage-leukemia translocation to 10 homolog (Drosophila)
Mouse	chr2:18096207-18104788:-	BC050860	BC050860	Mus musculus cDNA clone MGC:59463 IMAGE:6331376, complete cds. CDS=120..2588
Human	chr10:60606353-60677533:+	AL834339	AL834339	Homo sapiens mRNA; cDNA DKFZp761M0113 (from clone DKFZp761M0113).
Human	chr10:60675895-60792594:-	BC036453	BC036453	Homo sapiens, Similar to RIKEN cDNA 1200015N20 gene, clone MGC:33233 IMAGE:5270033, mRNA, complete cds.
Mouse	chr10:70331297-70429576:-	4921513D22	D630013N20	unclassifiable
Mouse	chr10:70214273-70332544:+	9330137H23	A530085M12	RIKEN cDNA 1200015N20 gene
Human	chr10:69760937-69772959:+	AX810704	AX810704	Sequence 28 from Patent EP1333092.
Human	chr10:69770870-69837212:-	AF461266	AF461266	Homo sapiens RUFY2 (RUFY2) mRNA, complete cds.
Mouse	chr10:62780406-62789631:-	7530418N21	BC049652	Mus musculus, clone IMAGE:6741456, mRNA.
Mouse	chr10:62745994-62784484:+	B230311F01	B230311F01	differentially expressed in normal and neoplastic cells
Human	chr10:74597920-74671945:+	AK025189	BC015394	Homo sapiens, clone MGC:21703 IMAGE:4431919, mRNA, complete cds.
Human	chr10:74664307-74665796:-	AF143875	AF143875	Homo sapiens clone IMAGE:113308 mRNA sequence.
Mouse	chr14:16633458-16670118:+	BC059221	AK129252	Mus musculus mRNA for mKIAA0974 protein. CDS=214..1323
Mouse	chr14:16663443-16664135:-	AI508498	-	-
Human	chr10:74925288-74935320:+	BU150385	BC080555	Homo sapiens cDNA clone IMAGE:6261730.
Human	chr10:74927302-75005475:-	BC026037	AK127081	Homo sapiens cDNA FLJ45138 fis, clone BRAWH3039258.
Mouse	chr14:16831410-16855983:+	1810062O18	1810062O18	hypothetical protein
Mouse	chr14:16834208-16926359:-	AW911953	C030002J06	hypothetical Ubiquitin carboxyl-terminal hydrolase family 2 containing protein
Human	chr10:75174137-75201939:+	AK098512	BC018928	Homo sapiens SEC24 related gene family, member C (S. cerevisiae), transcript variant 2, mRNA (cDNA clone MGC:14092 IMAGE:4123623), complete cds.
Human	chr10:75199028-75202751:-	AK126671	AK126671	Homo sapiens cDNA FLJ44715 fis, clone BRACE3021430.
Mouse	chr14:16959604-16980148:+	3021402E24	BC040370	Mus musculus hypothetical protein LOC218811, mRNA (cDNA clone MGC:25454 IMAGE:4239204), complete cds. CDS=75..3365
Mouse	chr14:16972530-16988324:-	6230400D17	6230400D17	hypothetical protein
Human	chr10:80498798-80746282:+	BC007288	BC007288	Homo sapiens, Similar to osa, clone IMAGE:3347864, mRNA.
Human	chr10:80373089-80498823:-	AW449810	AK098249	Homo sapiens cDNA FLJ40930 fis, clone UTERU2006899.
Mouse	chr14:21754654-21964265:+	BC058646	BC058646	Mus musculus cDNA clone MGC:76345 IMAGE:6405777, complete cds. CDS=525..3725
Mouse	chr14:21549961-21756873:-	C130026E14	D930049A15	unclassifiable
Human	chr10:90332673-90333598:+	BI838705	-	-
Human	chr10:90023601-90333069:-	AK002080	AK002080	Homo sapiens cDNA FLJ11218 fis, clone PLACE1008095.
Mouse	chr19:32704512-32705530:+	2810449D17	2810449D17	unclassifiable
Mouse	chr19:32450341-32705062:-	F630047D06	F630047D06	hypothetical protein
Human	chr10:91393825-91400405:+	BX091541	-	-
Human	chr10:91332721-91395309:-	AY027661	AY027661	Homo sapiens pantothenate kinase 1 alpha (PANK1) mRNA, complete cds, alternatively spliced.
Mouse	chr19:34099800-34103565:+	4632412I06	4632412I06	hypothetical protein
Mouse	chr19:34030071-34101060:-	AF347700	AF347700	Mus musculus pantothenate kinase 1 alpha (Pank1) mRNA, complete cds. CDS=378..2024
Human	chr10:100196109-100203552:+	BX103104	-	-
Human	chr10:100127208-100196699:-	BG719278	BC006131	Homo sapiens, clone MGC:13047 IMAGE:3626506, mRNA, complete cds.
Mouse	chr19:42115690-42118232:+	C230096I11	C230096I11	unclassifiable
Mouse	chr19:42091763-42116636:-	F630115D10	F630115D10	Hermansky-Pudlak syndrome 1 homolog (human)
Human	chr10:101409239-101505934:+	BC028948	AK055540	Homo sapiens cDNA FLJ30978 fis, clone HHDPC2000102, highly similar to Homo sapiens lysosomal apyrase-like protein 1 (LALP1) mRNA.
Human	chr10:101444040-101482632:-	BX537557	BX537557	Homo sapiens mRNA; cDNA DKFZp686C1869 (from clone DKFZp686C1869).
Mouse	chr19:43026330-43071874:+	9230108M13	M5C1032P19	ectonucleoside triphosphate diphosphohydrolase
Mouse	chr19:43069910-43089658:-	D130084L13	D130084L13	SIMILAR TO COX15 HOMOLOG, CYTOCHROME C OXIDASE ASSEMBLY PROTEIN (YEAST) (FRAGMENT) homolog [Mus musculus]
Human	chr10:101409239-101505934:+	AL713774	AK055540	Homo sapiens cDNA FLJ30978 fis, clone HHDPC2000102, highly similar to Homo sapiens lysosomal apyrase-like protein 1 (LALP1) mRNA.
Human	chr10:101459623-101460600:-	BM741633	-	-
Mouse	chr19:43026330-43071874:+	9230108M13	M5C1032P19	ectonucleoside triphosphate diphosphohydrolase
Mouse	chr19:43069910-43089658:-	D130084L13	D130084L13	SIMILAR TO COX15 HOMOLOG, CYTOCHROME C OXIDASE ASSEMBLY PROTEIN (YEAST) (FRAGMENT) homolog [Mus musculus]

Human	chr10:102746850-102757583:+	AL834338	BC006212	Homo sapiens KIAA1813 protein, mRNA (cDNA clone MGC:2586 IMAGE:3161855), complete cds.
Human	chr10:102757426-102780880:-	AK024422	AK024422	Homo sapiens mRNA for FLJ00011 protein, partial cds.
Mouse	chr19:44351127-44363766:+	G430046J22	G430046J22	leucine zipper, putative tumor suppressor 2
Mouse	chr19:44363557-44383265:-	9130207N01	9130207N01	weakly similar to FLJ00011 PROTEIN (FRAGMENT) [Homo sapiens]
Human	chr10:105026892-105040098:+	BC006359	BC006359	Homo sapiens internexin neuronal intermediate filament protein, alpha, mRNA (cDNA clone MGC:12702 IMAGE:4125949), complete cds.
Human	chr10:104948480-105027357:-	BI668548	BC040734	Homo sapiens, clone IMAGE:5198998, mRNA.
Mouse	chr19:46359235-46369877:+	1422000G19	-	-
Mouse	chr19:46228719-46359739:-	9330019J22	9330019J22	hypothetical protein
Human	chr10:105243632-105342299:+	U87864	BC026336	Homo sapiens, neuralized-like (Drosophila), clone MGC:26482 IMAGE:4812302, mRNA, complete cds.
Human	chr10:105333953-105334736:-	CA312912	-	-
Mouse	chr19:46523370-46603987:+	6820434L06	BC058386	Mus musculus neuralized homolog (Drosophila), mRNA (cDNA clone MGC:66919 IMAGE:6831952), complete cds. CDS=473..2197
Mouse	chr19:46596793-46598882:-	A930014J05	A930014J05	unclassifiable
Human	chr10:105344876-105346192:+	BU071214	-	-
Human	chr10:105343773-105605241:-	AK123570	AK056469	Homo sapiens cDNA FLJ31907 fis, clone NT2RP7004396, highly similar to Mus musculus mRNA for Fish protein.
Mouse	chr19:46606135-46609608:+	C230050L11	C230050L11	hypothetical protein
Mouse	chr19:46604724-46808973:-	A530092M21	AJ007012	Mus musculus mRNA for Fish protein. CDS=277..3651
Human	chr10:105348326-105348825:+	BE503994	-	-
Human	chr10:105343773-105605241:-	BX647371	AK056469	Homo sapiens cDNA FLJ31907 fis, clone NT2RP7004396, highly similar to Mus musculus mRNA for Fish protein.
Mouse	chr19:46606135-46609608:+	C230050L11	C230050L11	hypothetical protein
Mouse	chr19:46604724-46808973:-	A530092M21	AJ007012	Mus musculus mRNA for Fish protein. CDS=277..3651
Human	chr10:112621555-112649754:+	AL049932	BC031049	Homo sapiens, programmed cell death 4 (neoplastic transformation inhibitor), clone MGC:33047 IMAGE:5277621, mRNA, complete cds.
Human	chr10:112648476-112669023:-	BC073157	BC073157	Homo sapiens hypothetical protein LOC92482, mRNA (cDNA clone IMAGE:6063114), partial cds.
Mouse	chr19:53262704-53300333:+	F830225F13	6030445M03	programmed cell death 4
Mouse	chr19:53298646-53315100:-	A230104C04	2310065J03	weakly similar to somatotropin intron-related protein RDE.25
Human	chr10:112826776-112830655:+	AF284095	AF284095	Homo sapiens alpha-2A adrenergic receptor mRNA, complete cds.
Human	chr10:112827351-112828959:-	CK823481	-	-
Mouse	chr19:53415655-53419456:+	C630032H01	C630032H01	adrenergic receptor, alpha 2a
Mouse	chr19:53414624-53417357:-	B230352O11	B230352O11	unclassifiable
Human	chr10:115302727-115339356:+	D49742	D49742	Human mRNA for HGF activator like protein, complete cds.
Human	chr10:115338573-115413874:-	BX641052	BX640730	Homo sapiens mRNA; cDNA DKFZp451P181 (from clone DKFZp451P181); complete cds.
Mouse	chr19:55670918-55705852:+	4432414L05	4432414L05	hyaluronic acid binding protein 2
Mouse	chr19:55705073-55774654:-	U76618	U76618	Mus musculus N-RAP mRNA, complete cds. CDS=1523..5050
Human	chr10:115923853-115924354:+	AI143965	-	-
Human	chr10:115870611-115923974:-	BC030557	BX537964	Homo sapiens mRNA; cDNA DKFZp686K1351 (from clone DKFZp686K1351); complete cds.
Mouse	chr19:56210320-56210897:+	1700122L01	1700122L01	hypothetical protein
Mouse	chr19:56175634-56210548:-	E230029C14	AF349751	Mus musculus oocyte-testis gene 1 (Otg1) mRNA, complete cds. CDS=1..2754
Human	chr10:118599013-118661287:+	CN358600	BX647301	Homo sapiens mRNA; cDNA DKFZp781N1041 (from clone DKFZp781N1041).
Human	chr10:118420693-118599706:-	BC041412	BC041412	Homo sapiens heat shock 70kDa protein 12A, mRNA (cDNA clone IMAGE:5285193), partial cds.
Mouse	chr19:58341980-58369998:+	6430537H07	6430537H07	hypothetical Enolase containing protein
Mouse	chr19:58194325-58342231:-	4930408G03	D130065H16	heat shock 70kDa protein 12A
Human	chr10:118990594-119028994:+	BC030593	BC030593	Homo sapiens, clone IMAGE:4828946, mRNA.
Human	chr10:119026775-119124968:-	AA055652	BC028375	Homo sapiens, similar to PDZ domain proteins, clone MGC:27107 IMAGE:4837939, mRNA, complete cds.
Mouse	chr19:58659457-58695965:+	9530080J18	9530080J18	reserpine-sensitive vesicular monoamine transporter homolog [Rattus norvegicus]
Mouse	chr19:58691810-58744404:-	E430018M18	E430018M18	unclassifiable
Human	chr10:119291946-119299049:+	AF301598	AF301598	Homo sapiens empty spiracles-like protein (EMX2) mRNA, complete cds.
Human	chr10:119222714-119294569:-	AY117413	AY117034	Homo sapiens empty spiracles homolog 2 (EMX2) antisense variant_2 mRNA, complete sequence.
Mouse	chr19:58856951-58863937:+	AY117415	AY117415	Mus musculus empty spiracles-like protein 2 mRNA, complete cds. CDS=845..1606
Mouse	chr19:58823685-58857214:-	AY117414	AY117414	Mus musculus EMX2OS mRNA, complete sequence.
Human	chr10:124703887-124747023:+	CA847782	AK094271	Homo sapiens cDNA FLJ36952 fis, clone BRACE2005773.
Human	chr10:124740312-124774200:-	CR749800	AK122899	Homo sapiens cDNA FLJ16565 fis, clone SYNOV4007711, highly similar to Homo sapiens zinc finger protein, subfamily 1A, 5 (Pegasus) (PEGASUS).
Mouse	chr7:118942650-118964764:+	4632417G23	5730458D16	hypothetical P-loop containing nucleotide triphosphate hydrolases structure containing protein
Mouse	chr7:118957503-118982656:-	BC048183	BC048183	Mus musculus RIKEN cDNA 2610034F18 gene, mRNA (cDNA clone MGC:61239 IMAGE:6826723), complete cds. CDS=143..1402

Human	chr10:127502083-127532254:+	AK055691	AY064248	Homo sapiens BRCA2 and Cip1/p21 interacting protein splice variant alpha (BCCIP) mRNA, complete cds.
Human	chr10:127514891-127575023:-	AL162051	BC068471	Homo sapiens DEAH (Asp-Glu-Ala-His) box polypeptide 32, mRNA (cDNA clone MGC:87093 IMAGE:30343301), complete cds.
Mouse	chr7:121334063-121345877:+	2410046F21	1110013J05	similar to TOK-1BETA [Homo sapiens]
Mouse	chr7:121345665-121407511:-	I920073F14	4732469F02	HELICASE DDX32
Human	chr10:134201294-134446974:+	BC062300	Z31695	H.sapiens mRNA for 43 kDa inositol polyphosphate 5-phosphatase.
Human	chr10:134445566-134448187:-	BM471872	-	-
Mouse	chr7:127039280-127230046:+	BC056341	BC056341	Mus musculus hypothetical protein LOC212111, mRNA (cDNA clone MGC:73425 IMAGE:5703345), complete cds. CDS=282..1520
Mouse	chr7:127229770-127233191:-	2010010A07	2010010A07	NK6 transcription factor related, locus 2 (Drosophila)
Human	chr10:135011274-135015547:+	CR620153	CR620153	full-length cDNA clone CS0DD009YJ05 of Neuroblastoma Cot 50-normalized of Homo sapiens (human).
Human	chr10:134982016-135014722:-	AK092113	AK092113	Homo sapiens cDNA FLJ34794 fis, clone NT2NE2005676, highly similar to Homo sapiens spindle pole body protein spc97 homolog GCP2 mRNA.
Mouse	chr7:127687293-127691507:+	2410004P19	2410004P19	hypothetical protein
Mouse	chr7:127648680-127687576:-	F930035K11	8030448J16	GAMMA-TUBULIN COMPLEX PROTEIN 2 homolog [Homo sapiens]
Human	chr11:526641-544916:+	BC035936	AK024495	Homo sapiens mRNA for FLJ00101 protein, partial cds.
Human	chr11:544855-550779:-	BC037936	BC039077	Homo sapiens, clone MGC:35138 IMAGE:5168905, mRNA, complete cds.
Mouse	chr7:128884997-128901063:+	BC027807	5730427C23	hypothetical Leucine-rich repeat containing protein
Mouse	chr7:128900994-128902005:-	1600016N20	1600016N20	hypothetical protein
Human	chr11:685189-694129:+	BC038383	BC008671	Homo sapiens, Similar to RIKEN cDNA 5530601I19 gene, clone MGC:9743 IMAGE:3854028, mRNA, complete cds.
Human	chr11:634225-696711:-	AF049460	AF049460	Homo sapiens nuclear DEAF-1 related transcriptional regulator protein 8 mRNA, complete cds.
Mouse	chr7:129018528-129028118:+	6330577M18	6330577M18	hypothetical protein
Mouse	chr7:128987938-129029745:-	G430140O12	BC046399	Mus musculus deformed epidermal autoregulatory factor 1 (Drosophila), mRNA (cDNA clone MGC:51639 IMAGE:5063995), complete cds. CDS=316..1668
Human	chr11:2118307-2126472:+	CR602283	AB030733	Homo sapiens PEG8/IGF2AS mRNA, imprinting gene, complete cds.
Human	chr11:2106918-2139025:-	M29645	M29645	Human insulin-like growth factor II mRNA, complete cds.
Mouse	chr7:130364465-130375128:+	AB030734	AB030734	Mus musculus Peg8/Igf2as mRNA, imprinting gene.
Mouse	chr7:130355538-130371588:-	CN527329	BC053489	Mus musculus insulin-like growth factor 2, mRNA (cDNA clone MGC:60598 IMAGE:30013295), complete cds. CDS=87..629
Human	chr11:2353979-2375224:+	CR625241	M33680	Human 26-kDa cell surface protein TAPA-1 mRNA, complete cds.
Human	chr11:2306556-2355802:-	BC019904	BC019904	Homo sapiens, clone IMAGE:4940779, mRNA.
Mouse	chr7:130757511-130776072:+	G930050D20	F630118D04	CD 81 antigen
Mouse	chr7:130726556-130758449:-	9430050L03	C630032D20	unclassifiable
Human	chr11:2378294-2381684:+	BC050616	BC050616	Homo sapiens tumor suppressing subtransferable candidate 4, mRNA (cDNA clone MGC:60084 IMAGE:5744058), complete cds.
Human	chr11:2375947-2378416:-	AK095568	AK095568	Homo sapiens cDNA FLJ38249 fis, clone FCBBF2007633.
Mouse	chr7:130757511-130776072:+	AB041597	F630118D04	CD 81 antigen
Mouse	chr7:130773925-130799414:-	CF727047	AF228681	Mus musculus MTR1 (Mtr1) mRNA, complete cds. CDS=88..3564
Human	chr11:3643811-3645373:+	BC033833	BC033833	Homo sapiens, clone IMAGE:4154817, mRNA.
Human	chr11:3643393-3649190:-	AF199235	AF199235	Homo sapiens nicotinic acetylcholine receptor subunit alpha 10 mRNA, complete cds.
Mouse	chr7:89524533-89536723:-	2310050E11	2310050E11	ADP-ribosyltransferase 1
Mouse	chr7:89521638-89527200:+	7630402G21	7630402G21	NEURONAL NICOTINIC ACETYLCHOLINE RECEPTOR SUBUNIT homolog [Rattus norvegicus]
Human	chr11:6506530-6571562:+	AK074178	BX647806	Homo sapiens mRNA; cDNA DKFZp686J0796 (from clone DKFZp686J0796).
Human	chr11:6492639-6582226:-	AI942468	AB007869	Homo sapiens KIAA0409 mRNA, partial cds.
Mouse	chr7:93221878-93223505:+	A330093I21	-	-
Mouse	chr7:93221857-93266082:-	F630215D14	F630021J24	similar to Hypothetical protein KIAA0409 (Cerebral protein-1) (Fragment) [Homo sapiens]
Human	chr11:6581537-6588681:+	CR625625	CR625625	full-length cDNA clone CS0DG004YO19 of B cells (Ramos cell line) of Homo sapiens (human).
Human	chr11:6492639-6582226:-	BI762183	AB007869	Homo sapiens KIAA0409 mRNA, partial cds.
Mouse	chr7:93265287-93271622:+	2610044H13	2610044H13	integrin linked kinase
Mouse	chr7:93221857-93266082:-	F630021J24	F630021J24	similar to Hypothetical protein KIAA0409 (Cerebral protein-1) (Fragment) [Homo sapiens]
Human	chr11:6581537-6588681:+	BC001554	CR625625	full-length cDNA clone CS0DG004YO19 of B cells (Ramos cell line) of Homo sapiens (human).
Human	chr11:6582578-6590476:-	AL833496	AL833496	Homo sapiens mRNA; cDNA DKFZp686M1629 (from clone DKFZp686M1629).
Mouse	chr7:93265287-93271622:+	U94479	2610044H13	integrin linked kinase
Mouse	chr7:93268090-93273060:-	B930022J16	B930022J16	TAF10 RNA polymerase II, TATA box binding protein (TBP)-associated factor, 30 kDa
Human	chr11:7997389-8117922:+	U54644	U82467	Human tub homolog (TUB) mRNA, complete cds.
Human	chr11:8071088-8147163:-	AK128611	AL832601	Homo sapiens mRNA; cDNA DKFZp451C1317 (from clone DKFZp451C1317).
Mouse	chr7:96219373-96303888:+	M5C1110B01	A930010O03	tubby candidate gene
Mouse	chr7:96303695-96352759:-	C230070A05	6330519K19	RIC3 homolog [Homo sapiens]

Human	chr11:8942644-8955650:+	BX365986	-	-
Human	chr11:8925324-8943073:-	CD105088	BC040124	Homo sapiens, Similar to chromosome 11 open reading frame 15, clone MGC:48749 IMAGE:6053804, mRNA, complete cds.
Mouse	chr7:97022572-97023457:+	4930431P19	4930431P19	hypothetical protein
Mouse	chr7:97006194-97023235:-	C630007E02	C630007E02	C11ORF15 PROTEIN homolog [Homo sapiens]
Human	chr11:16716524-16736477:+	BP226882	BC007103	Homo sapiens, small acidic protein, clone MGC:14746 IMAGE:4281282, mRNA, complete cds.
Human	chr11:15949555-16716766:-	AK097455	AF309034	Homo sapiens SOX6 mRNA, complete cds.
Mouse	chr7:103318398-103387860:+	BU516735	1110004F10	small acidic protein
Mouse	chr7:103344594-103375792:-	BC048672	BC048672	Mus musculus, clone IMAGE:6774078, mRNA.
Human	chr11:22603332-22791123:+	BC005077	BC013326	Homo sapiens, Similar to growth arrest-specific 2, clone IMAGE:4291917, mRNA.
Human	chr11:22600532-22603963:-	BC063038	AK023153	Homo sapiens cDNA FLJ13091 fis, clone NT2RP3002146.
Mouse	chr7:39318885-39452074:+	4631403A13	4631403A13	growth arrest specific 2
Mouse	chr7:39317640-39319330:-	6820427C17	B020005G17	unclassifiable
Human	chr11:26309547-26641412:+	AJ300461	AJ300461	Homo sapiens mRNA for C11ORF25 gene.
Human	chr11:26537154-26550399:-	AK128337	AK128337	Homo sapiens cDNA FLJ46479 fis, clone THYMU3025642.
Mouse	chr2:110574227-110841895:-	B230324K02	B230324K02	HYPOTHETICAL 114.7 KDA PROTEIN homolog [Homo sapiens]
Mouse	chr2:110613017-110631062:+	D730002G06	D730002G06	weakly similar to MUC15 protein precursor [Homo sapiens]
Human	chr11:31347953-31410958:+	AL050199	BC044931	Homo sapiens hypothetical protein LOC120526, mRNA (cDNA clone MGC:49947 IMAGE:6059077), complete cds.
Human	chr11:31409963-31487757:-	AK057788	AK057788	Homo sapiens cDNA FLJ25059 fis, clone CBL04610.
Mouse	chr2:105852484-105896281:-	6430539I15	1700030A21	hypothetical DnaJ N-terminal domain containing protein
Mouse	chr2:105797364-105859597:+	6430583O11	6430583O11	DJ1137O17.1 (SIMILAR TO PUTATIVE MITOCHONDRIAL INNER MEMBRANE PROTEASE SUBNUNIT 2) (FRAGMENT) homolog [Homo sapiens]
Human	chr11:32561892-32581591:+	CR608161	CR593695	full-length cDNA clone CS0DC003YN22 of Neuroblastoma Cot 25-normalized of Homo sapiens (human).
Human	chr11:32580368-32772779:-	AK128159	AK131470	Homo sapiens cDNA FLJ16638 fis, clone TESTI4025865.
Mouse	chr2:104891523-104909892:-	BC005598	I730035K05	dendritic cell protein GA17
Mouse	chr2:104779136-104913541:+	4931417A20	4931417A20	hypothetical protein
Human	chr11:33069324-33071230:+	AK027139	AK027139	Homo sapiens cDNA: FLJ23486 fis, clone LNG00379.
Human	chr11:33055310-33139619:-	BC010533	BC009792	Homo sapiens, clone MGC:13579 IMAGE:4274695, mRNA, complete cds.
Mouse	chr2:104551396-104557552:-	9330195I24	9330195I24	hypothetical protein
Mouse	chr2:104483247-104558929:+	BC003241	BC003241	Mus musculus cleavage stimulation factor, 3' pre-RNA, subunit 3, mRNA (cDNA clone MGC:6373 IMAGE:3498424), complete cds. CDS=154..2307
Human	chr11:34029752-34080734:+	BC001731	BC001731	Homo sapiens, membrane component, chromosome 11, surface marker 1, clone MGC:1378 IMAGE:3355481, mRNA, complete cds.
Human	chr11:34030649-34031328:-	AW169893	-	-
Mouse	chr2:103655953-103690655:-	G930025C23	B130003F06	GPI-anchored membrane protein 1
Mouse	chr2:103689281-103689848:+	M230012G03	-	-
Human	chr11:43920716-43922009:+	BC052560	BC052560	Homo sapiens cDNA clone IMAGE:6272440, partial cds.
Human	chr11:43921168-43925332:-	BC024745	AK093366	Homo sapiens cDNA FLJ36047 fis, clone TESTI2017951.
Mouse	chr2:93849798-93904791:-	E530011N06	BC018196	Mus musculus RIKEN cDNA 1810020C19 gene, mRNA (cDNA clone MGC:25757 IMAGE:3993214), complete cds. CDS=120..980
Mouse	chr2:93849798-93850179:+	W18035	-	-
Human	chr11:46905451-47142512:+	CK724853	BC001860	Homo sapiens hypothetical protein MGC4707, mRNA (cDNA clone MGC:4707 IMAGE:3534541), complete cds.
Human	chr11:47142424-47164586:-	AK098324	AK098324	Homo sapiens cDNA FLJ41005 fis, clone UTERU2017613, highly similar to Mus musculus zinc finger protein 289 (Zfp289) mRNA.
Mouse	chr2:91167300-91338773:-	BC032255	BC032255	Mus musculus RIKEN cDNA 1110051M20 gene, mRNA (cDNA clone MGC:40841 IMAGE:5368901), complete cds. CDS=49..1029
Mouse	chr2:91158867-91171430:+	2310032E02	BC005495	Mus musculus zinc finger protein 289, mRNA (cDNA clone MGC:7383 IMAGE:3487811), complete cds. CDS=67..1629
Human	chr11:47530485-47562693:+	AK127967	AK127967	Homo sapiens cDNA FLJ46081 fis, clone TESTI2005112, highly similar to NADH-ubiquinone oxidoreductase 30 kDa subunit, mitochondrial precursor (EC 1.6.5.3).
Human	chr11:47444064-47543648:-	BP358103	U63289	Human RNA-binding protein CUG-BP/hNab50 (NAB50) mRNA, complete cds.
Mouse	chr2:90802717-90812375:-	BI738603	I920013H23	hypothetical protein
Mouse	chr2:90811937-90814550:+	8430408E23	8430408E23	unclassifiable
Human	chr11:47530485-47562693:+	BC018974	AK127967	Homo sapiens cDNA FLJ46081 fis, clone TESTI2005112, highly similar to NADH-ubiquinone oxidoreductase 30 kDa subunit, mitochondrial precursor (EC 1.6.5.3).
Human	chr11:47550325-47557131:-	AK001749	AK001312	Homo sapiens cDNA FLJ10450 fis, clone NT2RP1000954, weakly similar to RING CANAL PROTEIN.
Mouse	chr2:90802717-90812375:-	I920013H23	I920013H23	hypothetical protein
Mouse	chr2:90798799-90805685:+	BC025103	BC025103	Mus musculus RIKEN cDNA 2510026C23 gene, mRNA (cDNA clone MGC:35684 IMAGE:4481431), complete cds. CDS=11..1615
Human	chr11:57039096-57040653:+	BX444320	-	-
Human	chr11:57008111-57039800:-	AB103033	AB103033	Homo sapiens lat3 mRNA for L-type amino acid transporter 3, complete cds.
Mouse	chr2:84732909-84734392:-	D930003E18	D930003E18	unclassifiable
Mouse	chr2:84733454-84757653:+	F430203O15	B630013A07	solute carrier family 43, member 1

Human	chr11:60366047-60375142:+	BC001378	AK025974	Homo sapiens cDNA: FLJ22321 fis, clone HRC05390.
Human	chr11:60374974-60380020:-	AF144308	AB008535	Homo sapiens mRNA for CRTH2, complete cds.
Mouse	chr19:10040588-10048389:-	9930013P05	9930013P05	BRAIN CDNA, CLONE MNCB-4327 (FRAGMENT) homolog [Mus musculus]
Mouse	chr19:10036267-10041618:+	AB109092	AB109092	Mus musculus crth2 mRNA for prostaglandin D2 receptor CRTH2, complete cds. CDS=254..1402
Human	chr11:60448290-60461207:+	AK023577	AK000546	Homo sapiens cDNA FLJ20539 fis, clone KAT11311.
Human	chr11:60461131-60476628:-	CR607140	AB020598	Homo sapiens mRNA for peptide transporter 3, complete cds.
Mouse	chr19:9956929-9981108:-	BC006896	C130073M18	UNKNOWN (PROTEIN FOR MGC:11927) homolog [Mus musculus]
Mouse	chr19:9938834-9959669:+	F830005G04	F830005G04	solute carrier family 15, member 3
Human	chr11:60857258-60877474:+	AK023915	CR593561	full-length cDNA clone CS0DL012YE24 of B cells (Ramos cell line) Cot 25-normalized of Homo sapiens (human).
Human	chr11:60823493-60857414:-	CD613382	AL831958	Homo sapiens mRNA; cDNA DKFZp451P0416 (from clone DKFZp451P0416).
Mouse	chr19:9686872-9704525:-	A530029H04	A530029H04	weakly similar to Dihydroxyacetone kinase [Thermoanaerobacter tengcongensis]
Mouse	chr19:9704192-9728909:+	CA328059	F830219B01	damage specific DNA binding protein 1
Human	chr11:60886049-60900918:+	CR596588	CR596588	full-length cDNA clone CS0DK011YD04 of HeLa cells Cot 25-normalized of Homo sapiens (human).
Human	chr11:60872798-60886338:-	CR609780	AK056751	Homo sapiens cDNA FLJ32189 fis, clone PLACE6002084, weakly similar to CYTOCHROME B561.
Mouse	chr19:9666117-9676472:-	BC058237	BC058237	Mus musculus RIKEN cDNA 2900055D14 gene, mRNA (cDNA clone MGC:68068 IMAGE:6307765), complete cds. CDS=424..912
Mouse	chr19:9676238-9704168:+	G730021J10	G730021J10	unclassifiable
Human	chr11:61316685-61350560:+	CN290841	BC000323	Homo sapiens flap structure-specific endonuclease 1, mRNA (cDNA clone MGC:8478 IMAGE:2821792), complete cds.
Human	chr11:61292708-61316739:-	AW104310	AF070661	Homo sapiens HSPC005 mRNA, complete cds.
Mouse	chr19:9298216-9303250:-	CN717062	I920048F04	flap structure specific endonuclease 1
Mouse	chr19:9303095-9306903:+	BG070542	I530004D22	NEF1 protein homolog [Gallus gallus]
Human	chr11:61316685-61350560:+	CR597752	BC000323	Homo sapiens flap structure-specific endonuclease 1, mRNA (cDNA clone MGC:8478 IMAGE:2821792), complete cds.
Human	chr11:61320475-61322675:-	CN290839	-	-
Mouse	chr19:9298216-9303250:-	BC010203	I920048F04	flap structure specific endonuclease 1
Mouse	chr19:9297448-9301463:+	4932411N02	4932411N02	hypothetical protein
Human	chr11:62189360-62192502:+	BX334837	CR623273	full-length cDNA clone CS0DI078YL03 of Placenta Cot 25-normalized of Homo sapiens (human).
Human	chr11:62186863-62196303:-	AK055736	BC001434	Homo sapiens hypothetical protein MGC2477, mRNA (cDNA clone MGC:2477 IMAGE:3138904), complete cds.
Mouse	chr19:7984583-7988225:-	2610018C13	5730408K05	unclassifiable
Mouse	chr19:7982813-7996971:+	1810009A15	BC039214	Mus musculus RIKEN cDNA 1110055N21 gene, mRNA (cDNA clone MGC:31173 IMAGE:4191047), complete cds. CDS=65..3121
Human	chr11:62231690-62233254:+	AF087900	AF087900	Homo sapiens guanine nucleotide-binding protein gamma-3 subunit mRNA, complete cds.
Human	chr11:62214312-62251520:-	BC009866	AK122942	Homo sapiens cDNA FLJ16651 fis, clone TESTI4036449, highly similar to Mus musculus G protein gamma 3 linked gene.
Mouse	chr19:7936010-7938411:-	6530402F15	6530402F15	guanine nucleotide binding protein (G protein), gamma 3 subunit
Mouse	chr19:7918687-7947764:+	BC061689	BC061689	Mus musculus Bernardinelli-Seip congenital lipodystrophy 2 homolog (human), mRNA (cDNA clone MGC:67749 IMAGE:6306706), complete cds. CDS=271..1602
Human	chr11:62295427-62311390:+	BC008785	BC008785	Homo sapiens TAF6-like RNA polymerase II, p300/CBP-associated factor (PCAF)-associated factor, 65kDa, mRNA (cDNA clone MGC:4288 IMAGE:3629165), complete cds.
Human	chr11:62310071-62311367:-	W91901	-	-
Mouse	chr19:7871606-7885498:-	BC020299	BC020299	Mus musculus TAF6-like RNA polymerase II, p300/CBP-associated factor (PCAF)-associated factor, mRNA (cDNA clone IMAGE:5065101).
Mouse	chr19:7873566-7876043:+	C530024J06	C530024J06	unclassifiable
Human	chr11:63730724-63747939:+	BC015584	BC004347	Homo sapiens, Similar to hypothetical protein FLJ20116, clone MGC:10966 IMAGE:3634032, mRNA, complete cds.
Human	chr11:63747847-63750819:-	BC005133	AY211494	Homo sapiens tRNA splicing 2' phosphotransferase 1 mRNA, complete cds.
Mouse	chr19:6712388-6732899:-	D930046D17	D930046D17	Unc-112 related protein 2 (Kindlin-3)
Mouse	chr19:6709561-6714158:+	C130074I11	C630033L15	tRNA splicing 2' phosphotransferase 1 homolog (S. cerevisiae)
Human	chr11:63794110-63813548:+	BC029297	BC029297	Homo sapiens, clone IMAGE:5174640, mRNA.
Human	chr11:63793876-63808764:-	U66879	U66879	Human Bcl-2 binding component 6 (bbc6) mRNA, complete cds.
Mouse	chr19:6663865-6665087:-	2010209D15	2010209D15	Bcl-associated death promoter
Mouse	chr19:6655291-6665328:+	I920073C12	L37296	Mus musculus (clone 41510) BAD protein mRNA, complete cds. CDS=471..1085
Human	chr11:63815373-63828824:+	CR624550	AF247042	Homo sapiens tandem pore domain potassium channel TRAAK (KCNK4) mRNA, complete cds.
Human	chr11:63827740-63829251:-	CB989594	-	-
Mouse	chr19:6635853-6648105:-	BC048452	AF056492	Mus musculus TRAAK K+ channel subunit mRNA, complete cds. CDS=284..1480
Mouse	chr19:6635616-6636419:+	A430107B04	A430107B04	unclassifiable
Human	chr11:63829581-63840793:+	BC011528	L38487	Human estrogen receptor-related protein (hERRa1) mRNA, 3' end, partial cds.
Human	chr11:63840540-63842132:-	BC029482	AF229068	Homo sapiens HSPC170 protein mRNA, complete cds.
Mouse	chr19:6624407-6635244:-	BC039774	BC039774	Mus musculus estrogen related receptor, alpha, mRNA (cDNA clone IMAGE:4234647), containing frame-shift errors.
Mouse	chr19:6623128-6624479:+	BC016191	BC016191	Mus musculus RIKEN cDNA 0610038D11 gene, mRNA (cDNA clone MGC:27564 IMAGE:4483787), complete cds. CDS=410..784

Human	chr11:64608248-64612450:+	AF001891	AF001891	Homo sapiens clone lambda MEN1 region unknown protein mRNA, complete cds.
Human	chr11:64609027-64613155:-	AK124141	AK124141	Homo sapiens cDNA FLJ42147 fis, clone TESTI4000530.
Mouse	chr19:5851188-5873311:-	BC002119	I730034G08	ANG2 (Chromosome 11 open reading frame2) homolog [Homo sapiens]
Mouse	chr19:5868400-5869985:+	4930470L04	4930470L04	hypothetical protein
Human	chr11:64646231-64651801:+	AB062395	CR600382	full-length cDNA clone CS0DF002YJ11 of Fetal brain of Homo sapiens (human).
Human	chr11:64651322-64658592:-	AB058713	AL834262	Homo sapiens mRNA; cDNA DKFZp761J117 (from clone DKFZp761J117); complete cds.
Mouse	chr19:5841986-5846129:-	I830013P06	I830013P06	mitochondrial ribosomal protein L49
Mouse	chr19:5834943-5842087:+	AK122558	BC057917	Mus musculus RIKEN cDNA 1200010C09 gene, mRNA (cDNA clone MGC:67727 IMAGE:5136398), complete cds. CDS=485..2323
Human	chr11:64705281-64736053:+	BC075862	BC075862	Homo sapiens calpain 1, (mu/l) large subunit, mRNA (cDNA clone MGC:90445 IMAGE:5223130), complete cds.
Human	chr11:64701648-64705926:-	BI517312	-	-
Mouse	chr19:5777448-5804804:-	F730204K23	4021402G16	calpain 1
Mouse	chr19:5801527-5807366:+	3830422M19	3830422M19	unclassifiable
Human	chr11:65049122-65062758:+	AF225424	AF297709	Homo sapiens telomerase regulation-associated protein mRNA, complete cds.
Human	chr11:65062606-65082275:-	AF318354	AK024477	Homo sapiens mRNA for FLJ00070 protein, partial cds.
Mouse	chr19:5547226-5560326:-	G430032F05	G430032F05	SCY1-like 1 (S. cerevisiae)
Mouse	chr19:5529811-5547536:+	1810044G20	L40459	Mus musculus latent transforming growth factor-beta binding protein (LTBP-3) mRNA, complete cds. CDS=157..3918
Human	chr11:65177648-65178343:+	CA439785	-	-
Human	chr11:65177641-65187255:-	BC014095	BC069248	Homo sapiens cDNA clone IMAGE:4123265, containing frame-shift errors.
Mouse	chr19:5435211-5438538:-	F630112B04	F630112B04	unclassifiable
Mouse	chr19:5426390-5437043:+	BC003818	F930025O12	v-rel reticuloendotheliosis viral oncogene homolog A (avian)
Human	chr11:65236065-65243654:+	U67734	U74667	Human tat interactive protein (TIP60) mRNA, complete cds.
Human	chr11:65239046-65244985:-	CR602154	CR602154	full-length cDNA clone CS0DJ012YI19 of T cells (Jurkat cell line) Cot 10-normalized of Homo sapiens (human).
Mouse	chr19:5391564-5399001:-	AB055409	I730060A18	HIV-1 tat interactive protein, homolog (human)
Mouse	chr19:5390782-5392346:+	BU058086	AF346604	Mus musculus AYP1 mRNA, complete cds. CDS=35..535
Human	chr11:65357990-65380943:+	AK055496	AK055496	Homo sapiens cDNA FLJ30934 fis, clone FEBRA2007017, moderately similar to Homo sapiens TRAF4-associated factor 2 mRNA.
Human	chr11:65350423-65397641:-	CR620871	BC018871	Homo sapiens EGF-containing fibulin-like extracellular matrix protein 2, mRNA (cDNA clone IMAGE:3610383).
Mouse	chr19:5281142-5299445:-	9930118N08	9930118N08	cofilin 1, non-muscle
Mouse	chr19:5279291-5284123:+	C920029L04	C920029L04	cofilin 1, non-muscle
Human	chr11:65381173-65391700:+	AK126820	AK126820	Homo sapiens cDNA FLJ44872 fis, clone BRAMY2022320.
Human	chr11:65350423-65397641:-	BE392698	BC018871	Homo sapiens EGF-containing fibulin-like extracellular matrix protein 2, mRNA (cDNA clone IMAGE:3610383).
Mouse	chr19:5271465-5277309:-	AF425647	BC026560	Mus musculus RIKEN cDNA 1200008A18 gene, mRNA (cDNA clone MGC:36246 IMAGE:5038349), complete cds. CDS=228..1883
Mouse	chr19:5274285-5276134:+	D030001O06	D030001O06	unclassifiable
Human	chr11:66140643-66192432:+	AK094046	AF315632	Homo sapiens coactivator activator mRNA, complete cds, alternatively spliced.
Human	chr11:66189043-66201959:-	AL050172	BC003503	Homo sapiens, Similar to RIKEN cDNA 4921506I22 gene, clone MGC:10380 IMAGE:3345085, mRNA, complete cds.
Mouse	chr19:4550573-4556297:-	A430072G02	A430072G02	unclassifiable
Mouse	chr19:4544926-4554415:+	BC019488	3110006N17	hypothetical RNA-binding region RNP-1 (RNA recognition motif) containing protein
Human	chr11:66841888-66922459:+	CR624269	U53174	Human cell cycle checkpoint control protein mRNA, complete cds.
Human	chr11:66922224-66945230:-	AK098311	AK127616	Homo sapiens cDNA FLJ45714 fis, clone FEKID2002637, highly similar to Serine/threonine protein phosphatase PP1-alpha 1 catalytic subunit (EC 3.1.3.16).
Mouse	chr19:3983661-4046149:-	G730002C09	G730002C09	RAD9 homolog (S. pombe)
Mouse	chr19:3980535-3983895:+	3830432B13	I830031O16	protein phosphatase 1, catalytic subunit, alpha isoform
Human	chr11:66940243-66949654:+	AB037815	AB037815	Homo sapiens mRNA for KIAA1394 protein, partial cds.
Human	chr11:66922224-66945230:-	AK127616	AK127616	Homo sapiens cDNA FLJ45714 fis, clone FEKID2002637, highly similar to Serine/threonine protein phosphatase PP1-alpha 1 catalytic subunit (EC 3.1.3.16).
Mouse	chr19:3952794-3981252:-	E430025F20	E430025F20	Hypothetical Microbodies C-terminal targeting signal/RabGAP/TBC domain containing protein homolog [Mus musculus]
Mouse	chr19:3958036-3962754:+	E130003F03	E130003F03	unclassifiable
Human	chr11:69165054-69178425:+	BC023620	X59798	Human PRAD1 mRNA for cyclin.
Human	chr11:69176881-69199365:-	AK124252	AF503940	Homo sapiens TAOS1 (ORAOV1) mRNA, complete cds.
Mouse	chr7:132640514-132641487:+	BM236767	-	-
Mouse	chr7:132640514-132650815:-	E130112I22	E130112C01	cyclin D1
Human	chr11:71469030-71499476:+	AF289588	BC012855	Homo sapiens, Similar to RIKEN cDNA 1700008D07 gene, clone MGC:9830 IMAGE:3863323, mRNA, complete cds.
Human	chr11:71495974-71501474:-	CR603805	AL080071	Homo sapiens mRNA; cDNA DKFZp564M082 (from clone DKFZp564M082); complete cds.
Mouse	chr7:89243925-89249337:-	F930017I19	F930017I19	unclassifiable
Mouse	chr7:89209258-89247401:+	6330414C15	BC006736	Mus musculus RIKEN cDNA 3200002M19 gene, mRNA (cDNA clone MGC:11592 IMAGE:3964006), complete cds. CDS=141..539

Human	chr11:71612393-71627839:+	Y14385	Y14385	Homo sapiens mRNA for inositol polyphosphate 5-phosphatase.
Human	chr11:71627769-71634356:-	CR590341	BC041564	Homo sapiens, Similar to aristaless homeobox (Drosophila), clone MGC:52227 IMAGE:5923445, mRNA, complete cds.
Mouse	chr7:89167972-89183734:-	BC049961	AF162781	Mus musculus SH2-containing inositol 5-phosphatase 2 mRNA, complete cds. CDS=388..4161
Mouse	chr7:89163652-89168069:+	X75014	X75014	M.musculus Phox2 mRNA for homeodomain protein. CDS=28..870
Human	chr11:93034525-93103172:+	AK057804	AB051518	Homo sapiens mRNA for KIAA1731 protein, partial cds.
Human	chr11:93103008-93157205:-	AK057183	AK027016	Homo sapiens cDNA: FLJ23363 fis, clone HEP15507.
Mouse	chr9:15156585-15191539:-	5830418K08	5830418K08	hypothetical protein
Mouse	chr9:15145886-15156668:+	4921518I16	G830041A09	hypothetical protein
Human	chr11:105452179-105474647:+	AF302110	AF302110	Homo sapiens alpha-aminoadipic semialdehyde dehydrogenase-phosphopantetheinyl transferase mRNA, complete cds.
Human	chr11:105427035-105453675:-	BI829778	BX640672	Homo sapiens mRNA; cDNA DKFZp686D14128 (from clone DKFZp686D14128); complete cds.
Mouse	chr9:4217340-4232189:-	BG404618	G730021M24	aminoadipate-semialdehyde dehydrogenase-phosphopantetheinyl transferase
Mouse	chr9:4232088-4254280:+	BU939765	8030404M02	hypothetical BTB/POZ domain/Microbodies C-terminal targeting signal/Kelch repeat containing protein
Human	chr11:107384823-107483712:+	CN369245	AF017061	Homo sapiens vasopressin-activated calcium mobilizing putative receptor protein (VACM-1) mRNA, complete cds.
Human	chr11:107384617-107385205:-	BG031574	-	-
Mouse	chr9:53769003-53821574:-	6330509I08	B230334O19	VASOPRESSIN-ACTIVATED CALCIUM-MOBILIZING RECEPTOR (VACM-1) (CULLIN HOMOLOG 5) (CUL-5) homolog [Rattus norvegicus]
Mouse	chr9:53821173-53835638:+	F930028P13	A630042J09	unclassifiable
Human	chr11:110978380-111106789:+	AB096248	AB096248	Homo sapiens LOH11CR1I gene, loss of heterozygosity, 11, chromosomal region 1 gene I product.
Human	chr11:111100988-111102649:-	BM511599	-	-
Mouse	chr9:51046453-51169449:-	G630080D20	G630080D20	weakly similar to PROBABLE SERINE/THREONINE PROTEIN KINASE SNF1LK (EC 2.7.1.-) (SALT- INDUCIBLE PROTEIN KINASE) (PROTEIN KINASE KID2) [Rattus norvegicus]
Mouse	chr9:51005901-51054943:+	4930534B21	4930534B21	Serine/threonine protein phosphatase 2A
Human	chr11:110978380-111106789:+	AB096248	AB096248	Homo sapiens LOH11CR1I gene, loss of heterozygosity, 11, chromosomal region 1 gene I product.
Human	chr11:111102847-111142379:-	BC027596	BC027596	Homo sapiens protein phosphatase 2 (formerly 2A), regulatory subunit A (PR 65), beta isoform, transcript variant 2, mRNA (cDNA clone MGC:26454 IMAGE:4831056), complete cds.
Mouse	chr9:51046453-51169449:-	G630080D20	G630080D20	weakly similar to PROBABLE SERINE/THREONINE PROTEIN KINASE SNF1LK (EC 2.7.1.-) (SALT- INDUCIBLE PROTEIN KINASE) (PROTEIN KINASE KID2) [Rattus norvegicus]
Mouse	chr9:51005901-51054943:+	4930534B21	4930534B21	Serine/threonine protein phosphatase 2A
Human	chr11:111254868-111261908:+	BC036892	AK027152	Homo sapiens cDNA: FLJ23499 fis, clone LNG02702.
Human	chr11:111249990-111289126:-	AK097483	AK097483	Homo sapiens cDNA FLJ40164 fis, clone TESTI2015914.
Mouse	chr9:50921969-50936642:-	A430061I10	A430061I10	weakly similar to CDNA: FLJ23499 FIS, CLONE LNG02702 [Homo sapiens]
Mouse	chr9:50928832-50933944:+	6430547E06	6430547E06	hypothetical Ferredoxin-fold anticodon binding domain containing protein
Human	chr11:111450020-111461086:+	BP872654	BX538107	Homo sapiens mRNA; cDNA DKFZp686K15145 (from clone DKFZp686K15145); complete cds.
Human	chr11:111439944-111450142:-	AA187445	CR622263	full-length cDNA clone CS0DI071YF24 of Placenta Cot 25-normalized of Homo sapiens (human).
Mouse	chr9:50765831-50778058:-	I220011B04	BC051390	Mus musculus cDNA clone IMAGE:4986186, partial cds.
Mouse	chr9:50777915-50785594:+	BC039645	BC039645	Mus musculus RIKEN cDNA 2700059L22 gene, mRNA (cDNA clone MGC:49408 IMAGE:5373181), complete cds. CDS=296..1243
Human	chr11:111450020-111461086:+	AB096251	BX538107	Homo sapiens mRNA; cDNA DKFZp686K15145 (from clone DKFZp686K15145); complete cds.
Human	chr11:111458666-111462717:-	BX431124	AK025397	Homo sapiens cDNA: FLJ21744 fis, clone COLF4965.
Mouse	chr9:50765831-50778058:-	BC051390	BC051390	Mus musculus cDNA clone IMAGE:4986186, partial cds.
Mouse	chr9:50764460-50774792:+	1110003M24	AF196314	Mus musculus small zinc finger-like protein DDP2 (Ddp2) mRNA, complete cds. CDS=45..296
Human	chr11:116511828-116586223:+	AK126420	AK126420	Homo sapiens cDNA FLJ44456 fis, clone UTERU2024002.
Human	chr11:116578548-116608451:-	BT006870	BC010696	Homo sapiens, proprotein convertase subtilisin/kexin type 7, clone MGC:17703 IMAGE:3868257, mRNA, complete cds.
Mouse	chr9:46031939-46038371:-	5031408O12	5031408O12	transgelin
Mouse	chr9:46008810-46032132:+	U48830	BC056456	Mus musculus proprotein convertase subtilisin/kexin type 7, mRNA (cDNA clone IMAGE:5715740), containing frame-shift errors.
Human	chr11:116667342-116668096:+	CB960709	-	-
Human	chr11:116661612-116692182:-	AF338816	AF201468	Homo sapiens APP beta-secretase mRNA, complete cds.
Mouse	chr9:45960604-45963414:-	CB590035	-	-
Mouse	chr9:45940728-45966645:+	C230026O08	C230037E16	beta-site APP cleaving enzyme
Human	chr11:116668724-116669497:+	BQ007699	-	-
Human	chr11:116661612-116692182:-	AF338816	AF201468	Homo sapiens APP beta-secretase mRNA, complete cds.
Mouse	chr9:45958999-45960581:-	9030618A07	9030618A07	unclassifiable
Mouse	chr9:45940728-45966645:+	C230026O08	C230037E16	beta-site APP cleaving enzyme
Human	chr11:117209644-117214867:+	CA941380	-	-
Human	chr11:117176764-117253355:-	AK055389	CR617323	full-length cDNA clone CS0DF011YP12 of Fetal brain of Homo sapiens (human).
Mouse	chr9:45497345-45497933:-	2310024F14	2310024F14	unclassifiable
Mouse	chr9:45471912-45497942:+	BC042579	BC051127	Mus musculus FXYD domain-containing ion transport regulator 6, mRNA (cDNA clone MGC:58114 IMAGE:6531451), complete cds. CDS=84..365

Human	chr11:117735509-117785776:+	BC039240	D50916	Human mRNA for KIAA0126 gene, complete cds.
Human	chr11:117774831-117777820:-	BU538441	-	-
Mouse	chr9:45017338-45024839:-	1500041B09	1500041B09	ATP synthase, H+ transporting, mitochondrial F0 complex, subunit g
Mouse	chr9:45024295-45025535:+	4933437C06	4933437C06	unclassifiable
Human	chr11:118259777-118272190:+	X68149	X68149	Homo sapiens BLR1 gene for Burkitt's lymphoma receptor 1.
Human	chr11:118272055-118301832:-	CR606474	AB094091	Homo sapiens DLNB11 mRNA, complete cds.
Mouse	chr9:44615761-44665852:-	4930565I22	4930565I22	Burkitt lymphoma receptor 1
Mouse	chr9:44586644-44615881:+	CO044513	F730201L05	DLNB11 protein homolog [Homo sapiens]
Human	chr11:118301083-118334478:+	BG489265	Y13645	Homo sapiens mRNA for uroplakin II.
Human	chr11:118272055-118301832:-	CN369702	AB094091	Homo sapiens DLNB11 mRNA, complete cds.
Mouse	chr9:44585489-44587778:-	D330004E16	D330004E16	unclassifiable
Mouse	chr9:44586644-44615881:+	M5H1102D20	F730201L05	DLNB11 protein homolog [Homo sapiens]
Human	chr11:118374058-118391713:+	AB094093	AB094093	Homo sapiens DLNB14 mRNA, complete cds.
Human	chr11:118391225-118394610:-	CR620392	M64716	Human ribosomal protein S25 mRNA, complete cds.
Mouse	chr9:44514134-44522543:-	BC055929	BC055929	Mus musculus cDNA clone IMAGE:4205568, partial cds. CDS=2..1018
Mouse	chr9:44511444-44514402:+	2810009D21	2810009D21	ribosomal protein S25
Human	chr11:118394352-118401372:+	CR616613	AK025623	Homo sapiens cDNA: FLJ21970 fis, clone HEP05733, highly similar to AF161520 Homo sapiens HSPC172 mRNA.
Human	chr11:118391225-118394610:-	BM768873	M64716	Human ribosomal protein S25 mRNA, complete cds.
Mouse	chr9:44507728-44511542:-	BC038898	BC038898	Mus musculus trafficking protein particle complex 4, mRNA (cDNA clone MGC:49061 IMAGE:5401311), complete cds. CDS=248..907
Mouse	chr9:44511444-44514402:+	CN840764	2810009D21	ribosomal protein S25
Human	chr11:118756662-118757501:+	AA405140	-	-
Human	chr11:118731134-118757631:-	BC041366	BC041366	Homo sapiens ubiquitin specific protease 2, mRNA (cDNA clone MGC:43844 IMAGE:5273400), complete cds.
Mouse	chr9:44168670-44216050:-	D430033O10	D430033O10	RING FINGER PROTEIN WITH LEUCINE ZIPPER RNF26 (RESERVED) homolog [Homo sapiens]
Mouse	chr9:44169365-44198119:+	BG294999	AF079565	Mus musculus ubiquitin-specific protease UBP41 (Ubp41) mRNA, complete cds. CDS=1..1062
Human	chr11:124048950-124073418:+	AF334735	AF334735	Homo sapiens sperm protein 17 mRNA, complete cds.
Human	chr11:124010895-124051409:-	AF300796	AF300796	Homo sapiens sialic acid-specific 9-O-acetylesterase I mRNA, complete cds.
Mouse	chr9:37618161-37628591:-	Z46299	Z46299	M.musculus Sp17 gene for sperm specific protein. CDS=95..544
Mouse	chr9:37570609-37663928:+	B230039I06	AF156856	Mus musculus cytosolic sialic acid 9-O-acetylesterase mRNA, complete cds. CDS=507..1841
Human	chr11:125658183-125672687:+	BC032474	AF410783	Homo sapiens TIR domain-containing adaptor wyatt mRNA, complete cds.
Human	chr11:125668027-125669359:-	BX508466	-	-
Mouse	chr9:35156418-35172428:-	9830107B17	A530066P14	toll-interleukin 1 receptor (TIR) domain-containing adaptor protein
Mouse	chr9:35160692-35165846:+	BI455134	-	-
Human	chr11:129377729-129380591:+	AK055956	AK055956	Homo sapiens cDNA FLJ31394 fis, clone NT2NE1000120.
Human	chr11:129274812-129377940:-	AL833172	BX648944	Homo sapiens mRNA; cDNA DKFZp686C1648 (from clone DKFZp686C1648).
Mouse	chr9:31261509-31264480:-	4930517E14	4930517E14	unclassifiable
Mouse	chr9:31264026-31313709:+	BC064128	BC064128	Mus musculus PR domain containing 10, mRNA (cDNA clone MGC:73898 IMAGE:3975690), complete cds. CDS=202..1227
Human	chr11:133444140-133527777:+	CR609180	AK074769	Homo sapiens cDNA FLJ90288 fis, clone NT2RP1000981, weakly similar to CELL SURFACE A33 ANTIGEN PRECURSOR.
Human	chr11:133525229-133600282:-	AI557215	AK124878	Homo sapiens cDNA FLJ42888 fis, clone BRHIP3008183.
Mouse	chr9:27046730-27106174:-	5730470H08	BC024357	Mus musculus junction adhesion molecule 3, mRNA (cDNA clone MGC:35813 IMAGE:4973185), complete cds. CDS=28..960
Mouse	chr9:26981882-27046813:+	BC033607	BC048190	Mus musculus RIKEN cDNA B130055D15 gene, mRNA (cDNA clone MGC:61361 IMAGE:6831876), complete cds. CDS=473..4144
Human	chr12:368846-422072:+	BI913313	AK027638	Homo sapiens cDNA FLJ14732 fis, clone NT2RP3001969, weakly similar to TRICHOHYALIN.
Human	chr12:200049-368978:-	CD557459	BC022392	Homo sapiens, clone MGC:24098 IMAGE:4612245, mRNA, complete cds.
Mouse	chr6:120723289-120763671:-	L930260P17	2400002C23	hypothetical protein
Mouse	chr6:120763396-120841617:+	A630021G15	BC011506	Mus musculus retinoblastoma binding protein 2, mRNA (cDNA clone IMAGE:4009470), with apparent retained intron.
Human	chr12:6186429-6511393:+	D63880	D63880	Human mRNA for KIAA0159 gene, complete cds.
Human	chr12:6471313-6473268:-	CR624922	CR624922	full-length cDNA clone CS0DC012YA04 of Neuroblastoma Cot 25-normalized of Homo sapiens (human).
Mouse	chr6:125784931-125809423:-	L930140P11	F830009F01	Condensin subunit 1 (Chromosome condensation-related SMC-associated protein 1) (Chromosome-associated protein D2) (mCAP-D2) (XCAP-D2 homolog)
Mouse	chr6:125809107-125811921:+	CN838463	2610511M02	mitochondrial ribosomal protein L51
Human	chr12:6846544-6850528:+	M10036	M10036	Human triosephosphate isomerase mRNA, complete cds.
Human	chr12:6850360-6868783:-	BC002983	BC002983	Homo sapiens, Similar to gene rich cluster, C9 gene, clone MGC:2519 IMAGE:3546861, mRNA, complete cds.
Mouse	chr6:125429229-125432939:-	2410143L05	2410143L05	triosephosphate isomerase
Mouse	chr6:125427304-125429262:+	BC010305	BC002005	Mus musculus gene rich cluster, C9 gene, mRNA (cDNA clone MGC:5776 IMAGE:3496382), complete cds. CDS=185..979

Human	chr12:8957759-8985330:+	BC073964	BC073964	Homo sapiens polyhomeotic-like 1 (Drosophila), mRNA (cDNA clone MGC:87926 IMAGE:5788132), complete cds.
Human	chr12:8983919-8993818:-	AA130765	CR457412	Homo sapiens full open reading frame cDNA clone RZPDo834D0814D for gene M6PR, mannose-6-phosphate receptor (cation dependent); complete cds, incl. stopcodon.
Mouse	chr6:122949300-122971981:-	U63386	4922505F11	polyhomeotic-like 1 (Drosophila)
Mouse	chr6:122939331-122949394:+	X64070	X64070	M.musculus gene for cation-dependent mannose-6-phosphate receptor. CDS=4487..8694
Human	chr12:21481854-21515453:+	AK095813	BC021662	Homo sapiens, hypothetical protein FLJ22028, clone MGC:22177 IMAGE:3826938, mRNA, complete cds.
Human	chr12:21513108-21545816:-	AY157499	L36140	Homo sapiens (clone 1311) DNA helicase (RECQL) mRNA, complete cds.
Mouse	chr6:142492318-142515267:+	G370139B07	G370139B07	hypothetical FAD-dependent pyridine nucleotide-disulphide oxidoreductase containing protein
Mouse	chr6:142497007-142535726:-	AB017105	AB017105	Mus musculus gene for DNA helicase Q1, complete cds. CDS=52..1947
Human	chr12:24946510-24948882:+	AK124863	AK124863	Homo sapiens cDNA FLJ42873 fis, clone BRHIP2026061.
Human	chr12:24855545-24993660:-	AL701736	AK056255	Homo sapiens cDNA FLJ31693 fis, clone NT2RI2005710, highly similar to BRANCHED-CHAIN AMINO ACID AMINOTRANSFERASE, CYTOSOLIC (EC 2.6.1.42).
Mouse	chr6:145207550-145210421:+	C230081E04	C230081E04	unclassifiable
Mouse	chr6:145158893-145236522:-	9630055I01	I730071G10	branched chain aminotransferase 1, cytosolic
Human	chr12:25096473-25152538:+	CR601375	CR601375	full-length cDNA clone CS0DN003YJ06 of Adult brain of Homo sapiens (human).
Human	chr12:25141069-25239361:-	BC047415	BC047415	Homo sapiens, hypothetical protein FLJ10921, clone IMAGE:5269578, mRNA.
Mouse	chr6:145275627-145334928:+	BC052909	E430001H03	lymphoid-restricted membrane protein
Mouse	chr6:145334825-145371027:-	4930513G12	AY423542	Mus musculus lung adenoma susceptibility 1 (Las1) mRNA, complete cds. CDS=93..2285
Human	chr12:25239411-25255925:+	CR601350	AK057730	Homo sapiens cDNA FLJ25001 fis, clone CBL00443.
Human	chr12:25248990-25295132:-	M54968	M54968	Human K-ras oncogene protein mRNA, complete cds.
Mouse	chr6:145371138-145377525:+	K430032N20	AF412300	Mus musculus clone 58 growth hormone-inducible soluble protein mRNA, complete cds. CDS=151..411
Mouse	chr6:145376690-145410230:-	BC014700	F630119B11	Kirsten rat sarcoma oncogene 2, expressed
Human	chr12:28223477-28625711:+	BI086262	AX747957	Sequence 1482 from Patent EP1308459.
Human	chr12:28207498-28234946:-	BC048978	BC048978	Homo sapiens, clone IMAGE:4830713, mRNA.
Mouse	chr6:147606759-147764244:+	1810060J02	1810060J02	similar to CDNA FLJ11088 FIS, CLONE PLACE1005287, WEAKLY SIMILAR TO INNER CENTROMERE PROTEIN [Homo sapiens]
Mouse	chr6:147568440-147607174:-	D930040L07	G730037D21	hypothetical protein
Human	chr12:29193280-29385292:+	BI019319	AL136843	Homo sapiens mRNA; cDNA DKFZp434C0730 (from clone DKFZp434C0730); complete cds.
Human	chr12:29381552-29425436:-	AL834128	AL834128	Homo sapiens mRNA; cDNA DKFZp451J152 (from clone DKFZp451J152); complete cds.
Mouse	chr6:148187630-148329115:+	A830011B04	A830011B04	weakly similar to MALE STERILITY PROTEIN 2-LIKE PROTEIN [Torpedo marmorata]
Mouse	chr6:148324041-148373655:-	4932416O14	4932416O14	PTX1 PROTEIN homolog [Homo sapiens]
Human	chr12:43896125-44112401:+	AW452627	AL832340	Homo sapiens mRNA; cDNA DKFZp451M105 (from clone DKFZp451M105).
Human	chr12:43853084-43897271:-	AK123755	AF103731	Homo sapiens putative glycolipid transfer protein mRNA, complete cds.
Mouse	chr15:96219216-96405053:+	G730005E18	F730003B03	hypothetical protein
Mouse	chr15:96216277-96220063:-	A130051J06	A130051J06	hypothetical protein
Human	chr12:45759653-45916713:+	BC072670	BC072670	Homo sapiens hypothetical protein MGC16044, mRNA (cDNA clone IMAGE:5726703).
Human	chr12:45755757-45760001:-	BC047595	AY454159	Homo sapiens DEGA/AMIGO-2 precursor, mRNA, complete cds.
Mouse	chr15:97688057-97834923:+	D130052A12	D130052A12	CDK2 (cyclin-dependent kinase 2)-associated protein 1
Mouse	chr15:97684973-97688296:-	A230062J08	A230062J08	hypothetical Leucine-rich repeat, outliers/Leucine-rich repeat/Immunoglobulin and major histocompatibility complex domain/Immunoglobulin-like/Immunoglobulin subtype/Leucine-rich repeat, typical subtype containing protein
Human	chr12:46397641-46422343:+	AL831948	AL831948	Homo sapiens mRNA; cDNA DKFZp761B0218 (from clone DKFZp761B0218).
Human	chr12:46417108-46439128:-	U78168	AK092448	Homo sapiens cDNA FLJ35129 fis, clone PLACE6008775, highly similar to Homo sapiens Rap1 guanine-nucleotide exchange factor mRNA.
Mouse	chr15:98208900-98210194:+	A130041K16	A130041K16	hypothetical protein
Mouse	chr15:98203977-98227307:-	6720450F13	BC020532	Mus musculus RIKEN cDNA 9330170P05 gene, mRNA (cDNA clone MGC:19192 IMAGE:4236136), complete cds. CDS=405..3035
Human	chr12:46433966-46462803:+	BI761530	BC002759	Homo sapiens hypothetical protein FLJ20489, mRNA (cDNA clone IMAGE:3632656), complete cds.
Human	chr12:46417108-46439128:-	AK092448	AK092448	Homo sapiens cDNA FLJ35129 fis, clone PLACE6008775, highly similar to Homo sapiens Rap1 guanine-nucleotide exchange factor mRNA.
Mouse	chr15:98219447-98230393:+	2310016P22	6820415M06	Rap guanine nucleotide exchange factor (GEF) 3
Mouse	chr15:98203977-98227307:-	6720450F13	BC020532	Mus musculus RIKEN cDNA 9330170P05 gene, mRNA (cDNA clone MGC:19192 IMAGE:4236136), complete cds. CDS=405..3035
Human	chr12:46433966-46462803:+	AK025856	BC002759	Homo sapiens hypothetical protein FLJ20489, mRNA (cDNA clone IMAGE:3632656), complete cds.
Human	chr12:46462760-46500623:-	AK026767	BC064840	Homo sapiens histone deacetylase 7A, mRNA (cDNA clone MGC:74915 IMAGE:6179239), complete cds.
Mouse	chr15:98237637-98251897:+	F730227G04	F730025G12	hypothetical protein
Mouse	chr15:98251866-98304451:-	A930005K21	I730050J12	histone deacetylase 7A
Human	chr12:47678417-47699285:+	CR591575	CR591575	full-length cDNA clone CS0DC022YB16 of Neuroblastoma Cot 25-normalized of Homo sapiens (human).
Human	chr12:47675198-47679460:-	CR591956	CR591956	full-length cDNA clone CS0DK002YG07 of HeLa cells Cot 25-normalized of Homo sapiens (human).
Mouse	chr15:99267602-99276758:+	B130046B21	B130046B21	hypothetical protein
Mouse	chr15:99231937-99270065:-	K430359L18	U20658	Mus musculus (wnt-10b) mRNA, complete cds. CDS=149..1318

Human	chr12:47755634-47953388:+	BU615242	CR624182	full-length cDNA clone CS0DF001YP06 of Fetal brain of Homo sapiens (human).
Human	chr12:47807367-47953384:-	CR611979	CR611979	full-length cDNA clone CS0DF026YM18 of Fetal brain of Homo sapiens (human).
Mouse	chr15:99411505-99499683:+	I1C0015P17	I1C0015P17	tubulin, alpha 6
Mouse	chr15:99392854-99499673:-	BM232856	I0C0037H23	tubulin, alpha 1
Human	chr12:47770306-47773362:+	BG718375	-	-
Human	chr12:47769475-47774869:-	BC033507	BC033507	Homo sapiens, Similar to desert hedgehog homolog (Drosophila), clone MGC:35145 IMAGE:5169286, mRNA, complete cds.
Mouse	chr15:99354962-99356789:+	5830455I15	5830455I15	unclassifiable
Mouse	chr15:99352766-99360179:-	6430555F24	6430555F24	desert hedgehog homolog, (Drosophila)
Human	chr12:48784015-48791651:+	L34041	BC032234	Homo sapiens, glycerol-3-phosphate dehydrogenase 1 (soluble), clone MGC:34464 IMAGE:5229925, mRNA, complete cds.
Human	chr12:48787652-48788508:-	CA944404	-	-
Mouse	chr15:100178185-100186245:+	0610008N20	9530001I16	glycerol-3-phosphate dehydrogenase 1 (soluble)
Mouse	chr15:100176658-100185594:-	C130048I16	C130048I16	unclassifiable
Human	chr12:51727020-51744430:+	BX647126	AF417490	Homo sapiens tensin2 mRNA, complete cds.
Human	chr12:51744367-51759455:-	BM997053	AK074694	Homo sapiens cDNA FLJ90213 fis, clone MAMMA1002142, weakly similar to NON-RECEPTOR TYROSINE KINASE SPORE LYSIS A (EC 2.7.1.112).
Mouse	chr15:102578113-102593934:+	BC025818	AF424789	Mus musculus tensin 2 mRNA, complete cds. CDS=39..4184
Mouse	chr15:102593876-102613765:-	I920042B03	I920042B03	hypothetical SPla/RYanodine receptor SPRY/Gonadotropin-releasing hormone containing protein
Human	chr12:51860755-51870921:+	BC053557	BC053557	Homo sapiens cDNA clone MGC:61706 IMAGE:6162269, complete cds.
Human	chr12:51837714-51861402:-	BQ679860	AK126934	Homo sapiens cDNA FLJ44987 fis, clone BRAWH3005892.
Mouse	chr15:102680779-102693136:+	G730025D22	G730025D22	OriLyt TD-element binding protein 7 (Hypothetical protein) homolog [Homo sapiens]
Mouse	chr15:102654528-102682254:-	BI454620	1300015E02	deoxyribonuclease II alpha
Human	chr12:52121656-52161217:+	AK130583	AB188306	Homo sapiens PCBP2 mRNA for poly(rC) binding protein 2, complete cds.
Human	chr12:52160543-52180114:-	BC050050	BC050050	Homo sapiens, Similar to mitogen activated protein kinase kinase kinase 12, clone MGC:42552 IMAGE:4824512, mRNA, complete cds.
Mouse	chr15:102952023-102981931:+	5031415E16	5031415E16	poly(rC) binding protein 2
Mouse	chr15:102979510-102998927:-	4931421L15	4931421L15	mitogen activated protein kinase kinase kinase 12
Human	chr12:52164254-52165369:+	CN354487	-	-
Human	chr12:52160543-52180114:-	BC037585	BC050050	Homo sapiens, Similar to mitogen activated protein kinase kinase kinase 12, clone MGC:42552 IMAGE:4824512, mRNA, complete cds.
Mouse	chr15:102984336-102986865:+	BG066357	-	-
Mouse	chr15:102979510-102998927:-	U23789	4931421L15	mitogen activated protein kinase kinase kinase 12
Human	chr12:52913278-52914219:+	BF964791	-	-
Human	chr12:52910991-52960222:-	BU175488	CR605673	full-length cDNA clone CS0DC003YP05 of Neuroblastoma Cot 25-normalized of Homo sapiens (human).
Mouse	chr15:103675012-103679337:+	AF216290	AF216290	Mus musculus heterochromatin protein 1 alpha mRNA, complete cds. CDS=67..642
Mouse	chr15:103632300-103724659:-	E430008N04	BC004707	Mus musculus chromobox homolog 5 (Drosophila HP1a), mRNA (cDNA clone MGC:5952 IMAGE:3499489), complete cds. CDS=131..706
Human	chr12:54361686-54364662:+	AY358508	BC020509	Homo sapiens, Similar to RIKEN cDNA 0610006F02 gene, clone MGC:17301 IMAGE:3849188, mRNA, complete cds.
Human	chr12:54364619-54392462:-	AK057949	BC050280	Homo sapiens integrin, alpha 7, mRNA (cDNA clone MGC:33821 IMAGE:5284443), complete cds.
Mouse	chr10:129086124-129088917:-	BF234014	0610006F02	hypothetical S-adenosyl-L-methionine-dependent methyltransferases structure containing protein
Mouse	chr10:129061619-129086167:+	L23423	L23423	Mouse (BALB/c) alpha-7 integrin mRNA, complete cds. CDS=175..3582
Human	chr12:54497970-54504139:+	BC012543	AB064961	Homo sapiens mRNA for adoplin-2, complete cds.
Human	chr12:54432628-54510832:-	AY188447	AF161434	Homo sapiens HSPC316 mRNA, partial cds.
Mouse	chr10:128944939-128948667:-	0610012C09	0610012C09	hypothetical protein
Mouse	chr10:128931099-128946485:+	C230066C20	C230066C20	DOPAMINE RECEPTOR INTERACTING PROTEIN homolog [Rattus norvegicus]
Human	chr12:54509796-54516309:+	AY358778	AK095340	Homo sapiens cDNA FLJ38021 fis, clone CTONG2012847.
Human	chr12:54515482-54523034:-	U38320	BC030206	Homo sapiens, Similar to matrix metalloproteinase 19, clone IMAGE:5209132, mRNA.
Mouse	chr10:128916466-128932480:-	G630062L16	G630062L16	unclassifiable
Mouse	chr10:128917947-128927864:+	F630109P11	F630109P11	matrix metalloproteinase 19
Human	chr12:54798283-54804801:+	BC018708	AK027357	Homo sapiens cDNA FLJ14451 fis, clone HEMBB1001834.
Human	chr12:54800741-54808905:-	BI909810	-	-
Mouse	chr10:128668998-128674811:-	BC022677	6030405G22	hypothetical Zinc finger C-x8-C-x5-C-x3-H type containing protein
Mouse	chr10:128671349-128672320:+	CA466466	-	-
Human	chr12:54910110-54917901:+	AK172768	AK172768	Homo sapiens cDNA FLJ23929 fis, clone COL05747.
Human	chr12:54917855-54938427:-	AK056831	AK125379	Homo sapiens cDNA FLJ43389 fis, clone OCBBF2007068, weakly similar to Bos taurus ankyrin 1.
Mouse	chr10:128522697-128527993:-	2010205A06	BC028990	Mus musculus RIKEN cDNA 1810013D05 gene, mRNA (cDNA clone MGC:32429 IMAGE:5041383), complete cds. CDS=180..1787
Mouse	chr10:128503891-128522739:+	A330022I22	G431002C21	hypothetical Ankyrin-repeat containing protein

Human	chr12:54980181-54994853:+	AW118194	-	-
Human	chr12:54951750-54996399:-	CR596106	BX640838	Homo sapiens mRNA; cDNA DKFZp686D21216 (from clone DKFZp686D21216); complete cds.
Mouse	chr10:128463909-128464767:-	9030605P22	9030605P22	unclassifiable
Mouse	chr10:128464491-128489260:+	G530115E17	G530115E17	citrate synthase
Human	chr12:55148607-55151036:+	AK074733	AL832247	Homo sapiens mRNA; cDNA DKFZp686N0877 (from clone DKFZp686N0877).
Human	chr12:55150995-55168448:-	BC036929	BC048344	Homo sapiens, Similar to liver mitochondrial glutaminase, clone IMAGE:5243009, mRNA.
Mouse	chr10:128337176-128339099:-	BC014757	4633402N23	hypothetical SPla and the RYanodine Receptor (SPRY)/SPRY domain/Domain in various gamma-carboxylases and other proteins containing protein
Mouse	chr10:128321499-128337220:+	I920050I17	G730015M24	glutaminase 2 (liver, mitochondrial)
Human	chr12:55768963-55775526:+	X70991	BC065931	Homo sapiens cDNA clone MGC:75085 IMAGE:6157017, complete cds.
Human	chr12:55775454-55791455:-	BC075852	BC004973	Homo sapiens, signal transducer and activator of transcription 6, interleukin-4 induced, clone MGC:3649 IMAGE:2958389, mRNA, complete cds.
Mouse	chr10:127785473-127793125:-	U47543	U47543	Mus musculus NGFI-A binding protein 2 (NAB2) mRNA, complete cds. CDS=221..1798
Mouse	chr10:127767511-127785544:+	BM122652	C730038I05	signal transducer and activator of transcription 6
Human	chr12:55808543-55859881:+	BC045107	BC021204	Homo sapiens cDNA clone IMAGE:4110815, complete cds.
Human	chr12:55824670-55827669:-	BM714117	-	-
Mouse	chr10:127662656-127745636:-	I830174K15	G530110P14	low density lipoprotein receptor-related protein 1
Mouse	chr10:127727939-127730623:+	BI411066	-	-
Human	chr12:55909720-55914985:+	BC008711	BC011911	Homo sapiens serine hydroxymethyltransferase 2 (mitochondrial), mRNA (cDNA clone MGC:20325 IMAGE:4139001), complete cds.
Human	chr12:55914952-55920794:-	AF164796	AK094275	Homo sapiens cDNA FLJ36956 fis, clone BRACE2005869, moderately similar to Homo sapiens NADH:ubiquinone oxidoreductase MLRQ subunit mRNA.
Mouse	chr10:127641607-127646939:-	I920089B02	D130016H13	SERINE HYDROXYMETHYLTRANSFERASE (EC 2.1.2.1) (SERINE METHYLASE) (GLYCINE HYDROXYMETHYLTRANSFERASE) (SHMT)
Mouse	chr10:127639423-127641638:+	BC064011	1110007I24	NADH:ubiquinone oxidoreductase MLRQ subunit homolog (NUOMS) homolog [Homo sapiens]
Human	chr12:56167344-56197945:+	AK091858	AK122956	Homo sapiens cDNA FLJ16674 fis, clone THYMU3008136, highly similar to Methionyl-tRNA synthetase (EC 6.1.1.10).
Human	chr12:56196279-56200573:-	CR616853	BC003637	Homo sapiens DNA-damage-inducible transcript 3, mRNA (cDNA clone MGC:4154 IMAGE:3530545), complete cds.
Mouse	chr10:127414175-127436266:-	9330175M06	BC058968	Mus musculus methionine-tRNA synthetase, mRNA (cDNA clone IMAGE:6816085), partial cds.
Mouse	chr10:127415228-127420771:+	BC013718	2010315M21	DNA-damage inducible transcript 3
Human	chr12:56202886-56210200:+	BC034295	AK056399	Homo sapiens cDNA FLJ31837 fis, clone NT2RP7000069, weakly similar to MUCIN 1 PRECURSOR.
Human	chr12:56209583-56227298:-	BF223768	AK096242	Homo sapiens cDNA FLJ38923 fis, clone NT2NE2011823, highly similar to DYNACTIN, 50 KDA ISOFORM.
Mouse	chr10:127406436-127413498:-	BC038259	K430314N15	Methyl-CpG binding domain protein 6 homolog [Homo sapiens]
Mouse	chr10:127390742-127407016:+	AI847110	G630083P05	dynactin 2
Human	chr12:56290342-56306201:+	AK057416	AK057416	Homo sapiens cDNA FLJ32854 fis, clone TESTI2003498, moderately similar to TRIPLE FUNCTIONAL DOMAIN PROTEIN.
Human	chr12:56305945-56313252:-	M83651	M83651	Human beta-1,4 N-acetylgalactosaminyltransferase mRNA, complete cds.
Mouse	chr10:127292023-127301280:-	D630010I07	D630010I07	hypothetical protein
Mouse	chr10:127285718-127293407:+	G630077K20	G630077K20	UDP-N-acetyl-alpha-D-galactosamine:(N-acetylneuraminyl)-galactosylglucosylceramide-beta-1, 4-N-acetylgalactosaminyltransferase
Human	chr12:56418063-56430237:+	BX647402	BX647402	Homo sapiens mRNA; cDNA DKFZp686N1153 (from clone DKFZp686N1153).
Human	chr12:56427026-56436085:-	BC003644	CR602927	full-length cDNA clone CS0DH005YN01 of T cells (Jurkat cell line) of Homo sapiens (human).
Mouse	chr10:127188197-127192151:-	2700085A14	2700085A14	SARCOMA AMPLIFIED SEQUENCE homolog [Homo sapiens]
Mouse	chr10:127185380-127189215:+	2610203O19	2610203O19	cyclin-dependent kinase 4
Human	chr12:68923041-69035041:+	BM752795	AF044215	Homo sapiens unknown mRNA, complete cds.
Human	chr12:68921967-68923407:-	BI596851	-	-
Mouse	chr10:116275787-116372464:-	BC065171	6330525K15	CCR4-NOT transcription complex, subunit 2
Mouse	chr10:116299679-116374142:+	5330438D12	5330438D12	hypothetical protein
Human	chr12:70119991-70266358:+	AK075399	AK075399	Homo sapiens cDNA PSEC0089 fis, clone PLACE1001148, highly similar to Homo sapiens orphan G protein-coupled receptor HG38 mRNA.
Human	chr12:70262340-70263899:-	AI962439	-	-
Mouse	chr10:115238062-115375802:-	4022446J01	4022446J01	G protein-coupled receptor 49
Mouse	chr10:115240286-115241092:+	4930586K15	4930586K15	G protein-coupled receptor 49
Human	chr12:70343062-70384107:+	BI964807	AL136607	Homo sapiens mRNA; cDNA DKFZp564I0422 (from clone DKFZp564I0422); complete cds.
Human	chr12:70289519-70347772:-	BC015679	BX647709	Homo sapiens mRNA; cDNA DKFZp686A0722 (from clone DKFZp686A0722).
Mouse	chr10:115155126-115173218:-	A630021N01	C130002K22	hypothetical protein
Mouse	chr10:115172384-115174619:+	K330328M14	-	-
Human	chr12:73217837-73221501:+	AK056642	AK056642	Homo sapiens cDNA FLJ32080 fis, clone OCBBF2000015.
Human	chr12:73218192-73218680:-	AW404679	-	-
Mouse	chr10:112700317-112707305:-	CB245162	9930036K04	unclassifiable
Mouse	chr10:112706780-112709432:+	E130311F08	E130311F08	hypothetical Arginine-rich region profile containing protein

Human	chr12:74160727-74181983:+	AF400440	X91911	H.sapiens mRNA for RTVP-1 protein.
Human	chr12:74174309-74176146:-	BX350135	-	-
Mouse	chr10:111770850-111788257:-	BC025083	F630012P11	GLI pathogenesis-related 1 (glioma)
Mouse	chr10:111758066-111773833:+	7330409K10	6430558M04	HIV-1 REV BINDING PROTEIN 2 homolog [Homo sapiens]
Human	chr12:74160727-74181983:+	X91911	X91911	H.sapiens mRNA for RTVP-1 protein.
Human	chr12:74176622-74191807:-	BC033887	BC033887	Homo sapiens HIV-1 rev binding protein 2, mRNA (cDNA clone MGC:33073 IMAGE:5268517), complete cds.
Mouse	chr10:111770850-111788257:-	BC025083	F630012P11	GLI pathogenesis-related 1 (glioma)
Mouse	chr10:111758066-111773833:+	7330409K10	6430558M04	HIV-1 REV BINDING PROTEIN 2 homolog [Homo sapiens]
Human	chr12:81254776-81375484:+	AK026442	BC029120	Homo sapiens, Similar to hypothetical protein FLJ22789, clone MGC:34762 IMAGE:5189049, mRNA, complete cds.
Human	chr12:81248551-81255034:-	AW732888	AF161477	Homo sapiens HSPC128 mRNA, complete cds.
Mouse	chr10:105438634-105516826:-	C820019H10	C820019H10	unclassifiable
Mouse	chr10:105516539-105523279:+	BM460917	6820447N13	Similar to BR22 TTF-1 associated protein homolog [Mus musculus]
Human	chr12:86930091-86946405:+	BX640968	AK095477	Homo sapiens cDNA FLJ38158 fis, clone DFNES2001091.
Human	chr12:86945253-87038461:-	AB002371	AB002371	Human mRNA for KIAA0373 gene, complete cds.
Mouse	chr10:100272762-100292005:-	3010023K05	4930571E09	hypothetical protein
Mouse	chr10:100188529-100280809:+	F830117J24	BC004690	Mus musculus cDNA sequence BC004690, mRNA (cDNA clone MGC:7859 IMAGE:3501291), complete cds. CDS=183..2090
Human	chr12:88421114-88436553:+	BI464251	-	-
Human	chr12:88315963-88422492:-	BX647258	AK074772	Homo sapiens cDNA FLJ90291 fis, clone NT2RP1001031, weakly similar to VEGETATIBLE INCOMPATIBILITY PROTEIN HET-E-1.
Mouse	chr10:98886739-98889028:-	5830415M17	5830415M17	hypothetical Glycine-rich region profile/Arginine-rich region profile containing protein
Mouse	chr10:98887984-98978716:+	BC050888	BC050888	Mus musculus RIKEN cDNA 4933430F16 gene, mRNA (cDNA clone IMAGE:6418526), partial cds.
Human	chr12:100593193-100640386:+	BC050429	BC050429	Homo sapiens choline phosphotransferase 1, mRNA (cDNA clone MGC:54156 IMAGE:6473945), complete cds.
Human	chr12:100624894-100635718:-	AK125930	AK125930	Homo sapiens cDNA FLJ43942 fis, clone TESTI4014276, moderately similar to Rattus norvegicus Synaptonemal complex protein 3 (Sycp3).
Mouse	chr10:88199154-88251055:-	D530004I04	1810073D20	CHOLINEPHOSPHOTRANSFERASE 1 BETA homolog [Homo sapiens]
Mouse	chr10:88206252-88219901:+	Y08485	Y08485	M.musculus mRNA for synaptonemal complex protein. CDS=89..853
Human	chr12:101016423-101093790:+	BG105175	AK000648	Homo sapiens cDNA FLJ20641 fis, clone KAT02782.
Human	chr12:101092704-101094090:-	M57703	BC018048	Homo sapiens, clone MGC:26374 IMAGE:4794545, mRNA, complete cds.
Mouse	chr10:87838414-87894211:-	9530018P16	9530018P16	ANTISENSE RNA OVERLAPPING MCH protein
Mouse	chr10:87838090-87839392:+	A230109K23	A230079H18	Pro-MCH precursor [Contains: Neuropeptide-glycine-glutamic acid (NGE) (Neuropeptide G-E); Neuropeptide-glutamic acid-isoleucine (NEI) (Neuropeptide E-I); Melanin-concentrating hormone (MCH)]
Human	chr12:102862049-102885978:+	BM459353	U51166	Human G/T mismatch-specific thymine DNA glycosylase mRNA, complete cds.
Human	chr12:102885228-102960428:-	BC022343	AK056056	Homo sapiens cDNA FLJ31494 fis, clone NT2NE2004716, weakly similar to Homo sapiens AD-017 protein mRNA, complete cds.
Mouse	chr10:82287242-82308052:+	E130317C12	E130317C12	thymine DNA glycosylase
Mouse	chr10:82307685-82347828:-	1110021D20	1110021D20	hypothetical Nucleotide-diphospho-sugar transferases structure containing protein
Human	chr12:105851967-105875134:+	BF980328	AK001731	Homo sapiens cDNA FLJ10869 fis, clone NT2RP4001677.
Human	chr12:105873536-105883405:-	BC025984	BC025984	Homo sapiens, transcription termination factor-like protein, clone MGC:22661 IMAGE:4106188, mRNA, complete cds.
Mouse	chr10:84770325-84788316:+	1700007D05	1700007D05	hypothetical protein
Mouse	chr10:84787131-84795725:-	C030035F06	C030035F06	similar to TRANSCRIPTION TERMINATION FACTOR-LIKE PROTEIN [Homo sapiens]
Human	chr12:108349283-108349952:+	BU753589	-	-
Human	chr12:108349180-108377875:-	BC040062	BC040062	Homo sapiens, Similar to MSTP028 protein, clone MGC:46001 IMAGE:5494016, mRNA, complete cds.
Mouse	chr5:111769095-111777637:+	4631401O15	4631401O15	MYOSIN-1H (FRAGMENT) homolog [Mus musculus]
Mouse	chr5:111775847-111792788:-	G270004A11	BC006935	Mus musculus potassium channel tetramerisation domain containing 10, mRNA (cDNA clone MGC:11654 IMAGE:3601917), complete cds. CDS=59..1006
Human	chr12:109368944-109390912:+	CR623722	CR623722	full-length cDNA clone CS0DK003YJ10 of HeLa cells Cot 25-normalized of Homo sapiens (human).
Human	chr12:109353011-109369793:-	BI828738	BC008416	Homo sapiens, clone MGC:14560 IMAGE:4048678, mRNA, complete cds.
Mouse	chr5:119877969-119886292:-	G530145D02	1500011H22	weakly similar to Protein predicted by clone 23733 [Homo sapiens]
Mouse	chr5:119885807-119896833:+	A630089G19	A630089G19	similar to PROTEIN X 0004 [Homo sapiens]
Human	chr12:109432751-109434081:+	AI807828	-	-
Human	chr12:109433770-109483784:-	CR749216	AF385435	Homo sapiens T-cell activation protein phosphatase 2C mRNA, complete cds.
Mouse	chr5:119837151-119868163:-	9130009G01	9130009G01	RAD9 homolog B (S. cerevisiae)
Mouse	chr5:119798260-119838209:+	A930103H12	D030027E24	T-cell activation protein phosphatase 2C homolog [Homo sapiens]
Human	chr12:109514605-109550802:+	BC044885	AK055891	Homo sapiens cDNA FLJ31329 fis, clone MAMGL1000032.
Human	chr12:109528366-109599782:-	AY359054	AY359054	Homo sapiens clone DNA59607 ATWD578 (UNQ578) mRNA, complete cds.
Mouse	chr5:119751590-119777878:-	6720407N02	A330095D17	Hypothetical cysteine-rich region containing protein homolog [Mus musculus]
Mouse	chr5:119720514-119755715:+	F630110H18	F630110H18	hypothetical Cation channel, non-ligand gated containing protein

Human	chr12:112053562-112059801:+	BM975869	-	-
Human	chr12:112057698-112086004:-	BC005848	AF478457	Homo sapiens ATP-dependent RNA helicase mRNA, complete cds.
Mouse	chr5:118118901-118120208:-	1190018L05	1190018L05	hypothetical protein
Mouse	chr5:118105357-118121179:+	6030486C03	BC043699	Mus musculus, Similar to RIKEN cDNA 2410015A15 gene, clone IMAGE:5344158, mRNA.
Human	chr12:116917226-116938914:+	BM505055	BC001866	Homo sapiens, replication factor C (activator 1) 5 (36.5kD), clone MGC:1155 IMAGE:3544137, mRNA, complete cds.
Human	chr12:116933212-116962950:-	AF163324	BC015887	Homo sapiens WD repeat and SOCS box containing protein 2, mRNA (cDNA clone MGC:10210 IMAGE:3910968), complete cds.
Mouse	chr5:114824334-114835288:-	G530123A15	G530123A15	replication factor C (activator 1) 5
Mouse	chr5:114803545-114825740:+	G430113B20	AF072881	Mus musculus SOCS box-containing WD protein SWiP-2 (Swip2) mRNA, complete cds. CDS=78..1292
Human	chr12:119338613-119364276:+	BC034962	BC034962	Homo sapiens, clone IMAGE:4822098, mRNA, partial cds.
Human	chr12:119362191-119370316:-	U30825	CR603096	full-length cDNA clone CS0DC007YP24 of Neuroblastoma Cot 25-normalized of Homo sapiens (human).
Mouse	chr5:112747172-112755535:-	B930015F03	9330178C18	hypothetical Glu-tRNAGln amidotransferase C subunit containing protein
Mouse	chr5:112741352-112747909:+	BC012217	BC012217	Mus musculus splicing factor, arginine/serine rich 9, mRNA (cDNA clone MGC:7233 IMAGE:3483731), complete cds. CDS=133..801
Human	chr12:119879269-119903619:+	CR595845	M57732	Human hepatic nuclear factor 1 (TCF1) mRNA, complete cds, clones HCL10, HCL12, HCL17, and HCL20.
Human	chr12:119901009-119917020:-	BM724029	CR618697	full-length cDNA clone CS0DN003YD11 of Adult brain of Homo sapiens (human).
Mouse	chr5:112359606-112382238:-	M57966	M57966	Mouse hepatocyte nuclear factor 1 (HNF-1) mRNA, complete cds. CDS=199..2085
Mouse	chr5:112353403-112361028:+	6430539H06	6430539H06	hypothetical Lysine-rich region profile containing protein
Human	chr12:120300564-120331146:+	AK055282	CR601814	full-length cDNA clone CS0DL007YE15 of B cells (Ramos cell line) Cot 25-normalized of Homo sapiens (human).
Human	chr12:120309122-120481609:-	AB031230	AJ459424	Homo sapiens mRNA for JEMMA protein (PCCX2 gene).
Mouse	chr5:120352773-120402023:+	4022417L22	I0C0028L11	ring finger protein 34
Mouse	chr5:120399646-120544041:-	AF176524	E430001G17	Homo sapiens F-box and leucine-rich repeat protein 11
Human	chr12:120613393-120683627:+	AK123181	BC035884	Homo sapiens, clone IMAGE:4991579, mRNA.
Human	chr12:120678140-120694602:-	AK000254	AK000254	Homo sapiens cDNA FLJ20247 fis, clone COLF6493.
Mouse	chr5:120605718-120647461:+	B930087K24	3830425B10	transmembrane protein induced by tumor necrosis factor alpha
Mouse	chr5:120640610-120661886:-	F830106F02	F830106F02	ras homolog gene family, member f
Human	chr12:120703507-120704029:+	BX116539	-	-
Human	chr12:120695893-120704532:-	BC038786	BC038786	Homo sapiens, clone IMAGE:5271722, mRNA.
Mouse	chr5:120668058-120670985:+	4932422M17	4932422M17	hypothetical protein
Mouse	chr5:120662917-120670531:-	C630044O15	-	-
Human	chr12:121212970-121218379:+	AK095746	AK095746	Homo sapiens cDNA FLJ38427 fis, clone FEBRA2013019, weakly similar to UDP-GlcNAc:betaGal beta-1,3-N-acetylglucosaminyltransferase 3.
Human	chr12:121217089-121275948:-	AK057778	AL833244	Homo sapiens mRNA; cDNA DKFZp761B1220 (from clone DKFZp761B1220).
Mouse	chr5:121055409-121061048:+	C230098J07	C230098J07	unclassifiable
Mouse	chr5:121056802-121072316:-	4932411H16	4932411H16	diablo homolog (Drosophila)
Human	chr12:122467071-122482541:+	AK097550	BC028485	Homo sapiens, clone IMAGE:3536581, mRNA.
Human	chr12:122477218-122543380:-	AK096697	AK096697	Homo sapiens cDNA FLJ39378 fis, clone PERIC2000214.
Mouse	chr5:122028677-122039129:+	K430356M01	BC058361	Mus musculus RIKEN cDNA 6330548G22 gene, mRNA (cDNA clone MGC:66719 IMAGE:5693273), complete cds. CDS=96..830
Mouse	chr5:122038623-122076980:-	AB041584	BC051945	Mus musculus RIKEN cDNA 2900002H16 gene, mRNA (cDNA clone MGC:62198 IMAGE:5716540), complete cds. CDS=409..1629
Human	chr12:122611526-122630641:+	AL040483	BC030020	Homo sapiens DEAD (Asp-Glu-Ala-Asp) box polypeptide 55, mRNA (cDNA clone MGC:33209 IMAGE:4821394), complete cds.
Human	chr12:122630450-122643189:-	CR612620	CR624872	full-length cDNA clone CS0DE011YP09 of Placenta of Homo sapiens (human).
Mouse	chr5:122098407-122116298:+	K230321B13	BC043052	Mus musculus DEAD (Asp-Glu-Ala-Asp) box polypeptide 55, mRNA (cDNA clone IMAGE:5708637), partial cds. CDS=1..1809
Mouse	chr5:122115189-122124647:-	5330440K01	5330440K01	eukaryotic translation initiation factor 2B, subunit 1 (alpha)
Human	chr13:20175014-20195241:+	AY078238	AY078238	Homo sapiens interleukin 27 precursor (IL27) mRNA, complete cds.
Human	chr13:20174266-20175857:-	CR596712	CR596712	full-length cDNA clone CS0DI028YF10 of Placenta Cot 25-normalized of Homo sapiens (human).
Mouse	chr14:49513278-49531672:+	2310031C12	2310031C12	interleukin 17D
Mouse	chr14:49512307-49513405:-	BE648689	-	-
Human	chr13:20930724-20932752:+	CR597960	CR597960	full-length cDNA clone CS0DI008YJ04 of Placenta Cot 25-normalized of Homo sapiens (human).
Human	chr13:20826048-20931509:-	BC034944	BC034944	Homo sapiens, clone IMAGE:4824131, mRNA.
Mouse	chr14:49881714-49882728:+	4930542A17	4930542A17	hypothetical protein
Mouse	chr14:49824595-49882221:-	BC019536	BC019536	Mus musculus RIKEN cDNA 5033406L14 gene, mRNA (cDNA clone MGC:28444 IMAGE:4039734), complete cds. CDS=100..1206
Human	chr13:26896681-26907960:+	U20272	D32257	Human GTF3A mRNA for Xenopus transcription factor IIIA homologue, complete cds.
Human	chr13:26901947-26904057:-	AK124468	AK124468	Homo sapiens cDNA FLJ42477 fis, clone BRACE2031527.
Mouse	chr5:144273680-144280645:+	BC064891	AF391799	Mus musculus transcription factor IIIA mRNA, partial cds. CDS=1..1203
Mouse	chr5:144276604-144288831:-	BC052439	BC052439	Mus musculus RIKEN cDNA 2810012L14 gene, mRNA (cDNA clone IMAGE:6406303), with apparent retained intron.

Human	chr13:26896681-26907960:+	CR600569	D32257	Human GTF3A mRNA for Xenopus transcription factor IIIA homologue, complete cds.
Human	chr13:26907776-26922728:-	CR594840	BC046166	Homo sapiens, mitochondrial translational initiation factor 3, clone MGC:57728 IMAGE:3460180, mRNA, complete cds.
Mouse	chr5:144273680-144280645:+	BC064891	AF391799	Mus musculus transcription factor IIIA mRNA, partial cds. CDS=1..1203
Mouse	chr5:144276604-144288831:-	BC052439	BC052439	Mus musculus RIKEN cDNA 2810012L14 gene, mRNA (cDNA clone IMAGE:6406303), with apparent retained intron.
Human	chr13:36470953-36481751:+	CR595030	AK096810	Homo sapiens cDNA FLJ39491 fis, clone PROST2015924, weakly similar to Homo sapiens Opa-interacting protein OIP2 mRNA.
Human	chr13:36481449-36531843:-	AF370384	BC030686	Homo sapiens transcription factor (p38 interacting protein), mRNA (cDNA clone MGC:26084 IMAGE:4826077), complete cds.
Mouse	chr3:54821435-54828151:-	2310032N20	BC059089	Mus musculus RIKEN cDNA 2310032N20 gene, mRNA (cDNA clone MGC:70125 IMAGE:6509820), complete cds. CDS=370..1200
Mouse	chr3:54785575-54821520:+	BC052702	F830011C07	hypothetical Glutamine-rich region profile containing protein
Human	chr13:40533393-40556148:+	BI548336	AK001557	Homo sapiens cDNA FLJ10695 fis, clone NT2RP3000403, highly similar to Homo sapiens formin binding protein 21 mRNA.
Human	chr13:40402882-40533576:-	AL691606	BX640798	Homo sapiens mRNA; cDNA DKFZp686H0575 (from clone DKFZp686H0575); complete cds.
Mouse	chr14:71233171-71254758:-	I420022O12	6720428N02	WW domain binding protein 4
Mouse	chr14:71254417-71355732:+	BC057134	BC057134	Mus musculus E74-like factor 1, mRNA (cDNA clone MGC:73921 IMAGE:6853870), complete cds. CDS=673..2511
Human	chr13:44937042-45008768:+	CR609778	AK026305	Homo sapiens cDNA: FLJ22652 fis, clone HSI07445, highly similar to AF131829 Homo sapiens clone 25226 mRNA sequence.
Human	chr13:44934942-44937631:-	AK057884	AK057884	Homo sapiens cDNA FLJ25155 fis, clone CBR07976.
Mouse	chr14:67462507-67514711:-	BC038030	BC038030	Mus musculus component of oligomeric golgi complex 3, mRNA (cDNA clone MGC:47119 IMAGE:4020725), complete cds. CDS=91..2553
Mouse	chr14:67514294-67516057:+	C030017I19	C030017I19	weakly similar to CDNA FLJ25155 FIS, CLONE CBR07976 [Homo sapiens]
Human	chr13:50813169-50836872:+	BU741166	AX772926	Sequence 1 from Patent WO03046006.
Human	chr13:50826001-50925344:-	BF673043	AL833524	Homo sapiens mRNA; cDNA DKFZp686I1337 (from clone DKFZp686I1337).
Mouse	chr14:54654494-54683149:+	E130113E03	E130113E03	hypothetical Serpins containing protein
Mouse	chr14:54667147-54752025:-	E430013K21	BC059263	Mus musculus cDNA clone MGC:67608 IMAGE:6409580, complete cds. CDS=455..3106
Human	chr13:51056586-51239110:+	BC014004	BC014004	Homo sapiens WD repeat and FYVE domain containing 2, mRNA (cDNA clone MGC:20275 IMAGE:3842589), complete cds.
Human	chr13:51054756-51056965:-	AK056283	AK056283	Homo sapiens cDNA FLJ31721 fis, clone NT2RI2006667.
Mouse	chr14:54827173-54950091:+	D930017N21	B130024L21	WD40-AND FYVE-DOMAIN CONTAINING PROTEIN 2 homolog [Homo sapiens]
Mouse	chr14:54826396-54827704:-	F930044L06	F930044L06	unclassifiable
Human	chr13:94162971-94166501:+	AK055459	AK055459	Homo sapiens cDNA FLJ30897 fis, clone FEBRA2005476.
Human	chr13:94162975-94163726:-	BM783259	-	-
Mouse	chr14:110557857-110560384:+	A330041I15	A330041I15	unclassifiable
Mouse	chr14:110554752-110558655:-	BQ876146	AY142959	Mus musculus HMG-box protein SOX21 (Sox21) mRNA, complete cds. CDS=268..1098
Human	chr13:94751209-94761265:+	BU158437	-	-
Human	chr13:94470084-94751684:-	AF071202	AY081219	Homo sapiens multidrug resistance-associated protein (ABCC4) mRNA, complete cds.
Mouse	chr14:111027361-111029326:+	B230330H24	B230330H24	unclassifiable
Mouse	chr14:110803904-111027927:-	B130008G09	B130008G09	ATP-binding cassette, sub-family C (CFTR/MRP), member 4
Human	chr13:100876188-101173457:+	CA440406	AF072752	Homo sapiens ten integrin EGF-like repeat domains protein precursor (ITGBL1) mRNA, complete cds.
Human	chr13:101167202-101168770:-	BM728000	-	-
Mouse	chr14:115985881-116271786:+	E230003L14	B930011D01	similar to TEN INTEGRIN EGF-LIKE REPEAT DOMAINS PROTEIN PRECURSOR [Homo sapiens]
Mouse	chr14:116270447-116272064:-	A630033B20	A630033B20	unclassifiable
Human	chr14:20608269-20628249:+	AI928047	AK074057	Homo sapiens mRNA for FLJ00128 protein.
Human	chr14:20628045-20642634:-	BC000694	AB015427	Homo sapiens mRNA for zinc finger protein 219, complete cds.
Mouse	chr14:44725234-45121075:+	E970014O03	E970014O03	similar to Human full-length cDNA 5-PRIME end of clone CS0DI001YE04 of placenta of Homo sapiens (Human) (Fragment) [Homo sapiens]
Mouse	chr14:44746318-44760617:-	E130018L11	AB063578	Mus musculus ZNF219 mRNA for zinc finger protein 219, complete cds. CDS=295..2475
Human	chr14:20636936-20641723:+	AA284845	-	-
Human	chr14:20628045-20642634:-	AK056849	AB015427	Homo sapiens mRNA for zinc finger protein 219, complete cds.
Mouse	chr14:44755542-44759671:+	G630016D24	G630016D24	hypothetical protein
Mouse	chr14:44746318-44760617:-	F630021E17	AB063578	Mus musculus ZNF219 mRNA for zinc finger protein 219, complete cds. CDS=295..2475
Human	chr14:20818082-20889300:+	AF265666	AJ417067	Homo sapiens mRNA for RPGR-interacting protein 1 (RPGRIP1 gene).
Human	chr14:20888430-20889338:-	BQ015193	-	-
Mouse	chr14:44850571-44903426:+	A230095N11	4932410O22	retinitis pigmentosa GTPase regulator interacting protein 1
Mouse	chr14:44898424-44936954:-	AF323667	AF323667	Mus musculus chromatin-specific transcription elongation factor, 140 kDa subunit (Factp140) mRNA, complete cds. CDS=52..3195
Human	chr14:21014754-21041606:+	CN361773	AB018280	Homo sapiens mRNA for KIAA0737 protein, complete cds.
Human	chr14:20996700-21014972:-	AF468652	AF468652	Homo sapiens RAB2B mRNA, complete cds.
Mouse	chr14:45018833-45036156:+	K430304K16	I920088P11	Epidermal Langerhans cell protein LCP1
Mouse	chr14:45001400-45019238:-	4930528G15	BC046334	Mus musculus RIKEN cDNA 1500012D09 gene, mRNA (cDNA clone MGC:54924 IMAGE:6492838), complete cds. CDS=97..747

Human	chr14:21014754-21041606:+	CR603862	AB018280	Homo sapiens mRNA for KIAA0737 protein, complete cds.
Human	chr14:21036115-21049353:-	AK098655	BC014427	Homo sapiens, clone IMAGE:4564560, mRNA.
Mouse	chr14:45018833-45036156:+	9430052H12	I920088P11	Epidermal Langerhans cell protein LCP1
Mouse	chr14:45034428-45044889:-	AF135789	AF135789	Mus musculus m6a methyltransferase mRNA, complete cds. CDS=97..1443
Human	chr14:22633757-22639511:+	CR622831	BC009645	Homo sapiens, hypothetical protein FLJ20671, clone MGC:4950 IMAGE:3458006, mRNA, complete cds.
Human	chr14:22597612-22634663:-	AF124726	AF124726	Homo sapiens acinusL mRNA, complete cds.
Mouse	chr14:46659128-46663693:+	AB041657	AB041657	Mus musculus brain cDNA, clone MNCb-2990. CDS=907..1335
Mouse	chr14:46614983-46659911:-	AF168782	AF168782	Mus musculus acinusL protein mRNA, complete cds. CDS=153..4169
Human	chr14:23771488-23778314:+	BX161436	BX161436	human full-length cDNA clone CS0DI014YE13 of Placenta of Homo sapiens (human).
Human	chr14:23748627-23771822:-	BG107047	D23662	Homo sapiens mRNA for ubiquitin-like protein, complete cds.
Mouse	chr14:47646068-47653327:+	5730544D12	5830487D22	GUANOSINE MONOPHOSPHATE REDUCTASE ISOLOG (GMP REDUCTASE) (GMPR2 FOR GUANOSINE MONOPHOSPHATE REDUCTASE ISOLOG) homolog [Homo sapiens]
Mouse	chr14:47636387-47646251:-	I730034J01	I920047M10	neural precursor cell expressed, developmentally down-regulated gene 8
Human	chr14:23838908-23857082:+	AB095941	AB095941	Homo sapiens mRNA for KIAA2021 protein.
Human	chr14:23844142-23850495:-	BC000982	AK122828	Homo sapiens cDNA FLJ16433 fis, clone BRACE3013936, highly similar to Cell death activator CIDE-B.
Mouse	chr14:47719821-47733139:+	I920041N19	I920041N19	hypothetical Pumilio/Puf RNA-binding/ARM repeat fold containing protein
Mouse	chr14:47728173-47732560:-	AF041377	BC046340	Mus musculus cell death-inducing DNA fragmentation factor, alpha subunit-like effector B, mRNA (cDNA clone MGC:54676 IMAGE:6478230), complete cds. CDS=127..786
Human	chr14:23968332-23980388:+	AB002321	AB002321	Human mRNA for KIAA0323 gene, partial cds.
Human	chr14:23978808-23981950:-	AF226050	BC000989	Homo sapiens, HCDI protein, clone MGC:5313 IMAGE:2900140, mRNA, complete cds.
Mouse	chr14:47859070-47872908:+	BC034330	AK122247	Mus musculus mRNA for mKIAA0323 protein. CDS=27..2219
Mouse	chr14:47871411-47874386:-	BC031783	C530043L06	similar to HCDI PROTEIN [Homo sapiens]
Human	chr14:30959032-30991839:+	N34214	BC041327	Homo sapiens, clone IMAGE:5276962, mRNA.
Human	chr14:30939223-30959506:-	BC062720	BC062720	Homo sapiens cDNA clone MGC:72028 IMAGE:6733067, complete cds.
Mouse	chr12:47190105-47191607:+	C230081H03	C230081H03	hypothetical protein
Mouse	chr12:47174762-47190532:-	BI658364	-	-
Human	chr14:34624992-34627015:+	BC037797	BC037797	Homo sapiens, Similar to hypothetical protein FLJ20378, clone IMAGE:4794562, mRNA.
Human	chr14:34624424-34661499:-	CR601345	CR622598	full-length cDNA clone CS0DI032YH10 of Placenta Cot 25-normalized of Homo sapiens (human).
Mouse	chr12:50385915-50404963:+	D830016P21	BC048158	Mus musculus RIKEN cDNA 1700047I17 gene, mRNA (cDNA clone MGC:61221 IMAGE:5698044), complete cds. CDS=115..738
Mouse	chr12:50217166-50416439:-	CB235447	2700097O09	hypothetical S-adenosyl-L-methionine-dependent methyltransferases structure containing protein
Human	chr14:36058272-36061969:+	BX161496	BX161496	human full-length cDNA clone CS0DJ013YG01 of T cells (Jurkat cell line) of Homo sapiens (human).
Human	chr14:36012163-36059167:-	U43203	AY102071	Homo sapiens surfactant associated protein H mRNA, complete sequence.
Mouse	chr12:51728069-51801141:+	CK794438	-	-
Mouse	chr12:51724684-51729635:-	CB723747	BC057607	Mus musculus thyroid transcription factor 1, mRNA (cDNA clone MGC:67328 IMAGE:6416507), complete cds. CDS=614..1732
Human	chr14:36710982-36712767:+	BC012470	AK057476	Homo sapiens cDNA FLJ32914 fis, clone TESTI2006409.
Human	chr14:36218829-36711616:-	AJ278148	AJ278148	Homo sapiens partial mRNA for oxodicarboxylate carrier (ODC gene).
Mouse	chr12:52395512-52396184:+	BU962695	-	-
Mouse	chr12:51906557-52396109:-	A630030I10	A630030I10	MOTOCHONDRIAL OXODICARBOXYLATE CARRIER homolog [Rattus norvegicus]
Human	chr14:44623055-44655329:+	BG818213	BC028683	Homo sapiens PRP39 pre-mRNA processing factor 39 homolog (yeast), mRNA (cDNA clone IMAGE:4821362), partial cds.
Human	chr14:44654552-44674272:-	CR590899	BC016288	Homo sapiens, FK506-binding protein 3 (25kD), clone MGC:9482 IMAGE:3919642, mRNA, complete cds.
Mouse	chr12:60288959-60316250:+	BC029153	F630006B21	PRP39 pre-mRNA processing factor 39 homolog (yeast)
Mouse	chr12:60315288-60329180:-	I1C0025P14	I1C0025P14	FK506 binding protein 3
Human	chr14:49933481-49934813:+	BQ082534	-	-
Human	chr14:49865872-49952929:-	AF390028	AF390028	Homo sapiens serine/threonine protein kinase kkialre-like 1 mRNA, complete cds.
Mouse	chr12:65053782-65105343:+	4930512B01	4930472D12	unclassifiable
Mouse	chr12:65008739-65054761:-	4933411O17	4933411O17	cyclin-dependent kinase-like 1 (CDC2-related kinase)
Human	chr14:50068978-50169532:+	BF979636	AF444143	Homo sapiens brain-specific GTP-binding protein mRNA, complete cds.
Human	chr14:49954953-50097594:-	U77129	BC036013	Homo sapiens, mitogen-activated protein kinase kinase kinase 5, clone MGC:32909 IMAGE:5272190, mRNA, complete cds.
Mouse	chr12:65156006-65233898:+	M5C1012D11	M5C1012D11	spastic paraplegia 3A homolog (human)
Mouse	chr12:65067170-65156592:-	BC048173	BC048173	Mus musculus mitogen-activated protein kinase kinase kinase 5, mRNA (cDNA clone IMAGE:6825201), containing frame-shift errors.
Human	chr14:51525943-51667191:+	AU143395	AF151857	Homo sapiens CGI-99 protein mRNA, complete cds.
Human	chr14:51541270-51606295:-	BX648241	BX648241	Homo sapiens mRNA; cDNA DKFZp686D12108 (from clone DKFZp686D12108).
Mouse	chr14:16095628-16108132:-	9430026H22	9430026H22	CLE7 homolog [Gallus gallus]
Mouse	chr14:16035524-16096066:+	AK128978	BC054746	Mus musculus nidogen 2, mRNA (cDNA clone MGC:65650 IMAGE:6405073), complete cds. CDS=64..4275

Human	chr14:54903771-54906839:+	AL133091	AL133091	Homo sapiens mRNA; cDNA DKFZp434F0728 (from clone DKFZp434F0728).
Human	chr14:54902862-54948329:-	AK131251	AK131251	Homo sapiens cDNA FLJ16178 fis, clone BRHIP3000017.
Mouse	chr14:40211658-40213895:+	AI591984	-	-
Mouse	chr14:40210571-40238121:-	4932411G10	4932411G10	hypothetical protein
Human	chr14:57932397-57945172:+	AY358266	AY358266	Homo sapiens clone DNA184700 TIMM9 (UNQ9438) mRNA, complete cds.
Human	chr14:57943929-57963998:-	AF150100	BC020213	Homo sapiens, translocase of inner mitochondrial membrane 9 homolog (yeast), clone MGC:31874 IMAGE:4640518, mRNA, complete cds.
Mouse	chr12:66378429-66390223:+	4930553D19	BC048520	Mus musculus, clone IMAGE:6741198, mRNA.
Mouse	chr12:66390173-66403688:-	1500011K02	1500011K02	translocase of inner mitochondrial membrane 10 homolog (yeast)
Human	chr14:59020930-59041834:+	BX161501	CR605227	full-length cDNA clone CS0DH002YI05 of T cells (Jurkat cell line) of Homo sapiens (human).
Human	chr14:59041016-59061827:-	AI344951	-	-
Mouse	chr12:67356768-67372706:+	2900009D01	1200003C05	SIMILAR TO HYPOTHETICAL PROTEIN homolog [Mus musculus]
Mouse	chr12:67372471-67456462:-	4921526G01	-	-
Human	chr14:66777579-66872289:+	AL832326	AL832326	Homo sapiens mRNA; cDNA DKFZp451E015 (from clone DKFZp451E015); complete cds.
Human	chr14:66830835-66896735:-	AK125287	CR619369	full-length cDNA clone CS0DG004YJ05 of B cells (Ramos cell line) of Homo sapiens (human).
Mouse	chr12:74046346-74138153:+	4931438I24	4931438I24	membrane protein, palmitoylated 5 (MAGUK p55 subfamily member 5)
Mouse	chr12:74136210-74137026:-	AW495450	-	-
Human	chr14:67069766-67126081:+	AK124787	AB033026	Homo sapiens mRNA for KIAA1200 protein, partial cds.
Human	chr14:67118425-67136770:-	CR605373	L19783	Human GPI-H mRNA, complete cds.
Mouse	chr12:74326404-74378840:+	5730574D04	AK122464	Mus musculus mRNA for mKIAA1200 protein. CDS=3..4346
Mouse	chr12:74377852-74401124:-	I730035J11	9230116L04	PHOSPHATIDYLINOSITOL GLYCAN, CLASS H homolog [Homo sapiens]
Human	chr14:67156332-67188187:+	BC029050	BC029050	Homo sapiens, arginase, type II, clone MGC:35329 IMAGE:5179867, mRNA, complete cds.
Human	chr14:67183374-67232243:-	BC042390	CR604112	full-length cDNA clone CS0DK003YB20 of HeLa cells Cot 25-normalized of Homo sapiens (human).
Mouse	chr12:74425883-74451502:+	U90886	BC023349	Mus musculus arginase type II, mRNA (cDNA clone MGC:31006 IMAGE:5254031), complete cds. CDS=47..1111
Mouse	chr12:74446138-74447134:-	5830408N04	5830408N04	unclassifiable
Human	chr14:67156332-67188187:+	BC029050	BC029050	Homo sapiens, arginase, type II, clone MGC:35329 IMAGE:5179867, mRNA, complete cds.
Human	chr14:67183374-67232243:-	BC042390	CR604112	full-length cDNA clone CS0DK003YB20 of HeLa cells Cot 25-normalized of Homo sapiens (human).
Mouse	chr12:74425883-74451502:+	BC023349	BC023349	Mus musculus arginase type II, mRNA (cDNA clone MGC:31006 IMAGE:5254031), complete cds. CDS=47..1111
Mouse	chr12:74449166-74467869:-	0610007H13	0610007H13	vesicle transport through interaction with t-SNAREs 1B homolog
Human	chr14:67210757-67212682:+	CB995072	-	-
Human	chr14:67183374-67232243:-	AF060902	CR604112	full-length cDNA clone CS0DK003YB20 of HeLa cells Cot 25-normalized of Homo sapiens (human).
Mouse	chr12:74467316-74487266:+	K330301O19	4933431G12	cell line MC/9.IL4 derived transcript 1
Mouse	chr12:74449166-74467869:-	0610007H13	0610007H13	vesicle transport through interaction with t-SNAREs 1B homolog
Human	chr14:68934849-69004846:+	BP299886	BC047682	Homo sapiens, hypothetical protein FLJ11274, clone MGC:51849 IMAGE:6066186, mRNA, complete cds.
Human	chr14:68916336-68935097:-	CF147345	U66871	Human enhancer of rudimentary homolog mRNA, complete cds.
Mouse	chr12:75944170-75984293:+	BC058649	BC058649	Mus musculus RIKEN cDNA 4833420E20 gene, mRNA (cDNA clone IMAGE:6406316), partial cds.
Mouse	chr12:75934308-75944628:-	CN723570	A830025P17	ERH PROTEIN
Human	chr14:72594974-72659765:+	BC062440	BX647116	Homo sapiens mRNA; cDNA DKFZp686M06144 (from clone DKFZp686M06144).
Human	chr14:72594386-72595876:-	CR739627	-	-
Mouse	chr12:78945732-78997519:+	D130074N24	D130074N24	Similar to S164 protein homolog [Mus musculus]
Mouse	chr12:78860332-78946914:-	BI733386	D330037O08	zinc finger, FYVE domain containing 1
Human	chr14:73486387-73499916:+	BX248000	BX248000	human full-length cDNA clone CS0DM001YP16 of Fetal liver of Homo sapiens (human).
Human	chr14:73468732-73486700:-	CD300479	-	-
Mouse	chr12:79682112-79694251:+	CF105039	2210404I09	coenzyme Q6 homolog (yeast)
Mouse	chr12:79665764-79682283:-	9330169D17	9330169D17	similar to CDNA FLJ31697 FIS, CLONE NT2RI2005851, WEAKLY SIMILAR TO PLECTIN [Homo sapiens]
Human	chr14:73555816-73619319:+	AL834487	AK097531	Homo sapiens cDNA FLJ40212 fis, clone TESTI2021116.
Human	chr14:73593306-73620949:-	CR613362	BC032371	Homo sapiens, aldehyde dehydrogenase 6 family, member A1, clone MGC:40271 IMAGE:5163797, mRNA, complete cds.
Mouse	chr12:79729550-79763342:+	6430510N09	4933402K21	hypothetical Lysine-rich region profile containing protein
Mouse	chr12:79750644-79771479:-	BC033440	I920188E21	aldehyde dehydrogenase family 6, subfamily A1
Human	chr14:75197088-75620143:+	BX510229	AY237126	Homo sapiens SRC1 and TIF2 associated binding protein (STAMP) mRNA, complete cds.
Human	chr14:75185884-75197296:-	BX248022	AF134159	Homo sapiens potential membrane protein C14orf1 mRNA, complete cds.
Mouse	chr12:81149153-81386015:+	4732494O09	AB093278	Mus musculus mRNA for mKIAA0998 protein. CDS=154..4155
Mouse	chr12:81139852-81149387:-	BU148398	1190004E09	Protein C14orf1 (HSPC288) (Protein AD-011) (x0006) homolog [Homo sapiens]

Human	chr14:79747514-79991810:+	BC039670	BC039670	Homo sapiens, clone IMAGE:5167652, mRNA.
Human	chr14:79733621-79747723:-	AF093774	AF093774	Homo sapiens type 2 iodothyronine deiodinase mRNA, complete cds and 3'UTR.
Mouse	chr12:85330227-85383436:+	4931407H24	4931407H24	unclassifiable
Mouse	chr12:85316579-85331059:-	AF096875	AF096875	Mus musculus type 2 deiodinase mRNA, complete cds. CDS=155..955
Human	chr14:88099067-88150425:+	AL359625	AY578061	Homo sapiens putative NY-REN-37 antigen isoform 2 mRNA, complete cds, alternatively spliced.
Human	chr14:88148529-88251227:-	BX648569	AK128086	Homo sapiens cDNA FLJ46207 fis, clone TESTI4010902, weakly similar to Echinoderm microtubule-associated protein.
Mouse	chr12:93362029-93440218:+	BC024856	4021401G05	Nuclear protein UKp68 homolog [Rattus norvegicus]
Mouse	chr12:93401867-93516666:-	C230032N07	C130068M19	hypothetical G-protein beta WD-40 repeats containing protein
Human	chr14:92830457-92832286:+	BX099352	-	-
Human	chr14:92773649-92869191:-	AK001510	BX248762	human full-length cDNA 5-PRIME end of clone XCL0BB001ZA04 of Neuroblastoma of Homo sapiens (human).
Mouse	chr12:97517832-97554615:+	K630139B05	-	-
Mouse	chr12:97460759-97558195:-	BC057303	BC041669	Mus musculus BTB (POZ) domain containing 7, mRNA (cDNA clone MGC:49574 IMAGE:3482657), complete cds. CDS=451..3843
Human	chr14:93562433-93585032:+	CR457345	AK025569	Homo sapiens cDNA: FLJ21916 fis, clone HEP03994.
Human	chr14:93577546-93580310:-	AW732863	-	-
Mouse	chr12:98056611-98085417:+	BC052409	K230314A01	Hypothetical protein FLJ21916 (Ubiquitin-specific protease otubain 2) homolog [Homo sapiens]
Mouse	chr12:98081948-98085872:-	D930049C16	D930049C16	unclassifiable
Human	chr14:93562433-93585032:+	CR457345	AK025569	Homo sapiens cDNA: FLJ21916 fis, clone HEP03994.
Human	chr14:93577546-93580310:-	AW732863	-	-
Mouse	chr12:98056611-98085417:+	K230314A01	K230314A01	Hypothetical protein FLJ21916 (Ubiquitin-specific protease otubain 2) homolog [Homo sapiens]
Mouse	chr12:98078964-98081913:-	C230023N19	C230023N19	unclassifiable
Human	chr14:102458746-102469867:+	AX747751	AF328788	Homo sapiens amnionless mRNA, complete cds.
Human	chr14:102468469-102593626:-	AF128625	AF128625	Homo sapiens CDC42-binding protein kinase beta (CDC42BPB) mRNA, complete cds.
Mouse	chr12:106029329-106038558:+	A230087F16	A230087F16	inferred: KIAA1124 protein {Homo sapiens}
Mouse	chr12:106037542-106122288:-	BC033425	AY277589	Mus musculus CDC42-binding protein kinase beta (Cdc42bpb) mRNA, complete cds. CDS=89..5230
Human	chr14:103165270-103247461:+	Z43533	L04733	Homo sapiens kinesin light chain mRNA, complete cds.
Human	chr14:103230933-103233395:-	BM825411	-	-
Mouse	chr12:106462621-106560500:+	BC031426	6030405E13	kinesin light chain 1
Mouse	chr12:106555711-106566599:-	4432412E01	4432412E01	similar to X-RAY REPAIR CROSS-COMPLEMENTING PROTEIN 3 (X-RAY REPAIR COMPLEMENTING DEFECTIVE REPAIR IN CHINESE HAMSTER CELLS 3) [Homo sapiens]
Human	chr15:28902174-29022605:+	AB023235	AB023235	Homo sapiens mRNA for KIAA1018 protein, complete cds.
Human	chr15:29008555-29071099:-	AL833089	BC063296	Homo sapiens cDNA clone MGC:71646 IMAGE:30345912, complete cds.
Mouse	chr7:51329658-51338440:-	BM949398	-	-
Mouse	chr7:51279778-51332915:+	C230007M15	BC055074	Mus musculus expressed sequence BB128963, mRNA (cDNA clone MGC:60820 IMAGE:30052208), complete cds. CDS=23..2092
Human	chr15:32304490-32309649:+	CR619572	AK075227	Homo sapiens cDNA FLJ90746 fis, clone PLACE1011516.
Human	chr15:32309487-32417552:-	AK056019	BC051709	Homo sapiens mRNA similar to solute carrier family 12 (potassium/chloride transporters), member 6 (cDNA clone IMAGE:5298663).
Mouse	chr2:112253501-112259005:-	6330598A03	6330598A03	hypothetical protein
Mouse	chr2:112156507-112254293:+	BC051061	AF211854	Mus musculus K-Cl cotransporter 3a mRNA, complete cds, alternatively spliced. CDS=4..3456
Human	chr15:38013617-38115089:+	AL137627	AB037759	Homo sapiens mRNA for KIAA1338 protein, partial cds.
Human	chr15:38112489-38115101:-	BM802003	-	-
Mouse	chr2:118170193-118256810:+	AJ243533	AJ243533	Mus musculus mRNA for GCN2 eIF2alpha kinase. CDS=93..5039
Mouse	chr2:118255328-118261287:-	F830102O09	F830102O09	signal recognition particle 14
Human	chr15:38886573-38894059:+	AK122779	AK122779	Homo sapiens cDNA FLJ16329 fis, clone SYNOV2009172.
Human	chr15:38847361-38886963:-	BC000048	BC000048	Homo sapiens hypothetical protein FLJ10634, mRNA (cDNA clone MGC:944 IMAGE:3507470), complete cds.
Mouse	chr2:118990193-118998626:+	D130022F08	1500041L05	hypothetical FYVE Zn-finger, rabphilin/VPS27/FAB1 type/Cytochrome c family heme-binding site containing protein
Mouse	chr2:118954073-118990371:-	BC013487	BC013487	Mus musculus RIKEN cDNA 1700025B16 gene, mRNA (cDNA clone MGC:19126 IMAGE:4211118), complete cds. CDS=10..921
Human	chr15:40352709-40491810:+	CF594101	AK074037	Homo sapiens mRNA for FLJ00088 protein.
Human	chr15:40290018-40353044:-	BC005335	CR596160	full-length cDNA clone CS0DI082YM13 of Placenta Cot 25-normalized of Homo sapiens (human).
Mouse	chr2:120185472-120243276:+	9630047K19	4732440O11	weakly similar to GLUCOSIDASE II [Sus scrofa]
Mouse	chr2:120136666-120185692:-	E430020E17	E430020E17	hypothetical protein
Human	chr15:41409864-41433393:+	BI761521	BC075857	Homo sapiens cDNA clone MGC:90359 IMAGE:5199077, complete cds.
Human	chr15:41407263-41410100:-	BC015949	BC015949	Homo sapiens leucine carboxyl methyltransferase 2, mRNA (cDNA clone MGC:9534 IMAGE:3922888), complete cds.
Mouse	chr2:120922006-120938256:+	4930578F03	C530042F19	hypothetical Adenosine and AMP deaminase containing protein
Mouse	chr2:120918833-120922260:-	L930067E14	E330022A13	similar to P21WAF1/CIP1 PROMOTER-INTERACTING PROTEIN (FRAGMENT) [Homo sapiens]

Human	chr15:41450560-41485517:+	AK027703	AK027703	Homo sapiens cDNA FLJ14797 fis, clone NT2RP4001256, highly similar to Homo sapiens mRNA for gamma tubulin ring complex protein.
Human	chr15:41482547-41485782:-	AK125173	AK125173	Homo sapiens cDNA FLJ43183 fis, clone FCBBF3018826.
Mouse	chr2:120952230-120982028:+	B230343J24	C730031A08	GAMMA TUBULIN RING COMPLEX PROTEIN homolog [Homo sapiens]
Mouse	chr2:120976411-121052983:-	B230115F22	AJ414734	Mus musculus mRNA for 53BP1 protein. CDS=9..5882
Human	chr15:41485540-41487747:+	BM550311	-	-
Human	chr15:41482547-41485782:-	AK125173	AK125173	Homo sapiens cDNA FLJ43183 fis, clone FCBBF3018826.
Mouse	chr2:120952230-120982028:+	B230343J24	C730031A08	GAMMA TUBULIN RING COMPLEX PROTEIN homolog [Homo sapiens]
Mouse	chr2:120976411-121052983:-	B230115F22	AJ414734	Mus musculus mRNA for 53BP1 protein. CDS=9..5882
Human	chr15:41485540-41487747:+	AA043058	-	-
Human	chr15:41486699-41590104:-	BX537418	AF078776	Homo sapiens p53 tumor suppressor-binding protein 1 mRNA, complete cds.
Mouse	chr2:120952230-120982028:+	B230343J24	C730031A08	GAMMA TUBULIN RING COMPLEX PROTEIN homolog [Homo sapiens]
Mouse	chr2:120976411-121052983:-	B230115F22	AJ414734	Mus musculus mRNA for 53BP1 protein. CDS=9..5882
Human	chr15:41487790-41489261:+	AA009975	-	-
Human	chr15:41486699-41590104:-	BX537418	AF078776	Homo sapiens p53 tumor suppressor-binding protein 1 mRNA, complete cds.
Mouse	chr2:120952230-120982028:+	B230343J24	C730031A08	GAMMA TUBULIN RING COMPLEX PROTEIN homolog [Homo sapiens]
Mouse	chr2:120976411-121052983:-	B230115F22	AJ414734	Mus musculus mRNA for 53BP1 protein. CDS=9..5882
Human	chr15:41825827-41852769:+	BU620128	CR614986	full-length cDNA clone CS0DI025YG10 of Placenta Cot 25-normalized of Homo sapiens (human).
Human	chr15:41852086-41880033:-	AK057528	AK093233	Homo sapiens cDNA FLJ35914 fis, clone TESTI2010354, highly similar to Homo sapiens RNA polymerase II elongation factor ELL3 mRNA.
Mouse	chr2:121220519-121221175:+	G730031F18	G730031F18	unclassifiable
Mouse	chr2:121220586-121225854:-	B020010F16	B020010F16	elongation factor RNA polymerase II-like 3
Human	chr15:41855987-41881781:+	AK095876	BC009869	Homo sapiens cDNA clone IMAGE:3940599, partial cds.
Human	chr15:41852086-41880033:-	AK093233	AK093233	Homo sapiens cDNA FLJ35914 fis, clone TESTI2010354, highly similar to Homo sapiens RNA polymerase II elongation factor ELL3 mRNA.
Mouse	chr2:121237279-121240083:+	BC060244	BC060244	Mus musculus cDNA clone IMAGE:6829806, partial cds.
Mouse	chr2:121232753-121238599:-	A930015D22	A930015D22	hypothetical TMS membrane protein/tumour differentially expressed protein (TDE) containing protein
Human	chr15:41855987-41881781:+	AK095876	BC009869	Homo sapiens cDNA clone IMAGE:3940599, partial cds.
Human	chr15:41852086-41880033:-	AK093233	AK093233	Homo sapiens cDNA FLJ35914 fis, clone TESTI2010354, highly similar to Homo sapiens RNA polymerase II elongation factor ELL3 mRNA.
Mouse	chr2:121230774-121236273:+	3830423B21	4922505A21	small EDRK-rich factor 2
Mouse	chr2:121232753-121238599:-	BC026459	A930015D22	hypothetical TMS membrane protein/tumour differentially expressed protein (TDE) containing protein
Human	chr15:42616558-42644124:+	AK002006	U97670	Homo sapiens eukaryotic translation initiation factor eIF3, p35 subunit mRNA, complete cds.
Human	chr15:42641845-42743154:-	BC024161	AB058743	Homo sapiens mRNA for KIAA1840 protein, partial cds.
Mouse	chr2:121777569-121805621:+	B230022C21	F630111P04	eukaryotic translation initiation factor 3, subunit 1 alpha
Mouse	chr2:121802543-121867431:-	BC019404	M5C1055I17	hypothetical Intradiol ring-cleavage dioxygenase/WD40-like containing protein
Human	chr15:42646831-42647779:+	AA027121	-	-
Human	chr15:42641845-42743154:-	BC067798	AB058743	Homo sapiens mRNA for KIAA1840 protein, partial cds.
Mouse	chr2:121777569-121805621:+	B230022C21	F630111P04	eukaryotic translation initiation factor 3, subunit 1 alpha
Mouse	chr2:121802543-121867431:-	BC019404	M5C1055I17	hypothetical Intradiol ring-cleavage dioxygenase/WD40-like containing protein
Human	chr15:43601326-43665780:+	CF137512	-	-
Human	chr15:43562613-43602294:-	BC026089	BC026089	Homo sapiens solute carrier family 30 (zinc transporter), member 4, mRNA (cDNA clone MGC:27012 IMAGE:4829839), complete cds.
Mouse	chr2:122451070-122470477:+	BM935220	G730032J11	unclassifiable
Mouse	chr2:122430256-122451662:-	5930401A14	5930401A14	solute carrier family 30 (zinc transporter), member 4
Human	chr15:46200461-46222079:+	AF348468	AF348468	Homo sapiens ion transporter JSX (JSX) mRNA, complete cds.
Human	chr15:46214118-46216264:-	BX537526	BX537526	Homo sapiens mRNA; cDNA DKFZp686E0292 (from clone DKFZp686E0292).
Mouse	chr2:124817147-124837847:+	F630045L20	F630045L20	hypothetical K+-dependent Na+/Ca+ exchanger related-protein containing protein
Mouse	chr2:124829998-124831493:-	6720430A17	6720430A17	unclassifiable
Human	chr15:46200461-46222079:+	AF348468	AF348468	Homo sapiens ion transporter JSX (JSX) mRNA, complete cds.
Human	chr15:46214118-46216264:-	BX537526	BX537526	Homo sapiens mRNA; cDNA DKFZp686E0292 (from clone DKFZp686E0292).
Mouse	chr2:124817147-124837847:+	F630045L20	F630045L20	hypothetical K+-dependent Na+/Ca+ exchanger related-protein containing protein
Mouse	chr2:124831590-124832998:-	BI738410	-	-
Human	chr15:46200461-46222079:+	AF348468	AF348468	Homo sapiens ion transporter JSX (JSX) mRNA, complete cds.
Human	chr15:46218916-46257850:-	BX647197	AB037762	Homo sapiens mRNA for KIAA1341 protein, partial cds.
Mouse	chr2:124817147-124837847:+	F630045L20	F630045L20	hypothetical K+-dependent Na+/Ca+ exchanger related-protein containing protein
Mouse	chr2:124833614-124872684:-	9430071B01	E130320N24	myelin basic protein expression factor 2, repressor

Human	chr15:46222147-46222945:+	BE327994	-	-
Human	chr15:46218916-46257850:-	AB037762	AB037762	Homo sapiens mRNA for KIAA1341 protein, partial cds.
Mouse	chr2:124817147-124837847:+	F630045L20	F630045L20	hypothetical K+-dependent Na+/Ca+ exchanger related-protein containing protein
Mouse	chr2:124833614-124872684:-	9430071B01	E130320N24	myelin basic protein expression factor 2, repressor
Human	chr15:47235111-47447228:+	AL832745	AL832745	Homo sapiens mRNA; cDNA DKFZp686P1613 (from clone DKFZp686P1613).
Human	chr15:47406451-47700407:-	AK023699	AK057362	Homo sapiens cDNA FLJ32800 fis, clone TESTI2002544.
Mouse	chr2:125608132-125733321:+	C230059B13	C230059B13	N-ACETYLGALACTOSAMINE KINASE (EC 2.7.1.-) (GALNAC KINASE) (GALACTOKINASE 2) homolog [Homo sapiens]
Mouse	chr2:125732506-125738065:-	CF106436	-	-
Human	chr15:53398450-53435138:+	AK093138	CR591869	full-length cDNA clone CS0DE003YI24 of Placenta of Homo sapiens (human).
Human	chr15:53419527-53487945:-	BC042826	AK022459	Homo sapiens cDNA FLJ12397 fis, clone MAMMA1002769, weakly similar to Homo sapiens cell cycle progression restoration 8 protein (CPR8) mRNA.
Mouse	chr9:73324833-73357121:-	9530050K08	6430517H14	phosphatidylinositol glycan, class B
Mouse	chr9:73302847-73333800:+	BC043049	6330521P04	Weakly similar to CCP8
Human	chr15:54323505-54525363:+	BX508946	BC028119	Homo sapiens, similar to putative, clone MGC:40181 IMAGE:5172473, mRNA, complete cds.
Human	chr15:54501039-54544627:-	BC034991	AK002084	Homo sapiens cDNA FLJ11222 fis, clone PLACE1008177.
Mouse	chr9:72762127-72828924:-	6430566E15	AB000619	Mus musculus tsec-1 mRNA, complete cds. CDS=211..1254
Mouse	chr9:72756013-72777399:+	2810408J05	2810408J05	meiosis-specific nuclear structural protein 1
Human	chr15:54711013-54737062:+	BI255921	-	-
Human	chr15:54709666-54817393:-	BC040532	BC040532	Homo sapiens, Similar to hypothetical protein FLJ20095, clone IMAGE:5261474, mRNA.
Mouse	chr9:72679821-72728156:-	A930005F02	A930005F02	unclassifiable
Mouse	chr9:72593416-72681863:+	I920030K07	I920030K07	hypothetical protein
Human	chr15:62230967-62236733:+	AK126655	AK126655	Homo sapiens cDNA FLJ44699 fis, clone BRACE3016020.
Human	chr15:62233942-62242457:-	CR599100	BC008848	Homo sapiens peptidylprolyl isomerase B (cyclophilin B), mRNA (cDNA clone MGC:14109 IMAGE:3502055), complete cds.
Mouse	chr9:66306007-66310592:-	6330551A05	6330551A05	similar to SORTING NEXIN SNX22 [Homo sapiens]
Mouse	chr9:66300972-66307462:+	BC013061	AK128977	Mus musculus cDNA fis, clone TRACH3016614, moderately similar to Peptidyl-prolyl cis-trans isomerase B precursor (EC 5.2.1.8). CDS=48..476
Human	chr15:64584484-64653492:+	AK027468	BX640701	Homo sapiens mRNA; cDNA DKFZp686A05136 (from clone DKFZp686A05136).
Human	chr15:64626860-64645356:-	AX746525	AX746525	Sequence 50 from Patent EP1308459.
Mouse	chr9:64374720-64410331:-	BC027435	BC027435	Mus musculus RIKEN cDNA 2310031L18 gene, mRNA (cDNA clone MGC:35649 IMAGE:2812472), complete cds. CDS=10..1779
Mouse	chr9:64354723-64375692:+	AF309072	AF309072	Mus musculus Klotho-LPH related protein (Klph) mRNA, complete cds. CDS=275..1975
Human	chr15:68932672-69129490:+	BC026157	AK123672	Homo sapiens cDNA FLJ41678 fis, clone HCASM2003076.
Human	chr15:68960735-68972152:-	BX441483	BC072414	Homo sapiens THAP domain containing 10, mRNA (cDNA clone MGC:87938 IMAGE:6167441), complete cds.
Mouse	chr9:60778469-60897472:-	BC016574	BC016574	Mus musculus RIKEN cDNA D430025H09 gene, mRNA (cDNA clone MGC:27966 IMAGE:3594577), complete cds. CDS=43..2103
Mouse	chr9:60897033-60899711:+	B930082K07	B930082K07	unclassifiable
Human	chr15:72677896-72715681:+	AK091692	L29217	Homo sapiens clk3 mRNA, complete cds.
Human	chr15:72692588-72694990:-	BG773262	-	-
Mouse	chr9:57956113-58042305:-	I530001F08	BC049776	Mus musculus mRNA similar to dead ringer homolog 2 (Drosophila) (cDNA clone MGC:59291 IMAGE:6394061), complete cds. CDS=303..1151
Mouse	chr9:57970715-57973538:+	L230038K21	-	-
Human	chr15:73447519-73448978:+	BC010081	BC010081	Homo sapiens mannosidase, alpha, class 2C, member 1, mRNA (cDNA clone IMAGE:4329693), partial cds.
Human	chr15:73430986-73448018:-	BC038594	BC038594	Homo sapiens, Similar to mannosidase, alpha, class 2C, member 1, clone IMAGE:5527998, mRNA.
Mouse	chr9:57280613-57335485:-	5730407N16	5730407N16	unclassifiable
Mouse	chr9:57334885-57346917:+	F630112C22	E430007I19	mannosidase, alpha, class 2C, member 1
Human	chr15:73548762-73554715:+	AK126088	AK126088	Homo sapiens cDNA FLJ44100 fis, clone TESTI4043710.
Human	chr15:73546515-73658825:-	M83738	M83738	Human protein-tyrosine phosphatase (PTPase MEG2) mRNA, complete cds.
Mouse	chr9:57260825-57276161:-	F530012N16	F530012N16	hypothetical protein
Mouse	chr9:57199112-57267016:+	BC053017	BC053017	Mus musculus cDNA clone MGC:62214 IMAGE:5708923, complete cds. CDS=627..2408
Human	chr15:79262147-79393460:+	AK128740	AL833554	Homo sapiens mRNA; cDNA DKFZp686P063 (from clone DKFZp686P063).
Human	chr15:79380532-79381158:-	AI457472	-	-
Mouse	chr7:70684985-70885703:-	A130012E11	A130012E11	interleukin 16
Mouse	chr7:70771975-70793248:+	D330012B13	D330012B13	interleukin 16
Human	chr15:81216370-81223014:+	BX538250	BX538250	Homo sapiens mRNA; cDNA DKFZp686A1429 (from clone DKFZp686A1429).
Human	chr15:81221171-81271875:-	AL833295	AL833295	Homo sapiens mRNA; cDNA DKFZp451H129 (from clone DKFZp451H129).
Mouse	chr7:68615595-68623547:+	2900076A07	2900076A07	unclassifiable
Mouse	chr7:68622352-68624566:-	D830021B09	D830021B09	unclassifiable

Human	chr15:81567328-81604597:+	BC063487	BC063487	Homo sapiens transmembrane 6 superfamily member 1, mRNA (cDNA clone MGC:74918 IMAGE:5240735), complete cds.
Human	chr15:81593875-81596498:-	AK055438	AK055438	Homo sapiens cDNA FLJ30876 fis, clone FEBRA2004412.
Mouse	chr7:68939142-68976493:+	9630017P07	9630017P07	transmembrane 6 superfamily member 1
Mouse	chr7:68973301-69026523:-	9330209E08	9330209E08	hepatoma-derived growth factor, related protein 3
Human	chr15:81567328-81604597:+	BC063487	BC063487	Homo sapiens transmembrane 6 superfamily member 1, mRNA (cDNA clone MGC:74918 IMAGE:5240735), complete cds.
Human	chr15:81596516-81667774:-	AL109779	BC040554	Homo sapiens, Similar to likely ortholog of mouse hepatoma-derived growth factor, related protein 3, clone IMAGE:5303793, mRNA.
Mouse	chr7:68939142-68976493:+	9630017P07	9630017P07	transmembrane 6 superfamily member 1
Mouse	chr7:68973301-69026523:-	9330209E08	9330209E08	hepatoma-derived growth factor, related protein 3
Human	chr15:87588184-87661964:+	AK027564	AK055176	Homo sapiens cDNA FLJ30614 fis, clone CTONG2001139.
Human	chr15:87660538-87679076:-	AK130624	X98093	H.sapiens mRNA for DNA polymerase gamma, mitochondrial protein.
Mouse	chr7:66471716-66530078:+	BC027836	BC027836	Mus musculus cDNA sequence BC025462, mRNA (cDNA clone MGC:38195 IMAGE:5322726), complete cds. CDS=70..1254
Mouse	chr7:66526030-66546072:-	9430069H22	5330409N10	polymerase (DNA directed), gamma
Human	chr15:87929960-87975290:+	BC002881	AK123612	Homo sapiens cDNA FLJ41618 fis, clone CTONG3003905.
Human	chr15:87972205-87992578:-	AY358384	AY358384	Homo sapiens clone DNA47465 EQYK340 (UNQ340) mRNA, complete cds.
Mouse	chr7:66738813-66776714:+	BC028450	9830163L23	hypothetical protein
Mouse	chr7:66776664-66785609:-	BC034093	BC034093	Mus musculus, clone IMAGE:4484074, mRNA.
Human	chr15:89274414-89298327:+	BQ432658	BC045635	Homo sapiens, Similar to expressed sequence AW538196, clone IMAGE:5297091, mRNA.
Human	chr15:89275107-89276785:-	CR625970	BC033794	Homo sapiens, Similar to RIKEN cDNA 1110033O09 gene, clone MGC:45386 IMAGE:5122164, mRNA, complete cds.
Mouse	chr7:67425614-67429526:-	6330436H02	6330436H02	weakly similar to nonhistone chromosomal protein HMG-2 (fragments) [Bos primigenius taurus]
Mouse	chr7:67424637-67432198:+	A830036O08	BC038310	Mus musculus, similar to RIKEN cDNA 1110033O09 gene, clone IMAGE:5119020, mRNA.
Human	chr15:89310162-89332853:+	BI829394	-	-
Human	chr15:89310272-89338834:-	BC003138	BC005140	Homo sapiens protein regulator of cytokinesis 1, mRNA (cDNA clone MGC:1671 IMAGE:3504761), complete cds.
Mouse	chr7:67374680-67405931:-	B930093B02	B930093B02	similar to HECT DOMAIN AND RLD 2 [Homo sapiens]
Mouse	chr7:67375902-67397733:+	E430020K16	E430020K16	protein regulator of cytokinesis 1
Human	chr16:67018-75852:+	CR612592	CR612592	full-length cDNA clone CS0DC013YB16 of Neuroblastoma Cot 25-normalized of Homo sapiens (human).
Human	chr16:74267-128859:-	X90857	X90857	H.sapiens mRNA for -14 gene, containing globin regulatory element.
Mouse	chr11:32122062-32128271:+	BC014754	BC014754	Mus musculus N-methylpurine-DNA glycosylase, mRNA (cDNA clone MGC:25347 IMAGE:4486938), complete cds. CDS=31..1032
Mouse	chr11:32121199-32163278:-	G630038I01	G630038I01	C16ORF35 homolog [Mus musculus]
Human	chr16:670088-672800:+	CR593928	AF217968	Homo sapiens clone PP1131 unknown mRNA.
Human	chr16:671668-680416:-	BC073785	AL136863	Homo sapiens mRNA; cDNA DKFZp434F054 (from clone DKFZp434F054); complete cds.
Mouse	chr17:24429326-24435987:-	AF129086	BC027427	Mus musculus STIP1 homology and U-Box containing protein 1, mRNA (cDNA clone MGC:35920 IMAGE:4191866), complete cds. CDS=509..1423
Mouse	chr17:24427559-24430535:+	F830034L21	F830034L21	hypothetical protein
Human	chr16:778623-788077:+	BC006278	AK128869	Homo sapiens cDNA FLJ46847 fis, clone UTERU3005264.
Human	chr16:788032-790725:-	AB030207	AB030207	Homo sapiens mRNA for G gamma subunit, complete cds, clone:h2-35.
Mouse	chr17:24317457-24325950:-	D630020J05	D630020J05	hypothetical protein
Mouse	chr17:24315699-24318351:+	BC048431	AY029485	Mus musculus G protein gamma subunit 13 mRNA, complete cds. CDS=90..293
Human	chr16:1143242-1211773:+	AF070604	AF073931	Homo sapiens low-voltage activated calcium channel alpha 1H mRNA, complete cds.
Human	chr16:1211655-1405893:-	AF175522	AF175522	Homo sapiens transmembrane tryptase mRNA, complete cds.
Mouse	chr17:23972667-24032167:-	AF051947	M5C1001I06	calcium channel, voltage-dependent, T type, alpha 1H subunit
Mouse	chr17:23967655-23972824:+	1110058D23	1110058D23	tryptase gamma 1
Human	chr16:1323647-1339443:+	AL834321	AL834321	Homo sapiens mRNA; cDNA DKFZp761G05121 (from clone DKFZp761G05121).
Human	chr16:1339242-1341913:-	BC011785	BC016699	Homo sapiens, clone MGC:22731 IMAGE:4097703, mRNA, complete cds.
Mouse	chr17:23841041-23854663:-	AK122358	AK122358	Mus musculus mRNA for mKIAA0734 protein. CDS=101..3785
Mouse	chr17:23838552-23841181:+	BC006899	K230309N03	hypothetical Possible metal-binding region in RNase L inhibitor, RLI containing protein
Human	chr16:1341925-1353353:+	CR618970	BC014592	Homo sapiens, hypothetical protein CAB56184, clone MGC:26282 IMAGE:4824054, mRNA, complete cds.
Human	chr16:1353206-1404744:-	AK124977	AK124977	Homo sapiens cDNA FLJ42987 fis, clone BRTHA2008669, moderately similar to Homo sapiens unkempt-like (Drosophila) (UNKL).
Mouse	chr17:23831713-23838522:-	6430527N14	6430527N14	hypothetical Extracytoplasmic domain of cation-dependent mannose 6-phosphate receptor structure containing protein
Mouse	chr17:23786772-23832825:+	1300004G08	BC059910	Mus musculus RIKEN cDNA 1300004G08 gene, mRNA (cDNA clone IMAGE:6825134), partial cds.
Human	chr16:1353523-1354374:+	BI751989	-	-
Human	chr16:1353206-1404744:-	AK124977	AK124977	Homo sapiens cDNA FLJ42987 fis, clone BRTHA2008669, moderately similar to Homo sapiens unkempt-like (Drosophila) (UNKL).
Mouse	chr17:23831713-23838522:-	6430527N14	6430527N14	hypothetical Extracytoplasmic domain of cation-dependent mannose 6-phosphate receptor structure containing protein
Mouse	chr17:23786772-23832825:+	1300004G08	BC059910	Mus musculus RIKEN cDNA 1300004G08 gene, mRNA (cDNA clone IMAGE:6825134), partial cds.

Human	chr16:1763328-1771712:+	BC041011	AK074080	Homo sapiens mRNA for FLJ00151 protein.
Human	chr16:1766712-1783735:-	AK074190	AK057850	Homo sapiens cDNA FLJ25121 fis, clone CBR06051.
Mouse	chr17:23486397-23535220:-	D030072I11	M5C1050C12	mitogen-activated protein kinase 8 interacting protein 3
Mouse	chr17:23484873-23490382:+	2310012N15	3300001M01	SPRY DOMAIN-CONTAINING SOCS BOX PROTEIN SSB-3 homolog [Mus musculus]
Human	chr16:1816969-1830485:+	CN260930	BC063017	Homo sapiens cDNA clone MGC:74876 IMAGE:5226963, complete cds.
Human	chr16:1785622-1817196:-	CR620966	BC002627	Homo sapiens hydroxyacylglutathione hydrolase, mRNA (cDNA clone MGC:3589 IMAGE:3608098), complete cds.
Mouse	chr17:23446619-23448091:-	BC011330	D130077P09	Similar to homoprotocatechuate catabolism bifunctional isomerase/decarboxylase homolog
Mouse	chr17:23437801-23462368:+	K330040H06	I920088O22	Hydroxyacylglutathione hydrolase (EC 3.1.2.6) (Glyoxalase II) (Glx II)
Human	chr16:1962001-1971788:+	AK096738	BC010231	Homo sapiens, clone MGC:17508 IMAGE:3455740, mRNA, complete cds.
Human	chr16:1968914-1972756:-	BC015917	BC015917	Homo sapiens NADPH oxidase organizer 1, transcript variant a, mRNA (cDNA clone MGC:20258 IMAGE:4661469), complete cds.
Mouse	chr17:23295350-23305061:-	9830139K06	9830139K06	transducin (beta)-like 3
Mouse	chr17:23293635-23297931:+	F930014D17	F930014D17	NADPH oxidase organizer 1
Human	chr16:2195206-2199423:+	AK022227	AK021536	Homo sapiens cDNA FLJ11474 fis, clone HEMBA1001723, highly similar to Homo sapiens G protein beta subunit mRNA.
Human	chr16:2199255-2204844:-	BC039154	CR607189	full-length cDNA clone CS0DK007YF11 of HeLa cells Cot 25-normalized of Homo sapiens (human).
Mouse	chr17:23069450-23074980:-	5730519J11	A430060P14	G protein beta subunit-like
Mouse	chr17:23069784-23071369:+	9930021D14	9930021D14	hypothetical Haem peroxidase superfamily containing protein
Human	chr16:2265910-2266572:+	BM990801	-	-
Human	chr16:2265879-2330618:-	X97187	U78735	Human ABC3 mRNA, complete cds.
Mouse	chr17:23000531-23010780:-	A130072E10	A130072E10	hypothetical protein
Mouse	chr17:22948259-23006466:+	BC042663	F630103K24	ATP-binding cassette, sub-family A (ABC1), member 3
Human	chr16:2953946-2958382:+	BC009383	BC009383	Homo sapiens kringle containing transmembrane protein 2, transcript variant 3, mRNA (cDNA clone MGC:16709 IMAGE:4127918), complete cds.
Human	chr16:2955903-2960243:-	BX391057	-	-
Mouse	chr17:22336105-22340734:-	AJ457192	AJ457192	Mus musculus mRNA for Kremen2 protein (Kremen2 gene). CDS=182..1567
Mouse	chr17:22338139-22349747:+	9930118B15	9930118B15	kringle containing transmembrane protein 2
Human	chr16:2959247-2963491:+	BC033703	CR623107	full-length cDNA clone CS0DN002YG04 of Adult brain of Homo sapiens (human).
Human	chr16:2962793-2970506:-	CR597027	CR603670	full-length cDNA clone CS0DI007YP18 of Placenta Cot 25-normalized of Homo sapiens (human).
Mouse	chr17:22331091-22335303:-	BC011185	1500004C10	hypothetical Uncharacterised protein family Hly-III/UPF0073 containing protein
Mouse	chr17:22321219-22331642:+	AF175892	K230010M16	Membrane-associated tyrosine-and threonine-specific cdc2-inhibitory kinase
Human	chr16:3014021-3017757:+	CR624641	CR624641	full-length cDNA clone CS0DL007YK18 of B cells (Ramos cell line) Cot 25-normalized of Homo sapiens (human).
Human	chr16:3012622-3014303:-	BC065514	BC065514	Homo sapiens cDNA clone IMAGE:5748501, partial cds.
Mouse	chr17:22263502-22268782:-	F830014G06	F830014G06	similar to HYPOTHETICAL 34.8 KDA PROTEIN [Homo sapiens]
Mouse	chr17:22268506-22270429:+	2810484E09	2810484E09	FIBROBLAST GROWTH FACTOR REGULATED PROTEIN 2
Human	chr16:3601730-3670145:+	BC064332	BC064332	Homo sapiens cDNA clone IMAGE:5805504, partial cds.
Human	chr16:3640638-3707573:-	BC035759	AK093344	Homo sapiens cDNA FLJ36025 fis, clone TESTI2016701, highly similar to TUMOR NECROSIS FACTOR TYPE 1 RECEPTOR ASSOCIATED PROTEIN.
Mouse	chr16:3720273-3723358:+	BC014718	BC030394	Mus musculus deoxyribonuclease I, mRNA (cDNA clone MGC:40629 IMAGE:4913032), complete cds. CDS=165..1163
Mouse	chr16:3716165-3761168:-	I1C0037K05	E860021P12	Heat shock protein 75 kDa, mitochondrial precursor (HSP 75) (Tumor necrosis factor type 1 receptor associated protein) (TRAP-1) (TNFR- associated protein 1)
Human	chr16:4948319-5076277:+	BC031095	AB007880	Homo sapiens KIAA0420 mRNA, complete cds.
Human	chr16:5074302-5087790:-	BC010084	BC010084	Homo sapiens hypothetical protein SB153, mRNA (cDNA clone MGC:19636 IMAGE:2822323), complete cds.
Mouse	chr16:4915044-4926332:+	BC011281	I530009P21	asparagine-linked glycosylation 1 homolog (yeast, beta-1,4-mannosyltransferase)
Mouse	chr16:4925575-4937379:-	5330411D15	5330411D15	8 days embryo whole body cDNA, RIKEN full-length enriched library, clone:5730409G15 product:hypothetical S-adenosyl-L-methionine-dependent methyltransferases structure containing protein, full insert sequence (Fragment) homolog [M
Human	chr16:10745144-10770711:+	CR613650	CR613650	full-length cDNA clone CS0DC007YE05 of Neuroblastoma Cot 25-normalized of Homo sapiens (human).
Human	chr16:10762278-10820122:-	BC050569	BC050569	Homo sapiens, clone IMAGE:5241448, mRNA.
Mouse	chr16:10102334-10115723:+	BC047926	BC055436	Mus musculus nucleotide binding protein 1, mRNA (cDNA clone MGC:65267 IMAGE:3660324), complete cds. CDS=28..990
Mouse	chr16:10109929-10137758:-	6330539F10	6330539F10	unclassifiable
Human	chr16:11669771-11680516:+	AL161976	BC036443	Homo sapiens, stannin, clone MGC:26142 IMAGE:4823944, mRNA, complete cds.
Human	chr16:11680437-11744235:-	BC013727	BC002856	Homo sapiens hypothetical protein LOC51061, mRNA (cDNA clone MGC:4177 IMAGE:3635977), complete cds.
Mouse	chr16:10755348-10769505:+	BC006961	BC006961	Mus musculus, RIKEN cDNA 2810407J07 gene, clone IMAGE:3707801, mRNA.
Mouse	chr16:10769404-10829143:-	BC020363	BC020363	Mus musculus RIKEN cDNA 2810408E11 gene, mRNA (cDNA clone IMAGE:3498473).
Human	chr16:19635279-19776417:+	AX748356	AF520570	Homo sapiens unknown mRNA.
Human	chr16:19476769-19803740:-	BG431910	AF202640	Homo sapiens orphan G-protein coupled receptor (GPRC5B) mRNA, complete cds.
Mouse	chr7:106152565-106269115:+	A230094G09	A230094G09	hypothetical IQ calmodulin-binding region/IQ motif profile/Arginine-rich region profile containing protein
Mouse	chr7:106268867-106292038:-	AF378831	AF378831	Mus musculus orphan G protein-coupled receptor (Raig2) mRNA, complete cds. CDS=126..1358

Human	chr16:20593237-20716404:+	D16350	D16350	Human SA mRNA for SA gene product, complete cds.
Human	chr16:20652487-20819181:-	BC010503	BX648185	Homo sapiens mRNA; cDNA DKFZp686G23203 (from clone DKFZp686G23203).
Mouse	chr7:107057547-107084137:+	F530003P09	F530003P09	SA rat hypertension-associated homolog
Mouse	chr7:107065303-107090682:-	4930518K06	4930518K06	RIKEN cDNA 4933424N09 gene
Human	chr16:23371583-23376428:+	CD176148	-	-
Human	chr16:23307312-23372004:-	BP308365	AY358632	Homo sapiens clone DNA125181 COG7 (UNQ3082) mRNA, complete cds.
Mouse	chr7:109279172-109286805:+	AL362276	-	-
Mouse	chr7:109177052-109279404:-	A730033G19	A730033G19	Conserved oligomeric Golgi complex component 7 homolog [Homo sapiens]
Human	chr16:28797305-28823331:+	BX537784	BX537784	Homo sapiens mRNA; cDNA DKFZp779O2152 (from clone DKFZp779O2152); complete cds.
Human	chr16:28804710-28844897:-	AK026935	AY129017	Homo sapiens clone FP18346 unknown mRNA.
Mouse	chr7:113920574-113937826:-	4733401N01	AY081946	Mus musculus calcium-transporting ATPase (Atp2a1) mRNA, complete cds. CDS=183..3167
Mouse	chr7:113903462-113920625:+	4831410J04	BC015287	Mus musculus RIKEN cDNA 2610011A08 gene, mRNA (cDNA clone MGC:18901 IMAGE:4240442), complete cds. CDS=374..1846
Human	chr16:29782415-29786875:+	BM909113	AK131031	Homo sapiens cDNA FLJ27521 fis, clone TST09269.
Human	chr16:29777178-29782558:-	AK097691	AK097691	Homo sapiens cDNA FLJ40372 fis, clone TESTI2034940, highly similar to CDP-DIACYLGLYCEROL--INOSITOL 3-PHOSPHATIDYLTRANSFERASE (EC 2.7.8.11).
Mouse	chr7:114442801-114447179:-	E030037N21	D830044I16	unclassifiable
Mouse	chr7:114446724-114451582:+	4930583C08	4930583C08	CDP-DIACYLGLYCEROL--INOSITOL 3-PHOSPHATIDYLTRANSFERASE (EC 2.7.8.11) (PHOSPHATIDYLINOSITOL SYNTHASE) (PTDINS SYNTHASE) (PI SYNTHASE) homolog [Rattus norvegicus]
Human	chr16:29819201-29838686:+	AF070642	AF070642	Homo sapiens clone 24488 mRNA sequence.
Human	chr16:29825158-29845054:-	AK123825	BC036228	Homo sapiens, Similar to MSTP028 protein, clone MGC:39734 IMAGE:5276078, mRNA, complete cds.
Mouse	chr7:114416648-114420983:-	6330589E13	A830007L07	hypothetical protein
Mouse	chr7:114399945-114441691:+	AF534881	9330155G24	TYPE I TRANSMEMBRANE RECEPTOR PRECURSOR homolog [Homo sapiens]
Human	chr16:29914175-29924615:+	CR620749	CR618846	full-length cDNA clone CS0DI001YD02 of Placenta Cot 25-normalized of Homo sapiens (human).
Human	chr16:29911812-29914896:-	AK075145	AK075145	Homo sapiens cDNA FLJ90664 fis, clone PLACE1005239, moderately similar to Homo sapiens mRNA for HIRIP3 protein.
Mouse	chr7:114322666-114333455:-	BC055928	BC025527	Mus musculus expressed sequence AI854876, mRNA (cDNA clone IMAGE:5323944), with apparent retained intron.
Mouse	chr7:114333021-114336463:+	BC055687	BC055687	Mus musculus hypothetical protein B130036O03, mRNA (cDNA clone MGC:66546 IMAGE:6414655), complete cds. CDS=481..2286
Human	chr16:29914175-29924615:+	BC035693	CR618846	full-length cDNA clone CS0DI001YD02 of Placenta Cot 25-normalized of Homo sapiens (human).
Human	chr16:29924336-29942092:-	BC055284	D31897	Homo sapiens mRNA for Doc2, complete cds.
Mouse	chr7:114322666-114333455:-	6820444P08	BC025527	Mus musculus expressed sequence AI854876, mRNA (cDNA clone IMAGE:5323944), with apparent retained intron.
Mouse	chr7:114318574-114323790:+	BC055768	BC055768	Mus musculus double C2, alpha, mRNA (cDNA clone MGC:67326 IMAGE:6413208), complete cds. CDS=652..1869
Human	chr16:30015270-30024015:+	AK097453	AK097453	Homo sapiens cDNA FLJ40134 fis, clone TESTI2012308.
Human	chr16:30023630-30042328:-	AK026256	CR603463	full-length cDNA clone CS0DN005YA14 of Adult brain of Homo sapiens (human).
Mouse	chr7:114245962-114247678:-	9530079E12	9530079E12	hypothetical protein
Mouse	chr7:114237143-114246505:+	1110015E22	1110015E22	hypothetical Glycerophosphoryl diester phosphodiesterase containing protein
Human	chr16:30617031-30664018:+	BF313701	AB002307	Homo sapiens mRNA for KIAA0309 gene, partial cds.
Human	chr16:30616526-30617880:-	BI763942	-	-
Mouse	chr7:114983083-115007457:+	F630005G17	F630004O05	Transcriptional activator SRCAP homolog [Homo sapiens]
Mouse	chr7:114981553-114983940:-	1700008J07	1700008J07	hypothetical protein
Human	chr16:30694287-30695129:+	BM473700	-	-
Human	chr16:30692354-30694855:-	AK057076	AK057076	Homo sapiens cDNA FLJ32514 fis, clone SMINT1000091.
Mouse	chr7:115059779-115075036:+	A730013O04	F630113L02	ring finger protein 40
Mouse	chr7:115074982-115075526:-	1700086F19	1700120K04	RIKEN cDNA 1700120K04 gene
Human	chr16:30694287-30695129:+	BM473700	-	-
Human	chr16:30692354-30694855:-	AK057076	AK057076	Homo sapiens cDNA FLJ32514 fis, clone SMINT1000091.
Mouse	chr7:115075124-115075877:+	BC028646	BC028646	Mus musculus, clone IMAGE:1328707, mRNA.
Mouse	chr7:115074982-115075526:-	1700113F03	1700120K04	RIKEN cDNA 1700120K04 gene
Human	chr16:30876116-30903486:+	AB002337	AB002337	Homo sapiens mRNA for KIAA0339 protein, partial cds.
Human	chr16:30903457-30907092:-	BC022562	AL834347	Homo sapiens mRNA; cDNA DKFZp761C1717 (from clone DKFZp761C1717).
Mouse	chr7:115256706-115274963:+	5730419I01	BC049883	Mus musculus cDNA sequence BC010250, mRNA (cDNA clone MGC:55143 IMAGE:5387990), complete cds. CDS=130..2679
Mouse	chr7:115270496-115273922:-	9430064I24	9430064I24	hypothetical protein
Human	chr16:30904006-30907974:+	AK057436	BC004929	Homo sapiens, 3 beta-hydroxy-delta 5-C27-steroid oxidoreductase, clone MGC:10497 IMAGE:3626629, mRNA, complete cds.
Human	chr16:30903457-30907092:-	AL834347	AL834347	Homo sapiens mRNA; cDNA DKFZp761C1717 (from clone DKFZp761C1717).
Mouse	chr7:115256706-115274963:+	5730419I01	BC049883	Mus musculus cDNA sequence BC010250, mRNA (cDNA clone MGC:55143 IMAGE:5387990), complete cds. CDS=130..2679
Mouse	chr7:115270496-115273922:-	9430064I24	9430064I24	hypothetical protein

Human	chr16:30993265-31002334:+	BC035589	BC035589	Homo sapiens, KIAA0296 gene product, clone MGC:45126 IMAGE:5532753, mRNA, complete cds.
Human	chr16:31002246-31014455:-	CR618714	AY358456	Homo sapiens clone DNA40571 EDTP308 (UNQ308) mRNA, complete cds.
Mouse	chr7:115347307-115357156:+	6820429M01	6820429M01	similar to HYPOTHETICAL ZINC FINGER PROTEIN KIAA0296 [Homo sapiens]
Mouse	chr7:115357000-115362221:-	CF587233	BC039632	Mus musculus cDNA sequence BC039632, mRNA (cDNA clone IMAGE:5124409), partial cds. CDS=3..1283
Human	chr16:31377818-31385989:+	AY217348	AY217348	Homo sapiens FLJ00019-like protein mRNA, complete cds.
Human	chr16:31359130-31378148:-	AK125571	AK125571	Homo sapiens cDNA FLJ43583 fis, clone SKNMC2006173.
Mouse	chr7:115711841-115719663:+	I420101H07	BC032200	Mus musculus cDNA sequence BC032200, mRNA (cDNA clone MGC:36606 IMAGE:5342166), complete cds. CDS=116..2896
Mouse	chr7:115709009-115712592:-	D130085G03	D130085G03	similar to MSZF76 (FRAGMENT) [Mus musculus]
Human	chr16:31401820-31409592:+	M95549	M95549	Homo sapiens sodium/glucose cotransporter-like protein mRNA, complete cds.
Human	chr16:31406018-31427225:-	BC054514	BC054514	Homo sapiens cDNA clone IMAGE:4153640, partial cds.
Mouse	chr7:115740707-115747482:+	BC022226	AK128900	Mus musculus cDNA fis, clone TRACH2018380, highly similar to SODIUM/GLUCOSE COTRANSPORTER 2.
Mouse	chr7:115746428-115773298:-	BC061499	D330006P18	hypothetical protein
Human	chr16:52025862-52083062:+	X74594	BC034490	Homo sapiens, clone MGC:26535 IMAGE:4838444, mRNA, complete cds.
Human	chr16:52070370-52072963:-	AK123139	AK123139	Homo sapiens cDNA FLJ41144 fis, clone BRACE2036830.
Mouse	chr8:90391666-90445306:+	BC020528	BC020528	Mus musculus retinoblastoma-like 2, mRNA (cDNA clone MGC:18400 IMAGE:4240592), complete cds. CDS=68..3475
Mouse	chr8:90427358-90522413:-	5730568E19	5730568E19	fused toes
Human	chr16:52025862-52083062:+	AK129969	BC034490	Homo sapiens, clone MGC:26535 IMAGE:4838444, mRNA, complete cds.
Human	chr16:52082406-52095824:-	CR602186	AK023320	Homo sapiens cDNA FLJ13258 fis, clone OVARC1000862, moderately similar to M.musculus mRNA for FT1.
Mouse	chr8:90391666-90445306:+	BC020528	BC020528	Mus musculus retinoblastoma-like 2, mRNA (cDNA clone MGC:18400 IMAGE:4240592), complete cds. CDS=68..3475
Mouse	chr8:90427358-90522413:-	5730568E19	5730568E19	fused toes
Human	chr16:54782698-54948857:+	AA211156	BC030027	Homo sapiens guanine nucleotide binding protein (G protein), alpha activating activity polypeptide O, mRNA (cDNA clone MGC:33450 IMAGE:4791738), complete cds.
Human	chr16:54784027-54785938:-	AK124129	AK124129	Homo sapiens cDNA FLJ42135 fis, clone TESTI2035309.
Mouse	chr8:93156533-93316293:+	1700127H18	M36777	Mouse GTP-binding protein alpha subunit (G0A-alpha) mRNA, complete cds. CDS=22..1086
Mouse	chr8:93158028-93159919:-	4930488L21	4930488L21	inferred: RIKEN cDNA 4930488L21 gene / putative [Mus musculus]
Human	chr16:56038850-56052688:+	CR626697	BC064946	Homo sapiens hypothetical protein DKFZp434K046, mRNA (cDNA clone MGC:75177 IMAGE:6023461), complete cds.
Human	chr16:56019582-56038941:-	BC067303	AF248964	Homo sapiens CUA001 mRNA, complete cds.
Mouse	chr8:94186167-94202742:+	BU898899	F630022A16	hypothetical Homeodomain-like containing protein
Mouse	chr8:94165626-94186204:-	K530328B11	E430025G11	hypothetical S-adenosyl-L-methionine-dependent methyltransferases structure containing protein
Human	chr16:56053800-56064108:+	BC028157	BC003159	Homo sapiens, polymerase (RNA) II (DNA directed) polypeptide C (33kD), clone MGC:4212 IMAGE:2957867, mRNA, complete cds.
Human	chr16:56063331-56077922:-	CR623999	BC003541	Homo sapiens, hypothetical protein FLJ10488, clone MGC:10806 IMAGE:3611472, mRNA, complete cds.
Mouse	chr8:94205341-94221381:+	A130010H16	A130010H16	polymerase (RNA) II (DNA directed) polypeptide C
Mouse	chr8:94211674-94224176:-	BC004705	F830023M11	downstream of tyrosine kinase 4
Human	chr16:65343156-65346144:+	AL080152	AL080152	Homo sapiens mRNA; cDNA DKFZp434F104 (from clone DKFZp434F104).
Human	chr16:65312297-65343232:-	BF306383	BX648863	Homo sapiens mRNA; cDNA DKFZp686J08252 (from clone DKFZp686J08252).
Mouse	chr8:103796912-103798427:+	K230015D01	K230015D01	hypothetical Proline-rich region profile containing protein
Mouse	chr8:103772315-103797688:-	C920003I06	C130001C04	Dynein light intermediate chain 2, cytosolic (LIC53/55) (LIC-2) homolog [Homo sapiens]
Human	chr16:65525848-65540040:+	BX538086	BX538086	Homo sapiens mRNA; cDNA DKFZp686H0466 (from clone DKFZp686H0466); complete cds.
Human	chr16:65523459-65526294:-	CN363827	AF151886	Homo sapiens CGI-128 protein mRNA, complete cds.
Mouse	chr8:103997107-104011193:+	BU847445	-	-
Mouse	chr8:103995138-103997259:-	CK030814	1110019N10	hypothetical Domain of unknown function DUF59 containing protein
Human	chr16:65701345-65739948:+	BC004556	BC004556	Homo sapiens lin-10 protein homolog, mRNA (cDNA clone MGC:10534 IMAGE:3957147), complete cds.
Human	chr16:65739239-65742618:-	BC012191	BC012191	Homo sapiens, clone MGC:20513 IMAGE:4550086, mRNA, complete cds.
Mouse	chr8:104538413-104566323:+	BC057919	BC057919	Mus musculus cDNA sequence BC006874, mRNA (cDNA clone MGC:68061 IMAGE:5134302), complete cds. CDS=225..1493
Mouse	chr8:104565906-104568789:-	I920192F14	BC047209	Mus musculus, Similar to hypothetical protein MGC4655, clone IMAGE:6314722, mRNA.
Human	chr16:65761558-65767141:+	AF064599	BC012798	Homo sapiens nucleolar protein 3 (apoptosis repressor with CARD domain), mRNA (cDNA clone MGC:2009 IMAGE:3506011), complete cds.
Human	chr16:65767003-65781580:-	BC007594	AY255669	Homo sapiens unknown mRNA.
Mouse	chr8:104589409-104595207:+	E130314L04	E130314L04	apoptosis repressor with CARD domain [Homo sapiens]
Mouse	chr8:104593677-104603132:-	BC043046	BC059045	Mus musculus RIKEN cDNA 4931428F04 gene, mRNA (cDNA clone MGC:69815 IMAGE:6816353), complete cds. CDS=117..1520
Human	chr16:65869592-65880903:+	BC054486	BC082974	Homo sapiens cDNA clone IMAGE:6291175.
Human	chr16:65880829-65918165:-	BC070103	BC070103	Homo sapiens FLJ40162 protein, mRNA (cDNA clone IMAGE:5268205), partial cds.
Mouse	chr8:104686542-104696128:+	4931414L13	4931414L13	hypothetical PH domain profile/Dbl domain (dbl/cdc24 rhoGEF family)/Pleckstrin homology (PH) domain containing protein
Mouse	chr8:104696069-104698087:-	CA466336	-	-

Human	chr16:66236343-66248973:+	AB113647	AB113647	Homo sapiens RLTPR mRNA for RGD, leucine-rich repeat, tropomodulin and proline-rich containing protein, partial cds.
Human	chr16:66248916-66252214:-	AF070535	AK023726	Homo sapiens cDNA FLJ13664 fis, clone PLACE1011649.
Mouse	chr8:105004425-105011773:+	5830489H19	5830489H19	hypothetical Proline-rich region profile containing protein
Mouse	chr8:105009434-105015000:-	D630046L01	D630046L01	nuclear receptor-binding SET-domain protein 1
Human	chr16:66257476-66260162:+	AK123796	AK123796	Homo sapiens cDNA FLJ41802 fis, clone NHNPC2002565.
Human	chr16:66254349-66310825:-	CR618514	BC000757	Homo sapiens hypothetical protein MGC11335, mRNA (cDNA clone MGC:2690 IMAGE:2820364), complete cds.
Mouse	chr8:105021233-105023820:+	BU938133	4933405L10	hypothetical protein
Mouse	chr8:105017223-105021764:-	BC023921	BC023921	Mus musculus RIKEN cDNA E130303B06 gene, mRNA (cDNA clone IMAGE:5323439), with apparent retained intron.
Human	chr16:66586190-66670727:+	CR611004	AK000406	Homo sapiens cDNA FLJ20399 fis, clone KAT00581.
Human	chr16:66612678-66615271:-	AK002144	AK002144	Homo sapiens cDNA FLJ11282 fis, clone PLACE1009476, weakly similar to PUTATIVE ATP-DEPENDENT RNA HELICASE T26G10.1 IN CHROMOSOME III.
Mouse	chr8:105305586-105368139:+	5730411I06	5730411I06	hypothetical Double-stranded RNA binding (DsRBD) domain containing protein
Mouse	chr8:105323867-105326132:-	G830011O22	G830011O22	DEAD (Asp-Glu-Ala-Asp) box polypeptide 28
Human	chr16:66676243-66820667:+	H59048	CR607695	full-length cDNA clone CS0DA001YC06 of Neuroblastoma of Homo sapiens (human).
Human	chr16:66817320-66818301:-	N77274	-	-
Mouse	chr8:105373899-105444685:+	4832416C08	G270001E04	nuclear factor of activated T-cells, cytoplasmic, calcineurin-dependent 3
Mouse	chr8:105441826-105443859:-	A430034G18	A430034G18	unclassifiable
Human	chr16:66676243-66820667:+	CR607695	CR607695	full-length cDNA clone CS0DA001YC06 of Neuroblastoma of Homo sapiens (human).
Human	chr16:66820437-66827988:-	AK025571	BC069241	Homo sapiens hypothetical protein FLJ21918, mRNA (cDNA clone IMAGE:6429279), partial cds.
Mouse	chr8:105373899-105444685:+	E430019L12	G270001E04	nuclear factor of activated T-cells, cytoplasmic, calcineurin-dependent 3
Mouse	chr8:105444467-105451125:-	BU758282	BC031444	Mus musculus RIKEN cDNA 9530027K23 gene, mRNA (cDNA clone MGC:25913 IMAGE:4222237), complete cds. CDS=208..2361
Human	chr16:66855566-66893223:+	CR749475	CR749291	Homo sapiens mRNA; cDNA DKFZp686K15246 (from clone DKFZp686K15246).
Human	chr16:66889564-66902352:-	AK091674	AK091674	Homo sapiens cDNA FLJ34355 fis, clone FEBRA2012181.
Mouse	chr8:105483001-105514630:+	BF661229	G430079F19	Similar to Y+L amino acid transporter 1
Mouse	chr8:105514585-105525082:-	2010007L18	F830011P17	Metalloproteinase (Fragment) homolog [Rattus norvegicus]
Human	chr16:67778568-67916532:+	BC013021	AF132747	Homo sapiens SKD2 protein mRNA, complete cds.
Human	chr16:67913395-67931014:-	AK092372	AK056344	Homo sapiens cDNA FLJ31782 fis, clone NT2RI2008336, highly similar to Homo sapiens component of oligomeric golgi complex 8 (COG8), mRNA.
Mouse	chr8:106342340-106356878:+	F630005E18	I420002G08	vacuolar protein sorting 4a (yeast)
Mouse	chr8:106352668-106367857:-	I920034C21	C330016B07	BRAIN CDNA, CLONE MNCB-5704
Human	chr16:68705667-68753941:+	AL044613	AK000941	Homo sapiens cDNA FLJ10079 fis, clone HEMBA1001896, weakly similar to DIMETHYLGLYCINE DEHYDROGENASE PRECURSOR (EC 1.5.99.2).
Human	chr16:68750361-68764852:-	AK096066	AK096066	Homo sapiens cDNA FLJ38747 fis, clone KIDNE2012361.
Mouse	chr8:110421035-110466186:+	4930402E16	4930402E16	PYRUVATE DEHYDROGENASE PHOSPHATASE REGULATORY SUBUNIT homolog [Bos taurus]
Mouse	chr8:110455125-110471903:-	E230008O13	E230008O13	unclassifiable
Human	chr16:69252652-69257240:+	AK122592	AK122592	Homo sapiens mRNA for FLJ00418 protein.
Human	chr16:69252603-69277455:-	AB115770	AB115770	Homo sapiens ABBA-1 mRNA for actin-bundling protein with BAIAP2 homology, complete cds.
Mouse	chr8:110065293-110130815:-	K430047D07	BC016254	Mus musculus RIKEN cDNA 2010004A03 gene, mRNA (cDNA clone MGC:28891 IMAGE:4912097), complete cds. CDS=563..1270
Mouse	chr8:110045708-110065633:+	D330003B17	BC060632	Mus musculus Unknown (protein for MGC:79213), mRNA (cDNA clone MGC:79213 IMAGE:6848397), complete cds. CDS=239..2386
Human	chr16:71017348-71125289:+	AK055364	AK095618	Homo sapiens cDNA FLJ38299 fis, clone FCBBF3017255.
Human	chr16:70873944-71256409:-	AL525401	CR597739	full-length cDNA clone CS0DC011YD03 of Neuroblastoma Cot 25-normalized of Homo sapiens (human).
Mouse	chr8:108564025-108565416:-	2900056M07	2900056M07	hypothetical protein
Mouse	chr8:108565174-108589177:+	B230038E08	B230038E08	unclassifiable
Human	chr16:73590429-73702393:+	AF378524	AF378524	Homo sapiens nin283 mRNA, complete cds.
Human	chr16:73701930-73702393:-	BM987950	-	-
Mouse	chr8:110871983-110961608:+	8430426C06	8430426C06	hypothetical RING finger containing protein
Mouse	chr8:110959199-110966260:-	9230101F19	4930586D10	hypothetical FAD linked oxidase, C-terminal containing protein; DNA segment, Chr 8, Brigham & Women's Genetics 1320 expressed
Human	chr16:75826275-75882335:+	BE253544	-	-
Human	chr16:75839211-76026512:-	AA442575	AJ311903	Homo sapiens mRNA for ADAMTS18 protein (ADAMTS18 gene).
Mouse	chr8:112987728-113057998:+	4930598K05	4930481F22	hypothetical protein
Mouse	chr8:113050699-113204869:-	C130054I22	C130054I22	a disintegrin-like and metalloprotease (reprolysin type) with thrombospondin type 1 motif, 18
Human	chr16:82736043-82769865:+	BX648657	BX648657	Homo sapiens mRNA; cDNA DKFZp686O0128 (from clone DKFZp686O0128).
Human	chr16:82768949-82778163:-	L39059	BC028131	Homo sapiens, Similar to TATA box binding protein (TBP)-associated factor, RNA polymerase I, C, 110kD, clone MGC:39976 IMAGE:5205601, mRNA, complete cds.
Mouse	chr8:119004521-119039800:+	4930457P18	4930457P18	hypothetical Outer arm dynein light chain 1 structure containing protein
Mouse	chr8:119039217-119046586:-	D330013I04	D330013I04	TATA box binding protein (Tbp)-associated factor, RNA polymerase I, C

Human	chr16:87300372-87309304:+	BC021829	BC021056	Homo sapiens, clone IMAGE:2989282, mRNA.
Human	chr16:87309247-87331692:-	AK092007	D87071	Human mRNA for KIAA0233 gene, complete cds.
Mouse	chr8:121950326-121958782:+	G630097J24	G630097J24	hypothetical Adenine nucleotide alpha hydrolases structure containing protein
Mouse	chr8:121956343-122026306:-	AK129095	BC039210	Mus musculus RIKEN cDNA 2310061F22 gene, mRNA (cDNA clone IMAGE:4163995), partial cds.
Human	chr16:88314916-88333992:+	AK056405	AK056405	Homo sapiens cDNA FLJ31843 fis, clone NT2RP7000271, moderately similar to Mus musculus zinc finger protein 276 C2H2 type (Zfp276) mRNA.
Human	chr16:88328212-88410567:-	X99226	X99226	H.sapiens mRNA for FAA protein.
Mouse	chr8:122720829-122737671:+	AF178935	4932443F20	zinc finger protein (C2H2 type) 276
Mouse	chr8:122733421-122735083:-	E330014M07	E330014M07	unclassifiable
Human	chr16:88314916-88333992:+	AK056405	AK056405	Homo sapiens cDNA FLJ31843 fis, clone NT2RP7000271, moderately similar to Mus musculus zinc finger protein 276 C2H2 type (Zfp276) mRNA.
Human	chr16:88328212-88410567:-	X99226	X99226	H.sapiens mRNA for FAA protein.
Mouse	chr8:122720829-122737671:+	4932443F20	4932443F20	zinc finger protein (C2H2 type) 276
Mouse	chr8:122735366-122785720:-	AF178934	AF208116	Mus musculus fanconi anemia complementation group A (Fanca) mRNA, complete cds. CDS=26..4345
Human	chr16:88329915-88334836:+	CA426799	-	-
Human	chr16:88328212-88410567:-	X99226	X99226	H.sapiens mRNA for FAA protein.
Mouse	chr8:122720829-122737671:+	4932443F20	4932443F20	zinc finger protein (C2H2 type) 276
Mouse	chr8:122735366-122785720:-	AF178934	AF208116	Mus musculus fanconi anemia complementation group A (Fanca) mRNA, complete cds. CDS=26..4345
Human	chr16:88613602-88638880:+	BX647947	BC036781	Homo sapiens, clone IMAGE:5725898, mRNA.
Human	chr16:88633671-88641534:-	BC044232	BC044232	Homo sapiens, clone IMAGE:5722891, mRNA.
Mouse	chr7:128526813-128535281:-	C330022C24	C330022C24	unclassifiable
Mouse	chr7:128524610-128527563:+	2810420C16	1190003J15	hypothetical Transthyretin/Transthyretin-related containing protein
Human	chr17:2153710-2175648:+	AL551800	AK023169	Homo sapiens cDNA FLJ13107 fis, clone NT2RP3002501, weakly similar to THREONINE DEHYDRATASE CATABOLIC (EC 4.2.1.16).
Human	chr17:1909883-2153819:-	AB018275	AB018275	Homo sapiens mRNA for KIAA0732 protein, partial cds.
Mouse	chr11:74516268-74538555:-	F830203E10	F830203E10	serine racemase
Mouse	chr11:74538378-74777003:+	BC047279	BC047279	Mus musculus, Similar to chromosome 17 open reading frame 31, clone IMAGE:6397317, mRNA.
Human	chr17:2153710-2175648:+	AK023169	AK023169	Homo sapiens cDNA FLJ13107 fis, clone NT2RP3002501, weakly similar to THREONINE DEHYDRATASE CATABOLIC (EC 4.2.1.16).
Human	chr17:2172438-2187551:-	AB037822	AB037822	Homo sapiens mRNA for KIAA1401 protein, partial cds.
Mouse	chr11:74516268-74538555:-	0610015N12	F830203E10	serine racemase
Mouse	chr11:74509392-74521897:+	AK129350	E430031H11	hypothetical Glutamic acid-rich region profile containing protein
Human	chr17:3518528-3519740:+	CR609408	CR609408	full-length cDNA clone CS0DI057YF14 of Placenta Cot 25-normalized of Homo sapiens (human).
Human	chr17:3507690-3519369:-	BM909559	AF028823	Homo sapiens Tax interaction protein 1 mRNA, complete cds.
Mouse	chr11:72788074-72789597:-	1110037P24	1110001A14	hypothetical protein
Mouse	chr11:72788966-72794602:+	BU054686	1300011C24	TAX INTERACTION PROTEIN 1 (GLUTAMINASE-INTERACTING PROTEIN 3) (TAX-INTERACTING PROTEIN 1) homolog [Homo sapiens]
Human	chr17:4348882-4389392:+	BC065221	BC041772	Homo sapiens, Similar to spinster-like protein, clone IMAGE:4814561, mRNA, partial cds.
Human	chr17:4388940-4405428:-	AL133098	AF147709	Homo sapiens MYB-binding protein 1A (MYBBP1A) mRNA, complete cds.
Mouse	chr11:72064193-72102525:-	BC011467	F830003N21	Similar to spinster-like protein homolog [Mus musculus]
Mouse	chr11:72053881-72064325:+	D130058J15	D130058J15	MYB binding protein (P160) 1a
Human	chr17:5126284-5230384:+	BC041700	BC041700	Homo sapiens, rabaptin-5, clone MGC:48839 IMAGE:6046320, mRNA, complete cds.
Human	chr17:5205839-5264187:-	AA019366	Y08612	Homo sapiens mRNA for nuclear pore complex protein 88 (Nup88 gene).
Mouse	chr11:70457317-70556121:+	CD354728	6330437J23	rabaptin, RAB GTPase binding effector protein 1
Mouse	chr11:70555614-70582558:-	D030031G21	D030031G21	preimplantation protein 2
Human	chr17:5263924-5277280:+	BC004451	BC004451	Homo sapiens, Similar to RIKEN cDNA 2400006N03 gene, clone MGC:4189 IMAGE:2820882, mRNA, complete cds.
Human	chr17:5276673-5312979:-	CR607361	BC050409	Homo sapiens, clone IMAGE:6474118, mRNA.
Mouse	chr11:70582755-70590489:+	I6C0022E21	BC047996	Mus musculus RIKEN cDNA 2400006N03 gene, mRNA (cDNA clone MGC:59595 IMAGE:6509910), complete cds. CDS=295..954
Mouse	chr11:70590363-70595582:-	2410089D17	2410089D17	complement component 1, q subcomponent binding protein
Human	chr17:5330440-5334858:+	AK056072	AK056072	Homo sapiens cDNA FLJ31510 fis, clone NT2RI1000027.
Human	chr17:5243229-5330855:-	BI462164	BC009549	Homo sapiens, clone IMAGE:3897859, mRNA.
Mouse	chr11:70632144-70639934:+	2810002M02	2810002M02	hypothetical Mis12 containing protein
Mouse	chr11:70619527-70632397:-	I830043H18	D030056M07	hypothetical protein, MGC: 11613
Human	chr17:7095029-7103985:+	AL713741	BC002762	Homo sapiens cDNA clone MGC:3373 IMAGE:3629369, complete cds.
Human	chr17:7084181-7108026:-	CR594337	CR599503	full-length cDNA clone CS0DD008YP10 of Neuroblastoma Cot 50-normalized of Homo sapiens (human).
Mouse	chr11:69580780-69594299:-	I830080F04	I830080F04	retinoic acid induced 12
Mouse	chr11:69593330-69612567:+	3110001F03	BC018265	Mus musculus Dullard homolog (Xenopus laevis), mRNA (cDNA clone MGC:25664 IMAGE:4486464), complete cds. CDS=317..1051

Human	chr17:7225134-7233807:+	BC035782	U43408	Human tyrosine kinase (Tnk1) mRNA, complete cds.
Human	chr17:7233770-7248177:-	BC011735	AK055822	Homo sapiens cDNA FLJ31260 fis, clone KIDNE2005854, highly similar to Homo sapiens phospholipid scramblase 3 mRNA.
Mouse	chr11:69461141-69471330:-	4022441E23	4022441E23	tyrosine kinase, non-receptor, 1
Mouse	chr11:69458803-69464614:+	4732498B20	4732498B20	phospholipid scramblase 3
Human	chr17:7427571-7435344:+	AK027742	AK027742	Homo sapiens cDNA FLJ14836 fis, clone OVARC1001702.
Human	chr17:7432220-7434455:-	AB006867	AB006867	Homo sapiens mRNA for hSOX20 protein, complete cds.
Mouse	chr11:69269253-69275205:-	BC026776	A530086E16	mannose-P-dolichol utilization defect 1
Mouse	chr11:69267592-69269283:+	X98369	2410119O10	SRY-box containing gene 15
Human	chr17:7701789-7706331:+	AK057061	AK057061	Homo sapiens cDNA FLJ32499 fis, clone SKNSH2000347, weakly similar to CYTOCHROME B2 PRECURSOR (EC 1.1.2.3).
Human	chr17:7700724-7729330:-	BC051846	BC051846	Homo sapiens hypothetical protein MGC14151, mRNA (cDNA clone MGC:60160 IMAGE:4476019), complete cds.
Mouse	chr11:69003483-69008189:-	BG083615	-	-
Mouse	chr11:69008043-69009606:+	AA718836	1500034E06	hypothetical Small nuclear ribonucleoprotein (Sm protein) containing protein
Human	chr17:8032976-8033731:+	AF116704	AF116704	Homo sapiens PRO2472 mRNA, complete cds.
Human	chr17:8032377-8034250:-	BC009261	BC070272	Homo sapiens hypothetical protein FLJ20014, mRNA (cDNA clone MGC:88261 IMAGE:4672989), complete cds.
Mouse	chr11:68670274-68673039:-	9630042H03	9630042H03	hypothetical Arginine-rich region profile containing protein
Mouse	chr11:68672273-68674134:+	2310047M10	2310047M10	hypothetical protein
Human	chr17:8132540-8134134:+	AF168714	AF168714	Homo sapiens x 005 protein mRNA, complete cds.
Human	chr17:8131806-8139442:-	BC041597	BC041597	Homo sapiens, Similar to RIKEN cDNA 1810012H11 gene, clone IMAGE:5455996, mRNA.
Mouse	chr11:68585040-68587741:-	BC018290	BC018290	Mus musculus RIKEN cDNA 2400006H24 gene, mRNA (cDNA clone IMAGE:3499194), containing frame-shift errors.
Mouse	chr11:68580681-68586925:+	9530097O21	9530097O21	RAN guanine nucleotide release factor
Human	chr17:8279895-8330401:+	AL832648	AL832648	Homo sapiens mRNA; cDNA DKFZp451M0318 (from clone DKFZp451M0318).
Human	chr17:8318248-8474797:-	BC000280	M69181	Human nonmuscle myosin heavy chain-B (MYH10) mRNA, partial cds.
Mouse	chr11:68420700-68421553:-	BC028549	BC028549	Mus musculus, clone IMAGE:1380624, mRNA.
Mouse	chr11:68304115-68429188:+	AK122578	M5C1080G13	myosin heavy chain 10, non-muscle
Human	chr17:10227174-10381910:+	AI208901	-	-
Human	chr17:10336349-10500192:-	AF111784	BX510904	Homo sapiens mRNA; cDNA DKFZp451A123 (from clone DKFZp451A123); complete cds.
Mouse	chr11:66808228-66811782:-	9430073C21	9430073C21	myosin, heavy polypeptide 2, skeletal muscle, adult
Mouse	chr11:66691641-66921182:+	BC008538	BC008538	Mus musculus myosin, heavy polypeptide 2, skeletal muscle, adult, mRNA (cDNA clone MGC:7639 IMAGE:3495412), complete cds. CDS=65..4861
Human	chr17:12633581-12835686:+	AL080100	AB014572	Homo sapiens mRNA for KIAA0672 protein, complete cds.
Human	chr17:12835312-12862082:-	CA442985	AK001392	Homo sapiens cDNA FLJ10530 fis, clone NT2RP2000985.
Mouse	chr11:64614587-64775509:-	BC056366	BC056366	Mus musculus expressed sequence AU040829, mRNA (cDNA clone MGC:73418 IMAGE:6825221), complete cds. CDS=324..2618
Mouse	chr11:64591583-64614622:+	F830045F10	F830045F10	elaC homolog 2 (E. coli)
Human	chr17:15884511-15885318:+	BC031588	BC031588	Homo sapiens, clone IMAGE:5193340, mRNA.
Human	chr17:15873196-16059735:-	AL137641	AF044209	Homo sapiens nuclear receptor co-repressor N-CoR mRNA, complete cds.
Mouse	chr11:61931930-61935698:+	B230205G03	B230205G03	nuclear receptor co-repressor 1
Mouse	chr11:61929095-62151818:-	AB093281	U35312	Mus musculus nuclear receptor co-repressor mRNA, complete cds. CDS=117..7478
Human	chr17:15884511-15885318:+	BC031588	BC031588	Homo sapiens, clone IMAGE:5193340, mRNA.
Human	chr17:15885080-15885834:-	BX502596	-	-
Mouse	chr11:61931930-61935698:+	B230205G03	B230205G03	nuclear receptor co-repressor 1
Mouse	chr11:61929095-62151818:-	AB093281	U35312	Mus musculus nuclear receptor co-repressor mRNA, complete cds. CDS=117..7478
Human	chr17:16059689-16195843:+	BX441012	AB017165	Homo sapiens PIG-L mRNA, complete cds.
Human	chr17:15873196-16059735:-	AA426524	AF044209	Homo sapiens nuclear receptor co-repressor N-CoR mRNA, complete cds.
Mouse	chr11:62069971-62128585:+	BC052190	F930035M18	phosphatidylinositol glycan, class L
Mouse	chr11:61929095-62151818:-	I730022N15	U35312	Mus musculus nuclear receptor co-repressor mRNA, complete cds. CDS=117..7478
Human	chr17:17087242-17091977:+	CD171782	-	-
Human	chr17:17090661-17125342:-	CR590945	CR600698	full-length cDNA clone CS0DE006YH01 of Placenta of Homo sapiens (human).
Mouse	chr11:59422607-59431018:+	G830001I12	G830001I12	hypothetical protein
Mouse	chr11:59429941-59460754:-	E430018C16	AF071313	Mus musculus COP9 complex subunit 3 (COPS3) mRNA, complete cds. CDS=44..1315
Human	chr17:17525512-17655498:+	AL834468	AY172136	Homo sapiens retinoic acid induced 1 (RAI1) mRNA, complete cds.
Human	chr17:17655378-17681050:-	S66168	BC057388	Homo sapiens sterol regulatory element binding transcription factor 1, mRNA (cDNA clone MGC:54239 IMAGE:5786483), complete cds.
Mouse	chr11:59717493-59811737:+	F630012D23	BC049785	Mus musculus, clone IMAGE:6511305, mRNA, partial cds. CDS=1..639
Mouse	chr11:59811625-59835231:-	BC056922	D630008H06	sterol regulatory element binding factor 1

Human	chr17:18069626-18088914:+	BC051466	D50550	Human LLGL mRNA, complete cds.
Human	chr17:18088854-18102806:-	BC025300	BC025300	Homo sapiens, flightless I homolog (Drosophila), clone MGC:39265 IMAGE:4584634, mRNA, complete cds.
Mouse	chr11:60312230-60326728:+	D16141	BC055399	Mus musculus lethal giant larvae homolog, mRNA (cDNA clone MGC:62651 IMAGE:6308689), complete cds. CDS=139..3327
Mouse	chr11:60326643-60339805:-	BC032282	AF287264	Mus musculus cytoskeletal actin-modulating protein (Fliih) mRNA, complete cds. CDS=33..3848
Human	chr17:18168140-18172097:+	AK092035	AK092035	Homo sapiens cDNA FLJ34716 fis, clone MESAN2005284.
Human	chr17:18171904-18207602:-	BC007979	BC038598	Homo sapiens, serine hydroxymethyltransferase 1 (soluble), clone MGC:46169 IMAGE:4523709, mRNA, complete cds.
Mouse	chr11:60390065-60400827:+	2310076G09	C430032E20	Smith-Magenis syndrome chromosome region, candidate 8 homolog (human)
Mouse	chr11:60400643-60424258:-	BC026055	4022425K22	serine hydroxymethyl transferase 1 (soluble)
Human	chr17:18794714-18864729:+	BC034380	BC039868	Homo sapiens, clone MGC:49034 IMAGE:4157703, mRNA, complete cds.
Human	chr17:18812827-18848842:-	BX362821	-	-
Mouse	chr11:61285322-61333339:-	F530201E06	F530201E06	Na+/glucose cotransporter-related protein homolog [Oryctolagus cuniculus]
Mouse	chr11:61296607-61322490:+	4732401E24	4732401E24	hypothetical Phospholipase D/nuclease structure containing protein
Human	chr17:19081302-19180621:+	AK001491	AB028988	Homo sapiens mRNA for KIAA1065 protein, complete cds.
Human	chr17:19178605-19180791:-	AL137568	AL137568	Homo sapiens mRNA; cDNA DKFZp586J2021 (from clone DKFZp586J2021).
Mouse	chr11:61129789-61192227:-	BC039138	3221401G07	epsin 2
Mouse	chr11:61127698-61131146:+	9330177E19	9330177E19	unclassifiable
Human	chr17:19221659-19227450:+	BC030134	BC007992	Homo sapiens mitogen-activated protein kinase 7, transcript variant 2, mRNA (cDNA clone MGC:3875 IMAGE:3009873), complete cds.
Human	chr17:19227348-19231146:-	BC035560	CR598399	full-length cDNA clone CS0DE014YN13 of Placenta of Homo sapiens (human).
Mouse	chr11:61101352-61106946:-	BC033598	BC033598	Mus musculus mitogen-activated protein kinase 7, mRNA (cDNA clone IMAGE:4507550), with apparent retained intron.
Mouse	chr11:61097971-61101439:+	1110053C02	1110053C02	MICROFIBRIL-ASSOCIATED GLYCOPROTEIN 4 homolog [Homo sapiens]
Human	chr17:23670288-23680230:+	BM998924	L19183	Human MAC30 mRNA, 3' end.
Human	chr17:23679478-23686642:-	BC002640	BC002640	Homo sapiens intraflagellar transport protein IFT20, mRNA (cDNA clone MGC:4279 IMAGE:3609552), complete cds.
Mouse	chr11:78154069-78163333:-	BQ177669	F630201P20	similar to MAC30 protein (Fragment) [Homo sapiens]
Mouse	chr11:78148917-78154288:+	BE990547	-	-
Human	chr17:23750229-23752162:+	BF339050	-	-
Human	chr17:23745787-23758342:-	AL832613	AL832613	Homo sapiens mRNA; cDNA DKFZp451K1917 (from clone DKFZp451K1917).
Mouse	chr11:78082782-78084615:-	CB845273	-	-
Mouse	chr11:78078261-78084501:+	F630106D15	BC057976	Mus musculus DNA segment, Chr 11, ERATO Doi 18, expressed, mRNA (cDNA clone MGC:68046 IMAGE:5052851), complete cds. CDS=63..1442
Human	chr17:23949935-23968524:+	AK098638	BC001556	Homo sapiens, clone IMAGE:3464359, mRNA.
Human	chr17:23958444-23965338:-	AK057735	AK057735	Homo sapiens cDNA FLJ25006 fis, clone CBL00989.
Mouse	chr11:77905401-77914068:-	4930549P06	4930549P06	unclassifiable
Mouse	chr11:77903397-77909361:+	BC030499	A230108M20	hypothetical protein
Human	chr17:24077069-24093671:+	BF982335	AY267371	Homo sapiens NIMA-family kinase NEK8 (NEK8) mRNA, complete cds.
Human	chr17:24075493-24079080:-	BC014072	BC014072	Homo sapiens hypothetical protein BC014072, mRNA (cDNA clone MGC:20499 IMAGE:3957355), complete cds.
Mouse	chr11:77789813-77791747:-	A730022B19	A730022B19	unclassifiable
Mouse	chr11:77789267-77795098:+	B430107C01	B430107C01	hypothetical TRAM, LAG1 and CLN8 homology containing protein
Human	chr17:24609741-24610425:+	AV650032	-	-
Human	chr17:24606980-24645292:-	AK001838	AB037742	Homo sapiens mRNA for KIAA1321 protein, partial cds.
Mouse	chr11:77327766-77337849:-	CA876857	AJ239052	Mus musculus mRNA for beta-A3/A1 crystallin protein (Cryba1 gene). CDS=13..660
Mouse	chr11:77326821-77330522:+	9530056D24	9530056D24	unclassifiable
Human	chr17:24917270-24924308:+	BI909891	CR612916	full-length cDNA clone CS0DI063YG22 of Placenta Cot 25-normalized of Homo sapiens (human).
Human	chr17:24910946-24918174:-	AY358212	AY358212	Homo sapiens clone DNA170281 PPWG6510 (UNQ6510) mRNA, complete cds.
Mouse	chr11:77120655-77128536:-	I730035O16	2410019G02	hypothetical protein
Mouse	chr11:77127673-77151161:+	B430113H14	A430072G18	hypothetical protein
Human	chr17:30358466-30360732:+	CR591264	CR591264	full-length cDNA clone CS0DC023YK19 of Neuroblastoma Cot 25-normalized of Homo sapiens (human).
Human	chr17:30360244-30472625:-	BC028424	AK097811	Homo sapiens cDNA FLJ40492 fis, clone TESTI2044309, highly similar to DNA REPAIR PROTEIN RAD51.
Mouse	chr11:82394441-82420746:+	9930109I05	BC049240	Mus musculus ligase III, DNA, ATP-dependent, mRNA (cDNA clone MGC:54652 IMAGE:4504384), complete cds. CDS=76..3114
Mouse	chr11:82417147-82484542:-	AK128983	F630006N07	RING finger protein SAKURA (Fragment) homolog [Rattus norvegicus]
Human	chr17:31916585-31929267:+	AK096494	AK096494	Homo sapiens cDNA FLJ39175 fis, clone OCBBF2003593, highly similar to BETA-SOLUBLE NSF ATTACHMENT PROTEIN.
Human	chr17:31925590-31974843:-	AL133017	BC008900	Homo sapiens hypothetical protein FLJ22865, mRNA (cDNA clone MGC:14882 IMAGE:3629870), complete cds.
Mouse	chr11:84512612-84518047:-	1110055A02	1110055A02	myosin head domain containing 1
Mouse	chr11:84481813-84512891:+	9130011J09	BC060115	Mus musculus RIKEN cDNA 1110055A02 gene, mRNA (cDNA clone IMAGE:6405702), partial cds.

Human	chr17:35472167-35503646:+	BC035137	BC035137	Homo sapiens, similar to thyroid hormone receptor, clone MGC:43240 IMAGE:5264048, mRNA, complete cds.
Human	chr17:35499007-35510508:-	M24900	M24898	Human triiodothyronine recptor (THRA1, ear1) mRNA, complete cds.
Mouse	chr11:98411759-98444525:+	D930024E14	BC046795	Mus musculus thyroid hormone receptor alpha, mRNA (cDNA clone MGC:61415 IMAGE:5717946), complete cds. CDS=403..1881
Mouse	chr11:98439054-98446500:-	9830147D03	I730089C19	nuclear receptor subfamily 1, group D, member 1
Human	chr17:36228870-36246048:+	CD689247	BC015365	Homo sapiens, clone MGC:21518 IMAGE:3900532, mRNA, complete cds.
Human	chr17:36227850-36232383:-	BC034697	BC034697	Homo sapiens, keratin 10 (epidermolytic hyperkeratosis; keratosis palmaris et plantaris), clone MGC:21369 IMAGE:4751594, mRNA, complete cds.
Mouse	chr11:99057330-99060827:+	BQ551984	-	-
Mouse	chr11:99056377-99060487:-	C130014E24	C130014E24	KERATIN, TYPE I CYTOSKELETAL 10 (CYTOKERATIN 10) (56 KDA CYTOKERATIN) (KERATIN, TYPE I CYTOSKELETAL 59 KDA) homolog [Mus musculus]
Human	chr17:37372285-37383786:+	AK124861	BC028040	Homo sapiens, 2',3'-cyclic nucleotide 3' phosphodiesterase, clone MGC:40095 IMAGE:5248370, mRNA, complete cds.
Human	chr17:37381965-37426893:-	BC003601	BX647209	Homo sapiens mRNA; cDNA DKFZp686I18256 (from clone DKFZp686I18256).
Mouse	chr11:100246027-100262852:+	A330007H09	A330007H09	cyclic nucleotide phosphodiesterase 1
Mouse	chr11:100253935-100291303:-	I1C0036L01	BC055729	Mus musculus DnaJ (Hsp40) homolog, subfamily C, member 7, mRNA (cDNA clone MGC:66940 IMAGE:6414499), complete cds. CDS=564..2048
Human	chr17:37422145-37431188:+	BC047369	BC063498	Homo sapiens I-kappa-B-interacting Ras-like protein 2, mRNA (cDNA clone MGC:74742 IMAGE:5248950), complete cds.
Human	chr17:37381965-37426893:-	BX647209	BX647209	Homo sapiens mRNA; cDNA DKFZp686I18256 (from clone DKFZp686I18256).
Mouse	chr11:100290299-100298734:+	9330102C03	4930527H08	KAPPA B-RAS 2 (I-KAPPA-B-INTERACTING RAS-LIKE PROTEIN 2) homolog [Homo sapiens]
Mouse	chr11:100253935-100291303:-	BC055729	BC055729	Mus musculus DnaJ (Hsp40) homolog, subfamily C, member 7, mRNA (cDNA clone MGC:66940 IMAGE:6414499), complete cds. CDS=564..2048
Human	chr17:37422145-37431188:+	AL137682	BC063498	Homo sapiens I-kappa-B-interacting Ras-like protein 2, mRNA (cDNA clone MGC:74742 IMAGE:5248950), complete cds.
Human	chr17:37431120-37434233:-	BC067901	BC080613	Homo sapiens cDNA clone MGC:99757 IMAGE:5922386, complete cds.
Mouse	chr11:100290299-100298734:+	D030024I01	4930527H08	KAPPA B-RAS 2 (I-KAPPA-B-INTERACTING RAS-LIKE PROTEIN 2) homolog [Homo sapiens]
Mouse	chr11:100298666-100363578:-	A930006D11	A930006D11	hypothetical protein
Human	chr17:37972604-37979480:+	AF213668	AF213668	Homo sapiens bHLHZip transcription factor BIGMAX gamma mRNA, complete cds.
Human	chr17:37977854-37983356:-	AK126369	AB030304	Homo sapiens mRNA for TBP-1 interacting protein, complete cds.
Mouse	chr11:100757833-100763330:+	U43548	2610020C01	transcription factor-like 4
Mouse	chr11:100763264-100766559:-	4933440A22	4933440A22	proteasome (prosome, macropain) 26S subunit, ATPase 3, interacting protein
Human	chr17:38204336-38217130:+	AL833052	AK097456	Homo sapiens cDNA FLJ40137 fis, clone TESTI2012776.
Human	chr17:38215623-38229834:-	BX647788	BX647788	Homo sapiens mRNA; cDNA DKFZp686I1686 (from clone DKFZp686I1686).
Mouse	chr11:100950135-100967440:+	4921513J16	4921513J16	unclassifiable
Mouse	chr11:100957075-100973409:-	6430407H14	6430407H14	beclin 1 (coiled-coil, myosin-like BCL2-interacting protein)
Human	chr17:38385649-38399235:+	BU189319	BC039247	Homo sapiens, clone MGC:33375 IMAGE:5271271, mRNA, complete cds.
Human	chr17:38356069-38386422:-	CR619011	CR619011	full-length cDNA clone CS0DF033YE07 of Fetal brain of Homo sapiens (human).
Mouse	chr11:101096208-101106796:+	D930024B17	F830205G14	Hypothetical RUN domain containing protein
Mouse	chr11:101089935-101096451:-	1700113I22	1700113I22	hypothetical HSP20-like chaperones structure containing protein
Human	chr17:39619877-39624823:+	BM999307	BC063489	Homo sapiens hypothetical protein MGC3123, mRNA (cDNA clone MGC:74919 IMAGE:5239969), complete cds.
Human	chr17:39624699-39632746:-	BC064932	BC037418	Homo sapiens, clone IMAGE:5499446, mRNA, partial cds.
Mouse	chr11:101956054-101960550:+	3110065C03	3110065C03	hypothetical Ubiquitin/Ubiquitin domain profile containing protein
Mouse	chr11:101960381-101968114:-	BC059880	BC059880	Mus musculus hypothetical protein E030022H21, mRNA (cDNA clone IMAGE:6813288), partial cds.
Human	chr17:39741307-39751565:+	AL133657	AL133657	Homo sapiens mRNA; cDNA DKFZp434A1727 (from clone DKFZp434A1727); partial cds.
Human	chr17:39736029-39748340:-	BM835539	-	-
Mouse	chr11:102064526-102073678:+	U73941	U73941	Mus musculus Rap2 interacting protein 8 (RPIP8) mRNA, complete cds. CDS=1..1341
Mouse	chr11:102070014-102070862:-	1700086L13	1700086L13	unclassifiable
Human	chr17:40654946-40680470:+	AF432213	AY278319	Homo sapiens leukocyte formin mRNA, complete cds.
Human	chr17:40671166-40674884:-	AK096807	AK096807	Homo sapiens cDNA FLJ39488 fis, clone PROST2015332.
Mouse	chr11:102841925-102870026:+	AF215666	A530030J22	formin-like 1
Mouse	chr11:102861770-102864175:-	BF469726	-	-
Human	chr17:40654946-40680470:+	AJ008112	AY278319	Homo sapiens leukocyte formin mRNA, complete cds.
Human	chr17:40680435-40680993:-	BU587659	-	-
Mouse	chr11:102841925-102870026:+	A530030J22	A530030J22	formin-like 1
Mouse	chr11:102869965-102879673:-	4930503A01	1700023F06	hypothetical protein
Human	chr17:43976715-44038772:+	BE676309	X84838	H.sapiens B1 mRNA for mucin-like antigen.
Human	chr17:43981231-44043378:-	U59298	X16667	Human HOX2G mRNA from the Hox2 locus.
Mouse	chr11:96014151-96024419:-	AW321046	-	-
Mouse	chr11:95994353-96019046:+	X66177	X66177	M.musculus mRNA for Hox 2.7 protein. CDS=481..1782

Human	chr17:43976715-44038772:+	CB046508	X84838	H.sapiens B1 mRNA for mucin-like antigen.
Human	chr17:44023618-44026322:-	M92299	M92299	Human homeobox 2.1 protein (HOX2A) mRNA, complete cds.
Mouse	chr11:95962146-95978033:-	W16155	0610040B09	unclassifiable
Mouse	chr11:95973718-95977245:+	BC040755	BC040755	Mus musculus homeo box B5, mRNA (cDNA clone IMAGE:4221781), with apparent retained intron.
Human	chr17:44340768-44361892:+	AK091644	AK130303	Homo sapiens cDNA FLJ26793 fis, clone PRS04911.
Human	chr17:44360571-44361417:-	BU674319	-	-
Mouse	chr11:95717475-95736511:-	5830473M12	5830473M12	hypothetical Ubiquitin-conjugating enzymes containing protein
Mouse	chr11:95719613-95719964:+	AW123579	-	-
Human	chr17:45805589-45813819:+	BC016470	AK055926	Homo sapiens cDNA FLJ31364 fis, clone NB9N41000121.
Human	chr17:45813484-45829913:-	AF119857	AK025328	Homo sapiens cDNA: FLJ21675 fis, clone COL09090, highly similar to AF119857 Homo sapiens PRO1855 mRNA.
Mouse	chr11:94316095-94325087:-	6820428D13	6820428D13	weakly similar to CDNA FLJ31364 FIS, CLONE NB9N41000121 [Homo sapiens]
Mouse	chr11:94300870-94316969:+	I920098M07	I420018N16	P34 protein homolog [Rattus sp]
Human	chr17:50333051-50396349:+	AJ010071	AK001893	Homo sapiens cDNA FLJ11031 fis, clone PLACE1004183, highly similar to Homo sapiens for TOM1-like protein.
Human	chr17:50351807-50401145:-	AF044321	AF044321	Homo sapiens cytochrome c oxidase assembly protein COX11 (COX11) mRNA, complete cds.
Mouse	chr11:90314582-90358730:-	D030056H14	D030056H14	ADAPTOR MOLECULE SRCASM
Mouse	chr11:90309265-90317103:+	F530002N02	F530002N02	similar to Cytochrome c oxidase assembly protein COX11, mitochondrial precursor [Homo sapiens]
Human	chr17:53439049-53440427:+	CR616222	CR616222	full-length cDNA clone CS0DB005YP24 of Neuroblastoma Cot 10-normalized of Homo sapiens (human).
Human	chr17:53421398-53439655:-	M69040	AK126318	Homo sapiens cDNA FLJ44342 fis, clone TRACH3005294.
Mouse	chr11:87613656-87649976:-	BE863191	-	-
Mouse	chr11:87648987-87669141:+	I830011H17	A430082O03	unclassifiable
Human	chr17:54538632-55274398:+	BP309323	AX775965	Sequence 235 from Patent WO03048202.
Human	chr17:55106779-55139769:-	BG778161	AF151905	Homo sapiens CGI-147 protein mRNA, complete cds.
Mouse	chr11:86184989-86285665:-	K1C0001L09	A730054P13	VACUOLE MEMBRANE PROTEIN 1 homolog [Rattus norvegicus]
Mouse	chr11:86285542-86294122:+	CF739368	6030462P20	similar to HYPOTHETICAL PROTEIN CGI-147 [Homo sapiens]
Human	chr17:55511157-55520838:+	BG548248	-	-
Human	chr17:55475325-55511218:-	AF349752	AF349752	Homo sapiens amplified in breast cancer 1 (ABC1) mRNA, complete cds.
Mouse	chr11:83354754-83355624:-	A930101I12	-	-
Mouse	chr11:83355134-83388505:+	2700008B19	4732466K05	hypothetical ARM repeat fold containing protein
Human	chr17:58137299-58169015:+	BC041872	BC041872	Homo sapiens, clone IMAGE:5271830, mRNA.
Human	chr17:58132407-58239437:-	AK122947	BX648705	Homo sapiens mRNA; cDNA DKFZp686P0339 (from clone DKFZp686P0339).
Mouse	chr11:105034377-105061767:+	A230096M02	A230096M02	unclassifiable
Mouse	chr11:105031923-105127860:-	4933417C16	4922503O18	hypothetical Proline-rich region profile containing protein
Human	chr17:59031963-59039457:+	AK094052	AK094052	Homo sapiens cDNA FLJ36733 fis, clone UTERU2012856, weakly similar to 26.4 KDA PROTEIN IN RUVC-ASPS INTERGENIC REGION.
Human	chr17:59032079-59039455:-	AW448977	-	-
Mouse	chr11:105737182-105745160:+	9330140N01	B230313O11	UPF0082 protein
Mouse	chr11:105736202-105737969:-	5330430P22	5330430P22	RIKEN cDNA 2310066I18 gene
Human	chr17:59053517-59127402:+	BC010464	U78876	Human MEK kinase 3 mRNA, complete cds.
Human	chr17:59126981-59132264:-	AX747464	AX747464	Sequence 989 from Patent EP1308459.
Mouse	chr11:105755712-105827781:+	D330027O12	9430093B06	mitogen activated protein kinase kinase kinase 3
Mouse	chr11:105827379-105832206:-	6430544F17	6430544F17	hypothetical LIM domain/LIM domain profile/Cytochrome c family heme-binding site containing protein
Human	chr17:59131356-59133777:+	BI550182	CR594811	full-length cDNA clone CS0DL006YD08 of B cells (Ramos cell line) Cot 25-normalized of Homo sapiens (human).
Human	chr17:59126981-59132264:-	BC051812	AX747464	Sequence 989 from Patent EP1308459.
Mouse	chr11:105831165-105832282:+	1700041A03	1700041A03	hypothetical protein
Mouse	chr11:105827379-105832206:-	F830011H16	6430544F17	hypothetical LIM domain/LIM domain profile/Cytochrome c family heme-binding site containing protein
Human	chr17:59933316-59964531:+	AK057326	BC009518	Homo sapiens, clone MGC:10965 IMAGE:3633884, mRNA, complete cds.
Human	chr17:59924832-59934097:-	AL705375	X52104	Human mRNA for p68 protein.
Mouse	chr11:106459765-106493159:+	D330027A14	4732496G21	hypothetical Calponin-homology domain, CH-domain structure containing protein
Mouse	chr11:106451478-106460310:-	I1C0003N03	G730030M03	DEAD (Asp-Glu-Ala-Asp) box polypeptide 5
Human	chr17:62671394-62688658:+	BI462408	-	-
Human	chr17:62594047-62672567:-	BP353830	BC056895	Homo sapiens helicase with zinc finger domain, mRNA (cDNA clone IMAGE:6144860), partial cds.
Mouse	chr11:107202882-107219498:-	A830035A12	A830035A12	similar to EMBRYONAL STEM CELL SPECIFIC GENE 1 [Mus musculus]
Mouse	chr11:107219055-107358068:+	2810017C19	BC060114	Mus musculus RIKEN cDNA 9630002H22 gene, mRNA (cDNA clone MGC:65693 IMAGE:6405580), complete cds. CDS=246..1286

Human	chr17:68695686-68716318:+	AL359611	BC047465	Homo sapiens, clone IMAGE:5285077, mRNA.
Human	chr17:68715087-68740074:-	AK074755	BX648968	Homo sapiens mRNA; cDNA DKFZp686G2260 (from clone DKFZp686G2260).
Mouse	chr11:113320298-113338183:+	6030445I15	K230004A13	component of oligomeric golgi complex 1
Mouse	chr11:113332448-113355284:-	2310042P03	E130315N09	hypothetical Serine-rich region containing protein
Human	chr17:68716729-68717429:+	CN358253	-	-
Human	chr17:68715087-68740074:-	AK074755	BX648968	Homo sapiens mRNA; cDNA DKFZp686G2260 (from clone DKFZp686G2260).
Mouse	chr11:113320298-113338183:+	6030445I15	K230004A13	component of oligomeric golgi complex 1
Mouse	chr11:113332448-113355284:-	2310042P03	E130315N09	hypothetical Serine-rich region containing protein
Human	chr17:70617625-70637955:+	BC011728	BC011728	Homo sapiens, hypothetical protein FLJ22160, clone MGC:19466 IMAGE:4423125, mRNA, complete cds.
Human	chr17:70637915-70639533:-	AF147311	AK000419	Homo sapiens cDNA FLJ20412 fis, clone KAT02154.
Mouse	chr11:115146796-115161599:+	BC023126	BC023126	Mus musculus cDNA sequence BC023126, mRNA (cDNA clone MGC:27721 IMAGE:2616092), complete cds. CDS=261..857
Mouse	chr11:115161541-115162991:-	BC024368	1810010F06	5' nucleotidase, deoxy (pyrimidine), cytosolic type C
Human	chr17:70638573-70640733:+	AK094291	AK094291	Homo sapiens cDNA FLJ36972 fis, clone BRACE2006245.
Human	chr17:70637915-70639533:-	AK000419	AK000419	Homo sapiens cDNA FLJ20412 fis, clone KAT02154.
Mouse	chr11:115162658-115164490:+	C530044P15	C530044P15	unclassifiable
Mouse	chr11:115161541-115162991:-	1810010F06	1810010F06	5' nucleotidase, deoxy (pyrimidine), cytosolic type C
Human	chr17:70769344-70774463:+	CR626245	CR600124	full-length cDNA clone CS0DK003YH22 of HeLa cells Cot 25-normalized of Homo sapiens (human).
Human	chr17:70744282-70769480:-	BG771920	BC063285	Homo sapiens golgi associated, gamma adaptin ear containing, ARF binding protein 3, mRNA (cDNA clone IMAGE:4821620), partial cds.
Mouse	chr11:114762560-115279165:+	2410008G21	B230354I04	unclassifiable
Mouse	chr11:115255382-115275651:-	BI329674	AK122212	Mus musculus mRNA for mKIAA0154 protein. CDS=1..2175
Human	chr17:70769344-70774463:+	BC000241	CR600124	full-length cDNA clone CS0DK003YH22 of HeLa cells Cot 25-normalized of Homo sapiens (human).
Human	chr17:70773904-70778909:-	AF225422	AF225422	Homo sapiens AD023 mRNA, complete cds.
Mouse	chr11:114762560-115279165:+	6820413L12	B230354I04	unclassifiable
Mouse	chr11:115279047-115284098:-	BC026745	BC026740	Mus musculus RIKEN cDNA 2310075G12 gene, mRNA (cDNA clone MGC:30339 IMAGE:4015105), complete cds. CDS=196..864
Human	chr17:70780296-70782363:+	BC021162	BC021162	Homo sapiens solute carrier family 25 (mitochondrial deoxynucleotide carrier), member 19, mRNA (cDNA clone IMAGE:3627625), partial cds.
Human	chr17:70780656-70805624:-	AL833387	AK075239	Homo sapiens cDNA FLJ90758 fis, clone SKNMC1000082, weakly similar to BRITTLE-1 PROTEIN PRECURSOR.
Mouse	chr11:115286591-115297836:+	7120478D08	7120478D08	unclassifiable
Mouse	chr11:115285285-115299426:-	F830220L17	2900089E13	solute carrier family 25 (mitochondrial deoxynucleotide carrier), member 19
Human	chr17:70964263-71008128:+	M62218	BC042942	Homo sapiens, Similar to KIAA0195 gene product, clone IMAGE:5295538, mRNA.
Human	chr17:71007936-71023222:-	AK025262	BC066643	Homo sapiens CASK interacting protein 2, mRNA (cDNA clone MGC:70409 IMAGE:5576059), complete cds.
Mouse	chr11:115436561-115470496:+	C030003A12	BC053088	Mus musculus RIKEN cDNA 2310067B10 gene, mRNA (cDNA clone MGC:62470 IMAGE:5709104), complete cds. CDS=143..4225
Mouse	chr11:115470111-115484825:-	9930124C21	AK129297	Mus musculus mRNA for mKIAA1139 protein. CDS=451..4125
Human	chr17:71174791-71215737:+	AF450482	AK095265	Homo sapiens cDNA FLJ37946 fis, clone CTONG2008792, highly similar to TRANSCRIPTIONAL REGULATOR PROTEIN HCNGP.
Human	chr17:71134520-71174864:-	AB042825	AB042824	Homo sapiens RECQL5 beta mRNA for DNA helicase recQ5 beta, complete cds.
Mouse	chr11:115604411-115638588:+	CA465692	BC021757	Mus musculus RIKEN cDNA 2700016D05 gene, mRNA (cDNA clone MGC:30549 IMAGE:5065414), complete cds. CDS=18..944
Mouse	chr11:115563722-115604621:-	4732486K21	BC059000	Mus musculus cDNA clone IMAGE:6848109, partial cds.
Human	chr17:71486896-71513675:+	BC047782	BX647274	Homo sapiens mRNA; cDNA DKFZp686C0493 (from clone DKFZp686C0493).
Human	chr17:71449183-71487090:-	BC008767	BC008767	Homo sapiens acyl-Coenzyme A oxidase 1, palmitoyl, transcript variant 1, mRNA (cDNA clone MGC:1198 IMAGE:3051501), complete cds.
Mouse	chr11:115869984-115886449:+	I730043L06	I730043L06	hypothetical protein
Mouse	chr11:115843012-115870179:-	BC056448	BC056448	Mus musculus cDNA clone MGC:66986 IMAGE:5704873, complete cds. CDS=342..2327
Human	chr17:72243542-72290878:+	AF015188	BC062563	Homo sapiens hypothetical protein ET, mRNA (cDNA clone MGC:74917 IMAGE:5140496), complete cds.
Human	chr17:72241790-72245602:-	AK124792	AK124792	Homo sapiens cDNA FLJ42802 fis, clone BRCAN2002562, moderately similar to Splicing factor, arginine/serine-rich 2.
Mouse	chr11:116514073-116546473:+	BG087609	6330559M05	ET PUTATIVE TRANSLATION PRODUCT (FRAGMENT) homolog [Mus musculus]
Mouse	chr11:116521028-116524249:-	D930005P03	D930005P03	splicing factor, arginine/serine-rich 2 (SC-35)
Human	chr17:73886323-73932854:+	AK024529	AK024529	Homo sapiens cDNA: FLJ20876 fis, clone ADKA02905.
Human	chr17:73931373-73980451:-	AK126059	AK128065	Homo sapiens cDNA FLJ46185 fis, clone TESTI4004917, moderately similar to Dynein beta chain, ciliary.
Mouse	chr11:117657986-117695141:+	C330016K12	C330027M21	PHOSPHATIDYLGLYCEROPHOSPHATE SYNTHASE homolog [Cricetulus griseus]
Mouse	chr11:117690216-117698570:-	BM938048	-	-
Human	chr17:75758428-75797725:+	AF322642	AK091123	Homo sapiens cDNA FLJ33804 fis, clone CTONG2000766, weakly similar to Rattus norvegicus caspase recruitment domain protein 9 mRNA.
Human	chr17:75795110-75808768:-	BX644172	AK095969	Homo sapiens cDNA FLJ38650 fis, clone HHDPC2007775, highly similar to N-SULPHOGLUCOSAMINE SULPHOHYDROLASE PRECURSOR (EC 3.10.1.1).
Mouse	chr11:118985635-119016547:+	BC029102	BC029102	Mus musculus caspase recruitment domain family, member 14, mRNA (cDNA clone MGC:28122 IMAGE:3979883), complete cds. CDS=325..3324
Mouse	chr11:119014554-119026667:-	9330164L14	9330164L14	N-sulfoglucosamine sulfohydrolase (sulfamidase)

Human	chr17:75935329-75984681:+	BX647946	BX647946	Homo sapiens mRNA; cDNA DKFZp686C2251 (from clone DKFZp686C2251).
Human	chr17:75940228-76003513:-	AK125341	AK125341	Homo sapiens cDNA FLJ43351 fis, clone NT2RP7005846.
Mouse	chr11:119118385-119120992:+	AI527084	-	-
Mouse	chr11:119119917-119162464:-	AA118431	A630033C17	unclassifiable
Human	chr17:77602289-77609226:+	BF002881	U20285	Human Gps1 (GPS1) mRNA, complete cds.
Human	chr17:77609037-77617052:-	BC062566	CR605112	full-length cDNA clone CS0DI018YL23 of Placenta Cot 25-normalized of Homo sapiens (human).
Mouse	chr11:120455399-120460412:+	BC003350	E430018N19	fusca protein homolog Gps1 homolog [Homo sapiens]
Mouse	chr11:120458559-120467533:-	C330024N15	E430007O22	hypothetical Uncharacterized protein family UPF0034 containing protein
Human	chr17:77779571-77812325:+	AK127319	CR620431	full-length cDNA clone CS0DK001YL04 of HeLa cells Cot 25-normalized of Homo sapiens (human).
Human	chr17:77790184-77824873:-	CR618512	CR593622	full-length cDNA clone CS0DI007YD13 of Placenta Cot 25-normalized of Homo sapiens (human).
Mouse	chr11:120619609-120631997:+	G730030P11	G730030P11	solute carrier family 16 (monocarboxylic acid transporters), member 3
Mouse	chr11:120629591-120662465:-	B430105I09	B430105I09	casein kinase 1, delta
Human	chr17:77969838-77993810:+	AK074405	CR607308	full-length cDNA clone CS0DL011YN22 of B cells (Ramos cell line) Cot 25-normalized of Homo sapiens (human).
Human	chr17:77993751-78001994:-	AK027743	AK054876	Homo sapiens cDNA FLJ30314 fis, clone BRACE2003527.
Mouse	chr11:120875562-120893798:+	F630011I03	F630011I03	hypothetical Glycoside hydrolase, family 20 containing protein
Mouse	chr11:120893717-120900451:-	BC026616	I530011L03	hypothetical protein
Human	chr17:78303219-78496019:+	BC012824	BC003094	Homo sapiens, tubulin-specific chaperone d, clone MGC:1538 IMAGE:3503192, mRNA, complete cds.
Human	chr17:78493314-78602975:-	AK054757	AY634364	Homo sapiens UDP-GlcNAc:betaGal beta-1,3-N-acetylglucosaminyltransferase-like 1 (B3GNTL1) mRNA, complete cds.
Mouse	chr11:121123076-121292649:+	A630012G03	C730026G13	similar to TUBULIN-SPECIFIC CHAPERONE D [Homo sapiens]
Mouse	chr11:121287328-121344282:-	BC062106	F830016F21	Hypothetical glycosyl transferase
Human	chr18:148477-204629:+	AK098497	U30888	Human tRNA-guanine transglycosylase mRNA, complete cds.
Human	chr18:204247-258209:-	AK055354	BC010381	Homo sapiens, nuclear matrix protein p84, clone MGC:13557 IMAGE:4046908, mRNA, complete cds.
Mouse	chr18:10215058-10252155:-	F630210K22	F630210K22	ubiquitin specific protease 14
Mouse	chr18:10179904-10217480:+	D730043H17	I530009K01	THO complex 1
Human	chr18:9465007-9528112:+	L42542	L42542	Human RLIP76 protein mRNA, complete cds.
Human	chr18:9463419-9465215:-	BI461584	-	-
Mouse	chr17:64211852-64248289:-	I0C0031I05	I0C0031I05	ralA binding protein 1
Mouse	chr17:64247921-64250097:+	B930024M16	B930024M16	unclassifiable
Human	chr18:11872707-11875684:+	BQ127976	-	-
Human	chr18:11873474-11898779:-	AL833636	AL833636	Homo sapiens mRNA; cDNA DKFZp686K2379 (from clone DKFZp686K2379).
Mouse	chr18:67750703-67752030:+	AA672243	-	-
Mouse	chr18:67750286-67771068:-	F630013G06	A530095G11	hypothetical Hemopexin domain/Serine/threonine specific protein phosphatase containing protein
Human	chr18:19337432-19365751:+	BC008305	AK057192	Homo sapiens cDNA FLJ32630 fis, clone SYNOV1000128, highly similar to Homo sapiens colon cancer-associated protein Mic1 (MIC1) mRNA.
Human	chr18:19340146-19420468:-	AF002020	AF002020	Homo sapiens Niemann-Pick C disease protein (NPC1) mRNA, complete cds.
Mouse	chr18:12534432-12555725:+	3110002H16	G730012O10	Similar to colon cancer-associated protein Mic1 homolog [Homo sapiens]
Mouse	chr18:12555421-12602132:-	1110027K07	AF003348	Mus musculus NPC1 (Npc1) mRNA, complete cds. CDS=124..3960
Human	chr18:19972940-19995565:+	BC011996	BC011996	Homo sapiens, clone MGC:9117 IMAGE:3859043, mRNA, complete cds.
Human	chr18:19993474-20231821:-	AL040447	AF323726	Homo sapiens OSBP-related protein 1 mRNA, complete cds.
Mouse	chr18:13106488-13120309:+	AF359382	4922505I06	calcium-binding tyrosine-(Y)-phosphorylation regulated (fibrousheathin 2)
Mouse	chr18:13119062-13307701:-	AI414535	4921521L15	oxysterol binding protein-like 1A
Human	chr18:30056927-30060391:+	AW161256	-	-
Human	chr18:29685062-30057214:-	BI118180	BT006763	Homo sapiens nucleolar protein 4 mRNA, complete cds.
Mouse	chr18:23449528-23453067:+	G630062B06	G630062B06	hypothetical protein
Mouse	chr18:23099934-23452419:-	5830487I06	5830487I06	similar to NOLP PROTEIN (HRIHFB2255 PROTEIN) [Homo sapiens]
Human	chr18:30056927-30060391:+	AW161256	-	-
Human	chr18:30057294-30058907:-	BI460399	-	-
Mouse	chr18:23449528-23453067:+	G630062B06	G630062B06	hypothetical protein
Mouse	chr18:23099934-23452419:-	5830487I06	5830487I06	similar to NOLP PROTEIN (HRIHFB2255 PROTEIN) [Homo sapiens]
Human	chr18:50049772-50101634:+	AF140501	AF140501	Homo sapiens DNA polymerase iota (POLI) mRNA, complete cds.
Human	chr18:50048714-50050396:-	CR609953	CR609953	full-length cDNA clone CS0DI029YH23 of Placenta Cot 25-normalized of Homo sapiens (human).
Mouse	chr18:71036578-71058516:-	CN835082	AF151691	Mus musculus DNA polymerase iota (PolI) mRNA, complete cds. CDS=95..2248
Mouse	chr18:71058269-71060121:+	D630022O08	A430085C19	unclassifiable

Human	chr19:458497-470654:+	BC009520	BC009520	Homo sapiens, clone IMAGE:3637796, mRNA, partial cds.
Human	chr19:441032-459031:-	CD387847	-	-
Mouse	chr10:79565441-79572748:+	AF303106	I830164E09	gene trap ROSA b-geo 22
Mouse	chr10:79564299-79566763:-	5330431D23	5330431D23	unclassifiable
Human	chr19:959714-961164:+	CN366367	-	-
Human	chr19:960647-972173:-	AK095756	AK095756	Homo sapiens cDNA FLJ38437 fis, clone FEBRA2016572.
Mouse	chr10:79867257-79873732:+	AF396649	AF396649	Mus musculus NMDA-type glutamate receptor subunit NR3B precursor (Nr3B) mRNA, complete cds. CDS=186..3197
Mouse	chr10:79873441-79880872:-	BC049378	BC049378	Mus musculus, clone IMAGE:5102043, mRNA.
Human	chr19:1128557-1179434:+	BC019334	AF217978	Homo sapiens clone PP1471 unknown mRNA.
Human	chr19:1179286-1189026:-	AW157257	BC028156	Homo sapiens, clone MGC:40084 IMAGE:5241080, mRNA, complete cds.
Mouse	chr10:80013040-80027931:+	F830205I24	F830205I24	serine/threonine kinase 11
Mouse	chr10:80027792-80038192:-	BC026893	9430034L21	downstream of Stk11
Human	chr19:2115151-2183577:+	R14228	AL080221	Homo sapiens mRNA; cDNA DKFZp586P1823 (from clone DKFZp586P1823).
Human	chr19:2181018-2188703:-	BE731425	AK097723	Homo sapiens cDNA FLJ40404 fis, clone TESTI2037375, highly similar to Gnrpx gene product.
Mouse	chr10:80663403-80703884:+	C430040N23	AY196089	Mus musculus strain C57BL/6J histone H3 methyltransferase DOT1 variant a mRNA, complete cds. CDS=239..4861
Mouse	chr10:80702825-80703577:-	BC027780	BC027780	Mus musculus cDNA clone IMAGE:5353924, partial cds.
Human	chr19:2187816-2203072:+	BC009903	BC009903	Homo sapiens splicing factor 3a, subunit 2, 66kDa, mRNA (cDNA clone MGC:2362 IMAGE:2959124), complete cds.
Human	chr19:2181018-2188703:-	AK097723	AK097723	Homo sapiens cDNA FLJ40404 fis, clone TESTI2037375, highly similar to Gnrpx gene product.
Mouse	chr10:80706455-80713457:+	BU058822	BC052697	Mus musculus splicing factor 3a, subunit 2, mRNA (cDNA clone MGC:64703 IMAGE:5715282), complete cds. CDS=78..1535
Mouse	chr10:80704521-80707302:-	E430020G10	E430020G10	guanine nucleotide releasing protein x
Human	chr19:2340784-2377258:+	AK131261	AJ488946	Homo sapiens mRNA for polyserase-IA protein.
Human	chr19:1988464-2407994:-	AK024764	CR599500	full-length cDNA clone CS0DD008YP14 of Neuroblastoma Cot 50-normalized of Homo sapiens (human).
Mouse	chr10:80780396-80807919:+	K630039O11	-	-
Mouse	chr10:80807875-80809393:-	2810433C04	2810433C04	MITOCHONDRIAL IMPORT INNER MEMBRANE TRANSLOCASE SUBUNIT TIM13 B homolog [Homo sapiens]
Human	chr19:3490155-3495028:+	BX396051	-	-
Human	chr19:3489259-3525288:-	AF218008	BC068439	Homo sapiens chromosome 19 open reading frame 28, mRNA (cDNA clone MGC:86957 IMAGE:5314853), complete cds.
Mouse	chr10:81275027-81277780:-	2210409H10	2210409H10	unclassifiable
Mouse	chr10:81269451-81278193:+	G530009D07	F630110N24	hypothetical protein
Human	chr19:4198076-4220087:+	AK001236	AK001236	Homo sapiens cDNA FLJ10374 fis, clone NT2RM2001936, weakly similar to 32.3 KD PROTEIN IN CWP1-MBR1 INTERGENIC REGION.
Human	chr19:4197336-4198355:-	AK098628	AK098628	Homo sapiens cDNA FLJ25762 fis, clone TST06289.
Mouse	chr17:54238573-54247424:+	I920038O14	I920038O14	similar to Hypothetical protein (F20887_2) [Homo sapiens]
Mouse	chr17:54234258-54238875:-	I920067E03	I920067E03	weakly similar to Cytokine receptor-like molecule (Epstein-Barr virus induced gene 3) [Mus musculus]
Human	chr19:4353647-4396015:+	BU621106	BC067093	Homo sapiens chromatin assembly factor 1, subunit A (p150), mRNA (cDNA clone MGC:71229 IMAGE:6339027), complete cds.
Human	chr19:4395996-4408808:-	CR590857	AF272893	Homo sapiens UBX domain-containing protein 1 (UBXD1) mRNA, complete cds, alternatively spliced.
Mouse	chr17:54319717-54351692:+	4831431E06	4831431E06	chromatin assembly factor 1, subunit A (p150)
Mouse	chr17:54346449-54354431:-	G370040L01	C230058D01	hypothetical UBX domain containing protein
Human	chr19:6418823-6419847:+	BG422817	-	-
Human	chr19:6418213-6432798:-	BX647304	BC033437	Homo sapiens, hypothetical protein FLJ22757, clone MGC:34149 IMAGE:5187180, mRNA, complete cds.
Mouse	chr17:55390255-55399274:+	C730003G22	5730439B18	crumbs homolog 3 (Drosophila)
Mouse	chr17:55397201-55409768:-	BC035208	BC035208	Mus musculus RIKEN cDNA 4432409M07 gene, mRNA (cDNA clone MGC:30613 IMAGE:3671384), complete cds. CDS=105..2465
Human	chr19:6420476-6421206:+	BM981128	-	-
Human	chr19:6418213-6432798:-	BX647304	BC033437	Homo sapiens, hypothetical protein FLJ22757, clone MGC:34149 IMAGE:5187180, mRNA, complete cds.
Mouse	chr17:55390255-55399274:+	C730003G22	5730439B18	crumbs homolog 3 (Drosophila)
Mouse	chr17:55397201-55409768:-	BC035208	BC035208	Mus musculus RIKEN cDNA 4432409M07 gene, mRNA (cDNA clone MGC:30613 IMAGE:3671384), complete cds. CDS=105..2465
Human	chr19:7600668-7618760:+	BU732008	BC002869	Homo sapiens syntaxin binding protein 2, mRNA (cDNA clone MGC:10436 IMAGE:3942156), complete cds.
Human	chr19:7602501-7605531:-	BC025387	BC025387	Homo sapiens, similar to PURKINJE CELL PROTEIN 2 (PROTEIN PCD-5) (PURKINJE CELL-SPECIFIC PROTEIN L7) (H. sapiens), clone IMAGE:4824135, mRNA.
Mouse	chr8:3584025-3588964:+	2610025D16	2610025D16	unclassifiable
Mouse	chr8:3585846-3588009:-	BC014694	BC024853	Mus musculus Purkinje cell protein 2 (L7), mRNA (cDNA clone MGC:36149 IMAGE:5360866), complete cds. CDS=124..510
Human	chr19:8361082-8375323:+	CR605393	CR592281	full-length cDNA clone CS0DF006YF01 of Fetal brain of Homo sapiens (human).
Human	chr19:8345336-8361567:-	BG470121	-	-
Mouse	chr17:32234378-32252423:-	G270142I04	I920035M11	RAB11B, member RAS oncogene family
Mouse	chr17:32251885-32253764:+	5730424I07	5730424I07	unclassifiable

Human	chr19:8415651-8460002:+	AF061832	AF061832	Homo sapiens M4 protein deletion mutant mRNA, complete cds.
Human	chr19:8415455-8416481:-	BC045573	BC045573	Homo sapiens, clone IMAGE:4829644, mRNA.
Mouse	chr17:32138002-32210405:-	BC054785	BC054785	Mus musculus RIKEN cDNA 2610023M21 gene, mRNA (cDNA clone IMAGE:6821144), partial cds.
Mouse	chr17:32176546-32177487:+	1700001C07	1700001C07	unclassifiable
Human	chr19:10057973-10064927:+	BC026180	BC026180	Homo sapiens, Similar to hypothetical protein FLJ11286, clone IMAGE:4826265, mRNA.
Human	chr19:10064013-10074469:-	AB054064	AF230330	Homo sapiens angiopoietin-related protein 5 (ARP5) mRNA, complete cds.
Mouse	chr9:20796057-20801789:+	A230050P20	BC031510	Mus musculus RIKEN cDNA A230050P20 gene, mRNA (cDNA clone IMAGE:3492596), containing frame-shift errors.
Mouse	chr9:20801218-20807048:-	BC025904	AB054065	Mus musculus mRNA for AGF, complete cds. CDS=118..1491
Human	chr19:10242670-10258294:+	J03132	M24283	Human major group rhinovirus receptor (HRV) mRNA, complete cds.
Human	chr19:10257479-10260695:-	BX648637	BX648637	Homo sapiens mRNA; cDNA DKFZp686H2138 (from clone DKFZp686H2138).
Mouse	chr9:20942047-20961494:+	X52264	I830051F16	intercellular adhesion molecule
Mouse	chr9:20960984-20965034:-	B230208K21	B230208K21	unclassifiable
Human	chr19:10258650-10260260:+	BC000046	BC000046	Homo sapiens intercellular adhesion molecule 4, Landsteiner-Wiener blood group, mRNA (cDNA clone MGC:2108 IMAGE:3505509), complete cds.
Human	chr19:10257479-10260695:-	BX648637	BX648637	Homo sapiens mRNA; cDNA DKFZp686H2138 (from clone DKFZp686H2138).
Mouse	chr9:20962109-20963279:+	AF296282	AF296282	Mus musculus ICAM-4 mRNA, complete cds. CDS=1..789
Mouse	chr9:20960984-20965034:-	B230208K21	B230208K21	unclassifiable
Human	chr19:10304977-10308406:+	AL137656	AL137656	Homo sapiens mRNA; cDNA DKFZp434E0516 (from clone DKFZp434E0516).
Human	chr19:10276473-10305205:-	BC037428	BC037428	Homo sapiens, clone MGC:46327 IMAGE:5532899, mRNA, complete cds.
Mouse	chr9:21025384-21062015:+	1700084C06	1700084C06	hypothetical protein
Mouse	chr9:20995495-21025628:-	I920018J04	1300006N24	hypothetical RNA-binding region RNP-1 (RNA recognition motif) containing protein
Human	chr19:10515567-10525233:+	BC016845	AK056210	Homo sapiens cDNA FLJ31648 fis, clone NT2RI2004059.
Human	chr19:10524754-10537702:-	AL833189	AL833189	Homo sapiens mRNA; cDNA DKFZp667K055 (from clone DKFZp667K055).
Mouse	chr9:21201667-21292915:+	5330413J09	E430019L19	CTL2 protein homolog
Mouse	chr9:21209839-21224351:-	BC021438	BC021438	Mus musculus similar to hypothetical protein FLJ12949, mRNA (cDNA clone MGC:29361 IMAGE:5039543), complete cds. CDS=364..2124
Human	chr19:10689740-10805180:+	AK097967	L36983	Homo sapiens dynamin (DNM) mRNA, complete cds.
Human	chr19:10803743-10808274:-	BC018831	CR618553	full-length cDNA clone CS0DI032YH02 of Placenta Cot 25-normalized of Homo sapiens (human).
Mouse	chr9:21363631-21446930:+	BC010233	1300015B16	dynamin 2
Mouse	chr9:21446550-21483869:-	G730037J02	G730037J02	interleukin 1 receptor-like 1 ligand
Human	chr19:10843219-10894763:+	BC046240	AL833242	Homo sapiens mRNA; cDNA DKFZp761A1520 (from clone DKFZp761A1520).
Human	chr19:10893861-10900360:-	CR591972	BC013014	Homo sapiens hypothetical protein MGC3262, mRNA (cDNA clone MGC:4456 IMAGE:2960233), complete cds.
Mouse	chr9:21463843-21530141:+	BC036974	BC036974	Mus musculus coactivator-associated arginine methyltransferase 1, mRNA (cDNA clone MGC:46828 IMAGE:4935077), complete cds. CDS=99..1856
Mouse	chr9:21526196-21530349:-	BC003289	1300010K09	RIKEN cDNA 1300010K09 gene
Human	chr19:11316357-11327049:+	AK127284	AK127284	Homo sapiens cDNA FLJ45351 fis, clone BRHIP3011831.
Human	chr19:11267486-11323545:-	AK127147	AF161365	Homo sapiens HSPC102 mRNA, partial cds.
Mouse	chr9:21867590-21876011:+	2510048L02	2510048L02	hypothetical ATP/GTP-binding site motif A (P-loop) containing protein
Mouse	chr9:21860930-21867675:-	CA463339	BC010787	Mus musculus hypothetical protein MGC18837, mRNA (cDNA clone MGC:18837 IMAGE:4211629), complete cds. CDS=181..750
Human	chr19:12638616-12647646:+	CR602746	AK074525	Homo sapiens cDNA FLJ90044 fis, clone HEMBA1002195, weakly similar to VEGETATIBLE INCOMPATIBILITY PROTEIN HET-E-1.
Human	chr19:12639629-12643170:-	AK091030	AK091030	Homo sapiens cDNA FLJ33711 fis, clone BRAWH2008219.
Mouse	chr8:84353246-84359441:-	BC020047	BC020047	Mus musculus, RIKEN cDNA 1500041N16 gene, clone IMAGE:4013829, mRNA.
Mouse	chr8:84358794-84375766:+	I920182N12	1200017C17	mannosidase 2, alpha B1
Human	chr19:12638616-12647646:+	BC005870	AK074525	Homo sapiens cDNA FLJ90044 fis, clone HEMBA1002195, weakly similar to VEGETATIBLE INCOMPATIBILITY PROTEIN HET-E-1.
Human	chr19:12647531-12653716:-	CR625197	CR616605	full-length cDNA clone CS0DL012YH23 of B cells (Ramos cell line) Cot 25-normalized of Homo sapiens (human).
Mouse	chr8:84353246-84359441:-	AY365434	BC020047	Mus musculus, RIKEN cDNA 1500041N16 gene, clone IMAGE:4013829, mRNA.
Mouse	chr8:84338150-84353380:+	I420001K20	F830043F07	Hypothetical Trp-Asp repeat
Human	chr19:12797525-12804903:+	CK141575	-	-
Human	chr19:12797291-12807250:-	AK093672	AK093672	Homo sapiens cDNA FLJ36353 fis, clone THYMU2007307.
Mouse	chr8:84230077-84245313:-	AI390931	I920019O09	Ribonuclease HI large subunit (EC 3.1.26.-) (RNase HI large subunit)
Mouse	chr8:84222378-84232148:+	BC023051	BC022744	Mus musculus cDNA sequence BC022744, mRNA (cDNA clone MGC:31644 IMAGE:4527012), complete cds. CDS=361..1104
Human	chr19:12862957-12886195:+	BX364432	AK098370	Homo sapiens cDNA FLJ25504 fis, clone CBR04473, highly similar to GLUTARYL-COA DEHYDROGENASE PRECURSOR (EC 1.3.99.7).
Human	chr19:12870890-12891082:-	CR620067	CR620067	full-length cDNA clone CS0DI051YN13 of Placenta Cot 25-normalized of Homo sapiens (human).
Mouse	chr8:84158980-84168457:-	G730018L23	F520005M14	glutaryl-Coenzyme A dehydrogenase
Mouse	chr8:84146595-84162487:+	1700013H19	1700013H19	hypothetical protein

Human	chr19:12917644-12925715:+	D21235	BC014026	Homo sapiens RAD23 homolog A (S. cerevisiae), mRNA (cDNA clone MGC:20578 IMAGE:4300551), complete cds.
Human	chr19:12920960-12929068:-	BM989930	AF479749	Homo sapiens CR6 interacting factor 1 mRNA, complete cds.
Mouse	chr8:84108442-84115094:-	A130094C19	A130094C19	RAD23a homolog (S. cerevisiae)
Mouse	chr8:84106247-84109905:+	2810429D03	2810429D03	similar to CKII BETA BINDING PROTEIN 2 [Homo sapiens]
Human	chr19:14108980-14143079:+	BX537706	BX537706	Homo sapiens mRNA; cDNA DKFZp686D12196 (from clone DKFZp686D12196).
Human	chr19:14119549-14178619:-	BC052627	BC052627	Homo sapiens latrophilin 1, mRNA (cDNA clone IMAGE:6670989), partial cds.
Mouse	chr8:83199792-83222817:-	F930003C09	F930003C09	hypothetical protein
Mouse	chr8:83180420-83209428:+	AK122380	AK122380	Mus musculus mRNA for mKIAA0821 protein. CDS=3..4223
Human	chr19:16468215-16493180:+	BC027869	AK025395	Homo sapiens cDNA: FLJ21742 fis, clone COLF4912.
Human	chr19:16489700-16514341:-	BC021294	BC021294	Homo sapiens calcium homeostasis endoplasmic reticulum protein, mRNA (cDNA clone MGC:29449 IMAGE:4053575), complete cds.
Mouse	chr8:71475429-71494657:+	G430003B11	G430003B11	hypothetical protein
Mouse	chr8:71494129-71582407:-	C530047D17	C530035L23	similar to TRANSCRIPTIONAL CO-ACTIVATOR CRSP70 [Homo sapiens]
Human	chr19:16544435-16547154:+	AK094229	AK094229	Homo sapiens cDNA FLJ36910 fis, clone BRACE2003845.
Human	chr19:16546718-16600873:-	AK128435	AF104253	Homo sapiens transcriptional co-activator CRSP70 (CRSP70) mRNA, complete cds.
Mouse	chr8:71526959-71539524:+	E230012O07	D630017K22	unclassifiable
Mouse	chr8:71494129-71582407:-	BC054737	C530035L23	similar to TRANSCRIPTIONAL CO-ACTIVATOR CRSP70 [Homo sapiens]
Human	chr19:17281330-17295445:+	AL832922	CR609206	full-length cDNA clone CS0DI066YI13 of Placenta Cot 25-normalized of Homo sapiens (human).
Human	chr19:17295032-17306648:-	BC037307	AB046843	Homo sapiens mRNA for KIAA1623 protein, partial cds.
Mouse	chr8:70502507-70509419:+	BC034701	BC034701	Mus musculus RIKEN cDNA 1500034J01 gene, mRNA (cDNA clone MGC:27873 IMAGE:3494430), complete cds. CDS=90..398
Mouse	chr8:70509334-70518401:-	BC059855	BC027735	Mus musculus, Similar to KIAA1623 protein, clone IMAGE:3711771, mRNA.
Human	chr19:18519231-18541197:+	AK091145	BX648739	Homo sapiens mRNA; cDNA DKFZp686B1038 (from clone DKFZp686B1038).
Human	chr19:18540490-18541191:-	AI433681	-	-
Mouse	chr8:69546157-69566989:-	BC048938	BC048938	Mus musculus RIKEN cDNA 2810422J05 gene, mRNA (cDNA clone MGC:56993 IMAGE:6399490), complete cds. CDS=142..675
Mouse	chr8:69549279-69553602:+	6430409E23	4930522P08	unclassifiable
Human	chr19:19005394-19029987:+	BX648486	BX648486	Homo sapiens mRNA; cDNA DKFZp686M0272 (from clone DKFZp686M0272).
Human	chr19:18962696-19005807:-	BC020586	BC020586	Homo sapiens, clone MGC:22011 IMAGE:4400185, mRNA, complete cds.
Mouse	chr8:69256658-69270954:-	C130039E20	C130039E20	hypothetical Armadillo repeat/Armadillo/plakoglobin ARM repeat profile containing protein
Mouse	chr8:69270695-69316340:+	A630079E18	AF518875	Mus musculus arginine/serine-rich 14 splicing factor (Sfrs14) mRNA, complete cds. CDS=436..3639
Human	chr19:19164038-19173681:+	AF077196	AF094760	Homo sapiens RFXANK (RFXANK) mRNA, complete cds.
Human	chr19:19173218-19175242:-	BC057837	BC057837	Homo sapiens TR4 orphan receptor associated protein TRA16, mRNA (cDNA clone MGC:71819 IMAGE:30345563), complete cds.
Mouse	chr8:69165969-69174375:-	D330022L16	D330022L16	regulatory factor X-associated ankyrin-containing protein
Mouse	chr8:69166504-69168929:+	2500003K14	2500003K14	regulatory factor X-associated ankyrin-containing protein
Human	chr19:38377006-38391613:+	AL583533	AK129963	Homo sapiens cDNA FLJ26453 fis, clone KDN03319, highly similar to Homo sapiens immunoglobulin superfamily, member 1 (IGSF1).
Human	chr19:38391410-38408596:-	BC022457	BC022457	Homo sapiens, solute carrier family 7, (cationic amino acid transporter, y+ system) member 10, clone IMAGE:4796275, mRNA.
Mouse	chr7:23622031-23635387:-	A930030M05	A930030M05	LRp105 homolog [Rattus norvegicus]
Mouse	chr7:23608579-23623761:+	BC054765	6330436L12	solute carrier family 7 (cationic amino acid transporter, y+ system), member 10
Human	chr19:39437282-39538313:+	BX648432	AB002353	Human mRNA for KIAA0355 gene, complete cds.
Human	chr19:39537403-39538250:-	CA419762	-	-
Mouse	chr7:22651245-22728093:-	BC060233	4931406P16	hypothetical protein
Mouse	chr7:22648920-22666880:+	E330029N23	E330029N23	unclassifiable
Human	chr19:40716154-40728061:+	BC036373	BC036373	Homo sapiens glyceraldehyde-3-phosphate dehydrogenase, testis-specific, mRNA (cDNA clone MGC:26494 IMAGE:4824753), complete cds.
Human	chr19:40723468-40728771:-	CR627053	CR627053	Homo sapiens mRNA; cDNA DKFZp686A03139 (from clone DKFZp686A03139).
Mouse	chr7:19703911-19717900:-	M60978	M60978	Mus musculus testis-specific isoform of glyceraldehyde 3-phosphate dehydrogenase (Gapd-S) mRNA, complete cds. CDS=28..1350
Mouse	chr7:19703712-19730353:+	C630016N16	C630016N16	hypothetical protein
Human	chr19:41297031-41308690:+	CR607995	CR607995	full-length cDNA clone CS0DC029YC12 of Neuroblastoma Cot 25-normalized of Homo sapiens (human).
Human	chr19:41296451-41298088:-	BC067794	BC067794	Homo sapiens polymerase (RNA) II (DNA directed) polypeptide I, 14.5kDa, mRNA (cDNA clone MGC:87224 IMAGE:5285070), complete cds.
Mouse	chr7:19201758-19210030:-	CA532794	0610008B20	TUBULIN-SPECIFIC CHAPERONE B (TUBULIN FOLDING COFACTOR B) (CYTOSKELETON-ASSOCIATED PROTEIN CKAPI) homolog [Homo sapiens]
Mouse	chr7:19209687-19211148:+	BC062812	BC062812	Mus musculus cDNA clone MGC:73656 IMAGE:3466417, complete cds. CDS=273..650
Human	chr19:43446936-43487491:+	AF213678	CR598970	full-length cDNA clone CS0DI044YA09 of Placenta Cot 25-normalized of Homo sapiens (human).
Human	chr19:43485623-43487500:-	CR625148	CR625148	full-length cDNA clone CS0DC025YH23 of Neuroblastoma Cot 25-normalized of Homo sapiens (human).
Mouse	chr7:18220738-18222986:-	9130604L22	9130604L22	
Mouse	chr7:18212505-18223252:+	6820444L10	6820444L10	PP4519 homolog [Homo sapiens]

Human	chr19:43585616-43591569:+	BC036482	BC036482	Homo sapiens hypothetical protein LOC147965, mRNA (cDNA clone IMAGE:5260820), with apparent retained intron.
Human	chr19:43591535-43608785:-	AK097292	AY048120	Homo sapiens RAS guanyl releasing protein 4 variant 1 (RASGRP4) mRNA, complete cds.
Mouse	chr7:18126488-18130214:-	BC058765	B230110F21	hypothetical protein
Mouse	chr7:18109026-18127933:+	F630118K18	F630118K18	RAS GUANYL RELEASING PROTEIN 4 DELETION FORM homolog [Mus musculus]
Human	chr19:43830100-43913011:+	CR596416	BC005033	Homo sapiens, clone MGC:12692 IMAGE:3842046, mRNA, complete cds.
Human	chr19:43912662-43919471:-	BC040022	AK125713	Homo sapiens cDNA FLJ43725 fis, clone TESOP2007978, moderately similar to Mus musculus Capn12 gene for calpain 12.
Mouse	chr7:17862265-17931375:-	I0C0003G06	I0C0003G06	actinin alpha 4
Mouse	chr7:17850634-17862607:+	AJ289243	AJ289243	Mus musculus aberrrant Capn12c gene transcript, clone IMAGE cDNA 914413. CDS=77..1426
Human	chr19:46548656-46629487:+	M22221	AK127598	Homo sapiens cDNA FLJ45695 fis, clone FEBRA2013570, highly similar to 2-oxoisovalerate dehydrogenase alpha subunit, mitochondrial precursor (EC 1.2.4.4).
Human	chr19:46621999-46626475:-	BM144285	AY277592	Homo sapiens beta galactosyltransferase (BGALT15) mRNA, complete cds.
Mouse	chr7:14355623-14385026:-	L47335	F630105A02	branched chain ketoacid dehydrogenase E1, alpha polypeptide
Mouse	chr7:14352750-14361286:+	F630010G18	F630010G18	Similar to beta-1,3-N-acetylglucosaminyltransferase 1 (Inferred: waekly similar to beta-1)
Human	chr19:47426007-47428907:+	AL545136	-	-
Human	chr19:47426178-47451142:-	D63424	BC027984	Homo sapiens, glycogen synthase kinase 3 alpha, clone MGC:40141 IMAGE:3903896, mRNA, complete cds.
Mouse	chr7:13963394-13972752:+	BC022632	D030024H03	weakly similar to DNA-BINDING PROTEIN (FRAGMENT) [Homo sapiens]
Mouse	chr7:13970721-13980341:-	2700086H06	2700086H06	GLYCOGEN SYNTHASE KINASE 3 (FRAGMENT) homolog [Xenopus laevis]
Human	chr19:47593161-47604436:+	BU566442	-	-
Human	chr19:47597499-47623418:-	BC070041	BC070041	Homo sapiens lipase, hormone-sensitive, mRNA (cDNA clone MGC:87080 IMAGE:5296155), complete cds.
Mouse	chr7:14120047-14293692:+	4732471J01	4930568C20	lipase, hormone sensitive
Mouse	chr7:14122702-14139217:-	4932412K13	4932412K13	lipase, hormone sensitive
Human	chr19:47619747-47850256:+	AK096849	AK096849	Homo sapiens cDNA FLJ39530 fis, clone PUAEN2004400.
Human	chr19:47597499-47623418:-	BC029301	BC070041	Homo sapiens lipase, hormone-sensitive, mRNA (cDNA clone MGC:87080 IMAGE:5296155), complete cds.
Mouse	chr7:14120047-14293692:+	4732471J01	4930568C20	lipase, hormone sensitive
Mouse	chr7:14122702-14139217:-	4932412K13	4932412K13	lipase, hormone sensitive
Human	chr19:48808093-48810490:+	BC034980	BC034980	Homo sapiens, clone IMAGE:4824581, mRNA.
Human	chr19:48803217-48815854:-	BC015950	BC045799	Homo sapiens, Similar to RIKEN cDNA 2410005H09 gene, clone MGC:51082 IMAGE:4813427, mRNA, complete cds.
Mouse	chr7:13247688-13249671:-	K630003F05	-	-
Mouse	chr7:13244211-13252887:+	2410005H09	A730069J11	hypothetical protein
Human	chr19:50528532-50546626:+	BC062998	BC020346	Homo sapiens kinesin light chain 2-like, mRNA (cDNA clone IMAGE:3863271).
Human	chr19:50544875-50566016:-	CR613691	X52221	H.sapiens ERCC2 gene, exons 1 & 2 (partial).
Mouse	chr7:10917514-10927181:-	BC020422	F930028G15	kinesin light chain 3
Mouse	chr7:10905087-10918776:+	1200008E14	D030024L06	excision repair cross-complementing rodent repair deficiency, complementation group 2
Human	chr19:50601307-50605864:+	U86751	U86751	Human nucleolar fibrillar center protein (ASE-1) mRNA, complete cds.
Human	chr19:50602395-50673926:-	AK092039	AK092039	Homo sapiens cDNA FLJ34720 fis, clone MESAN2005724, highly similar to DNA EXCISION REPAIR PROTEIN ERCC-1.
Mouse	chr7:10879084-10882777:-	CD776010	-	-
Mouse	chr7:10867855-10879602:+	BC011224	E430009H20	excision repair cross-complementing rodent repair deficiency, complementation group 1
Human	chr19:53650578-53677383:+	AF081466	AF081466	Homo sapiens inward rectifier potassium channel (KIR2.4) mRNA, complete cds.
Human	chr19:53660415-53664762:-	BC042033	BC042033	Homo sapiens, clone IMAGE:4827822, mRNA.
Mouse	chr7:33210638-33218643:-	6330419D17	B230202D16	pleckstrin homology, Sec7 and coiled/coil domains 2
Mouse	chr7:33218023-33218920:+	1700000A01	1700000A01	unclassifiable
Human	chr19:53814311-53825785:+	AK000599	BC006161	Homo sapiens, clone MGC:13202 IMAGE:3677636, mRNA, complete cds.
Human	chr19:53825099-53832448:-	D28468	CR616380	full-length cDNA clone CS0DC026YN24 of Neuroblastoma Cot 25-normalized of Homo sapiens (human).
Mouse	chr7:33111906-33120445:-	4933402E20	AF245448	Mus musculus sphingosine kinase type 2 isoform mRNA, complete cds. CDS=387..2240
Mouse	chr7:33107636-33112646:+	B230364H12	B230364H12	D site albumin promoter binding protein
Human	chr19:53915962-53930062:+	AI915980	-	-
Human	chr19:53915654-53935790:-	AY378097	AY378097	Homo sapiens Ras-interacting protein mRNA, complete cds.
Mouse	chr7:33038383-33041028:-	1110025D03	1110025D03	unclassifiable
Mouse	chr7:33024074-33041357:+	F830032D24	F830032D24	Ras interacting protein 1
Human	chr19:54002438-54005903:+	CB122240	-	-
Human	chr19:53990131-54006494:-	AK093627	AK093627	Homo sapiens cDNA FLJ36308 fis, clone THYMU2004916, highly similar to BRANCHED-CHAIN AMINO ACID AMINOTRANSFERASE, MITOCHONDRIAL PRECURSOR (EC 2.6.1.42).
Mouse	chr7:32969471-32977007:-	0610005C13	0610005C13	unclassifiable
Mouse	chr7:32971833-32991613:+	9430051K24	G930041K17	branched chain aminotransferase 2, mitochondrial

Human	chr19:54309430-54313529:+	AF311862	AY358744	Homo sapiens clone DNA143514 LIN-7B (UNQ3116) mRNA, complete cds.
Human	chr19:54313467-54314209:-	BC051004	AK001352	Homo sapiens cDNA FLJ10490 fis, clone NT2RP2000233.
Mouse	chr7:32763036-32765954:-	AF173082	BC031780	Mus musculus lin 7 homolog b (C. elegans), mRNA (cDNA clone MGC:18338 IMAGE:4164883), complete cds. CDS=26..649
Mouse	chr7:32761850-32766442:+	U88401	A730043P17	metastasis associated 2
Human	chr19:54648238-54681300:+	AK074136	AK074136	Homo sapiens mRNA for FLJ00209 protein.
Human	chr19:54641362-54648566:-	CA454187	CR611670	full-length cDNA clone CS0DJ015YP07 of T cells (Jurkat cell line) Cot 10-normalized of Homo sapiens (human).
Mouse	chr7:32535896-32549928:-	D130077D09	I920189H05	Similar to putatively membrane-anchored aldehyde dehydrogenase homolog [Mus musculus]
Mouse	chr7:32549647-32555328:+	1110061L23	1110061L23	4933413A04Rik protein (1110061L23Rik protein) (Hypothetical aldehyde dehydrogenase family containing protein)
Human	chr19:54962057-55002185:+	AK096757	BC014214	Homo sapiens adaptor-related protein complex 2, alpha 1 subunit, mRNA (cDNA clone MGC:20679 IMAGE:4111165), complete cds.
Human	chr19:55001935-55008379:-	AK057964	AK026341	Homo sapiens cDNA: FLJ22688 fis, clone HSI11003.
Mouse	chr7:32294336-32324943:-	BC031433	X14971	Mouse mRNA for alpha-adaptin (A). CDS=206..3139
Mouse	chr7:32290041-32296859:+	B230309D13	B230309D13	CDNA: FLJ22688 FIS, CLONE HSI11003 homolog [Homo sapiens]
Human	chr19:55045790-55055813:+	CR607948	AK057817	Homo sapiens cDNA FLJ25088 fis, clone CBL08845, highly similar to Homo sapiens PTOV1 (PTOV1) gene.
Human	chr19:55051527-55062978:-	AL050131	BC033822	Homo sapiens, clone MGC:45178 IMAGE:5218400, mRNA, complete cds.
Mouse	chr7:32256884-32263684:-	BC024632	BC024632	Mus musculus prostate tumor over expressed gene 1, mRNA (cDNA clone MGC:28629 IMAGE:4222241), complete cds. CDS=268..1518
Mouse	chr7:32250737-32259211:+	BM231626	C630037M17	polynucleotide kinase 3'- phosphatase
Human	chr19:55072550-55083819:+	BC003516	BC003516	Homo sapiens, Similar to RIKEN cDNA 4432405K22 gene, clone MGC:10751 IMAGE:3050014, mRNA, complete cds.
Human	chr19:55064103-55124600:-	AK074097	BC026103	Homo sapiens, clone IMAGE:4822638, mRNA.
Mouse	chr7:32229753-32242677:-	BC017607	E330015O10	interleukin-four induced gene 1
Mouse	chr7:32242303-32249016:+	I020030P12	D030060P21	AKT1 substrate 1 (proline-rich)
Human	chr19:55123535-55129004:+	AK091985	AK091985	Homo sapiens cDNA FLJ34666 fis, clone LIVER2000682.
Human	chr19:55064103-55124600:-	AK125857	BC026103	Homo sapiens, clone IMAGE:4822638, mRNA.
Mouse	chr7:32205853-32210255:-	I420025O10	I420025O10	activating transcription factor 5
Mouse	chr7:32209685-32234406:+	F730216I21	4930569A11	interleukin 4 induced 1
Human	chr19:55851112-55854379:+	AI862592	-	-
Human	chr19:55854326-55912007:-	BU633092	AF163302	Homo sapiens somatostatin receptor interacting protein splice variant a (SSTRIP) mRNA, complete cds.
Mouse	chr7:31754278-31780493:-	1700008O03	1700008O03	hypothetical protein
Mouse	chr7:31753806-31754324:+	BX634888	-	-
Human	chr20:2031168-2105681:+	AA889134	BC017340	Homo sapiens serine/threonine kinase 35, mRNA (cDNA clone IMAGE:4869353), partial cds.
Human	chr20:2029659-2031525:-	BC034792	BC034792	Homo sapiens, clone IMAGE:4823381, mRNA, partial cds.
Mouse	chr2:129549054-129581310:+	G730027B12	G730027B12	Similar to serine/threonine kinase 35 (Fragment) homolog [Mus musculus]
Mouse	chr2:129547387-129550509:-	4932416H05	4932416H05	unclassifiable
Human	chr20:5934601-5968703:+	AL832419	AL832419	Homo sapiens mRNA; cDNA DKFZp762C112 (from clone DKFZp762C112).
Human	chr20:5965538-5967573:-	AK026099	AK026099	Homo sapiens cDNA: FLJ22446 fis, clone HRC09457.
Mouse	chr2:132595689-132615808:+	5730490M08	0610009I22	similar to DJ967N21.6 (NOVEL CDP-ALCOHOL PHOSPHATIDYLTRANSFERASE FAMILY MEMBER PROTEIN) (CAPTASE-ISO) (HYPOTHETICAL 32.6 KDA PROTEIN) [Homo sapiens]
Mouse	chr2:132614671-132615888:-	E230002C16	E230002C16	unclassifiable
Human	chr20:19981194-20289349:+	BC028708	BC028708	Homo sapiens chromosome 20 open reading frame 26, mRNA (cDNA clone MGC:26982 IMAGE:4825941), complete cds.
Human	chr20:19962860-19984690:-	AF318303	AF318303	Homo sapiens CGI-201 protein, type II mRNA, alternatively spliced, complete cds.
Mouse	chr2:145686763-145795051:+	A230061L07	4930529M08	weakly similar to DJ1178H5.4.3 (NOVEL PROTEIN (ISOFORM 3)) (FRAGMENT) [Homo sapiens]
Mouse	chr2:145668682-145686993:-	B020019C18	1200013P10	MSTP021 homolog [Homo sapiens]
Human	chr20:25552543-25606184:+	BG329805	-	-
Human	chr20:25541571-25553136:-	AK074335	AL833116	Homo sapiens mRNA; cDNA DKFZp313O1829 (from clone DKFZp313O1829).
Mouse	chr2:151102333-151103732:+	A130094D17	A130094D17	unclassifiable
Mouse	chr2:151093034-151102747:-	D730045K14	1600031M04	DJ694B14.3 (NOVEL HALOACID DEHALOGENASE-LIKE HYDROLASE FAMILY PROTEIN SIMILAR TO (ARCHAEA) BACTERIAL PROTEINS) (FRAGMENT) homolog [Homo sapiens]
Human	chr20:33026867-33053908:+	BC007808	AB040945	Homo sapiens mRNA for KIAA1512 protein, partial cds.
Human	chr20:33053867-33144332:-	BC008836	AK074106	Homo sapiens mRNA for FLJ00177 protein.
Mouse	chr2:155692409-155697656:+	AW121532	BC047419	Mus musculus, clone IMAGE:5356392, mRNA.
Mouse	chr2:155697626-155755733:-	BC057330	BC057330	Mus musculus transient receptor potential cation channel, subfamily C, member 4 associated protein, mRNA (cDNA clone MGC:66944 IMAGE:6816155), complete cds. CDS=37..2430
Human	chr20:33278108-33328218:+	BC047614	BC047614	Homo sapiens, similar to matrix metalloproteinase 24 (membrane-inserted), clone IMAGE:5768916, mRNA, partial cds.
Human	chr20:33275983-33336202:-	CR615992	AF047433	Homo sapiens b(2)gcn homolog mRNA, complete cds.
Mouse	chr2:155838691-155881719:+	AB021226	AB021226	Mus musculus mRNA for membrane-type-5 matrix metalloproteinase, complete cds. CDS=75..1931
Mouse	chr2:155881079-155882629:-	2310008C15	I420019L15	weakly similar to Collagen-like protein [Herpesvirus saimiri]

Human	chr20:33506399-33568774:+	AF022655	AF049105	Homo sapiens centrosomal Nek2-associated protein 1 (C-NAP1) mRNA, complete cds.
Human	chr20:33527613-33542159:-	AW134548	-	-
Mouse	chr2:156019907-156062249:+	I1C0015M14	I1C0015M14	Similar to centrosomal protein 2 homolog (Fragment)
Mouse	chr2:156036628-156071800:-	B020006H19	B020006H19	hypothetical protein
Human	chr20:33750608-33752336:+	BM353871	AK000548	Homo sapiens cDNA FLJ20541 fis, clone KAT11364.
Human	chr20:33720025-33750695:-	AF097025	AK001470	Homo sapiens cDNA FLJ10608 fis, clone NT2RP2005239, highly similar to Homo sapiens cysteine desulfurase mRNA.
Mouse	chr2:156207502-156209145:+	AA058111	2010100O12	weakly similar to CG6878 protein (RH56103p) [Drosophila melanogaster]
Mouse	chr2:156186986-156207537:-	C820017M03	I420032E18	nitrogen fixation gene 1 (S. cerevisiae)
Human	chr20:34005936-34006704:+	BX097319	-	-
Human	chr20:34004960-34010808:-	BC041022	BC041022	Homo sapiens, similar to SCAN domain containing 1, clone IMAGE:3830122, mRNA.
Mouse	chr2:156375648-156438987:+	4921517L17	4921517L17	similar to DJ1121G12.3 (NOVEL GENE) (FRAGMENT) [Homo sapiens]
Mouse	chr2:156375195-156376096:-	2310003H23	2310003H23	LEUCINE-RICH-DOMAIN INTER-ACTING PROTEIN 1 (PPARGAMMA COFACTOR 2) (PEROXISOME PROLIFERATIVE ACTIVATED RECEPTOR, GAMMA, COACTIVATOR 2) homolog [Mus musculus]
Human	chr20:34635290-34674381:+	AF112213	CR618600	full-length cDNA clone CS0DI004YN09 of Placenta Cot 25-normalized of Homo sapiens (human).
Human	chr20:34674138-34708004:-	AK025645	BC042041	Homo sapiens, Similar to Src-like-adaptor 2, clone MGC:49845 IMAGE:4429896, mRNA, complete cds.
Mouse	chr2:156926343-156936912:+	0610010C15	1110008F13	PUTATIVE RAB5-INTERACTING PROTEIN (DJ977B1.3.1) homolog [Homo sapiens]
Mouse	chr2:156935806-156950570:-	5830437K10	5830437K10	MODULATOR OF ANTIGEN RECEPTOR SIGNALING MARS
Human	chr20:35157602-35158995:+	BC017557	BC017557	Homo sapiens, clone IMAGE:3908672, mRNA, partial cds.
Human	chr20:35058166-35157823:-	L14812	L14812	Human retinoblastoma related protein (p107) mRNA, complete cds.
Mouse	chr2:157267653-157270163:+	2610301H18	2610301H18	hypothetical protein
Mouse	chr2:157209242-157267885:-	I920096E10	U27177	Mus musculus p107 (p107) mRNA, complete cds. CDS=64..3255
Human	chr20:35240227-35303440:+	Y00282	Y00282	Human mRNA for ribophorin II.
Human	chr20:35163043-35241405:-	AK093432	BC030006	Homo sapiens, clone IMAGE:4822338, mRNA.
Mouse	chr2:157342355-157389671:+	1500001H18	1500001H18	ribophorin II
Mouse	chr2:157271895-157342898:-	4930455J15	4921509H06	hypothetical ARM repeat structure containing protein
Human	chr20:42541178-42556658:+	AK056178	AK056178	Homo sapiens cDNA FLJ31616 fis, clone NT2RI2003019.
Human	chr20:42556541-42558052:-	AA426598	-	-
Mouse	chr2:163665661-163682362:+	BC052389	F830034J19	##############
Mouse	chr2:163682256-163683266:-	9230112C11	9230112C11	unclassifiable
Human	chr20:43368904-43379876:+	AB024964	AB024964	Homo sapiens mRNA for transcription factor RBP-L, complete cds.
Human	chr20:43355501-43370381:-	AK074597	AK074597	Homo sapiens cDNA FLJ90116 fis, clone HEMBA1006902, highly similar to Homo sapiens mRNA for matrilin-4.
Mouse	chr2:164466254-164478561:+	D430025K11	Y10926	M.musculus mRNA for transcription factor RBP-L. CDS=104..1651
Mouse	chr2:164417417-164468273:-	A530080M16	U73004	Mus musculus secretory leukocyte protease inhibitor mRNA, complete cds. CDS=447..842
Human	chr20:43423991-43472664:+	CR622797	BC023562	Homo sapiens chromosome 20 open reading frame 35, mRNA (cDNA clone IMAGE:3639906).
Human	chr20:43435936-43469943:-	AK098015	AB017802	Homo sapiens mRNA for clg01, complete cds.
Mouse	chr2:164549242-164556435:+	BC030298	BC030298	Mus musculus DNA segment, Chr 2, Brigham & Women's Genetics 0891 expressed, mRNA (cDNA clone MGC:40737 IMAGE:5364949), complete cds. CDS=391..867
Mouse	chr2:164548644-164549576:-	G930023N09	G930023N09	hypothetical protein
Human	chr20:43895856-43905321:+	AK095851	AK095851	Homo sapiens cDNA FLJ38532 fis, clone HCHON2001099.
Human	chr20:43903767-43919451:-	AF014404	X86032	H.sapiens mRNA for thioesterase II.
Mouse	chr2:164849099-164856923:+	I920026B11	I920026B11	Sorting nexin 21 homolog [Homo sapiens]
Mouse	chr2:164852573-164867994:-	9130233C05	9130233C05	PEROXISOMAL ACYL-COENZYME A THIOESTER HYDROLASE 1 (EC 3.1.2.2) (PEROXISOMAL LONG-CHAIN ACYL-COA THIOESTERASE 1) homolog [Mus musculus]
Human	chr20:43952190-43960867:+	AK172808	AK172808	Homo sapiens cDNA FLJ23969 fis, clone HEP16905, highly similar to Lysosomal protective protein precursor (EC 3.4.16.5).
Human	chr20:43950163-43953333:-	AK054821	AK054821	Homo sapiens cDNA FLJ30259 fis, clone BRACE2002478, weakly similar to NEURALIZED PROTEIN.
Mouse	chr2:164895985-164904145:+	5031400F07	6430411I23	protective protein for beta-galactosidase
Mouse	chr2:164893843-164896676:-	1190009E12	1190009E12	hypothetical SOCS domain, C-terminus of STAT-inhibitors containing protein
Human	chr20:43952190-43960867:+	CR625597	AK172808	Homo sapiens cDNA FLJ23969 fis, clone HEP16905, highly similar to Lysosomal protective protein precursor (EC 3.4.16.5).
Human	chr20:43959579-43974374:-	CR597431	CR597431	full-length cDNA clone CS0DI012YG11 of Placenta Cot 25-normalized of Homo sapiens (human).
Mouse	chr2:164895985-164904145:+	6430411I23	6430411I23	protective protein for beta-galactosidase
Mouse	chr2:164902631-164920834:-	U37226	G370093F01	phospholipid transfer protein
Human	chr20:43996657-44010786:+	AA972428	AB050014	Homo sapiens mRNA for posphorylated CTD interacting factor PCIF1, complete cds.
Human	chr20:44010700-44034240:-	AF395833	BC041331	Homo sapiens zinc finger protein 335, mRNA (cDNA clone IMAGE:5285131), partial cds.
Mouse	chr2:164942417-164957567:+	A630040N15	F730014I05	BA465L10.1 (NOVEL PROTEIN SIMILAR TO DROSOPHILA CG11399) (HYPOTHETICAL 80.7 KDA PROTEIN) homolog [Homo sapiens]
Mouse	chr2:164954990-164974870:-	BC062921	BC062921	Mus musculus zinc finger protein 335, mRNA (cDNA clone MGC:86001 IMAGE:6848450), complete cds. CDS=285..2081

Human	chr20:49049314-49059963:+	CF994282	-	-
Human	chr20:49053600-49073054:-	BC046629	BC046629	Homo sapiens potassium voltage-gated channel, subfamily G, member 1, transcript variant 1, mRNA (cDNA clone MGC:41883 IMAGE:5287759), complete cds.
Mouse	chr2:168331602-168332449:+	4930570F10	4930570F10	unclassifiable
Mouse	chr2:168323235-168344854:-	L230004N17	-	-
Human	chr20:60918044-60942972:+	BC004412	L41162	Homo sapiens collagen alpha 3 type IX (COL9A3) mRNA, complete cds.
Human	chr20:60942811-60963602:-	AF070992	AB012124	Homo sapiens TCFL5 mRNA for transcription factor-like 5, complete cds.
Mouse	chr2:180285861-180310396:+	AF237721	5230401M17	procollagen, type IX, alpha 3
Mouse	chr2:180309947-180330933:-	AY234363	AY234363	Mus musculus factor in the germline beta (Figlb) mRNA, complete cds. CDS=38..1366
Human	chr20:61355775-61360320:+	AK131543	AK131543	Homo sapiens cDNA FLJ16779 fis, clone BRHIP3038037.
Human	chr20:61342577-61374496:-	BC041812	BC041812	Homo sapiens, Similar to chromosome 20 open reading frame 58, clone MGC:41932 IMAGE:5302801, mRNA, complete cds.
Mouse	chr2:180642671-180655019:+	A430053N14	A430053N14	unclassifiable
Mouse	chr2:180622976-180643061:-	C030019F02	C030019F02	BA261N11.2.2 (NOVEL PROTEIN, ISOFORM 2) (FRAGMENT) homolog [Homo sapiens]
Human	chr20:62055181-62058782:+	AK128497	AK128497	Homo sapiens cDNA FLJ46647 fis, clone TRACH3004288.
Human	chr20:62041498-62058215:-	AJ605558	AJ605558	Homo sapiens mRNA for putative uridine kinase (URKL1 gene), splice form F538DC.
Mouse	chr2:181266598-181273288:+	A330042M09	A330042M09	unclassifiable
Mouse	chr2:181257267-181280884:-	K530026P10	BC016535	Mus musculus RIKEN cDNA 1110007H10 gene, mRNA (cDNA clone MGC:27764 IMAGE:2655873), complete cds. CDS=6..1652
Human	chr20:62055181-62058782:+	AK128497	AK128497	Homo sapiens cDNA FLJ46647 fis, clone TRACH3004288.
Human	chr20:62058499-62071667:-	AB033022	AB033022	Homo sapiens mRNA for KIAA1196 protein, partial cds.
Mouse	chr2:181266598-181273288:+	A330042M09	A330042M09	unclassifiable
Mouse	chr2:181257267-181280884:-	K530026P10	BC016535	Mus musculus RIKEN cDNA 1110007H10 gene, mRNA (cDNA clone MGC:27764 IMAGE:2655873), complete cds. CDS=6..1652
Human	chr21:26028752-26066642:+	BX647755	BX647755	Homo sapiens mRNA; cDNA DKFZp686G2131 (from clone DKFZp686G2131).
Human	chr21:26010686-26029855:-	BC001178	BC001178	Homo sapiens, ATP synthase, H+ transporting, mitochondrial F0 complex, subunit F6, clone MGC:2243 IMAGE:3357779, mRNA, complete cds.
Mouse	chr16:85217526-85246475:+	B020037D14	BC052448	Mus musculus GA repeat binding protein, alpha, mRNA (cDNA clone MGC:63455 IMAGE:6408997), complete cds. CDS=447..1811
Mouse	chr16:85210495-85218252:-	BC010766	BC010766	Mus musculus ATP synthase, H+ transporting, mitochondrial F0 complex, subunit F, mRNA (cDNA clone MGC:18567 IMAGE:4219715), complete cds. CDS=345..671
Human	chr21:31953792-31963115:+	BT006676	X02317	Human mRNA for Cu/Zn superoxide dismutase (SOD).
Human	chr21:31960912-31964017:-	AI420884	-	-
Mouse	chr16:90655127-90661182:+	9530064N04	BC048874	Mus musculus superoxide dismutase 1, soluble, mRNA (cDNA clone MGC:58998 IMAGE:5401293), complete cds. CDS=117..581
Mouse	chr16:90659210-90719003:-	BM240246	B130053I10	CTD-BINDING SR-LIKE PROTEIN RA4 homolog [Rattus norvegicus]
Human	chr21:33837220-33872900:+	BC046101	BC046101	Homo sapiens, Similar to SON DNA binding protein, clone IMAGE:5552607, mRNA.
Human	chr21:33853716-33938102:-	AK001274	BC033023	Homo sapiens crystallin, zeta (quinone reductase)-like 1, transcript variant 1, mRNA (cDNA clone MGC:32911 IMAGE:5269248), complete cds.
Mouse	chr16:92112180-92144083:+	1110025J21	AK122418	Mus musculus mRNA for mKIAA1019 protein. CDS=2..1444
Mouse	chr16:92131224-92193331:-	3830417A16	5830446D17	SIMILAR TO CRYSTALLIN, ZETA (QUINONE REDUCTASE)-LIKE 1 homolog [Mus musculus]
Human	chr21:44351604-44375491:+	X95263	X95263	H.sapiens mRNA for PWP2 protein.
Human	chr21:44375368-44377770:-	AK096268	AK096268	Homo sapiens cDNA FLJ38949 fis, clone NT2NE2018376.
Mouse	chr10:78054244-78068508:-	E430008H02	E430008H02	hypothetical Quinoprotein alcohol dehydrogenase structure containing protein
Mouse	chr10:78053360-78056245:+	F730043H12	F730043H12	unclassifiable
Human	chr21:46529342-46530507:+	BG177516	-	-
Human	chr21:46479397-46530526:-	AJ010089	AJ010089	Homo sapiens mRNA for GANP protein.
Mouse	chr10:76331419-76340964:-	BC057308	BC057308	Mus musculus RIKEN cDNA A130042E20 gene, mRNA (cDNA clone MGC:66581 IMAGE:5703033), complete cds. CDS=362..856
Mouse	chr10:76340727-76387817:+	AK122313	AK122313	Mus musculus mRNA for mKIAA0572 protein. CDS=332..6310
Human	chr21:46568464-46690110:+	U52962	U52962	Human centrosomal protein kendrin mRNA, complete cds.
Human	chr21:46544523-46568852:-	BC031638	BC026042	Homo sapiens, clone IMAGE:4587655, mRNA.
Mouse	chr10:76222470-76314682:-	U05823	BC034865	Mus musculus pericentrin 2, mRNA (cDNA clone IMAGE:1383047), with apparent retained intron.
Mouse	chr10:76314142-76315638:+	4930406P12	4930406P12	hypothetical protein
Human	chr22:18303684-18332052:+	CR610886	AK130031	Homo sapiens cDNA FLJ26521 fis, clone KDN08050, highly similar to Catechol O-methyltransferase, membrane-bound form (EC 2.1.1.6).
Human	chr22:18237513-18304069:-	AF106697	AF106697	Homo sapiens thioredoxin reductase mRNA, complete cds.
Mouse	chr16:18183882-18203848:-	AU067212	5031438A14	catechol-O-methyltransferase
Mouse	chr16:18203421-18256077:+	AB027566	AB027566	Mus musculus TXNRD2 mRNA for thioredoxin reductase 2, complete cds. CDS=50..1612
Human	chr22:18303684-18332052:+	CR624969	AK130031	Homo sapiens cDNA FLJ26521 fis, clone KDN08050, highly similar to Catechol O-methyltransferase, membrane-bound form (EC 2.1.1.6).
Human	chr22:18328680-18378863:-	AL390148	U51269	Human armadillo repeat protein mRNA, complete cds.
Mouse	chr16:18183882-18203848:-	5031438A14	5031438A14	catechol-O-methyltransferase
Mouse	chr16:18125178-18184080:+	BC052393	BC052393	Mus musculus armadillo repeat gene deleted in velo-cardio-facial syndrome, mRNA (cDNA clone MGC:63360 IMAGE:5719806), complete cds. CDS=265..3135

Human	chr22:20308380-20308914:+	BM760073	-	-
Human	chr22:20306932-20308908:-	CR600451	CR600451	full-length cDNA clone CS0DH003YI08 of T cells (Jurkat cell line) of Homo sapiens (human).
Mouse	chr16:16915875-16925135:-	BC057359	BC057359	Mus musculus RIKEN cDNA 4930432J16 gene, mRNA (cDNA clone MGC:67213 IMAGE:6836034), complete cds. CDS=461..2086
Mouse	chr16:16921497-16937778:+	4930521M19	6430537H19	ubiquitin-conjugating enzyme E2L 3
Human	chr22:20337210-20376761:+	AK127259	AK127259	Homo sapiens cDNA FLJ45326 fis, clone BRHIP3006786, highly similar to Homo sapiens peptidylprolyl isomerase (cyclophilin)-like 2 (PPIL2).
Human	chr22:20376379-20379610:-	BC015229	AK096530	Homo sapiens cDNA FLJ39211 fis, clone OCBBF2006172.
Mouse	chr16:16863693-16888236:-	BC028899	2700063C07	peptidylprolyl isomerase (cyclophilin)-like 2
Mouse	chr16:16846834-16873494:+	4933412K12	4933412K12	yippee-like 1 (Drosophila)
Human	chr22:22429762-22432604:+	AK093880	AK093880	Homo sapiens cDNA FLJ36561 fis, clone TRACH2009348.
Human	chr22:22432575-22435184:-	CR594120	BC065232	Homo sapiens cDNA clone MGC:70831 IMAGE:5747937, complete cds.
Mouse	chr10:75808501-75811450:-	G730041L12	G730041L12	weakly similar to N27C7-3 protein [Homo sapiens]
Mouse	chr10:75803908-75808528:+	0610006L21	AY238604	Mus musculus Nur77 downstream protein 2 mRNA, complete cds. CDS=392..808
Human	chr22:23275985-23349526:+	AA005024	M24903	Human gamma-glutamyl transpeptidase (GGT) mRNA, complete cds.
Human	chr22:23260945-23276457:-	AK098754	BC002924	Homo sapiens hypothetical protein MGC1842, mRNA (cDNA clone IMAGE:3956179), partial cds.
Mouse	chr10:75388351-75408171:+	8430422N14	3300001O07	SMALL NUCLEAR RIBONUCLEOPROTEIN SM D3 (SNRNP CORE PROTEIN D3) (SM-D3) homolog [Homo sapiens]
Mouse	chr10:75186461-75388772:-	E430026O05	A530029A01	CDNA FLJ30119 FIS, CLONE BRACE1000070 homolog [Homo sapiens]
Human	chr22:25204400-25215559:+	CR599541	BC066962	Homo sapiens cDNA clone MGC:87474 IMAGE:4820477, complete cds.
Human	chr22:25211508-25233004:-	AK025739	AL050258	Novel human mRNA similar to mouse tuftelin-interacting protein 10 mRNA, AF097181.
Mouse	chr5:109684285-109692654:-	BC030678	2810002G02	tuftelin-interacting protein, 39 kD
Mouse	chr5:109675974-109687695:+	BC017682	G370042G14	tuftelin interacting protein 11
Human	chr22:25377802-25580454:+	AK127256	AK026502	Homo sapiens cDNA: FLJ22849 fis, clone KAIA987.
Human	chr22:25389811-25395229:-	H55674	-	-
Mouse	chr5:109562840-109578560:-	CB520069	A230057G18	unclassifiable
Mouse	chr5:109566672-109568086:+	CR520635	-	-
Human	chr22:27988618-28021069:+	CD358294	X66899	H.sapiens EWS mRNA.
Human	chr22:27980395-27988752:-	BM564366	CR594348	full-length cDNA clone CS0DI032YL04 of Placenta Cot 25-normalized of Homo sapiens (human).
Mouse	chr11:4964123-4994054:-	B230384I08	C530046A18	Ewing sarcoma homolog
Mouse	chr11:4993580-5000888:+	BE949317	F830022F09	hypothetical Ubiquitin-associated domain/Leucine-rich region profile/UBA-like containing protein
Human	chr22:30012237-30058561:+	BE798820	-	-
Human	chr22:30002133-30013078:-	CR612099	CR626709	full-length cDNA clone CS0DL003YN05 of B cells (Ramos cell line) Cot 25-normalized of Homo sapiens (human).
Mouse	chr11:3200214-3225966:-	4732480K10	4732480K10	hypothetical protein
Mouse	chr11:3225516-3237759:+	F630109D05	F630109D05	HGFL(L) protein
Human	chr22:30216755-30339091:+	AB011114	AB011114	Homo sapiens mRNA for KIAA0542 protein, partial cds.
Human	chr22:30339031-30382784:-	AF035304	AL050371	Homo sapiens mRNA; cDNA DKFZp566G2246 (from clone DKFZp566G2246).
Mouse	chr11:3026638-3098130:-	5330402M10	B230377G14	weakly similar to KIAA0542 PROTEIN (FRAGMENT) [Homo sapiens]
Mouse	chr11:3018809-3034570:+	BM197378	-	-
Human	chr22:36626164-36667144:+	BX353756	AL834373	Homo sapiens mRNA; cDNA DKFZp762G0913 (from clone DKFZp762G0913).
Human	chr22:36664028-36674176:-	AK057349	AK057349	Homo sapiens cDNA FLJ32787 fis, clone TESTI2002264.
Mouse	chr15:79435455-79463457:+	M5C1005I09	M5C1005I09	contigs=179 ver=1 seqid=109861
Mouse	chr15:79455906-79467808:-	4932415B03	4932415B03	DNA segment, Human EST J0827E04
Human	chr22:37188567-37204780:+	AK129888	BC001277	Homo sapiens, KDEL (Lys-Asp-Glu-Leu) endoplasmic reticulum protein retention receptor 3, clone MGC:5099 IMAGE:3462392, mRNA, complete cds.
Human	chr22:37203920-37226845:-	CR594526	BC029553	Homo sapiens, clone IMAGE:5263091, mRNA, partial cds.
Mouse	chr15:79844235-79857743:+	BF720229	9430028D08	KDEL (Lys-Asp-Glu-Leu) endoplasmic reticulum protein retention receptor 3
Mouse	chr15:79855528-79874553:-	BC038378	I830022F15	Probable RNA-dependent helicase p72 (DEAD-box protein p72) (DEAD-box protein 17) homolog [Homo sapiens]
Human	chr22:39925709-39952036:+	BC017191	AK056443	Homo sapiens cDNA FLJ31881 fis, clone NT2RP7002829, weakly similar to Scm-related gene containing four mbt domains.
Human	chr22:39950014-39961438:-	BC048421	BC068590	Homo sapiens hypothetical protein BC012882, mRNA (cDNA clone IMAGE:4819656), partial cds.
Mouse	chr15:82001256-82025783:+	BC023933	2810004A18	l(3)mbt-like 2 (Drosophila)
Mouse	chr15:82015764-82031680:-	D930017K21	D930017K21	hypothetical Cysteine-rich flanking region, C-terminal/Leucine-rich repeat/Leucine-rich repeat, typical subtype containing protein
Human	chr22:40189621-40249497:+	BC014092	BC014092	Homo sapiens aconitase 2, mitochondrial, mRNA (cDNA clone MGC:20605 IMAGE:4328775), complete cds.
Human	chr22:40246303-40265246:-	AK026524	AK026524	Homo sapiens cDNA: FLJ22871 fis, clone KAT02533.
Mouse	chr15:81959463-82252917:+	BC004645	BC004645	Mus musculus aconitase 2, mitochondrial, mRNA (cDNA clone MGC:7146 IMAGE:3158239), complete cds. CDS=77..2419
Mouse	chr15:82225949-82264019:-	6720482J09	6720482J09	RIKEN cDNA 5031409G22 gene

Human	chr22:40935338-40935946:+	BX104608	-	-
Human	chr22:40880517-40935371:-	BU177253	AB006630	Homo sapiens mRNA for KIAA0292 gene, partial cds.
Mouse	chr15:83211036-83217647:+	4931406O09	4931406O09	unclassifiable
Mouse	chr15:83166624-83345575:-	AY007594	AY007594	Mus musculus stromelysin-1 PDGF-responsive element binding protein (Spbp) mRNA, complete cds. CDS=186..6083
Human	chr22:41221083-41294889:+	AL450314	BC041802	Homo sapiens, Similar to kraken-like, clone MGC:41798 IMAGE:5299234, mRNA, complete cds.
Human	chr22:41222528-41302542:-	AK098201	AK098201	Homo sapiens cDNA FLJ40882 fis, clone UTERU2000424.
Mouse	chr15:83447199-83474368:+	0610008B10	BC064069	Mus musculus cDNA clone IMAGE:1447084, partial cds.
Mouse	chr15:83473539-83480494:-	1110014J01	E430018B18	RIKEN cDNA 1110014J01 gene
Human	chr22:41221083-41294889:+	AL450314	BC041802	Homo sapiens, Similar to kraken-like, clone MGC:41798 IMAGE:5299234, mRNA, complete cds.
Human	chr22:41222528-41302542:-	AK098201	AK098201	Homo sapiens cDNA FLJ40882 fis, clone UTERU2000424.
Mouse	chr15:83447199-83474368:+	BC064069	BC064069	Mus musculus cDNA clone IMAGE:1447084, partial cds.
Mouse	chr15:83459765-83463622:-	CF105313	-	-
Human	chr22:45076880-45077680:+	BM682632	-	-
Human	chr22:45077250-45253586:-	AF231024	AF231024	Homo sapiens protocadherin Flamingo 2 (FMI2) mRNA, complete cds.
Mouse	chr15:86236981-86256896:+	3221401N04	AY349617	Mus musculus 5-methylaminomethyl-2-thiouridylate-methyltransferase mRNA, complete cds; nuclear gene for mitochondrial product. CDS=58..1311
Mouse	chr15:86256801-86392745:-	AF031572	AF031572	Mus musculus seven-pass transmembrane receptor precursor (Celsr1) mRNA, complete cds. CDS=8..9112
Human	chr22:49236789-49253353:+	AW246550	BC001298	Homo sapiens, Similar to hypothetical protein 384D8_6, clone MGC:5305 IMAGE:3459027, mRNA, complete cds.
Human	chr22:49252141-49261153:-	AL021683	CR622550	full-length cDNA clone CS0DE009YM06 of Placenta of Homo sapiens (human).
Mouse	chr15:89748386-89826632:+	D630044C19	BC019422	Mus musculus, clone IMAGE:4039831, mRNA.
Mouse	chr15:89764297-89769704:-	BC019554	AB060274	Mus musculus mRNA for thymidine phosphorylase, complete cds. CDS=70..1485
Human	chrX:16564212-16622299:+	AK123142	AK123142	Homo sapiens cDNA FLJ41147 fis, clone BRACE2037295.
Human	chrX:16617063-16725851:-	AI570582	X72841	H.sapiens IEF 7442 mRNA.
Mouse	chrX:152748769-152799275:-	4932441K18	4932441K18	similar to LIPOPOLYSACCHARIDE SPECIFIC RESPONSE-5 PROTEIN (FRAGMENT) [Homo sapiens]
Mouse	chrX:152730225-152748951:+	C430005J07	C430005J07	retinoblastoma binding protein 7
Human	chrX:19121673-19139482:+	CR592178	CR609764	full-length cDNA clone CS0DF009YJ14 of Fetal brain of Homo sapiens (human).
Human	chrX:19137828-19260051:-	AK131477	AK131477	Homo sapiens cDNA FLJ16645 fis, clone TESTI4031745, moderately similar to Mitogen-activated protein kinase kinase kinase 5 (EC 2.7.1.-).
Mouse	chrX:150092062-150108283:-	I920176E04	I920176E04	pyruvate dehydrogenase E1 alpha 1
Mouse	chrX:149971879-150093378:+	BC031147	BC031147	Mus musculus cDNA sequence BC031147, mRNA (cDNA clone IMAGE:4975522), containing frame-shift errors.
Human	chrX:38416912-38422173:+	BC008908	BC008908	Homo sapiens hypothetical protein STRAIT11499, mRNA (cDNA clone MGC:15174 IMAGE:3638510), complete cds.
Human	chrX:38417039-38419354:-	AW470852	-	-
Mouse	chrX:9046734-9051425:+	I420047E24	I420047E24	hypothetical protein
Mouse	chrX:9038897-9050648:-	B930087C06	A230044A09	hypothetical protein
Human	chrX:40350906-40354207:+	BC039399	BC039399	Homo sapiens, clone IMAGE:5300185, mRNA.
Human	chrX:40245435-40351584:-	AB006651	AB006651	Homo sapiens EXLM1 mRNA, complete cds.
Mouse	chrX:11093720-11153213:+	E030048B20	9630027L18	unclassifiable
Mouse	chrX:11006655-11093794:-	BC065072	BC065072	Mus musculus cofactor required for Sp1 transcriptional activation subunit 2, mRNA (cDNA clone MGC:76389 IMAGE:6830820), complete cds. CDS=375..2687
Human	chrX:46215114-46215644:+	AI580817	-	-
Human	chrX:46214935-46219220:-	AK096921	AK096921	Homo sapiens cDNA FLJ39602 fis, clone SKNSH2005061.
Mouse	chrX:18391267-18431710:+	9330156B14	9330156B14	carbohydrate (N-acetylglucosamino) sulfotransferase 7
Mouse	chrX:18430572-18623528:-	6330509M05	A530087D17	NONSELECTIVE SODIUM POTASSIUM/PROTON EXCHANGER homolog [Homo sapiens]
Human	chrX:46760506-46802468:+	CN297919	BC008733	Homo sapiens RNA binding motif protein 10, transcript variant 1, mRNA (cDNA clone MGC:997 IMAGE:3163064), complete cds.
Human	chrX:46757866-46761157:-	AY359056	AY359056	Homo sapiens clone DNA59827 AAGL111 (UNQ111) mRNA, complete cds.
Mouse	chrX:18949224-18982626:+	A630065L18	A630065L18	RNA binding motif protein 10
Mouse	chrX:18947045-18949319:-	BQ445367	BC027265	Mus musculus nuclear protein 15.6, mRNA (cDNA clone MGC:27951 IMAGE:3589704), complete cds. CDS=352..753
Human	chrX:48311381-48323657:+	AF019968	AF019968	Homo sapiens Su(var)3-9 homolog (SUV39H) mRNA, complete cds.
Human	chrX:48313098-48351816:-	BE302996	-	-
Mouse	chrX:6350151-6363739:-	AF019969	E430001D09	
Mouse	chrX:6340566-6362092:+	2410038P04	2410038P04	suppressor of variegation 3-9 homolog 1 (Drosophila)
Human	chrX:48784552-48787952:+	CR619342	CR598083	full-length cDNA clone CS0DK011YK15 of HeLa cells Cot 25-normalized of Homo sapiens (human).
Human	chrX:48787522-48799216:-	AI382128	BC016856	Homo sapiens, LIM domain only 6, clone MGC:17145 IMAGE:3846372, mRNA, complete cds.
Mouse	chrX:5956920-5960369:-	BC030318	F630011A01	proteolipid protein 2
Mouse	chrX:5946358-5957826:+	D030068I23	BC023671	Mus musculus LIM domain only 6, mRNA (cDNA clone IMAGE:5039859), partial cds. CDS=2..1951

Human	chrX:48848304-48863358:+	BC000972	BC000972	Homo sapiens, JM1 protein, clone MGC:5137 IMAGE:3449051, mRNA, complete cds.
Human	chrX:48863268-48863768:-	BM685299	-	-
Mouse	chrX:5846279-5894496:-	D430019A04	D430019A04	protein phosphatase 1, regulatory (inhibitor) subunit 3F
Mouse	chrX:5862579-5884222:+	F830216M24	F830216M24	forkhead box P3
Human	chrX:54349846-54354954:+	U92980	U92980	Homo sapiens clone DT1P1A10 mRNA, CAG repeat region.
Human	chrX:54354908-54405620:-	BC034530	U11690	Human faciogenital dysplasia (FGD1) mRNA, complete cds.
Mouse	chrX:140974544-140983994:-	A330091M19	BC049668	Mus musculus RIKEN cDNA 2310007F12 gene, mRNA (cDNA clone MGC:58494 IMAGE:6535058), complete cds. CDS=17..592
Mouse	chrX:140934341-140977136:+	U22325	U22325	Mus musculus faciogenital dysplasis (Fgd1) mRNA, complete cds. CDS=105..2987
Human	chrX:54355894-54357495:+	BI758673	-	-
Human	chrX:54354908-54405620:-	U11690	U11690	Human faciogenital dysplasia (FGD1) mRNA, complete cds.
Mouse	chrX:140974544-140983994:-	A330091M19	BC049668	Mus musculus RIKEN cDNA 2310007F12 gene, mRNA (cDNA clone MGC:58494 IMAGE:6535058), complete cds. CDS=17..592
Mouse	chrX:140934341-140977136:+	U22325	U22325	Mus musculus faciogenital dysplasis (Fgd1) mRNA, complete cds. CDS=105..2987
Human	chrX:99881493-99904668:+	AI026698	CR594312	full-length cDNA clone CS0DL004YH18 of B cells (Ramos cell line) Cot 25-normalized of Homo sapiens (human).
Human	chrX:99904458-99935479:-	AF127763	AF127763	Homo sapiens mitogenic oxidase mRNA, complete cds.
Mouse	chrX:124066324-124093975:+	E430008L21	M5C1042B07	cleavage stimulation factor, 3' pre-RNA subunit 2
Mouse	chrX:124093569-124118916:-	9030203P20	9030203P20	NADPH oxidase 1
Human	chrX:118811682-118836565:+	AF514781	BC066357	Homo sapiens A-kinase anchoring protein 28, mRNA (cDNA clone MGC:87683 IMAGE:5295545), complete cds.
Human	chrX:118836512-118838685:-	AA131530	-	-
Mouse	chrX:29694753-29712899:-	4930581F07	4930581F07	similar to Testis-specific A-kinase-anchoring-protein [Rattus norvegicus]
Mouse	chrX:29670820-29694803:+	A130096L18	A130096L18	hypothetical Arginine-rich region/Serine-rich region/Lysine-rich region containing protein
