# Supplementary material for: Complex Loci in Human and Mouse Genomes
Source: PLoS Genet. 2006 Apr 28;2(4):e47. doi: 10.1371/journal.pgen.0020047 (PMC1449890; doi:10.1371/journal.pgen.0020047)
Supplement: Table S3 — (25 KB PDF) [file pgen.0020047.st003.pdf]

Table S3. Mouse-human positional equivalents detected among cis-antisense and bidirectionally promoted pairs by manual curation

Nr	MOUSE								HUMAN				
	Type	Conservation in cis-AS overlap (BL2SEQ)	TU ID	Genbank accession	Longest ORF	, aa	Identity or homology to other proteins (outside of interspersed repeats)	Rodent-specific repeats (B1 - B4)	Genbank accession	Longest ORF	, aa	Identity or homology to other proteins (outside of interspersed repeats)	Primate-specific repeats (Alu, MER1)
1	diverg-asp	No	1737	AK020967	+1	58	No	Yes	BE218154	-1	35	No	Yes
2	diverg-asp	Yes	**7588**	BY233094	+2	62	No	No	BG031574	+2	140	No	No
		No		AK041861			No	Yes					
3	diverg-asp	No	**18192**	BY734689	+2	46	No	No	BC032908	+1	58	No	Yes
4	diverg-asp	No	27201	AK043809	+3	96	No	No	AI685287	-3	79	homology to PREDICTED: carbonyl reductase 1 [Pan troglodytes] (84%) **and excellent carbonyl reductase multispecies homo**logies	No
5	diverg-asp	No	39876	NM_176900	+3	111	identity to LOC319362 **but no domain or real protein hits whatsoever**	Yes	**CR456603**	+1	136	homology to PREDICTED: hypothetical protein XP_530407 [Pan troglodytes] (94%) **but no real protein homolog**ies	No
6	diverg-asp	No	5155	BU961841	+2	96	No	No	BC012108	+1	52	No	Yes
7	diverg-asp	No	16020	AK015655	+2	81	No	Yes	BG718987	+2	64	No	No
		No							BM715436			No	No
8	diverg-asp	No	**24444**	AI429080	-3	61	No	Yes	AA025061	-3	34	No	No
9	bdp	No	**2006**	BU938475	+2	143	homology to unnamed protein (92%) **but no nonmouse hits**	No	BM464182	+1	105	No	Yes
		No							AW082177			n/a	No
10	bdp	No	14425	AK006945	0	n/a	n/a	No	AI498684	-2	54	No	No
11	bdp	No	15308	BB650455			n/a	Yes	**CR615110**	+2	75	No	Yes
		No		BB380155	-3	38	No	Yes					
12	bdp	No	17672	CN834920	+3	58	No	No	CB956378	+3	45	No	Yes
13	bdp	No	22277	AK077230	+1	62	No	Yes	CA396688	0	n/a	n/a	No
14	bdp	No	22793	AK033299	+3	46	No	Yes	AI498106	-2	66	No	No
15	bdp	No	31986	AK007271	+3	35	No	Yes	AA974494	-2	51	No	No
16	bdp	No	33790	BY750856	+2	63	No	No	**BI831438**	+3	84	No	No
		No		BY558636			No	No					
17	bdp	No	38155	AK020774	+3	42	No	No	CA305921	-1	200	No	No
18	bdp	No	39817	CK621361	+1	64	No	No	BU955825	+2	225	No	No
19	bdp	No	2972	BE955486	-3	65	No	Yes	AW014905	-3	82	No	No
20	bdp	No	28046	AK015582	+1	63	No	No	AI913482	-2	43	No	No
21	bdp	No	38895	AK020472	+1	55	No	Yes	AK095144	+3	104	No	Yes
22	bdp	No	34018	AK088170			No	No	BE798791	+1	144	No	No
		No		CF735676	+3	55	No	Yes	BI226068			No	Yes - **AluYc (human-specific?)**
23	bdp	No	32346	AK043240	+2	96	No	Yes	AL709003	+1	43	No	No
24	bdp	No	27079	BY730100	+1	79	No	No	**BC004343**	+1	136	TRAP-delta conserved domain;homology to TRAP-like protein precursor (clone M0286) - mouse [Pan troglodytes] (78%)	Yes
		No		BB463025			n/a	No	**BQ950030**			TRAP-delta conserved domain; identity to PREDICTED: similar to TRAP-like protein precursor (clone M0286) - mouse [Pan troglodytes]	No
25	bdp	No	7198	AK042399	+3	70	No	Yes	BI916802			No	No
		No							BF685776	+3	53	No	Yes
26	conv-asp	Yes	7635	AK076583	+3	61	No	No	BC058910	+3	98	No	No
27	conv-asp	No	10922	CN684178	+3	75	No	No	AA926773	-1	37	No	No
28	conv-asp	No	17140	BY726083	+1	39	No	Yes	BU674890	-3	42	No	No
				BB289010	-	-	-	Yes					
29	conv-asp	No	**18118**	BB843761	+2	45	No	No	**BQ017586**	-3	36	No	No
		No		BB738597	-3	42	No	Yes	**BM999219**	-3	49	No	No
30	conv-asp	Yes	**24321**	BB483251	-	-	-	No	AK127717	+1	161	identity to hypothetical gene supported by AK127717 [Homo sapiens]	No
31	conv-asp	No	35396	AK029557	+1	87	No	Yes	BM988348	-	-	-	No
		No							CA440001	-3	94	No	Yes
32	conv-asp	No	22630	AV271548	-	-	-	No	BF031909	+3	101	No	Yes
33	conv-asp	No	35716	CF198316	+1	116	homology to PREDICTED: hypothetical protein XP_355991 [Mus musculus]	No	AA496145	-2	108	homology to PREDICTED: hypothetical protein XP_530046 [Pan troglodytes]	No

(green)	spliced
**BOLD**	> 5 ESTs
