# Supplementary material for: Complex Loci in Human and Mouse Genomes
Source: PLoS Genet. 2006 Apr 28;2(4):e47. doi: 10.1371/journal.pgen.0020047 (PMC1449890; doi:10.1371/journal.pgen.0020047)
Supplement: Table S4 — (25 KB PDF) [file pgen.0020047.st004.pdf]

Table S4. *Cis*-antisense pairs with significantly correlated expression between pair members.

Plus strand gene				Minus strand gene				Conserved noncoding overlap[b]	Expression correlation	
Gene symbol	GNF1M probeset id	Overlap location	3'-TSS evidence[a]	Gene symbol	GNF1M probeset id	Overlap location	3'-TSS evidence[a]		Spearman correlation	Raw p-value
Ppp1ca	gnf1m05688_a_at	3'-UTR	yes	Rad9	gnf1m02706_a_at	3'-UTR	no	yes	0.71	<1E-08
Ncx5	gnf1m17670_a_at	CDS, 3'-UTR	no	Myef2	gnf1m15371_s_at	3'-UTR	yes	yes	0.82	<1E-08
Ilf2	gnf1m11737_a_at	3'-UTR	yes	Snapap	gnf1m11693_a_at	3'-UTR	no	no	0.81	<1E-08
Rhpn2	gnf1m07630_a_at	3-'UTR	yes	AK082457*	gnf1m10091_a_at	3'-UTR	no	yes	0.61	3.5E-07
Rpgrip1	gnf1m04381_a_at	3'-UTR	no	Supt16h	gnf1m05768_s_at	CDS, 3'-UTR	no	yes	0.59	8.0E-07
Capn12	gnf1m04176_s_at	3'-UTR	no	Actn4	gnf1m04177_a_at	3'UTR	yes	yes	0.58	1.8E-06
AK014383*	gnf1m29598_a_at	3'-UTR	no	AK076393**	gnf1m18166_at	n/a	no	no	0.56	4.0E-06
Gca	gnf1m07803_a_at	3'-UTR	no	AK084685**	gnf1m18215_a_at	n/a	no	yes***	0.55	6.5E-06
Rpe	gnf1m04646_a_at	3'-UTR	yes	AK082897*	gnf1m07548_a_at	3'-UTR	yes	yes***	0.54	9.8E-06
AK050988**	gnf1m30627_a_at	n/a	yes	Whsc2	gnf1m29717_at	3'-UTR	yes	yes	0.52	2.2E-05
AK017634**	gnf1m16840_at	n/a	no	Tra1	gnf1m02888_a_at	CDS	yes	n/a	0.50	4.6E-05
Dalrd3	gnf1m09988_a_at	CDS, 3'-UTR	no	Wdr6	gnf1m05671_a_at	3'-UTR	yes	yes	0.50	5.3E-05
Stk16	gnf1m11575_at	3'-UTR	no	Tuba4	gnf1m11816_a_at	3'-UTR	yes	yes	0.49	7.7E-05
AK033311**	gnf1m17949_at	n/a	no	Gng2	gnf1m23132_s_at	3'-UTR	yes	no	0.48	9.7E-05
Dnajb8	gnf1m03870_a_at	CDS	no	AK006579*	gnf1m15462_at	CDS, UTRs	no	n/a	0.48	1.2E-04
Lrrc40	gnf1m11975_at	3'-UTR	no	AK032571**	gnf1m18098_at	n/a	yes	yes***	0.47	1.8E-04
Gmpr2	gnf1m10152_a_at	CDS, 3'-UTR	yes	Tinf2	gnf1m23574_a_at	3'-UTR	no	no	0.46	1.9E-04

[a] Evidence of transcriptional start sites (TSSs) at 3'-ends of transcripts was assessed using the FANTOM3 Genomic Element Viewer (Carninci et al., Science 309:1559) to search for CAGE tag clusters directly following a sequence of three guanines. TSSs at 3'-ends of transcripts were described recently (Carninci et al., Science 309:1559) and found to be predominantly preceded by three G's at positions -3 to -1 (Carninci et al., submitted), which was also the case for all of the putative CAGE-supported 3'-end TSS reported here. There is ample evidence that transcription start sites in 3' UTRs are not due to truncated transcripts or some sequence related artifacts, since they are associated with a specific motif, appear only at 3' UTRs with this motif, which is often not the 3'-most motif of its kind nor is it used for transcription start outside 3' UTRs, and the sequence downstream of shows elevated conservation between human and mouse (Carninci et al., submitted).

[b] Conservation was qualitatively assessed by studying the Mouse/Rat/Human/Dog/Chicken conservation track in the UCSC Genome Browser (http://genome.ucsc.edu).

* hypothetical protein
** putative noncoding
*** conservation clearly limited to the region of overlap between pair members
