# Supplementary material for: Complex Loci in Human and Mouse Genomes
Source: PLoS Genet. 2006 Apr 28;2(4):e47. doi: 10.1371/journal.pgen.0020047 (PMC1449890; doi:10.1371/journal.pgen.0020047)
Supplement: Table S5 — (17 KB PDF) [file pgen.0020047.st005.pdf]

Table S5 Measures of gene density around cis-antisense pairs and TUs not involved in cis-antisense pairs.

		Characteristics of flanking regions (100 kb on each side)					
		Number of TUs (in 2 x 100 kb)		Fraction covered by TUs		Fraction covered by exons	
		Mean	Median	Mean	Median	Mean	Median
Mouse	Cis-antisense pair	7.75	7	57.1%	59.8%	10.64%	9.60%
	Remaining TUs	6.07	5	52.9%	55.3%	8.00%	6.70%
	Ratio	1.28	1.40	1.08	1.08	1.33	1.43
Human	Cis-antisense pair	7.45	7	60.9%	62.9%	9.74%	8.50%
	Remaining TUs	6.10	5	57.1%	59.9%	7.67%	6.40%
	Ratio	1.22	1.40	1.07	1.05	1.27	1.33
