# Supplementary material for: Complex Loci in Human and Mouse Genomes
Source: PLoS Genet. 2006 Apr 28;2(4):e47. doi: 10.1371/journal.pgen.0020047 (PMC1449890; doi:10.1371/journal.pgen.0020047)
Supplement: Table S6 — (18 KB PDF) [file pgen.0020047.st006.pdf]

Table S6 Conservation of transcriptional regulator-containing chains by manual curation

A. Four-category classification of TU conservation

		Exonic sequence of mouse TU	
		Conserved in human genome	Not conserved in human genome
TU at equivalent / orthologous genomic location	Present in human	True ortholog (n=662)	Positional equivalent (n=9)
	Absent in human	* (n=207)	** (n=7)

* TUs whose exons localize to genomic sequences conserved between mouse and human, despite absence of exons or comparable TUs at the orthologous human locations. Observed due to insufficient human EST/cDNA coverage masking the existence of low-expression orthologs; bona-fide rodent-specific genes arising in genomically conserved regions; and/or human ortholog EST/cDNA coverage failure to meet our standards for mapping quality.

** TUs whose exons localize to genomic sequences lacking human conservation. Observed due to insufficient human EST/cDNA coverage masking the existence of low-expression positional equivalents; bona-fide rodent-specific genes arising in rodent-specific genomic sequences; and/or human positional equivalent EST/cDNA coverage failure to meet our standards for mapping quality.

B. Chain conservation

One or more TUs have nonconserved exon sequence	15
All TUs are conserved in sequence, but some lack corresponding human transcript sequences	148
All TUs have well-defined orthologs	71
Total number of curated chains	234

Possible *cis*-antisense regulation of transcription factors would have an impact on their downstream target genes and thus influence the entire regulatory cascade of fundamental developmental and differentiation processes that those transcription factors regulate in vertebrates. To identify a large set of chains containing transcriptional regulators, we searched for mouse chains with transcripts represented in the Riken Transcription Factor Database of direct and indirect transcriptional regulators [1]. This search produced 234 chains containing transcriptional regulators (TR). For precise characterization of sequence conservation, structure conservation, and TU content at orthologous loci, we manually curated all TR-containing chains and introduced the 4-category classification outlined in Table A.

Each TU in a TR-containing chain was manually classified by viewing it in a comparative genome browser that was developed specifically for this study. The browser displays annotated human and mouse genomic regions side by side, by associating them through BLASTZ net alignments. “Conservation” was defined at the genomic level as alignability (using BLASTZ net alignments) of a sequence block between mouse and human, and at the transcript level as presence of genes with equivalent genomic footprints (starts, splice sites, ends) within one or more genomic conserved blocks between mouse and human. For putative positional equivalents, the following additional validation was performed: 1) visualization of both human and mouse loci in the UCSC Genome Browser [2]; 2) confirmation that pairs were not affected by assembly gaps or extensive duplications; and 3) BL2SEQ alignment [3] of every transcript supporting the mouse TU against every transcript supporting the existence of a human positional equivalent, and manual assessment of results, to confirm lack of exonic conservation.

At least 75% (662/885) of curated mouse TUs have human orthologs, and another 23% (207/885) have likely human orthologs for which transcripts have not been sequenced (Table A), suggesting that chain *members* are typically well conserved between human and mouse. Seventy-one mouse chains consisted entirely of TUs with well-defined human orthologs (Table B). However, the genomic structures of these 71 mouse chains were generally not conserved in human, suggesting lack of an intrinsic relationship between sequence conservation of chain member TUs and structure conservation of the chains. Only 7 (10%) of the 71 chains had fully conserved structures between human and mouse (identical patterns of genomic footprints, *cis*-antisense overlaps, and bidirectional promoter locations through the entire chain in both species); the number increased to 13 if we also counted bidirectional promoters in one species with head-to-head *cis*-antisense at the orthologous location in the other species. If the organization of TUs into chain structures has a functional importance, as we have previously hypothesized [4], these 13 fully conserved chains are likely to be under the greatest functional constraints of all chains examined, due to their rare combination of sequence and structure conservation. TRs in those highly conserved chains included estrogen-related receptor alpha, *Taf10*, cyclin T2, *Tbx2* and *Foxh1*.

1. Kanamori M, Konno H, Osato N, Kawai J, Hayashizaki Y, et al. (2004) A genome-wide and nonredundant mouse transcription factor database. Biochem Biophys Res Commun 322: 787-793.

2. Karolchik D, Baertsch R, Diekhans M, Furey TS, Hinrichs A, et al. (2003) The UCSC Genome Browser Database. Nucleic Acids Res 31: 51-54.
3. Tatusova TA, Madden TL (1999) BLAST 2 Sequences, a new tool for comparing protein and nucleotide sequences. FEMS Microbiol Lett 174: 247-250.
4. Lipovich L, King MC (2003) Novel transcriptional units and unconventional gene pairs in the human genome: toward a sequence-level basis for primate-specific phenotypes? Cold Spring Harb Symp Quant Biol 68: 461-470.
