# Supplementary material for: Complex Loci in Human and Mouse Genomes
Source: PLoS Genet. 2006 Apr 28;2(4):e47. doi: 10.1371/journal.pgen.0020047 (PMC1449890; doi:10.1371/journal.pgen.0020047)
Supplement: Table S7 — (19 KB PDF) [file pgen.0020047.st007.pdf]

Supplementary table S7 PCR primer sequences

A. Primer pairs for orientation-specific RT-PCR

	**Symbol**	**cDNA or EST accession nrs.**	**Product size**	**Sense primer**	**Antisense primer**
**Controls**	Eif3s6	NM_008388	106	AGTCTCTTCAGCAGCGAACAT	TGTGGCTGGTTAAGGAAGAGA
	Psmb6	AK132042	139	CCATTCAGGAGTCAGGGGTA	TAGCTTCTGCGTCGGTATGG
	Eef1g	NM_026007	151	GTTTGCAGAGAGCCAGCCTA	CAATGCCTGCTCACACTCAT
	Rpl18	NM_009077	164	CTCAAGGGTGGGGGTAAGAT	CGGACATAGGGTTTGGTTTG
	Rps27	NM_027015	113	ATGCCTCTCGCAAAGGATCT	GGGCATTTCACGTCCATAAA
	Rps27	NM_027015	120	TTAGCCATGCACAAACGGTA	GCTTTCAGTGCTGCTTCCTC
**Candidates**	1	BB653062;BB653244	190	CTGCGAGTGGACCTGGAC	GCCTAGTTCGCGCATAAAGT
	2	AK049815;CJ108710	108	ACTGGGACCATTTCCCAATC	AATATTCCGCTTTCGTGGTG
	3	CB232677;CJ117842	128	CCTGACATGGAAAACTGCAC	AGGTCACAAACGCTGAAGGT
	4	BB301747;AK134745	104	GGACAGCAAAACCCACACTG	TGTCCTGTGCACTGACTGGT
	5	AK141573;AK046211	202	ATCAGCCTTGACCGCTACTG	TCTGGTCGTTGATCTTGCAG
	6	AB041648;AK138162	145	CTTGGGAAAAGCCACCCTAT	GCCTTATCACAATCGCTCCA
	7	AK005277;AV339423	158	TCATCGACAACTGTGCCTTC	AGCCTCATCCTCTGTGATGG
	8	AK032006;AK083199	181	TGTACGGGCAGAACATCAAC	AGAATGATGTGGGGCAAGAT
	9	AK122577;AV339355	201	GCGGATCTGCAAAGACCTAC	GGACATTCAGGATTTCCAGGT
	10	AK032287;BB622382	150	GTGTGGCCTGTCTTCCACTT	CCTTCCCTGCTATCAAGGTG
	11	AU079131;BY123968	151	GCTCATCCAGTTCCATCTCA	GGAGGAGGAGGGGACTACAA
	12	AK083178;AK032490	149	CCAACATCCTCCCAAACATT	GGCAGTTCATGAGTGAGCAA
	13	AK038921;AK045416	147	GACATCTGTTGGGTGGGTTC	GGTGGTGACACAGAAGCTGA
	14	AK083132;AK162418	110	GGTTGGAGAAATGGCTTCAT	GCTTTCCTTGTGAAAACACTTG
	15	AK162869;BB178576	175	GGGCATTAACCCGGTCATTA	TGGACATCACAAGTCCTGCT
	16	CJ050083;AK034247	144	CCGAGCATCCCTTGAGAATA	CGCAAGATGGGAATTCAGAC
	17	AK129391;BB647791	137	TGCCTACATGGTGTCACCTC	CAGAGCGGAACAGGCTAAAG
	18	AK078282;AK134912	166	GCCATGGTAAAGCCCATGTA	AGAAAGAGGGCAGCCAAATC
	19	AU051773;AK134570	150	CCGCTTGAAGTCGCTGAA	GTCTTCACCTGGGTCTACCG
	20	BY130470;BB632028	154	GGAGCTCTGCACTGCTTCTT	TCACACGCTCACCCTGAATA

B. Primer pairs for quantitative real-time PCR

**Gene**	**Primer pair id**	**Product size**	**Forward primer**	**Reverse primer**
**Ddx49**	A1	160	GCCAACGTCCTCTCACAACT	CACTGTTCTACCAGCCACGA
	A2	115	GGACGTGACGTAGCATTCAC	CACTGTTCTACCAGCCACGA
	A3	150	TGGCTGGTAGAACAGTGTCG	CAAGATGGGAAGCACAAAGG
	A4	150	ATTCCTGGTGATGGATGAGG	TGCAGCTCCTTGAGTGTGTC
**Cope**	B1	145	ATAGTTCTCGGGAGGCTTTG	ATGTCACCGCACCTCTAACC
	B2	151	GCCTACGTGTCCCAGAAGAA	CGTTGATGCACTGCTGGTAG
	B3	175	GTGTCCCAGAAGAAGGTTGG	CAGGACTGGAGAGCTTCACG
	B4	141	AGCTACCAGCAGTGCATCAA	GGAGGGTTTGATCTCATCCA
	B5	155	GAGCAGGAGTGTGGATGTGA	CCAGCTTGAGGAGGATCTGA
